# Combining phylogenetic footprinting with motif models incorporating intra-motif dependencies

**DOI:** 10.1186/s12859-017-1495-1

**Published:** 2017-03-01

**Authors:** Martin Nettling, Hendrik Treutler, Jesus Cerquides, Ivo Grosse

**Affiliations:** 1 Institute of Computer Science, Martin Luther University Halle-Wittenberg, Halle, Germany; 2 Leibniz Institute of Plant Biochemistry, Halle, Germany; 3 Institut d’Investigació en Intel ·ligència Artificial, IIIA-CSIC, Campus UAB, Cerdanyola, Spain; 4 German Centre for Integrative Biodiversity Research (iDiv) Halle-Jena-Leipzig, Leipzig, Germany

**Keywords:** ChIP-Seq, Phylogenetic footprinting, Evolution, Transcription factor binding sites, Gene regulation

## Abstract

**Background:**

Transcriptional gene regulation is a fundamental process in nature, and the experimental and computational investigation of DNA binding motifs and their binding sites is a prerequisite for elucidating this process. Approaches for de-novo motif discovery can be subdivided in phylogenetic footprinting that takes into account phylogenetic dependencies in aligned sequences of more than one species and non-phylogenetic approaches based on sequences from only one species that typically take into account intra-motif dependencies. It has been shown that modeling (i) phylogenetic dependencies as well as (ii) intra-motif dependencies separately improves de-novo motif discovery, but there is no approach capable of modeling both (i) and (ii) simultaneously.

**Results:**

Here, we present an approach for de-novo motif discovery that combines phylogenetic footprinting with motif models capable of taking into account intra-motif dependencies. We study the degree of intra-motif dependencies inferred by this approach from ChIP-seq data of 35 transcription factors. We find that significant intra-motif dependencies of orders 1 and 2 are present in all 35 datasets and that intra-motif dependencies of order 2 are typically stronger than those of order 1. We also find that the presented approach improves the classification performance of phylogenetic footprinting in all 35 datasets and that incorporating intra-motif dependencies of order 2 yields a higher classification performance than incorporating such dependencies of only order 1.

**Conclusion:**

Combining phylogenetic footprinting with motif models incorporating intra-motif dependencies leads to an improved performance in the classification of transcription factor binding sites. This may advance our understanding of transcriptional gene regulation and its evolution.

**Electronic supplementary material:**

The online version of this article (doi:10.1186/s12859-017-1495-1) contains supplementary material, which is available to authorized users.

## Background

Gene regulation is an essential process in every living organism that controls the activity of gene expression and enables the concerted up- and down-regulation of gene products. Gene regulation involves a wide range of sub-processes such as transcriptional regulation including DNA methylation [1], histon modifications [2], and promotor escaping [3] as well as post-transcriptional regulation including modulated mRNA decay [4], siRNA interference [5, 6], and alternative splicing [7, 8]. One important process in gene regulation is the interaction of transcription factors (TFs) with their corresponding transcription factor binding sites (TFBSs) [9, 10]. The algorithmic discovery of TFBSs and the simultaneous inference of their motifs is known as de-novo motif discovery and a challenging task in bioinformatics. Many different approaches exist for de-novo motif discovery, which can be divided in two groups.

The first group comprises approaches based on sequences of only one species, which we refer to as one-species approaches in this work, using statistical models for the binding of TFs to their TFBSs. One of the most popular motif models is the simple position weight matrix (PWM) model, which does not take into account any dependency between different positions of the same TFBS, but there are also more complex motif models that take into account intra-motif dependencies. Irrespective of the wide variety of different motif models used, all of these approaches have in common that they do not take into account phylogenetic information available from orthologous sequences of phylogenetically related species.

Complex motif models that take into account intra-motif dependencies have been shown to outperform simpler motif models like the PWM model [11–13]. Examples for highly popular tools that model intra-motif dependencies are *Dimont* [14], *MEME-ChIP* [15], *DeepBind* [16], and *diChIPMunk* [17].

In contrast, the second group of de-novo motif discovery approaches known as phylogenetic footprinting incorporates orthologous sequences of at least two phylogenetically related species. The basic idea of these approaches is that TFBSs tend to be subject to negative selection during evolution, which can increase the recognition of TFBSs in the reference species. Phylogenetic motif models, which model the binding of TFs to their TFBSs and their evolution simultaneously, are based on evolutionary models such as the popular Felsenstein model [18]. Irrespective of the wide variety of different phylogenetic motif models used, all of these approaches have in common that they do not take into account intra-motif dependencies.

Not all sequences from the reference species may have orthologous sequences in phylogenetically related species, and not all aligned sequences may comprise functional TFBSs at the same alignment positions [19]. Moreover, alignment errors, binding site turnovers, and spurious alignments from convergent evolution may affect the utility of phylogenetic footprinting. Nevertheless, phylogenetic footprinting has been shown to outperform one-species approaches for many TFs and have become increasingly attractive due to next generation sequencing and the resulting avalanche of data [20–22].

Examples for highly popular phylogenetic footprinting tools that have been applied to eukaryotes and prokaryotes are *FootPrinter* [23], *PhyME* [24], *MONKEY* [25], *MicroFootprinter* [26], *Phylogenetic Gibbs Sampler* [27], *PhyloGibbs* [28], *PhyloGibbs-MP* [29], or *MotEvo* [30].

In summary, one-species approaches neglect phylogenetic information, whereas phylogenetic footprinting, which incorporates this information, neglects intra-motif dependencies. The main objective of this work is to develop an approach that combines these two ideas and to investigate if taking into account intra-motif dependencies can improve phylogenetic footprinting. Specifically, we propose a simple phylogenetic footprinting model (PFM) capable of taking into account both intra-motif dependencies and phylogenetic information in Methods, and we study if modeling intra-motif dependencies improves phylogenetic footprinting based on human ChIP-Seq data of 35 TFs and more than 10^5^ multiple alignments of human ChIP-seq positive regions and their orthologous sequences of 9 mammalian species ranging from chimp to cow in Results.

## Methods

In this section we describe (i) the studied datasets, (ii) the used notation and the likelihood calculation of the PFM, (iii) the performance measure, (iv) the calculation of the mutual information, and (v) details regarding the estimation algorithm and implementation of the proposed model.

### Data

We use freely available ChIP-Seq data for 50 transcription factors from the ENCODE project [31, 32]. The ChIP-seq experiments were performed by several production groups in the ENCODE Consortium and analysed by the ENCODE Analysis Working Group based on a uniform processing pipeline developed for the ENCODE Integrative Analysis effort [33]. We focus on datasets for the human H1-hESC cell line. The uniform processing pipeline utilizes the SPP peak caller [34] and biological replicates (at least two per transcription factor) are analysed jointly with a Irreproducible Discovery Rate (IDR) score of at least 2*%*. The resulting ChIP-seq regions of the Uniform TFBS track reference the hg19 assembly [35] and each comprise the chromosome, start position, end position, and an enrichment score. We exclude 15 datasets which yield repetitive motifs analog to [13] and hence retain datasets of 35 TFs.

For each TFs we select the top 20*%* of the available ChIP-seq regions ranked by enrichment score. We denote these regions as ChIP-seq positive regions and use them as basis for the positive dataset (Additional file 1: Table S1 and Additional file 1: Section 1.3). We denote the regions between ChIP-seq positive regions on one chromosome as ChIP-seq negative regions. For each TF we extract two regions of length 500 bp from each ChIP-seq negative region centered at one third and two thirds, and use these as basis for the negative dataset. Hence, there are roughly twice as many negative regions than positive regions. We remove regions from the positive and the negative region sets that are shorter than 20 bp. For each region in the positive and negative region sets we extract the corresponding alignment consisting of 46 mammals using the freely available multiple genome alignment from UCSC [36].

We apply the following steps to each alignment. We remove alignment columns with gap-symbols or ambiguous symbols in the human sequence and concatenate the remaining alignment columns. We retain the 10 species with the best alignment coverage, namely Human (hg19), Chimp (panTro), Baboon (papHam), Orangutan (ponAbe), Rhesus (rheMac), Marmoset (calJac), Horse, (equCab), Dog (canFam), Gorilla (gorGor), and Cow (bosTau). We replace ambiguous symbols with gap-symbols. We remove all alignments which comprise no base symbols for 20% or more species. See Additional file 1: Table S1 for statistics on the number of ChIP-Seq positive regions and the number of extracted alignments and see Additional file 1: Table S2 for details about the origin of the used ChIP-Seq data and Additional file 2 contains all extracted alignments.

### Phylogenetic footprinting model

#### Notation

Each dataset of each TF contains *N* alignments, with each alignment containing *O* sequences (one per observed species). Of course the number of alignments per TF, *N*, varies from TF to TF (See Additional file 1: Table S1). The *n*-th alignment is denoted by *X*_*n*_ and its length is denoted by *L*_*n*_. Each sequence of alignment *X*_*n*_ is composed of *L*_*n*_ symbols. We denote by $X_{n}^{u,o}$ the *u*-th symbol of the *o*-th sequence of the *n*-th alignment. All symbols belong to the set ${\mathcal {A}} = \{A,C,G,T,-\}$ where *A,C,G*, and *T* denote the bases and − denotes a gap in the alignment. Missing sequences in alignment *n* are represented by *L*_*n*_ gap symbols.

An alignment *X*_*n*_ may or may not contain a binding site. This is encoded in the variable *M*_*n*_, with *M*_*n*_=0 indicating that alignment *X*_*n*_ does not contain a motif and *M*_*n*_=1 indicating that alignment *X*_*n*_ does contain a motif. This model is known as *ZOOPS* (zero or one occurrence of a binding site per sequence) or *NOOPS* (noisy OOPS) model. Due to its simplicity and its modularity this model is widely used for de-novo motif discovery [37–40].

#### Likelihood

The probability that the alignment *X*_*n*_ is generated by our PFM can be written as

1 $$\begin{array}{*{20}l} p\left(X_{n} | \theta\right) = &\, p\left(X_{n}|M_{n}=0,\theta\right)\cdot p\left(M_{n}=0|\theta\right) \\ & +p\left(X_{n}|M_{n}=1,\theta\right) \cdot p\left(M_{n}=1|\theta\right)  \end{array} $$

with variable *M*_*n*_ taking a Bernoulli distribution and *θ* denoting model parameters, namely (i) the topology of the phylogenetic tree, (ii) the substitution probabilities, and (iii) the evolutionary model with its stationary probabilities for the flanking regions as well as for the binding site regions.

We need to specify the probability for non-motif-bearing *p*(*X*_*n*_|*M*_*n*_=0,*θ*) and for motif-bearing alignments *p*(*X*_*n*_|*M*_*n*_=1,*θ*). For reasons of clarity we omit *θ* in the following.

#### Likelihood of a non-motif-bearing alignment

Since sequences are assumed to be conditionally independent, the probability of an alignment decomposes as the product of the probability of each of its sequences:

2 $$\begin{array}{*{20}l} p\left(X_{n}|M_{n}=0\right) & = \prod_{o=1}^{O}p\left(X_{n}^{.,o}| M_{n}=0\right) \end{array} $$

Now, the probability of each sequence follows a homogeneous Markov Chain of order *C*:

3 $$\begin{array}{*{20}l} p\left(X_{n}^{.,o}| M_{n}=0\right) = \prod_{u=1}^{L_{n}} p\left(X_{n}^{u,o} | X_{n}^{p(u,1),o},M_{n}=0\right), \end{array} $$

where *p*(*u,k*) stands for the (at most *C*) predecessors of the *u*-th base for a sequence starting at position *k*, namely the set *p*(*u,k*)={*v*| max(*k,u*−*C*)≤*v*<*u*}, and

4 $$\begin{array}{*{20}l} p\left(X_{n}^{u,o} = a | X_{n}^{p(u,1),o}=\zeta,M_{n}=0\right) = \pi_{0}^{a,\zeta} \end{array} $$

where $\pi _{0}^{a,\zeta }$ is the parameter encoding the probability of a base *a* in the background sequence provided that its predecessors are in joint state *ζ*.

#### Likelihood of a motif-bearing alignment

We note *W* for the length of the motif. Since the motif can be present in different positions, the probability of a motif-bearing assignment is a weighted sum over each possible motif position *ℓ*_*n*_:

5 $$\begin{array}{*{20}l} p\left(X_{n}|M_{n}=1\right) = & \sum_{\ell_{n}=1}^{L_{n}-W+1}p\left(X_{n}|\ell_{n}, M_{n}=1, \theta,\right)\\&\times p\left(\ell_{n}|M_{n}=1\right) \end{array} $$

We assume motifs to be uniformly distributed a priori, thus having that $p(\ell _{n}|M_{n}=1) = \frac {1}{L_{n}-W+1}$. Again, conditional independence of sequences allows to express probability of an alignment as a product of the probability of its single sequences

6 $$\begin{array}{*{20}l} p\left(X_{n}| \ell_{n}, M_{n}=1\right) = \prod_{o=1}^{O} p\left(X_{n}^{.,o},\ell_{n},M_{n}=1\right) \end{array} $$

And the probability of each single sequence breaks into three parts: (i) an initial non-motif bearing part containing bases *i*(*ℓ*_*n*_)={1,…,*ℓ*_*n*_−1}, (ii) the motif, containing bases *m*(*ℓ*_*n*_)={*ℓ*_*n*_,…,*ℓ*_*n*_+*W*−1} and (iii) a final non-motif bearing part formed by bases *e*(*ℓ*_*n*_)={*ℓ*_*n*_+*W*,…,*L*_*n*_} :

7 $$\begin{array}{*{20}l} p\left(X_{n}^{.,o}|\ell_{n},M_{n}=1\right) = &\, p\left(X_{n}^{i(\ell_{n}),o}|\ell_{n},M_{n}=1\right)\\ &\times\left(X_{n}^{m(\ell_{n}),o}|\ell_{n},M_{n}=1\right) \\ &\times p\left(X_{n}^{e(\ell_{n}),o}|\ell_{n},M_{n}=1\right) \end{array} $$

with the non-motif bearing parts following a homogeneous Markov Chain of order *C* as described above and the motif-bearing part following a non-homogeneous Markov Chain defined as

8 $$\begin{array}{*{20}l} &p\left(X_{n}^{m(\ell_{n}),o}|\ell_{n},M_{n}=1\right)\\ &\quad= \prod_{u\in m(\ell_{n})}p\left(X_{n}^{u,o} | X_{n}^{p(u,\ell_{n}),o},\ell_{n}, M_{n}=1\right), \end{array} $$

with

9 $$\begin{array}{*{20}l} p\left(X_{n}^{u,o} = a | X_{n}^{p(u,\ell_{n}),o}=\zeta,\ell_{n},M_{n}=0\right) = \pi_{u-\ell_{n}+1}^{a,\zeta} \end{array} $$

where $\pi _{w}^{a,\zeta }$ is a parameter that encodes the probability of a base *a*, at position *w* of the motif provided that its predecessors are in joint state *ζ*.

#### Management of gaps

A sequence may have gaps introduced by the alignment algorithm. We compute the probability of a gap by summing over all possible nucleotides at that position in that sequence. For example to assess $p\left (X_{n}^{u,o} = - | X_{n}^{p(u,1),o}=\zeta,M_{n}=0\!\!\!\!\!\!\!\!{\phantom {\sum ^{0}_{0}}}\right)$, we use $\sum _{a\in \{A,C,G,T\}}p\left (X_{n}^{u,o}= a | X_{n}^{p(u,1),o}=\zeta,M_{n}=0\!\!\!\!\!\!\!\!{\phantom {\sum ^{0}_{0}}}\right).$

The used model estimation procedure and the freely available implementation are specified in Methods 5, and run times are exemplified in Additional file 1: Section 1.6.

### Measuring classification performance

We evaluate all PFMs by a stratified repeated random sub-sampling validation by estimating all PFMs from a training set and measuring classification performance on a test set as follows.

In step 1, we generate two training sets and two disjoint test sets for each of the 35 transcription factors as follows. We randomly select 70*%* but maximal 1000 alignments from the set of alignments of a particular transcription factor as positive training set, and we choose the set of the remaining alignments but maximal 1000 as positive test set. We randomly select 70*%* but maximal 1000 alignments from the corresponding set of negative alignments of this transcription factor, and we choose the set of the remaining alignments but maximal 1000 as negative test set.

In step 2, we train a foreground model on the positive training set and a background model on the negative training set by expectation maximization [41] using a numerical optimization procedure in the maximization step. In all cases, we attempt to find a motif of length *W*=20 bp. It is known that the motifs of many TFs have a length smaller than *W* bp, but adding some possibly uninformative positions in case of short motifs is less harmful than not being able to take into account all motif positions in case of long motifs. We restart the expectation maximization algorithm, which is deterministic for a given dataset and a given initialization, 100 times with different initializations and choose the foreground model and the background model with the maximum likelihood on the positive training data and the negative training data, respectively, for classification. We use a likelihood-ratio classifier of the two chosen foreground and background models, apply this classifier to the disjoint positive and negative test sets, and calculate the area under the receiver operating characteristics curve and the area under the precision recall curve as measures of classification performance.

We repeat both steps 25 times and determine (i) the mean area under the receiver operating characteristic curve and its standard error and (ii) the mean area under the precision recall curve and its standard error.

#### Relative increase of classification performance

We compute the relative increase or decrease of the classification performance of the PFM(1) and the PFM(2) relative to the PFM(0), where PFM(*C*) denotes a PFMs taking into account base dependencies of order *C*. We compute *R*_*PFM*(*C*)_ as the ratio of the improvement of the PFM(*C*) relative to the PFM(0) divided by the maximum possible improvement to the PFM(0) as given by

$$\begin{array}{*{20}l} R_{PFM(C)} = \frac{AUC_{PFM(C)} - {AUC}_{PFM(0)}} {1 - {AUC}_{PFM(0)}}. \end{array} $$

Negative values of *R*_*PFM*(*C*)_ denote a decrease of classification performance and positive values of *R*_*PFM*(*C*)_ denote an increase of classification performance up to a maximum of *R*_*PFM*(*C*)_=1 which denotes perfect classification (provided that the AUC of PFM(0) is smaller than 1).

### Mutual information

The mutual information (MI) is a standard measure for quantifying statistical dependencies. We compute the MI between a base at position *w* in a motif and its *C* preceding bases for *w*>*C* as follows

$$\begin{array}{*{20}l} I_{C}(w) = I\left(X_{w}, X_{w}^{C}\right) &= \sum_{a \in {\mathcal{A}}^{C}} \sum_{b \in {\mathcal{A}}} p\left(X_{w}^{C} = a, X_{w}=b\right)\\ &\quad\times\log_{2} \frac{p\left(X_{w}^{C} = a, X_{w}=b\right)}{p\left(X_{w}^{C} = a\right) p(X_{w}=b)} \end{array} $$

where *X*_*w*_ denotes the base at position *w* and $X_{w}^{C} =(X_{w-C},\ldots,X_{w-1})$ denotes the context of *X*_*w*_. *I*_*C*_(*w*) denotes the amount of information in the *C*-mer ending at position *w*−1 about its adjacent base at position *w*. *I*_*C*_(*w*) is undefined for *w*≤*C*.

We denote the vector of MIs values *I*_*C*_(*w*) for *w*∈{*C*+1,…,*W*} by *I*_*C*_=(*I*_*C*_(*C*+1),…,*I*_*C*_(*W*)), where *W* is the length of the motif, and we call this vector MI profile.

### Implementation

We implement the proposed PFM based on the freely available Java Framework *Jstacs* [42]. Among others, *Jstacs* provides ready-to-use sequence models for reuse, numerical and non-numerical optimization procedures for model estimation, serialization of models, and methods for the statistical evaluation of results. In contrast to existing tools which are typically focused on application, using *Jstacs* we are able to compare different PFMs in a detailed way by extracting mandatory information about the inferred models and the predicted binding sites.

Algorithm 1 shows the pseudocode for inferring a PFM from a set of alignments. The implementation of the proposed phylogenetic footprinting model is available at https://github.com/mgledi/PhyFoo/.



## Results and discussion

We propose a model for phylogenetic footprinting that is capable of taking into account intra-motif dependencies as specified in Methods 2. Specifically, we model intra-motif dependencies in TFBSs as well as dependencies among adjacent bases in flanking sequences by Markov models of orders 0,1, and 2, and we denote the proposed PFM by PFM(0), PFM(1), and PFM(2).

In the first subsection we study if the proposed PFMs can capture intra-motif dependencies of orders 1 and 2 in ChIP-Seq data of 35 TFs. In the second subsection we study if modeling base dependencies can improve phylogenetic footprinting. Both studies are based on human sequences extracted from ENCODE ChIP-seq data [33] and corresponding orthologous sequences of 9 mammalian species, yielding 35 data sets comprising 135196 multiple sequence alignments with an average length of 124 bases (Methods 1).

### Intra-motif dependencies can be captured by phylogenetic footprinting

In this subsection we study to which degree intra-motif dependencies can be captured using the PFMs of orders 1 and 2.

We measure the degree of intra-motif dependencies of order 1 between two neighboring bases or of order 2 between a dimer and its neighboring base by the MI as described in Methods 4. The MI quantifies the amount of information in a base or a dimer about the neighboring base in units of bits and ranges from 0 bits in case of statistical independence to 2 bits in case of deterministic dependency of the considered base on the preceding base or the preceding dimer. We compute the MI for every position of a binding site and call the resulting vector of MI values MI profile.

**Fig. 1:**
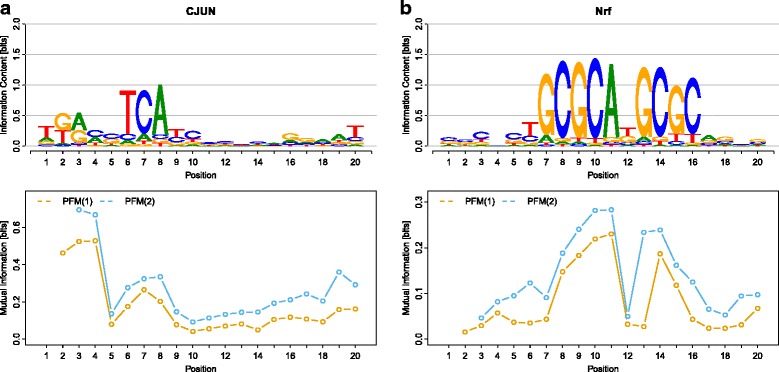
Sequence logos and intra-motif dependencies for the TFs **a** *CJUN* and **b** *Nrf*. We depict for both TFs (i) the sequence logo inferred by the PFM(2) from all species in the first row and (ii) the MI profiles of orders 1 and 2 inferred by the PFM(2) in the second row. The MI profiles of order 2 are larger than the MI profiles of order 1. Please see Additional file 3 for the MI profiles of all 35 TFs and Additional file 5 for all sequence logos of all 35 TFs for the PFMs of orders 0, 1, and 2

For each of the 35 TFs, we compute the two MI profiles of orders 1 and 2 from the motifs obtained by phylogenetic footprinting using the PFM(2). We present the resulting 35×2 MI profiles as Additional file 3 and the 2×2 MI profiles of the two TFs *CJUN* and *Nrf* as examples in Fig. 1 a.

First, we study the MI profiles of order 1 for these two TFs. For both TFs we find statistically significant intra-motif dependencies between neighboring bases at all positions. For *CJUN*, intra-motif dependencies of order 1 are particularly strong at motif positions 2 to 4, yielding a maximum MI of 0.52 bits at motif position 4. For *Nrf*, intra-motif dependencies of order 1 are particularly strong at motif positions 8 to 11 and 14 to 15, yielding a maximum MI of 0.23 bits at motif position 11.

Next, we study the MI profiles of order 2. Again, we find statistically significant intra-motif dependencies between dimers and their neighboring bases at all positions for both *CJUN* and *Nrf*. For *CJUN*, intra-motif dependencies of order 2 are particularly strong at motif positions 2 to 4, yielding a maximum MI of 0.70 bits at motif position 3. For *Nrf*, intra-motif dependencies of order 2 are particularly strong at motif positions 8 to 11 and 13 to 15, yielding a maximum MI of 0.28 bit at motif position 11.

Moreover, we find that intra-motif dependencies of order 2 are significantly stronger than the corresponding intra-motif dependencies of order 1 at several positions for both *CJUN* and *Nrf*. Comparing the MI profiles of orders 1 and 2, we find that the MI profile of order 2 is up to twofold higher than the MI profile of order 1 for *CJUN* and up to sevenfold higher for *Nrf*, stating that in both TFs there are significant intra-motif dependencies of order 2 beyond those expected from the corresponding intra-motif dependencies of order 1.

**Fig. 2:**
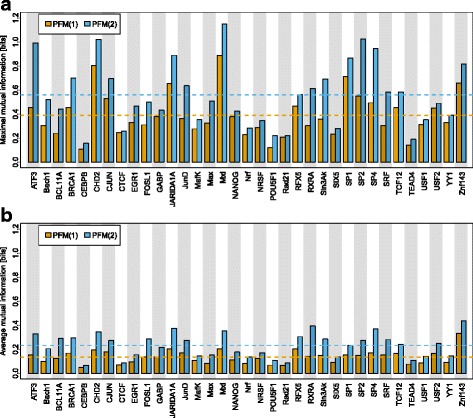
Maximum and average MIs of MI profiles inferred by the PFM(2) for all 35 TFs. In Fig. **a** we show the maximum MI of the MI profiles of orders 1 and 2. In Fig. **b** we show the average MI of the MI profiles of orders 1 and 2. The dashed lines indicate the mean of the maximum MIs and the mean of the average MIs for both MI profiles respectively. The degree of intra–motif dependencies depends of the TF and is always larger in case of intra–motif dependencies of order 2. Please see Additional file 3 for the MI profiles of all 35 TFs

Next, we study the MI profiles of orders 1 and 2 for all 35 TFs. In order to condense the results and to allow a visual comparison of the results for both profiles and all 35 TFs, we show for each MI profile and each TF the maximum and mean MI values in Fig. 2 a.

We find that the average of the 35 maximum MI values of order 1 is 0.39 bits, whereas the average of the 35 maximum MI values of order 2 is significantly greater at 0.56 bits. Likewise, we find that the average of the 35 mean MI values of order 1 is 0.14 bits, whereas the average of the 35 mean MI values of order 2 is significantly greater at 0.23 bits. These observations suggest that intra-motif dependencies are present in all of the studied TFs and that intra-motif dependencies of order 2 are typically stronger than those of order 1.

By scrutinizing Figs. 2 a and b, however, we also find that the maximum and mean MIs values vary significantly from TF to TF. For example, we find a maximum and mean MI value of order 1 of 0.11 bits and 0.05 bits for *CEBPB* and a maximum and mean MI value of order 1 of 0.89 bits and 0.20 bits for *Mxi*. Analogously, we find a maximum and mean MI value of order 2 of 0.16 bits and 0.07 bits for *CEBPB* and a maximum and mean MI value of order 2 of 1.15 bits and 0.37 bits for *Mxi*.

To study the possibility that these captured intra-motif dependencies are an artifact resulting from a mixture of different species-specific motifs, we finally study the similarity of the 10 species-specific motifs as well as the 20 species-specific MI profiles of orders 1 and 2. We find that the observed pairwise differences between the species-specific motifs are not significant (Additional file 1: Section 1.1.1). Moreover, we find that the species-specific MI profiles are similar to each other and to the corresponding MI profiles captured by phylogenetic footprinting (Additional file 4, Additional file 1: Section 1.1.2). Both findings indicate that the intra-motif dependencies shown in Fig. 1 b and in Additional file 3 cannot be explained as an artifact resulting from a mixture of different species-specific motifs.

### Modeling intra-motif dependencies improves phylogenetic footprinting

In this subsection we study if modeling base dependencies can improve phylogenetic footprinting.

First, we compute the classification performance of the PFMs of orders 0,1, and 2 as described in Methods 3. Second, we determine the increase of the classification performance of the PFMs taking into account base dependencies of orders 1 and 2 relative to the classification performance of the PFM neglecting base dependencies as described in Methods 3. Here, positive values indicate an increase of classification performance, while negative values indicate a decrease of classification performance.

**Fig. 3:**
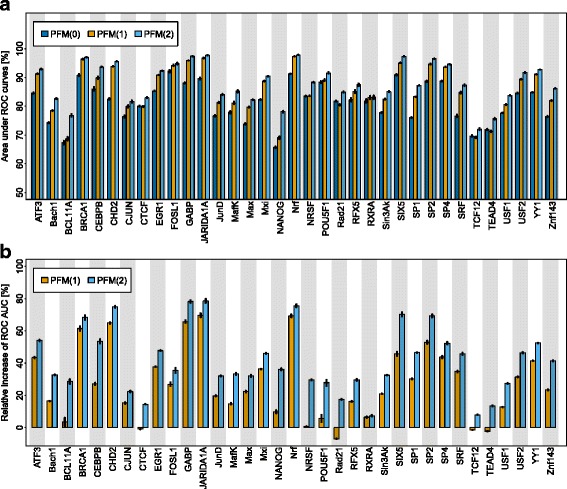
Classification performance for PFMs with base dependencies of orders 0,1 and 2. **a** We show the mean and standard error of the ROC AUC for PFMs of orders 0, 1, and 2 averaged over 25–fold stratified repeated random subsampling. **b** We plot the mean and standard error of the relative increase of the ROC AUC for the PFMs of orders 1 and 2 relative to the PFM or order 0 for each of the 35 TFs. Taking into account base dependencies of order 1 increases the classification performance for 31 TFs. Taking into account base dependencies of order 2 increases the classification performance in all cases and is larger compared to taking into account base dependencies of order 1 in all cases. See Additional file 6 for detailed ROC and PR curves for the PFMs of order 2

Figure 3 a shows the classification performances of the PFMs of orders 0,1, and 2 for each of the 35 TFs, and Fig. 3 b shows the corresponding relative increases. We find that modeling base dependencies of order 1 increases the classification performance in 31 of 35 cases, and we find that modeling base dependencies of order 2 increases the classification performance in all of the 35 cases. Moreover, we find that modeling base dependencies of order 2 always yields a higher classification performance than modeling base dependencies of order 1.

By scrutinizing Fig. 3 a, we find that the differences of the classification performances of the PFMs of orders 1 and 2 and the PFMs of order 0 vary significantly from TF to TF. For example, in case of base dependencies of order 1 we find the highest difference of 11*%* for CHD2 and the lowest difference of −1*%* for Rad21. In case of base dependencies of order 2 we find the highest difference of 13*%* for Rad21 and the lowest difference of 1*%* for RXRA.

By scrutinizing Fig. 3 b, we find that also the relative increases of classification performances vary significantly from TF to TF. For example, in case of base dependencies of order 1 we find the highest increase of 70*%* for JARIDA1A and the lowest increase of −7*%* for Rad21. In case of base dependencies of order 2 we find the highest increase of 78*%* for JARIDA1A and the lowest increase of 7*%* for RXRA.

**Fig. 4:**
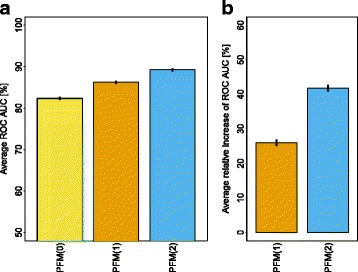
Classification performance averaged for all 35 TFs. **a** We show the ROC AUC for PFMs of orders 0, 1, and 2 in percent averaged over 25–fold stratified repeated random subsampling and averaged over all 35 TFs. The overall classification performance increases with the order of the PFM. **b** We show the improvement of the ROC AUC for the PFMs of orders 1 and 2 relative to the PFM of order 0 averaged over 25–fold stratified repeated random subsampling and averaged over all 35 TFs

Figure 4 summarizes the results by showing (a) the classification performance of the PFMs of orders 0,1, and 2 averaged over all 35 TFs and (b) the relative increases of classification performances averaged over all 35 TFs. We observe that the average classification performance increases significantly from order 0 to order 1 and from order 1 to order 2. Specifically, we find that the average classification performance of the PFM(1) is 4.6*%* higher than that of the PFM(0) and that the average classification performance of the PFM(2) is 3.5*%* higher than that of the PFM(1). We find that the average relative increase of the classification performance of the PFM(1) over that of the PFM(0) is 25*%* and that the average relative increase of the classification performance of the PFM(2) over that of the PFM(0) is 42*%*.

Next, we study the robustness of the proposed approach with respect to the number of species in the multiple sequence alignments. We perform the same study on the same 35 datasets with alignments comprising only subsets of the 10 species, and we find that for all subsets the classification performance increases significantly from order 0 to order 1 for many of the 35 TFs and from order 1 to order 2 for all of the 35 TFs (Additional file 1: Section 1.2).

These findings indicate that taking into account base dependencies improves phylogenetic footprinting, but they also indicate that this improvement is small. Given the fact that taking into account base dependencies improves one-species approaches, too, it could well be that the improvement obtained by taking into account base dependencies in one-species approaches is greater than in phylogenetic footprinting. Such a difference could result in the situation where the advantage of phylogenetic footprinting over one-species approaches when neglecting base dependencies decreases or even turns into a disadvantage when taking into account base dependencies.

To study to which degree the small improvement of phylogenetic footprinting by taking into account base dependencies might be overshadowed by a possibly greater improvement of one-species approaches, we compare the classification performances of the four cases of one-species approaches and phylogenetic footprinting when neglecting and taking into account base dependencies (Additional file 1: Section 1.3). Consistent to previous studies, we find that phylogenetic footprinting yields a higher (lower) classification performance compared to one-species approaches for 23 (12) of the 35 TFs when neglecting base dependencies. When taking into account base dependencies, however, phylogenetic footprinting yields a higher (lower) classification performance compared to one-species approaches in 31 (4) of the 35 TFs.

This finding indicates that the small improvement of phylogenetic footprinting by taking into account base dependencies is greater than the corresponding improvement of one-species approaches. It also indicates that the previously observed advantage of phylogenetic footprinting over one-species approaches when neglecting base dependencies (23 to 12) does not decrease or turn into a disadvantage, but becomes even more pronounced (31 to 4), when taking into account base dependencies. This increased advantage of phylogenetic footprinting over one-species approaches achieved by taking into account base dependencies is surprising as it indicates the presence of some synergy of modeling both phylogenetic and base dependencies.

We finally study for each of the 35 TFs which of the four models yields the highest classification performance, and we find that one-species approaches neglecting base dependencies yields the highest classification performance for one TF (*CEBPB*), one-species approaches taking into account base dependencies yields the highest classification performance for three TFs (*BCL11A*, *MafK*, and *RXRA*), phylogenetic footprinting neglecting base dependencies never yields the highest classification performance, and phylogenetic footprinting taking into account base dependencies yields the highest classification performance for 31 TFs. This finding indicates that phylogenetic footprinting can be improved by taking into account base dependencies, that one-species approaches using base dependencies can be improved by taking into account phylogenetic dependencies, and that there is a surprising synergy of simultaneously modeling both phylogenetic and base dependencies.

## Conclusions

In this work, we introduced a phylogenetic footprinting model capable of taking into account base dependencies and evaluated this phylogenetic footprinting model on ChIP-seq data of 35 TFs. We found significant intra-motif dependencies of orders 1 and 2 in all 35 datasets and that the inferred intra-motif dependencies of order 2 are stronger than those of order 1 for all 35 TFs. We also found that these intra-motif dependencies cannot be explained as an artifact resulting from a mixture of different species-specific motifs. We further found that the classification performance of the introduced phylogenetic footprinting model is higher than that of phylogenetic footprinting models neglecting base dependencies for all of the 35 TFs and higher than that of one-species approaches for 31 of the 35 TFs. These findings suggest that combining phylogenetic footprinting with motif models incorporating intra-motif dependencies may lead to an improved prediction of TFBSs and thus advance our understanding of transcriptional gene regulation and its evolution.

## Additional files

Additional file 1 Supplementary Material. This file is structured in three sections, presenting four additional studies, details about the implementation and some statistics regarding the datasets of all 35 TFs.
In Section 1, *Supplementary Results*, we first study differences among species–specific motifs of 35 TFs. We then study the robustness of the proposed PFM to different species compositions on data of 35 TFs. Third, we examine the impact of base dependencies and phylogenetic dependencies on classification performance. In the fourth subsection, we compare the proposed PFM(2) with a state of the art tool by Eggeling et al. 2015 [13] on data of 35 TFs. In the fifth subsection, we show statistics of the distances between ChIP-seq positive regions and the alignment coverage of ten species. Finally, we specify the run–time of our freely available implementation of the proposed PFM.
In Section 2, *Supplementary Methods*, we specify details about the estimation of species–specific motifs and we define a statistical test for the significance of differences among species–specific motifs.
In Section 3, *Supplementary Tables*, we show statistics of the datasets of 35 TFs, summarize results regarding the significance of species–specific motifs and the impact of base dependencies and phylogenetic dependencies, and show the alignment coverage of ten species for 35 TFs. (PDF 1034.24 kb)

Additional file 2 Sequence data. This archive contains data files of alignments of the ChIP-seq positive regions and negative control regions for each of the 35 TFs in FASTA format. (ZIP 83763.2 kb)

Additional file 3 Sequence logos, MI profiles of order 1, MI profiles of order 2, and species-specific MI profiles of orders 1 and 2. The file contains for each of the 35 TFs the sequence logo inferred using the PFM(2) aligned with MI profiles of order 1, the MI profiles of order 2, and species-specific MI profiles of orders 1 and 2 for each of the 10 species. (PDF 2129.92 kb)

Additional file 4 Tables of difference logos. The file contains for each of the 35 TFs a 10×10 table of difference logos for a pair-wise visual comparison of species-specific motifs. (ZIP 26112 kb)

Additional file 5 Sequence logos of predicted binding sites. The file contains sequence logos and their reverse complements of predicted binding sites inferred using the PFM(0), the PFM(1), and the PFM(2) for each of the 35 TFs. (PDF 11776 kb)

Additional file 6 ROC curves. The pdf file comprises for each TF one plot that shows the 25 ROC curves and one plot that shows the 25 PR curves from the 25–fold stratified repeated random sub-sampling validation procedure described in Methods 3. (PDF 2611.2 kb)

## Abbreviations

MI: mutual information
PFM: phylogenetic footprinting model
PWM: position weight matrix
TF: transcription factor
TFBS: transcription factor binding site
