# Supplementary material for: Combining phylogenetic footprinting with motif models incorporating intra-motif dependencies
Source: BMC Bioinformatics. 2017 Mar 1;18:141. doi: 10.1186/s12859-017-1495-1 (PMC5333389; doi:10.1186/s12859-017-1495-1)
Supplement: Supplementary file 1 — Supplementary Material. This file is structured in three sections, presenting four additional studies, details about the implementation and some statistics regarding the datasets of all 35 TFs. In Section 1, Supplementary Results, we first study differences among species–specific motifs of 35 TFs. We then study the robustness of the proposed PFM to different species compositions on data of 35 TFs. Third, we examine the impact of base dependencies and phylogenetic dependencies on classification performance. In the fourth subsection, we compare the proposed PFM(2) with a state of the art tool by Eggeling et al. 2015 [13] on data of 35 TFs. In the fifth subsection, we show statistics of the distances between ChIP-seq positive regions and the alignment coverage of ten species. Finally, we specify the run–time of our freely available implementation of the proposed PFM. In Section 2, Supplementary Methods, we specify details about the estimation of species–specific motifs and we define a statistical test for the significance of differences among species–specific motifs. In Section 3, Supplementary Tables, we show statistics of the datasets of 35 TFs, summarize results regarding the significance of species–specific motifs and the impact of base dependencies and phylogenetic dependencies, and show the alignment coverage of ten species for 35 TFs. (PDF 1034.24 kb) [file 12859_2017_1495_MOESM1_ESM.pdf]

1 Supplementary results

In the following subsections we describe four additional studies to substantiate the findings presented in the manuscript and provide some statistics regarding the used datasets and the run-time of our approach.

In the first **Results** subsection of the main manuscript we study to which degree intra-motif dependencies can be captured using the proposed phylogenetic footprinting models (PFMs). In **Supplementary Section 1.1** we study species–specific motifs to rule out the possibility that the estimated intra–motif dependencies presented in the manuscript result from a mixture of species–specific motifs. We find that species–specific motifs are highly similar for most transcription factors (TFs).

In the second **Results** subsection of the main manuscript and **Figures 3 and 4** we show that taking into account base dependencies improves phylogenetic footprinting. In **Supplementary Section 1.2** we study whether these results are robust to different species compositions. We find that the proposed PFM is robust with respect to different species compositions. Additionally, in **Supplementary Section 1.3** we study to which degree intra–motif dependencies and phylogenetic dependencies contribute to the improved motif prediction performance. We confirm previous findings that modeling base dependencies as well as phylogenetic dependencies improves classification performance. We find that modeling base dependencies improves classification performance to a higher degree than modeling phylogenetic dependencies and we find that modeling both typically results in the best classification performance. In **Section 1.4** we compare the classification performance of the proposed PFM to the classification performance of a state–of–the–art method by Eggeling *et al.* [1].

1.1 Species–specific motifs are highly similar for most TFs

Intra-motif dependencies may be a constant phenomenon conserved across the examined species or a rather dynamic phenomenon significantly changing during the evolution of these species. The latter case may imply that species–specific motifs are different to a certain degree. Consequently, the estimation of base dependencies across species may result in the estimation of spurious results. Hence, **first** we visually study differences among the species–specific motifs for each of the 35 TFs using *difference logos*, **second** we determine whether observable differences between species–specific motifs are significant or not, and **third** we examine the distribution of position-specific mutual informations (MIs) for each species. Therefore, we extracted for each of the 35 TFs one motif for each of the 10 species resulting in 35 $\times$ 10 species–specific motifs as described in **Supplementary Section 2.1**. Please note that the extracted species–specific motifs for other species than the reference species are not representative for these phylogenetically related species.

1.1.1 Primates show almost no differences in their sequence logos

We use the freely available R package *DiffLogo* for the visual inspection of motif differences [3]. *DiffLogo* enables the illustration and investigation of differences between highly similar motifs such as binding motifs of TFs from different experiments, different motif prediction algorithms, or different species. Hence, we use tables of difference logos generated with *DiffLogo* for a pair-wise comparison of all species–specific motifs. Each difference logo displays position-specific differences

of base distributions by a stack of bases which height is proportional to the base distribution difference quantified by the Jensen-Shannon divergence. The Jensen-Shannon divergence is zero in case of two identical base distributions and 1 in case of two maximally different base distributions. The tables of difference logos for all 35 TFs can be found in Additional File 1. All sequence logos of PFM(0), PFM(1), and PFM(2) can be found in Additional File 3.

Exemplary, **Supplementary Figure S1** shows the table of difference logos for the TF *Bach1*. We find that the species–specific motifs segregate into two main groups, where one group comprises seven higher primates and the second group comprises three species from the Laurasiatheria superorder, i.e., dog, horse, and cow. We find differences between the motifs of both groups at various motif positions, where the motif differences of relatively high degree are located at rather conserved motif positions as well as at more variable motif positions. For instance, we find relatively high differences at motif position 8, where guanine is more abundant in the primate motifs and the remaining bases are more abundant in the Laurasiatheria motifs. However, the maximum Jensen-Shannon divergence in all difference logos for *Bach1* is below 0.01 bits.

We examine the motif differences between species–specific motifs for all 35 TFs. We see for 14 TFs that the set of ten species segregates into the two groups of seven higher primates and three from the Laurasiatheria clade as before in case of *Bach1* (CEBPB, CTCF, EGR1, MafK, Max, NRSF, POU5F1, Rad21, SRF, TCF12, TEAD4, USF1, USF2, YY1). We find for the remaining 21 TFs that the motifs of the seven higher primates are more similar to each other compared to the motifs of dog, horse, and cow. With other words, in these cases the pairwise difference logos of dog, horse, and cow do not form a second cluster. The differences observed among species–specific motifs could partly result from missing binding sites in some species. **Supplementary Table S6** shows for each species and for each TF the proportion of sequences which are available for the computation of species–specific motifs.

1.1.2 Species–specific motifs are typically highly similar

We study the statistical significance of differences between species–specific motifs as follows. We examine for each of the 35 TFs the similarity of species–specific motifs using a statistical test for each two species ($(10*9)/2 = 45$ species pairs). We calculate for each TF and for each two species the p-value for the null hypothesis that two species–specific motifs arise from the same distribution as described in **Supplementary Section 2.2** resulting in $35 * 45 = 1575$ pairwise comparisons. We count for each two species how often we reject the null hypothesis for a confidence level of $\alpha = 0.05$. These counts range from 0 to 35, where 0 means that the species-specific motifs of two species show no significant differences for each transcription factor and 35 means that the species-specific motifs of two species show significant differences for each transcription factor. The binary nature of the results of statistical tests can lead to the issue that comparisons between three species are not transitive, i.e., if there are no significant differences between the species–specific motifs of species A and B and there are no significant differences between the species–specific motifs of species B and C it can happen that there are significant differences between the species–specific motifs of species A and C.

Supplementary Table S3 shows the results for each pair of species. We find that the seven primate-specific motifs are highly similar to each other and that the three species-specific motifs of cow, dog, and horse show greater differences compared to those of the seven primates. Using a significance level of 95%, we expect 5% of all 1575 pairwise differences to be significant by chance.

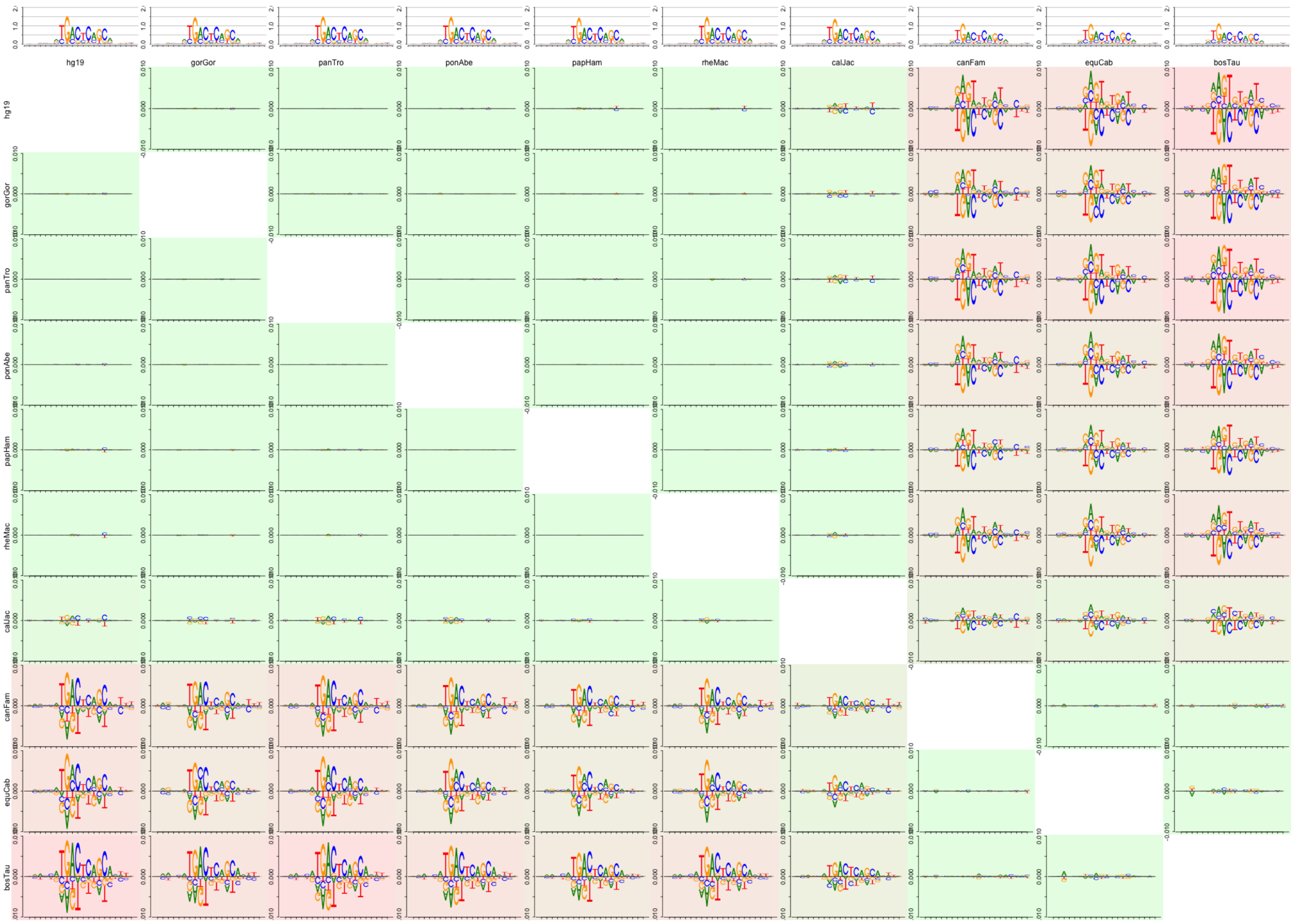

Figure S1: **Comparison of species-specific motifs for the TF *Bach1*.** We depict a table of difference logos with one row and one column for each species-specific motif to emphasize the differences between species-specific motifs. Each difference logos depicts the motif differences position-wise with a stack of bases, which height is calculated by the Jensen-Shannon divergence of the position-specific base distributions. The overall similarity between species-specific motifs is calculated by the sum of Jensen-Shannon divergences of all motif positions and depicted by the background color of the difference logos from green (similar) to red (dissimilar). The table of difference logos indicates, that the ten species-specific *Bach1* motifs primarily segregate into two clusters, where one cluster comprises seven primates and the other cluster comprises three non-primates (cow, dog, and horse).

We find for only 47 of 1575 pairwise comparisons that two species–specific motifs show significant differences. However, we find only 47 (3%) of the pairwise differences to be significant, stating that the observed differences are not greater than expected by chance.

Specifically, we find that these 47 cases apply only to 6 of the 35 TFs, namely Bach1, CEBPB, MafK, Max, SP1, and USF1 and typically only apply to comparisons between a primate species and one of the species dog, cow, and horse. We find no significant differences between the seven primates reflecting the close phylogenetic relationship and accordingly the high sequence similarity. Amongst the three species dog, cow, and horse we find for 1 of the 105 pairwise comparisons significant differences.

These results imply that the motifs estimated across species as presented in the previous section are typically not a mixture of species–specific motifs.

1.1.3 Intra–motif dependencies are highly similar for all species

We examine for each of the 35 TFs the distribution of species–specific MIs using MI profiles I_1^S and I_2^S as described in **Methods 4** for each species $S \in \{hg19,\ panTro,\ papHam,\ ponAbe,\ rheMac,\ calJac,\ equCab,\ canFam,\ gorGor,\ bosTau\}$. **Figure S2** shows two examples of species–specific MI profiles I_1^S and I_2^S for the two TFs *CJUN* and *Nrf*. All species–specific MI profiles are available in **Additional File 2**.

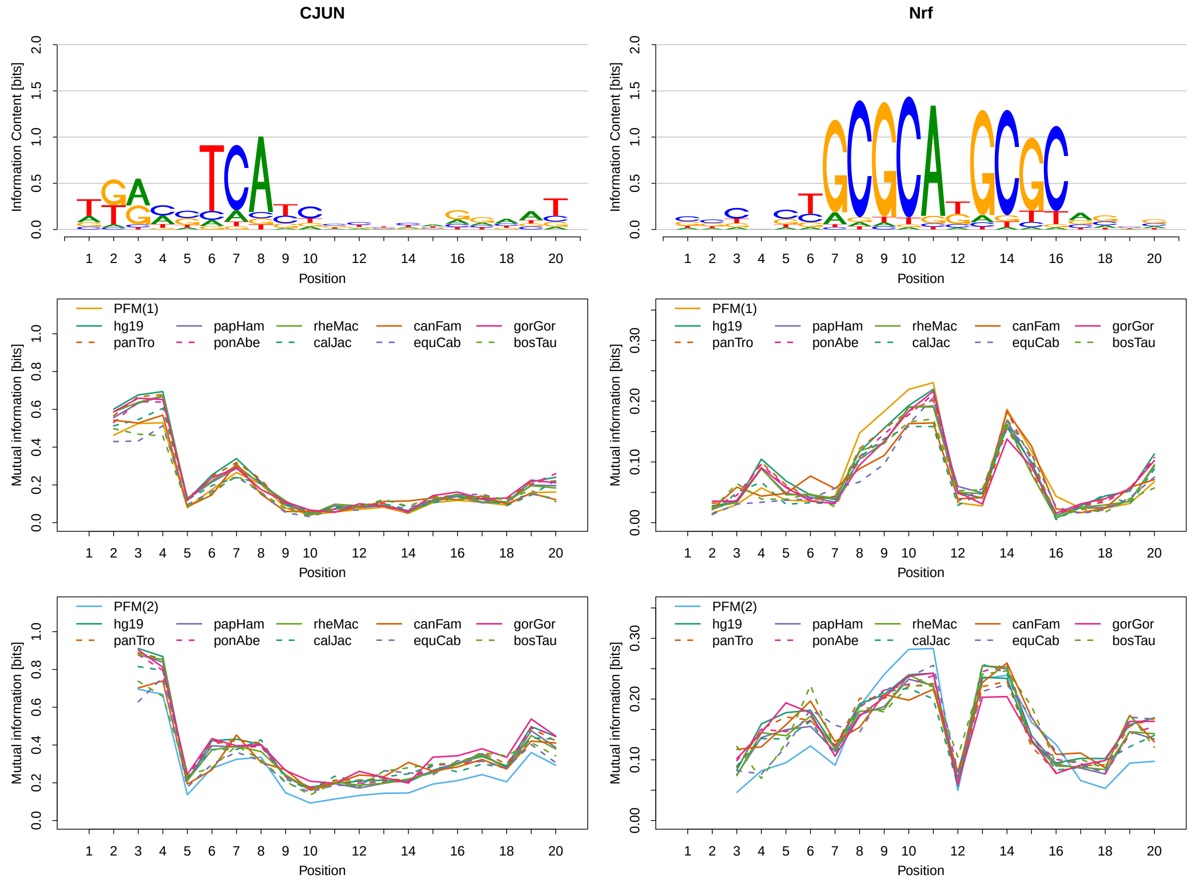

(a) **Sequence logo and MI for TF *CJUN***

(b) **Sequence logo and MI for TF *Nrf***

Figure S2: **Sequence logos and intra-motif dependencies for the TFs (left) *CJUN* and (right) *Nrf*.** We depict for both TFs (i) the sequence logo inferred by the PFM(2) from all species in the first row, (ii) the species-specific MI profiles inferred from the PFM(1) in the second row, and (iii) the species-specific MI profiles inferred from the PFM(2) in the third row. The species-specific MI profiles inferred from both models are highly similar to each other.

First, we study the species–specific MI profiles I_1^S. We find for each of the 35 TFs that the species–specific MI profiles I_1^S are highly similar for all species. We also find that the MIs in the MI profiles I_1 are sometimes stronger, sometimes weaker, and often averaged compared to the species–specific MIs I_1^S implying that the MI profiles I_1 inferred from all species are partly a result of interference of species–specific MIs. For example, in case of TF *CJUN* at motif positions $w \in \{2, 3, 4\}$, the MIs $I_1(w)$ are smaller than the MIs $I_1^S(w)$ for all species except horse and marmoset and in case of TF *Nrf* at motif positions $w \in \{8, 9, 10, 11\}$ the MIs $I_1(w)$ are higher than the MIs $I_1^S(w)$ for all species. Specifically, we find the largest difference between two I_1^S for

FOSL1 with 0.35 bits. However, the MI profiles I_1 inferred from all species are typically highly similar to the species–specific MI profiles I_1^S.

Second, we examine species–specific MI profiles I_2^S. We find for each of the 35 TFs that the species–specific MI profiles I_2^S are highly similar for all species. We also find that the MIs in the MI profiles I_2 are sometimes stronger, sometimes weaker, and sometimes averaged compared to the species–specific MIs I_2^S implying that the MI profiles I_2 inferred from all species are partly a result of interference of species–specific MIs. For example, in case of *CJUN* the MI profile $I_2(w)$ is typically smaller than the MI profile $I_2^S(w)$ for all species at all motif positions w and in case of *Nrf* at motif positions $w \in \{8 - 11\}$ the MIs $I_2(w)$ are higher than the MIs $I_2^S(w)$. Specifically, we find the largest difference between two I_2^S for *FOSL1* with 0.48 bits. However, the MI profiles I_2 inferred from all species are typically highly similar to the species–specific MI profiles I_2^S as in case of I_1 and I_1^S.

1.2 PFMs are robust according to different species compositions

We compare the performance of the PFM(0), the PFM(1), and the PFM(2) described in **Methods 2**. We measure the classification performance as described in **Methods 3** on datasets of 35 TFs described in **Methods 1**. For the evaluation of the robustness of our model, we vary the number of orthologous species the PFMs rely on. Specifically, we use four species (human, horse, dog, and cow), five species (human, chimp, horse, dog, and cow), seven species (human, chimp, baboon, rhesus, horse, dog, and cow), and ten species (human, chimp, baboon, orangutan, rhesus, marmoset, gorilla, horse, dog, and cow). See **Supplementary Figures S3, S4, S5**, and **Figure 5** of the main manuscript for the results. We find for each species composition that the PFM(1) typically outperforms the PFM(0) and that the PFM(2) outperforms both the PFM(0) and the PFM(1) in all cases. This finding indicates that modeling higher order markov models within phylogenetic footprinting seem to be robust despite the species composition and that the PFM with higher markov orders yields a better fit to the data.

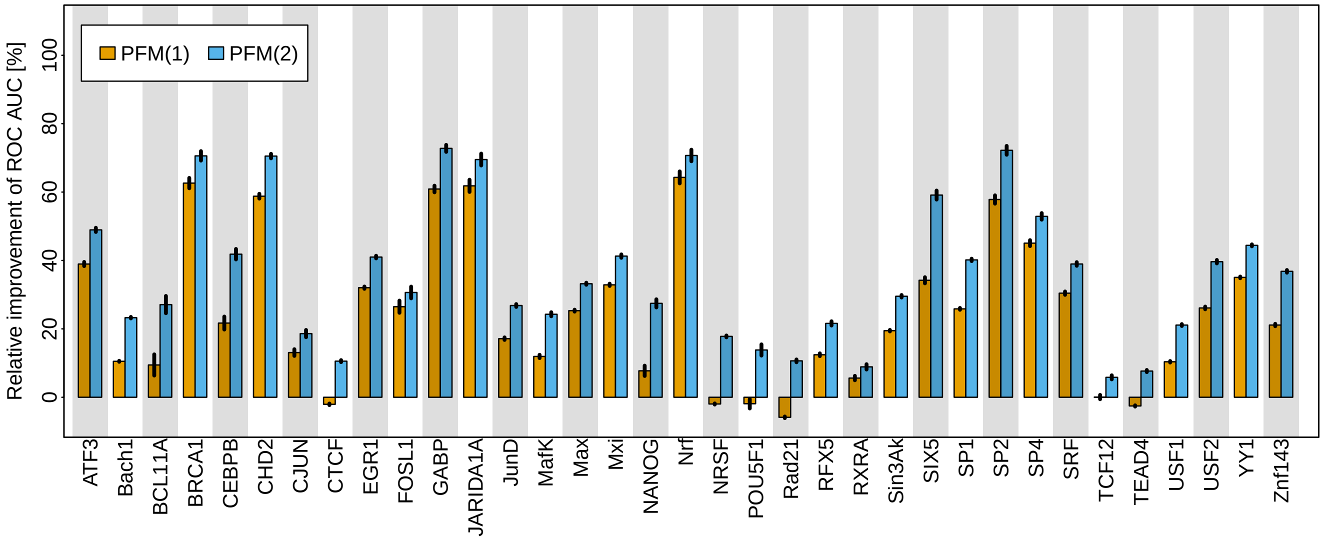

Figure S3: **Classification performance of PFM(1) and PFM(2) on four species**. We plot the mean and standard error of the relative improvement of the ROC AUC for the model PFM(1) and the model PFM(2) relative to the performance of the model PFM(0) for each of the 35 TFs on alignments of human, horse, dog, and cow. Modeling base dependencies of order 1 typically increases the classification performance and modeling base dependencies of order 2 increases the classification performance in all cases. The performance of the PFM(2) is larger than the performance of the PFM(1) in all cases.

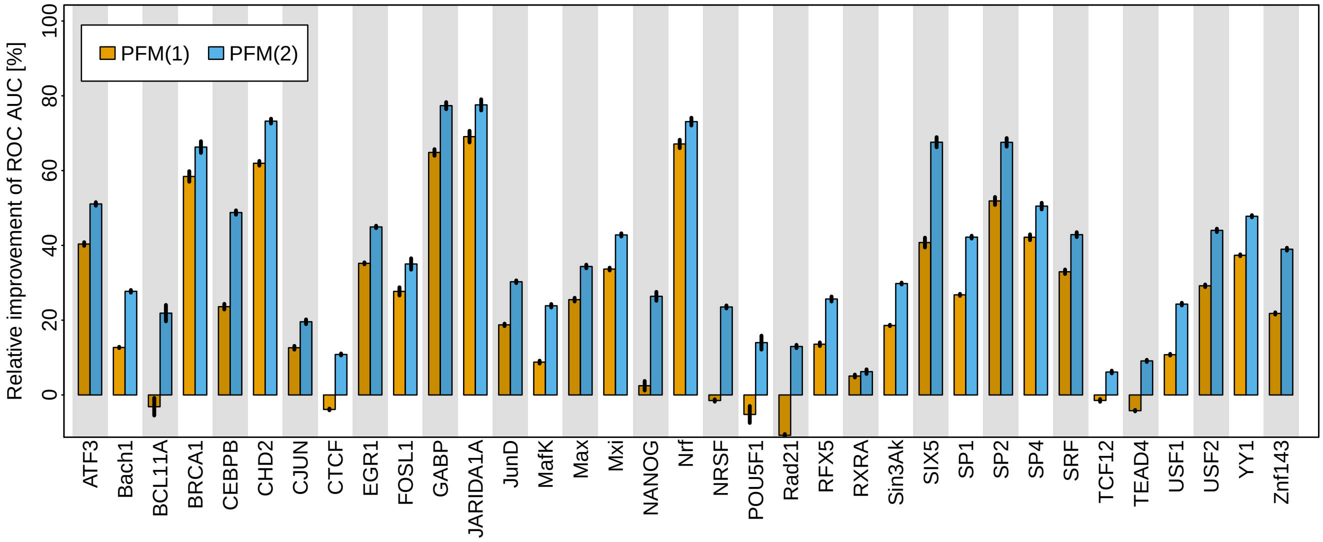

Figure S4: **Classification performance of PFM(1) and PFM(2) on four species**. We plot the mean and standard error of the relative improvement of the ROC AUC for the model PFM(1) and the model PFM(2) relative to the performance of the model PFM(0) for each of the 35 TFs on alignments of human, chimp, horse, dog, and cow. Modeling base dependencies of order 1 typically increases the classification performance and modeling base dependencies of order 2 increases the classification performance in all cases. The performance of the PFM(2) is larger than the performance of the PFM(1) in all cases.

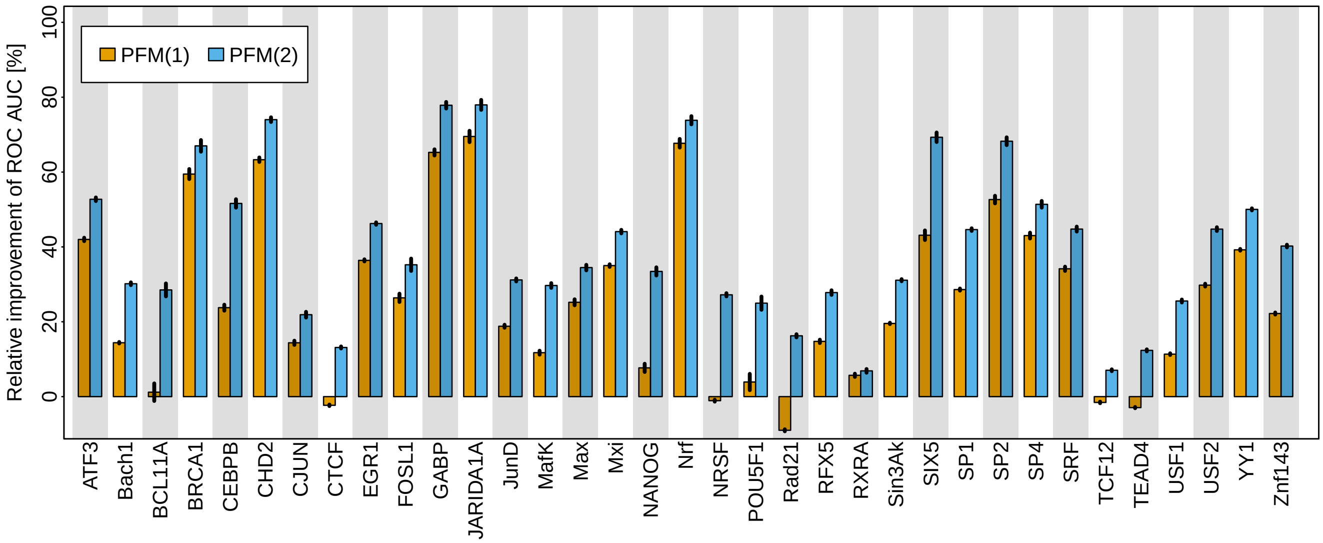

Figure S5: **Classification performance of PFM(1) and PFM(2) on four species**. We plot the mean and standard error of the relative improvement of the ROC AUC for the model PFM(1) and the model PFM(2) relative to the performance of the model PFM(0) for each of the 35 TFs on alignments of human, chimp, baboon, rhesus, horse, dog, and cow. Modeling base dependencies of order 1 typically increases the classification performance and modeling base dependencies of order 2 increases the classification performance in all cases. The performance of the PFM(2) is larger than the performance of the PFM(1) in all cases.

1.3 Taking into account phylogeny improves classification performance in almost all cases.

It has been shown that taking into account base dependencies improves one-species approaches neglecting phylogenetic dependencies and it has been shown that taking into account phylogenetic dependencies can improve one-species approaches neglecting base dependencies. In the manuscript we have shown that taking into account base dependencies improves phylogenetic footprinting. Unfortunately, it can not be concluded from these observations that a model taking into account base dependencies and phylogenetic dependencies outperforms a model taking into account base dependencies but neglecting phylogenetic dependencies, because phylogenetic dependencies may potentially impair the model taking into account base dependencies.

Here, we systematically study the impact of both higher order base dependencies and phylogenetic dependencies to classification performance. Therefore, we study the performances of four different models, namely **i)** a model taking into account neither base dependencies nor phylogenetic dependencies (**human(0)**), **ii)** a model taking into account base dependencies of order 2 and neglecting phylogenetic dependencies (**human(2)**), **iii)** a model neglecting base dependencies and taking into account phylogenetic dependencies **PFM(0)**, and **iv)** a model taking into account both base dependencies and phylogenetic dependencies (**PFM(2)**) as described in **Methods 2**. The models PFM(0) and PFM(2) take into account phylogenetic dependencies and are inferred from the alignments described in **Methods 1**. The models human(0) and human(2) do not take not into account phylogenetic dependencies and are inferred from the human sequences of the alignments described in **Methods 1**. The models human(0) and human(2) are special cases of PFM(0) and PFM(2) incorporating only one species.

Based on these four models we perform all pair–wise comparisons, namely a) human(0) against human(2), b) human(0) against PFM(0), c) PFM(0) against PFM(2), d) human(2) against PFM(2), e) human(0) against PFM(2), and f) human(2) against PFM(0).

For case a), it has been shown that human(2) typically outperforms human(0), i.e., that modeling base dependencies improves classification performance. For case b), it has also been shown that PFM(0) typically outperforms human(0), i.e., modeling phylogenetic dependencies improves classification performance. For case c), we have shown that PFM(2) outperforms PFM(0), i.e., that taking into account higher order base dependencies improves phylogenetic footprinting. For case e) we assume that PFM(2) outperforms human(0) considering the cases a) and b). The cases d) and f) are unknown so far.

We measure the classification performance of all four models as described in **Methods 3** on datasets of 35 TFs. **Figure S6** shows the corresponding values for the four models human(0), PFM(0),human(2), and PFM(2) for each of the 35 TFs. See **Supplementary Table S4** and **Supplementary Table S5** for statistics of the results shown in **Supplementary Figure S6**.

It is not surprising that the model taking into account both base dependencies and phylogenetic dependencies outperforms the model ignoring base dependencies and phylogenetic dependencies (case e). We find that modeling base dependencies typically improves classification performance (cases a and c). Interestingly, we find that modeling base dependencies clearly outperforms modeling phylogenetic dependencies (case f). In fact, solely taking into account phylogenetic dependencies shows only a partial improvement (case b), but taking into account both phylogenetic dependencies and base dependencies shows a clear performance improvement compared to solely taking into account base dependencies (case d). These results suggest that phylogenetic footprint-

ing approaches benefit from taking into account base dependencies and that approaches on single species already taking into account base dependencies benefit from taking into account phylogenetic dependencies.

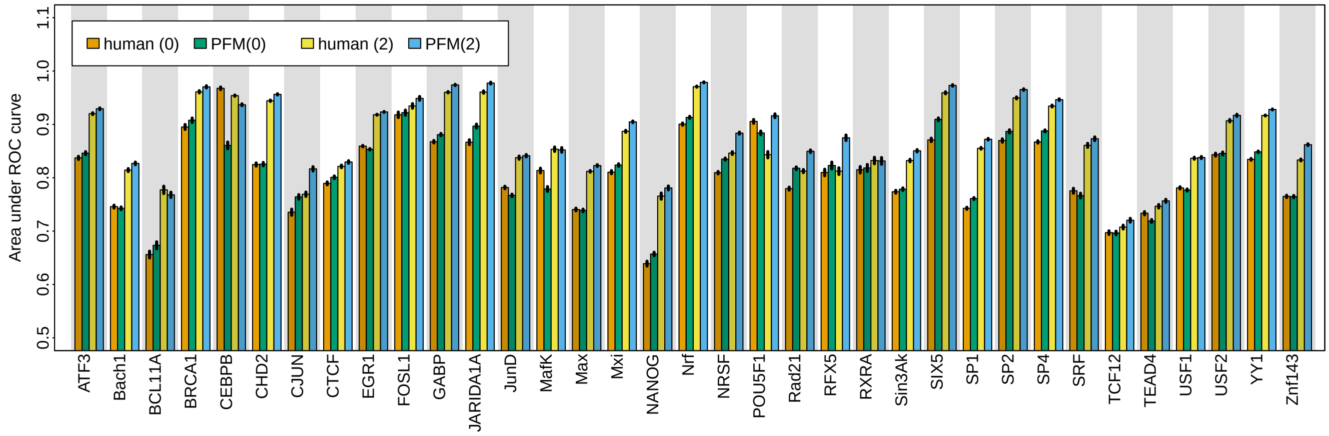

Figure S6: **Classification performance of the models human(0) and human(2) on human sequences and PFM(0) and PFM(2) on alignments of ten species for each of the** 35 **TFs.** We show the mean and standard error of the ROC AUC. See **Table S4** and **Table S5** for summary statistics.

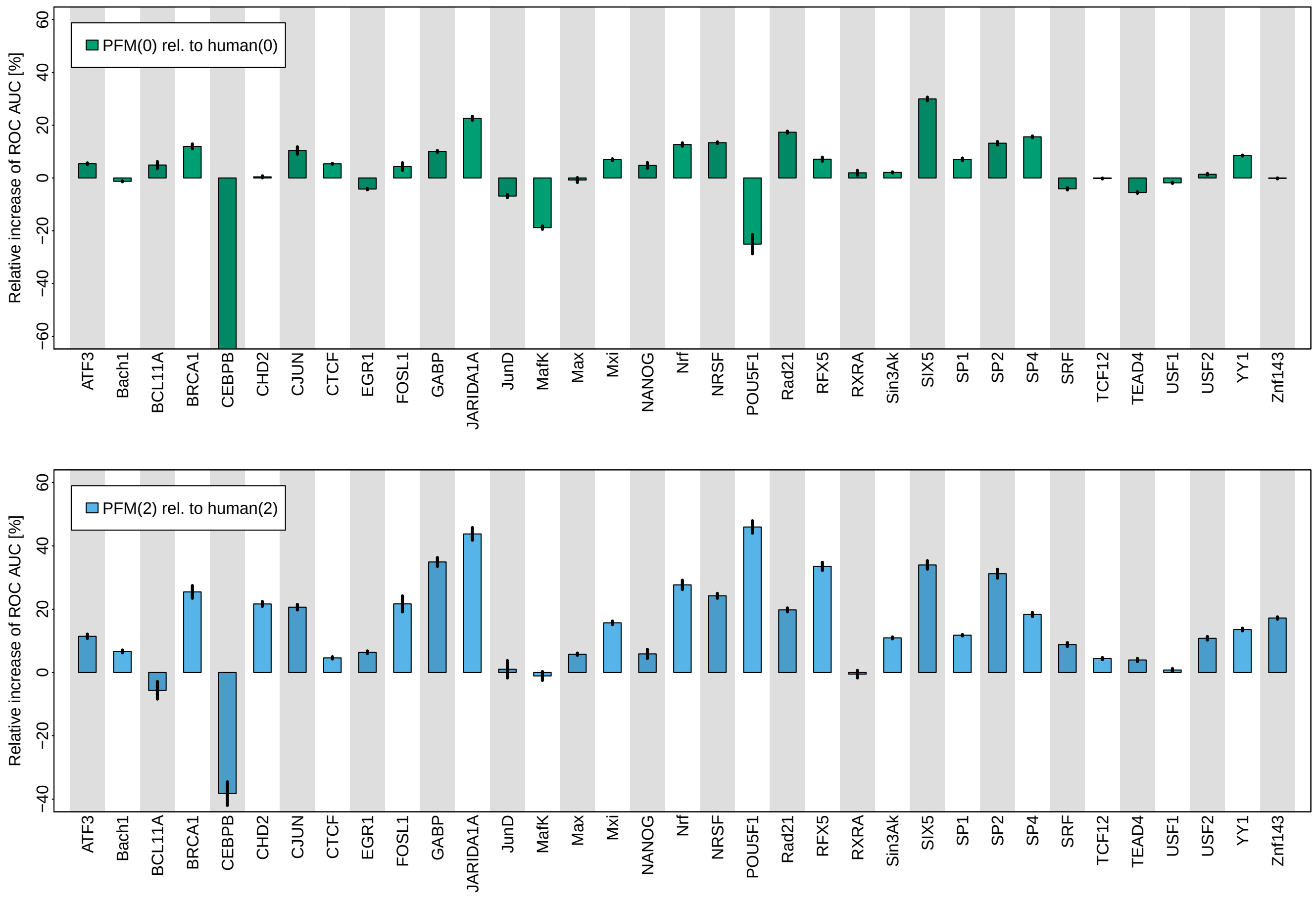

Figure S7: **Classification performance of the two models PFM(0) and PFM(2) incorporating phylogenetic dependencies for each of the 35 TFs.** We show the mean and standard error of the relative increase of ROC AUC of **(top)** PFM(0) relative to the classification performance of human(0) and **(bottom)** PFM(2) relative to the classification performance of human(2). Typically both models show a higher classification performance.

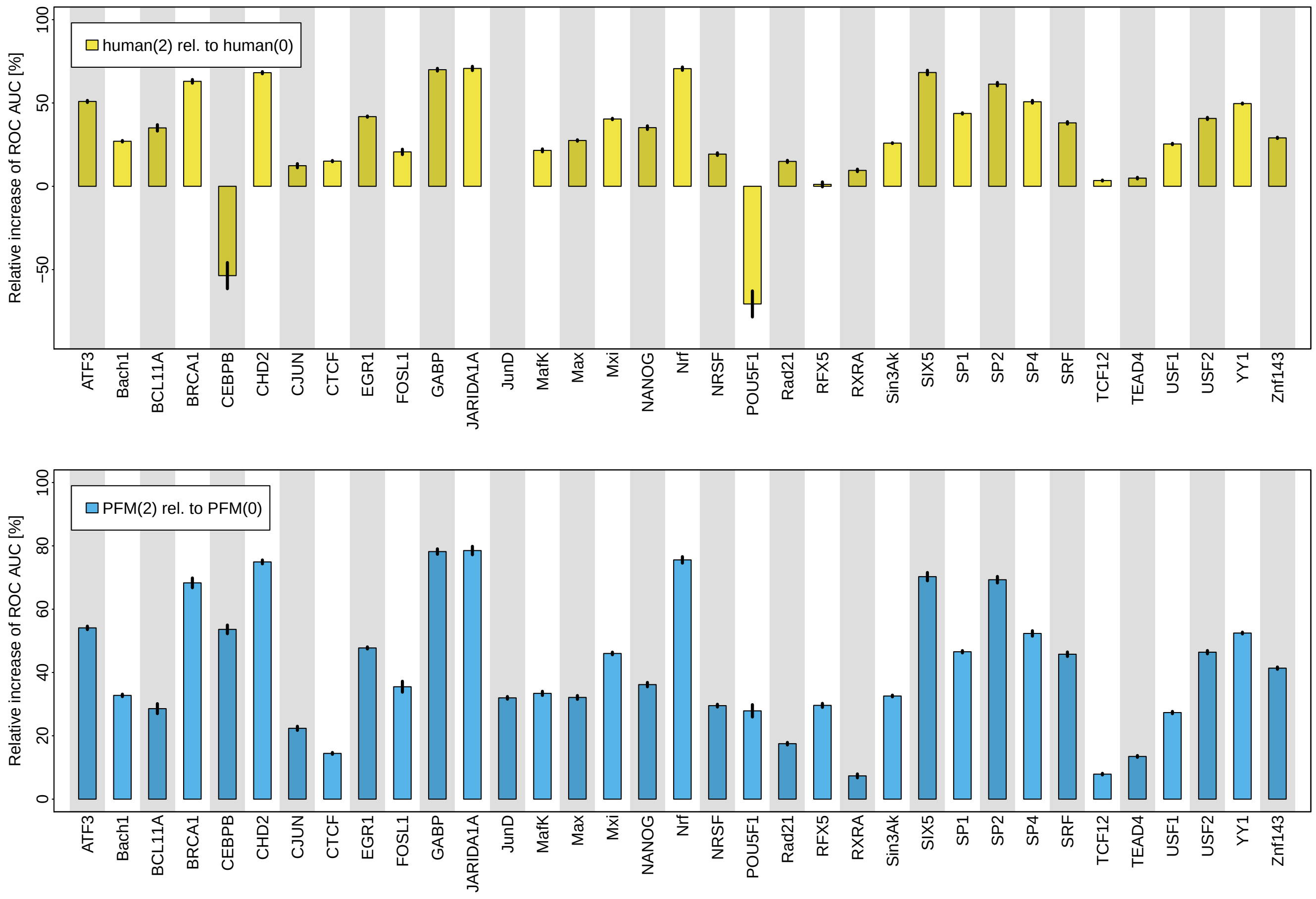

Figure S8: **Classification performance of the two models human(2) and PFM(2) incorporating base dependencies of order two for each of the 35 TFs.** We show the mean and standard error of the relative increase of ROC AUC of **(top)** human(2) relative to the classification performance of human(0) and **(bottom)** PFM(2) relative to the classification performance of PFM(0). Typically both models show a higher classification performance.

1.4 Comparison of a PMM(2) with a PFM(2) on 35 TFs

We compare the performance of the PFM of order 2 described in **Methods 2** and the parsimonious motif model (PMM) of order 2 proposed in Eggeling *et al* and denoted as PMM(2) [1]. We measure the classification performance as described in **Methods 3** on datasets of 35 TFs described in **Methods 1** (the PMM uses only human sequences). See **Supplementary Figure S9** for the results. We find that the mean performance of the PFM(2) is greater than the mean performance of the PMM(2) in 17 cases and we find that the mean performance of the PMM(2) is greater than the mean performance of the PFM(2) in 18 cases indicating that both parsimony and phylogeny can be beneficial depending on the TF of interest.

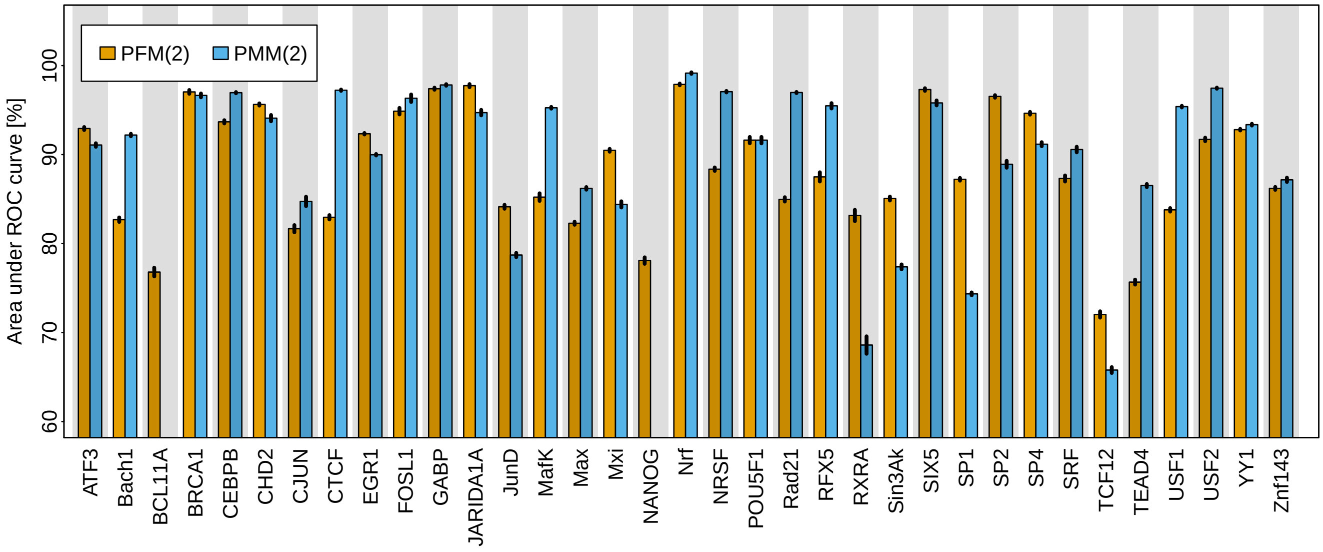

Figure S9: **Classification performance of the PMM(2) from Eggeling *et al* on human sequences and the proposed PFM(2) on alignments of 10 species for each of the 35 TFs.** We show the mean and standard error of the ROC AUC.

1.5 Data statistics

In the following we provide two statistics regarding the used datasets. In section 1.5.1 we study the distance between adjacent positive ChIP-Seq regions In section 1.5.2 we show the number of sequences used for the calculation of species-specific motifs.

1.5.1 Distances between adjacent ChIP-Seq positive regions

In the manuscript we describe in **Methods 1** how the positive and negative datasets we use in our studies were obtained. Here, we study the distances between two adjacent ChIP-Seq positive regions. The distance D between two adjacent ChIP-Seq positive regions A and B, where A is located before B, is defined by the difference between the last base of region A and the first base of region B. **Figure S10 shows the result.**

We find an median distance of 145414 bases and average distance of 971094 bases. We find that 99.7% of all distances are larger than 500, 94.2% are larger than 5000, and 90% are larger than 10000.

1.5.2 Sequence statistics for the calculation of species-specific motifs

Table S6 shows for each TF and for each species the number of binding site sequences that were incorporated into the calculation of the corresponding species–specific motif relative to the number of human binding site sequences.

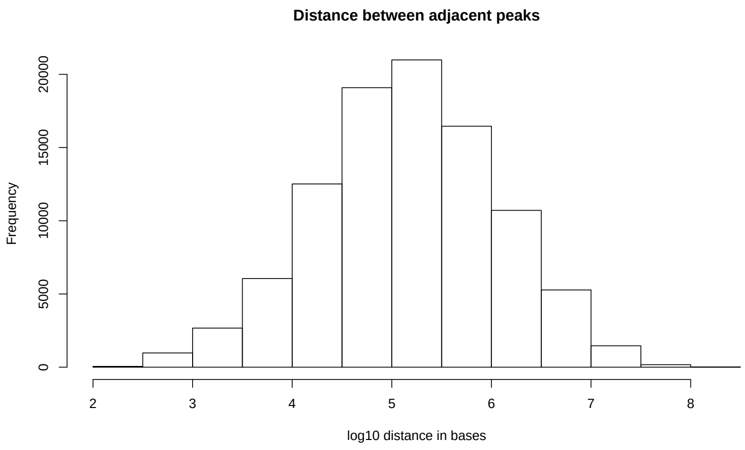

Figure S10: **Distances between adjacent ChIP-seq positive peaks aggregated for all** 35 **TFs.** We show a histogram of logarithmic distances ($\log_{10}$ in units of bp) between adjacent ChIP-seq positive peaks. 94.2% of the adjacent ChIP-seq positive peaks show distances of at least 5000 bp.

1.6 Run time

One EM run on 1000 alignments with an average length of 310 bases needs about five minutes to estimate a PFM of order 0 on a desktop computer. The run time increases linearly with the number of species, linearly with the number of alignments, and linearly with the length of the alignments. The run time increases exponentially (base four) with the order of the PFM. For example, 104 hours of CPU time are needed to perform a 25–fold stratified repeated random sub-sampling validation procedure for a PFM of order 0, 417 CPU hours for a PFM of order 1, and 1667 CPU hours for a PFM of order 2.

2 Supplementary Methods

2.1 Investigating species-specific motifs

We infer for each species and each TF an iMM(1) and iMM(2) as follows.

First, we train a PFM(2) on the complete positive training dataset by expectation maximization ([2]). We restart the expectation maximization algorithm 100 times with different initializations and choose the PFM(2) with the maximum likelihood.

Second, we use this model to calculate for each position ℓ_n in each alignment X_n the probability $p(X_n, M_n = 1, \ell_n)$ that a binding site occurs in alignment X_n at position ℓ_n. For each species o we store this probability and the corresponding sub–sequence of length W starting at position ℓ_n. We omit all sub–sequences containing gaps.

Third, for each species we calculate an iMM(1) and an iMM(2) from the corresponding set of species–specific sub–sequences and their probabilities $p(X_n, M_n = 1, \ell_n)$.

2.2 Statistical test for the similarity of species–specific motifs

Given two sets of weighted sub–sequences BS_1 and BS_2 we perform the following steps to calculate a p-value for the null hypothesis that the two sets BS_1 and BS_2 are generated one iMM(2). In this manuscript, each set corresponds to a set of probability–weighted sub–sequences obtained from the second step in the previous subsection.

First, we infer an iMM(2) on each set of weighted sub–sequences BS_1 and BS_2. We calculate the Jensen–Shannon divergence between both iMMs(2) denoted by $JS_{original}$.

Second, we build two new sets of weighted sub–sequences $\hat{BS}_1$ and $\hat{BS}_1$ by randomly assigning the sub–sequences from BS_1 and BS_2, such an median distance of $\hat{BS}_1$ contains the same number of sub–sequences as BS_1 and $\hat{BS}_2$ contains the same number of sub–sequences as BS_2. The assignment of the weights to the sub–sequences in preserved. We infer an iMM(2) on each set of weighted sub–sequences $\hat{BS}_1$ and $\hat{BS}_2$. We compute the Jensen–Shannon divergence between both iMMs(2) denoted by JS_{random}.

Third, we repeat the second step 1000 times and calculate the p-value as the number of cases where $JS_{random} < JS_{original}$ divided by 1000.

3 Supplementary Tables

Table S1: **Dataset statistics for human ChIP-Seq data and alignments.** For each of the 35 TFs we specify the (i) name of the TF, the (ii) number of ChIP-Seq positive regions, the (iii) number of alignments for ten species after quality control, and the (iv) average alignment length. Nearly all ChIP-Seq positive regions are covered by alignments of ten species.

TF name	# ChIP-Seq regions	# alignments	Avg. length
ATF3	961	960	192
Bach1	2291	2291	278
BCL11A	503	503	177
BRCA1	405	405	219
CEBPB	3111	3111	229
CHD2	1369	1369	237
CJUN	429	429	243
CTCF	10822	10819	161
Egr1	1748	1748	214
FOSL1	222	222	189
GABP	1130	1130	232
JARID1A	325	325	294
JunD	1689	1689	189
MafK	2285	2285	244
Max	2225	2225	268
Mxi	1270	1270	325
NANOG	1094	1094	175
Nrf	902	902	161
NRSF	2657	2657	285
POU5F1	799	799	206
Rad21	15136	15134	232
RFX5	339	339	304
RXRA	261	261	210
SIX5	685	685	191
Sin3Ak	1795	1795	261
SP1	3022	3022	219
SP2	493	493	277
SP4	1150	1150	506
SRF	1021	1021	169
TCF12	1566	1566	215
TEAD4	3971	3971	200
USF1	5208	5208	199
USF2	1390	1390	203
YY1	3665	3665	268
Znf1	6137	6135	264

Table S2: **Dataset details for human ChIP-Seq data.** For each of the 35 TFs we specify the (i) name of the TF, the (ii) laboratory code of the wet lab (HudsonAlpha for the HudsonAlpha Institute for Biotechnology, Stanford for the Stanford University, Broad for the Broad Institute of MIT and Harvard, and USC for the University of Southern California), the (iii) cell type, the (iv) treatment of the cell material, the (v) used antibody for immunoprecipitation, the (vi) version of the peak called dataset, and the (vii) ENCODE Data Coordination Center (DCC) accession number of the dataset.

TF name	Laboratory	Cell type	Treatment	Antibody	Data version	DCC accession
ATF3	HudsonAlpha	H1-hESC	None	ATF3	ENCODE Mar 2012 Freeze	wgEncodeEH001566
Bach1	Stanford	H1-hESC	None	Bach1_(sc-14700)	ENCODE Mar 2012 Freeze	wgEncodeEH002842
BCL11A	HudsonAlpha	H1-hESC	None	BCL11A	ENCODE Mar 2012 Freeze	wgEncodeEH001527
BRCA1	Stanford	H1-hESC	None	BRCA1_(A300-000A)	ENCODE Mar 2012 Freeze	wgEncodeEH002801
CEBPB	Stanford	H1-hESC	None	CEBPB	ENCODE Mar 2012 Freeze	wgEncodeEH002825
CHD2	Stanford	H1-hESC	None	CHD2_(AB68301)	ENCODE Mar 2012 Freeze	wgEncodeEH002827
CJUN	Stanford	H1-hESC	None	c-Jun	ENCODE Mar 2012 Freeze	wgEncodeEH001854
CTCF	HudsonAlpha	H1-hESC	None	CTCF_(SC-5916)	ENCODE Mar 2012 Freeze	wgEncodeEH001649
Egr1	HudsonAlpha	H1-hESC	None	Egr-1	ENCODE Mar 2012 Freeze	wgEncodeEH001538
FOSL1	HudsonAlpha	H1-hESC	None	FOSL1_(SC-183)	ENCODE Mar 2012 Freeze	wgEncodeEH001660
GABP	HudsonAlpha	H1-hESC	None	GABP	ENCODE Mar 2012 Freeze	wgEncodeEH001534
JARID1A	Broad	H1-hESC	None	JARID1A_(ab26049)	ENCODE Mar 2012 Freeze	wgEncodeEH002096
JunD	HudsonAlpha	H1-hESC	None	JunD	ENCODE Mar 2012 Freeze	wgEncodeEH001579
MafK	Stanford	H1-hESC	None	MafK_(ab50322)	ENCODE Mar 2012 Freeze	wgEncodeEH002828
Max	USC	H1-hESC	None	Max	ENCODE Mar 2012 Freeze	wgEncodeEH001757
Mxi	Stanford	H1-hESC	None	Mxi1_(AF4185)	ENCODE Mar 2012 Freeze	wgEncodeEH002829
NANOG	HudsonAlpha	H1-hESC	None	NANOG_(SC-33759)	ENCODE Mar 2012 Freeze	wgEncodeEH001635
Nrf	Stanford	H1-hESC	None	Nrf1	ENCODE Mar 2012 Freeze	wgEncodeEH001847
NRSF	HudsonAlpha	H1-hESC	None	NRSF	ENCODE Mar 2012 Freeze	wgEncodeEH001498
POU5F1	HudsonAlpha	H1-hESC	None	POU5F1_(SC-9081)	ENCODE Mar 2012 Freeze	wgEncodeEH001636
Rad21	HudsonAlpha	H1-hESC	None	Rad21	ENCODE Mar 2012 Freeze	wgEncodeEH001593
RFX5	Stanford	H1-hESC	None	RFX5_(200-401-194)	ENCODE Mar 2012 Freeze	wgEncodeEH001835
RXRA	HudsonAlpha	H1-hESC	None	RXRA	ENCODE Mar 2012 Freeze	wgEncodeEH001560
SIX5	HudsonAlpha	H1-hESC	None	SIX5	ENCODE Mar 2012 Freeze	wgEncodeEH001528
SP1	HudsonAlpha	H1-hESC	None	SP1	ENCODE Mar 2012 Freeze	wgEncodeEH001529
SP2	HudsonAlpha	H1-hESC	None	SP2_(SC-643)	ENCODE Mar 2012 Freeze	wgEncodeEH002302
SP4	HudsonAlpha	H1-hESC	None	SP4_(V-20)	ENCODE Mar 2012 Freeze	wgEncodeEH002317
SRF	HudsonAlpha	H1-hESC	None	SRF	ENCODE Mar 2012 Freeze	wgEncodeEH001533
TCF12	HudsonAlpha	H1-hESC	None	TCF12	ENCODE Mar 2012 Freeze	wgEncodeEH001531
TEAD4	HudsonAlpha	H1-hESC	None	TEAD4_(SC-101184)	ENCODE Mar 2012 Freeze	wgEncodeEH003214
Sin3Ak	HudsonAlpha	H1-hESC	None	Sin3Ak-20	ENCODE Mar 2012 Freeze	wgEncodeEH001530
USF1	HudsonAlpha	H1-hESC	None	USF-1	ENCODE Mar 2012 Freeze	wgEncodeEH001532
USF2	Stanford	H1-hESC	None	USF2	ENCODE Mar 2012 Freeze	wgEncodeEH001837
YY1	HudsonAlpha	H1-hESC	None	YY1_(SC-281)	ENCODE Mar 2012 Freeze	wgEncodeEH001567
Znf1	Stanford	H1-hESC	None	Znf143_(16618-1-AP)	ENCODE Mar 2012 Freeze	wgEncodeEH002802

Table S3: **Statistical test for the similarity of species–specific motifs.** For each two species we count for each of the 35 TFs the number of cases where we reject the null hypothesis that two species–specific motifs arise from identical probability distributions. We reject the null hypothesis in 47 of 1575 cases, where these cases only apply to 6 of the 35 TFs and typically only apply to comparisons between a primate species and one of the species dog, cow, and horse.

	hg19	panTro	papHam	ponAbe	rheMac	gorGor	calJac	canFam	equCab	bosTau
hg19		0	0	0	0	0	0	3	4	3
panTro			0	0	0	0	0	3	3	2
papHam				0	0	0	0	0	2	0
ponAbe					0	0	0	3	2	1
rheMac						0	0	2	1	1
gorGor							0	5	5	4
calJac								2	0	0
canFam									0	1
equCab										0
bosTau										

Table S4: **Pairwise comparisons of the four models human(0), human(2), PFM(0), and PFM(2) aggregated for** 35 **TFs.** We show the number of TFs, for which the model in the row outperforms the model in the column. For instance, PFM(2) outperforms PFM(0) in all of the 35 cases. The last column shows the row sum, i.e. how many models are outperformed by the model in the row for all 35 TFs.

	human(0)	human(2)	PFM(0)	PFM(2)	sum
human(0)	-	2	12	1	15
human(2)	33	-	32	4	69
PFM(0)	23	3	-	0	26
PFM(2)	34	31	35	-	100

Table S5: **Comparison of the four models human(0), human(2), PFM(0), and PFM(2) aggregated for** 35 **TFs.** We indicate in the rows whether base dependencies are taken into account and we indicate in the columns whether phylogenetic dependencies are taken into account. Each cell shows the number of TFs for which the respective model (in brackets) outperforms all other models.

	Without Phylogeny	With Phylogeny
Order 0	1 (human(0))	0 (PFM(0))
Order 2	3 (human(2))	31 (PFM(2))

Table S6: **Proportion of binding sites for the calculation of species–specific motifs.** For each of the 35 TFs we show for each of the ten species the number of putative binding site sequences available for the calculation of species–specific motifs relative to the number of putative binding site sequences from human. Dog, horse, and cow show the smallest alignment coverage for each of the 35 TFs.

	hg19	panTro	papHam	ponAbe	rheMac	gorGor	calJac	canFam	equCab	bosTau
ATF3	100 %	96 %	93 %	95 %	90 %	78 %	84 %	61 %	62 %	65 %
Bach1	100 %	96 %	92 %	93 %	90 %	73 %	83 %	58 %	62 %	57 %
BCL11A	100 %	98 %	96 %	96 %	94 %	70 %	89 %	74 %	77 %	68 %
BRCA1	100 %	93 %	93 %	93 %	90 %	73 %	85 %	63 %	63 %	67 %
CEBPB	100 %	97 %	93 %	94 %	91 %	72 %	83 %	60 %	66 %	59 %
CHD2	100 %	94 %	93 %	93 %	90 %	76 %	87 %	67 %	66 %	71 %
CJUN	100 %	97 %	94 %	93 %	92 %	78 %	87 %	66 %	70 %	65 %
CTCF	100 %	96 %	92 %	93 %	89 %	78 %	82 %	54 %	62 %	55 %
EGR1	100 %	94 %	92 %	91 %	88 %	73 %	81 %	54 %	55 %	60 %
FOSL1	100 %	92 %	91 %	90 %	89 %	79 %	83 %	54 %	57 %	61 %
GABP	100 %	95 %	94 %	95 %	91 %	76 %	86 %	62 %	63 %	66 %
JARIDA1A	100 %	96 %	95 %	96 %	90 %	77 %	90 %	70 %	71 %	75 %
JunD	100 %	96 %	94 %	92 %	90 %	77 %	85 %	62 %	65 %	64 %
MafK	100 %	97 %	92 %	94 %	91 %	69 %	83 %	61 %	68 %	58 %
Max	100 %	97 %	93 %	94 %	91 %	77 %	85 %	58 %	65 %	61 %
Mxi	100 %	96 %	93 %	93 %	90 %	75 %	85 %	61 %	65 %	66 %
NANOG	100 %	98 %	94 %	95 %	93 %	74 %	88 %	72 %	75 %	68 %
Nrf	100 %	96 %	92 %	93 %	89 %	75 %	84 %	61 %	59 %	65 %
NRSF	100 %	97 %	93 %	93 %	90 %	73 %	83 %	59 %	66 %	57 %
POU5F1	100 %	97 %	93 %	94 %	92 %	73 %	85 %	66 %	71 %	62 %
Rad21	100 %	96 %	91 %	93 %	89 %	74 %	82 %	55 %	63 %	54 %
RFX5	100 %	94 %	92 %	91 %	90 %	78 %	84 %	65 %	70 %	66 %
RXRA	100 %	97 %	95 %	94 %	91 %	79 %	85 %	61 %	67 %	62 %
Sin3Ak	100 %	96 %	93 %	93 %	91 %	75 %	85 %	62 %	66 %	65 %
SIX5	100 %	95 %	94 %	94 %	90 %	75 %	87 %	71 %	72 %	69 %
SP1	100 %	96 %	93 %	94 %	90 %	78 %	87 %	67 %	68 %	70 %
SP2	100 %	96 %	93 %	95 %	89 %	79 %	88 %	70 %	70 %	75 %
SP4	100 %	94 %	92 %	91 %	87 %	76 %	83 %	60 %	60 %	65 %
SRF	100 %	95 %	93 %	94 %	90 %	78 %	87 %	68 %	67 %	67 %
TCF12	100 %	97 %	93 %	94 %	90 %	75 %	84 %	61 %	66 %	58 %
TEAD4	100 %	97 %	93 %	94 %	91 %	74 %	84 %	61 %	67 %	59 %
USF1	100 %	96 %	92 %	94 %	90 %	77 %	83 %	58 %	62 %	59 %
USF2	100 %	96 %	92 %	94 %	90 %	77 %	84 %	59 %	61 %	62 %
YY1	100 %	95 %	94 %	94 %	90 %	77 %	87 %	67 %	66 %	69 %
Znf143	100 %	96 %	92 %	93 %	89 %	77 %	82 %	57 %	62 %	58 %

References

[1] Ralf Eggeling, Teemu Roos, Petri Myllymäki, and Ivo Grosse. Inferring intra-motif dependencies of dna binding sites from chip-seq data. *BMC bioinformatics*, 16(1):375, 2015.

[2] Charles E Lawrence and Andrew A Reilly. An expectation maximization (em) algorithm for the identification and characterization of common sites in unaligned biopolymer sequences. *Proteins: Structure, Function, and Bioinformatics*, 7(1):41–51, 1990.

[3] Martin Nettling, Hendrik Treutler, Jan Grau, Jens Keilwagen, Stefan Posch, and Ivo Grosse. Difflogo: a comparative visualization of sequence motifs. *BMC bioinformatics*, 16(1):1, 2015.
