# Supplementary material for: Combining phylogenetic footprinting with motif models incorporating intra-motif dependencies
Source: BMC Bioinformatics. 2017 Mar 1;18:141. doi: 10.1186/s12859-017-1495-1 (PMC5333389; doi:10.1186/s12859-017-1495-1)
Supplement: Supplementary file 3 — Sequence logos, MI profiles of order 1, MI profiles of order 2, and species-specific MI profiles of orders 1 and 2. The file contains for each of the 35 TFs the sequence logo inferred using the PFM(2) aligned with MI profiles of order 1, the MI profiles of order 2, and species-specific MI profiles of orders 1 and 2 for each of the 10 species. (PDF 2129.92 kb) [file 12859_2017_1495_MOESM3_ESM.pdf]

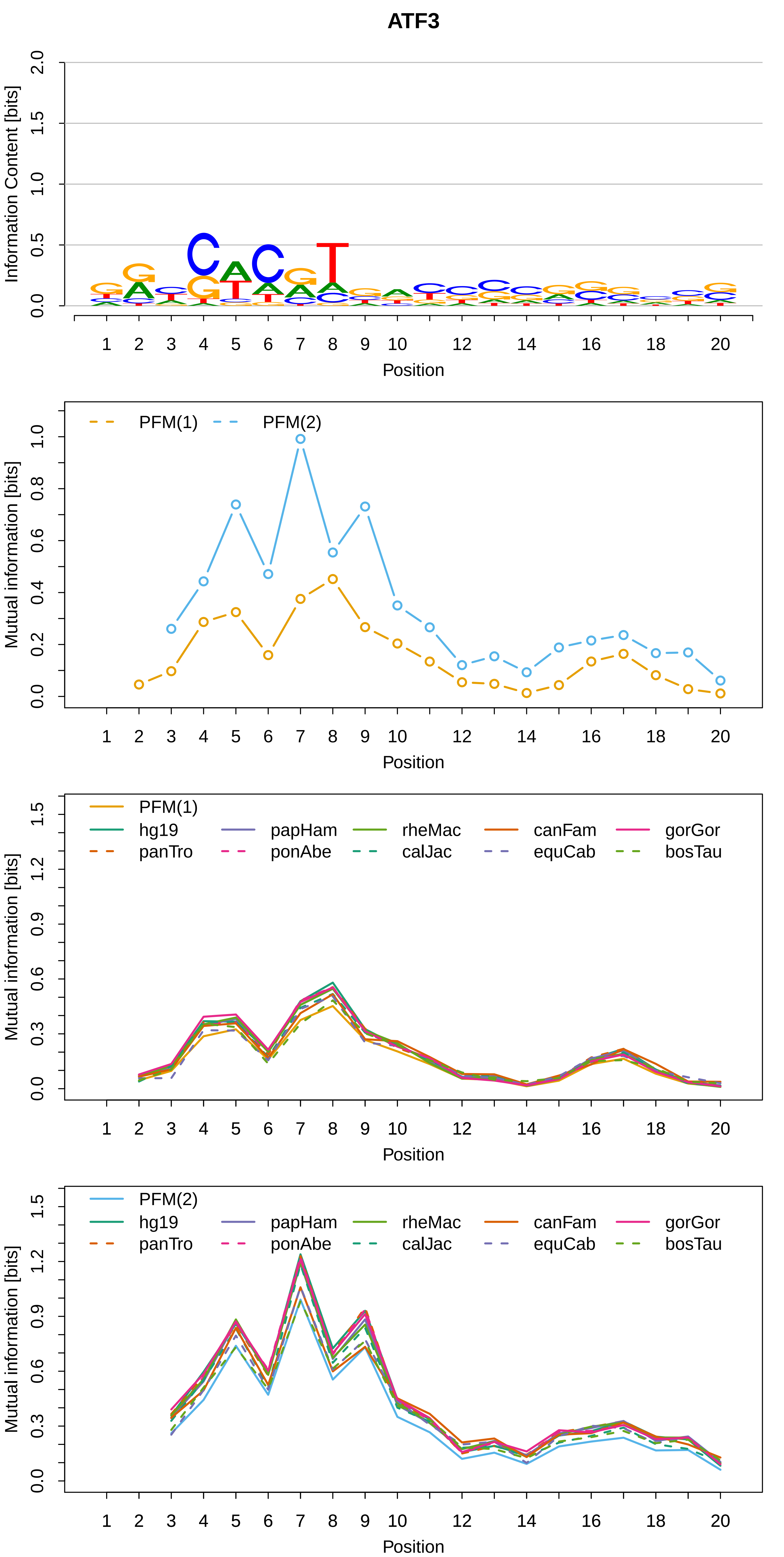

ATF3
Information Content [bits]
Position
Mutual information [bits]
PFM(1)
PFM(2)
hg19
papHam
rheMac
canFam
gorGor
panTro
ponAbe
calJac
equCab
bosTau

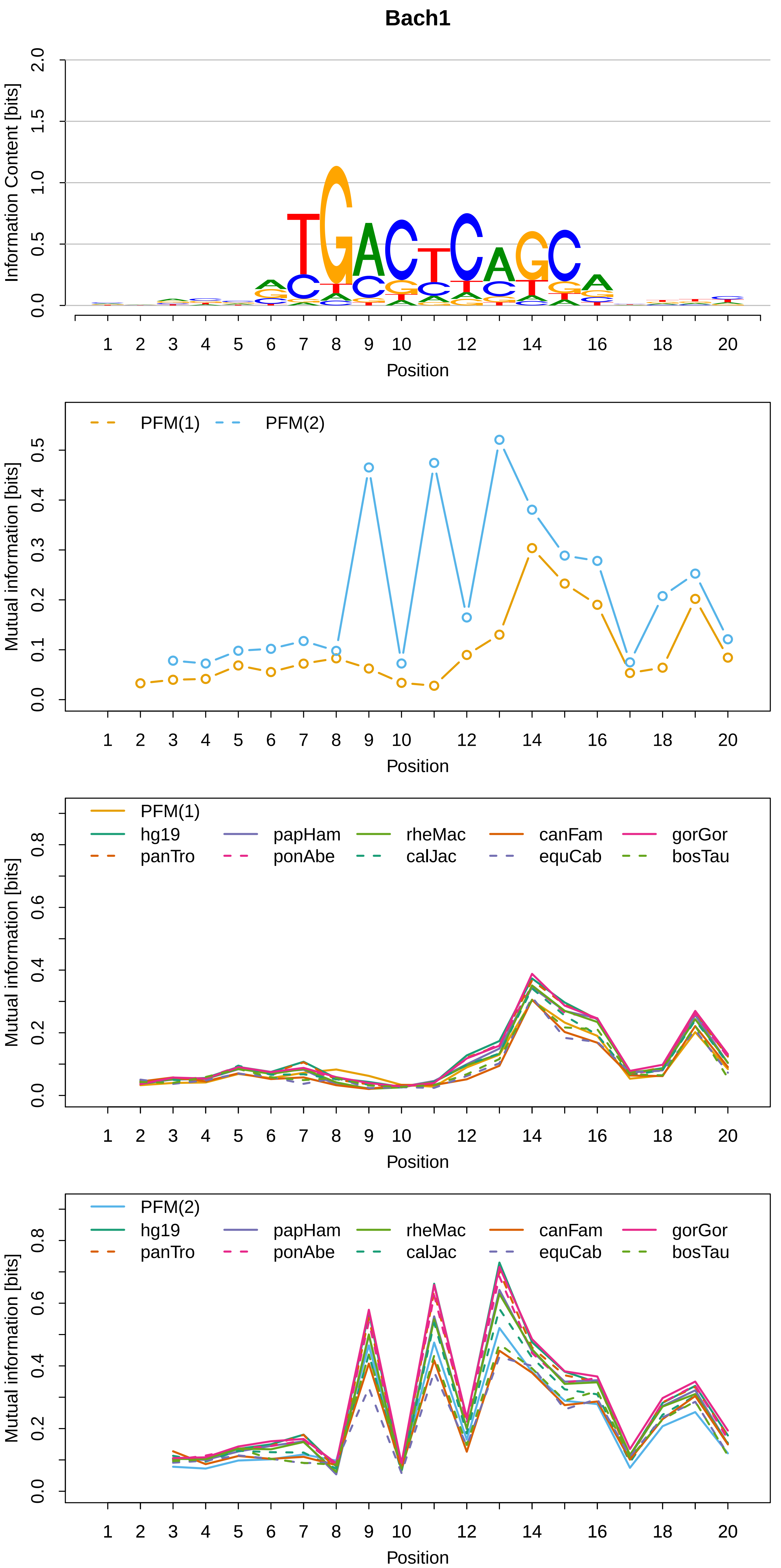
Bach1
Information Content [bits]
Position
Mutual information [bits]
PFM(1)
PFM(2)
hg19
panTro
papHam
ponAbe
rheMac
calJac
canFam
equCab
gorGor
bosTau

BCL11A

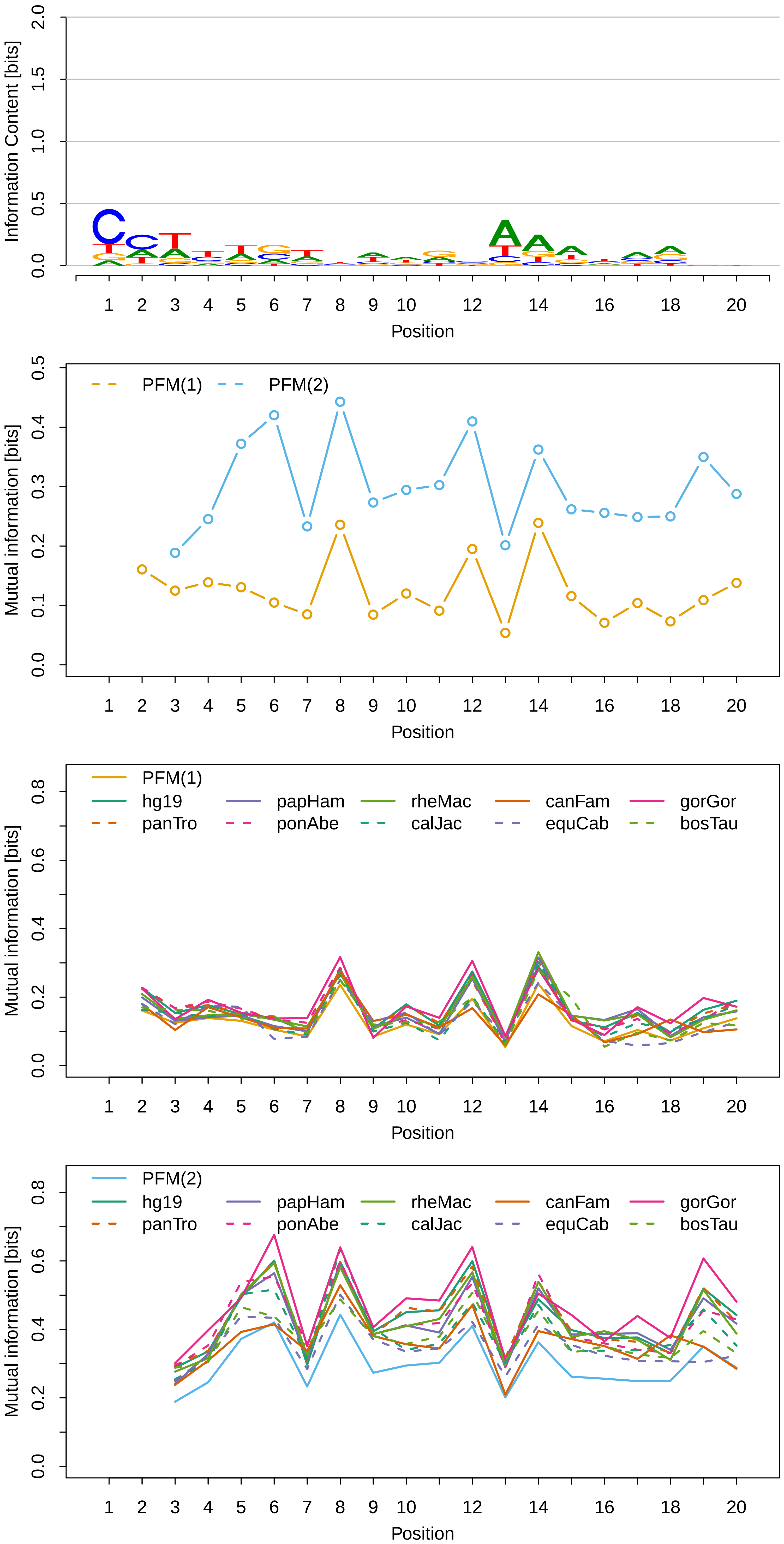
Information Content [bits]
0.0
0.5
1.0
1.5
2.0
1
2
3
4
5
6
7
8
9
10
12
14
16
18
20
Position
Mutual information [bits]
0.0
0.1
0.2
0.3
0.4
0.5
PFM(1)
PFM(2)
Position
Mutual information [bits]
0.0
0.2
0.4
0.6
0.8
PFM(1)
hg19
papHam
rheMac
canFam
gorGor
panTro
ponAbe
calJac
equCab
bosTau
Position
Mutual information [bits]
0.0
0.2
0.4
0.6
0.8
PFM(2)
hg19
papHam
rheMac
canFam
gorGor
panTro
ponAbe
calJac
equCab
bosTau
Position

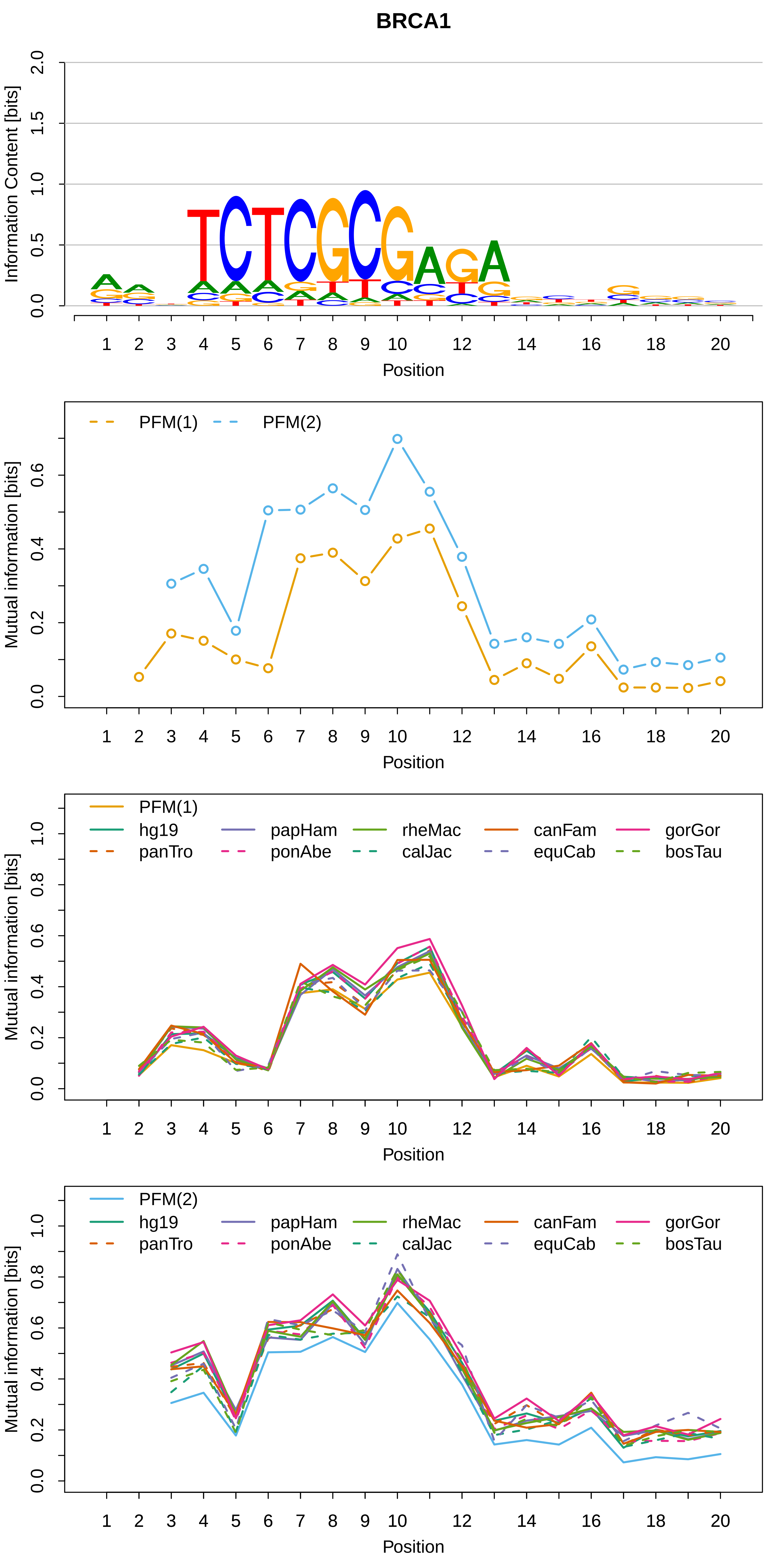

BRCA1
Information Content [bits]
Position
Mutual information [bits]
PFM(1)
PFM(2)
hg19
panTro
papHam
ponAbe
rheMac
calJac
canFam
equCab
gorGor
bosTau

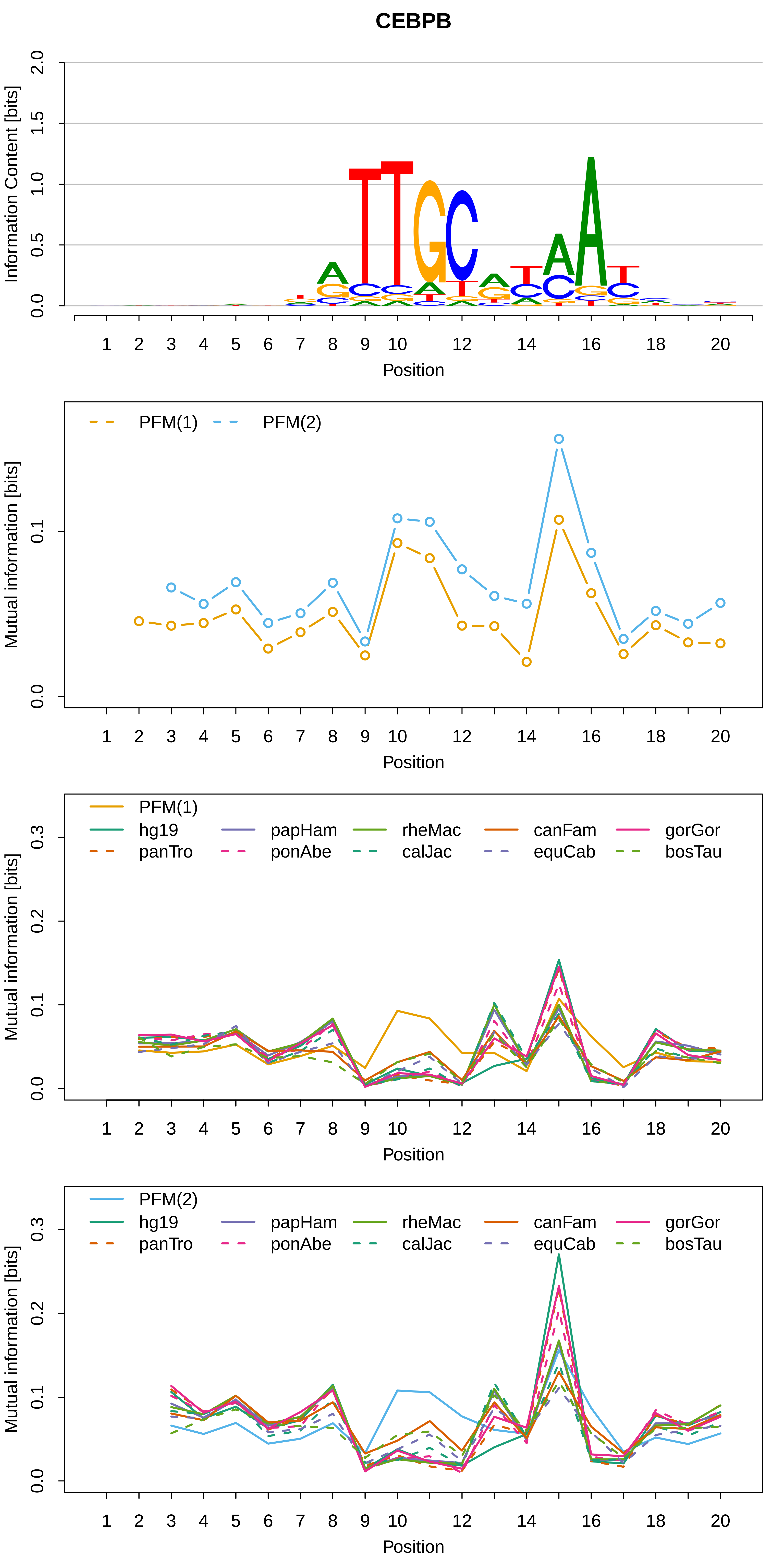

CEBPB
Information Content [bits]
Position
Mutual information [bits]
PFM(1)
PFM(2)
hg19
panTro
papHam
ponAbe
rheMac
calJac
canFam
equCab
gorGor
bosTau

CHD2

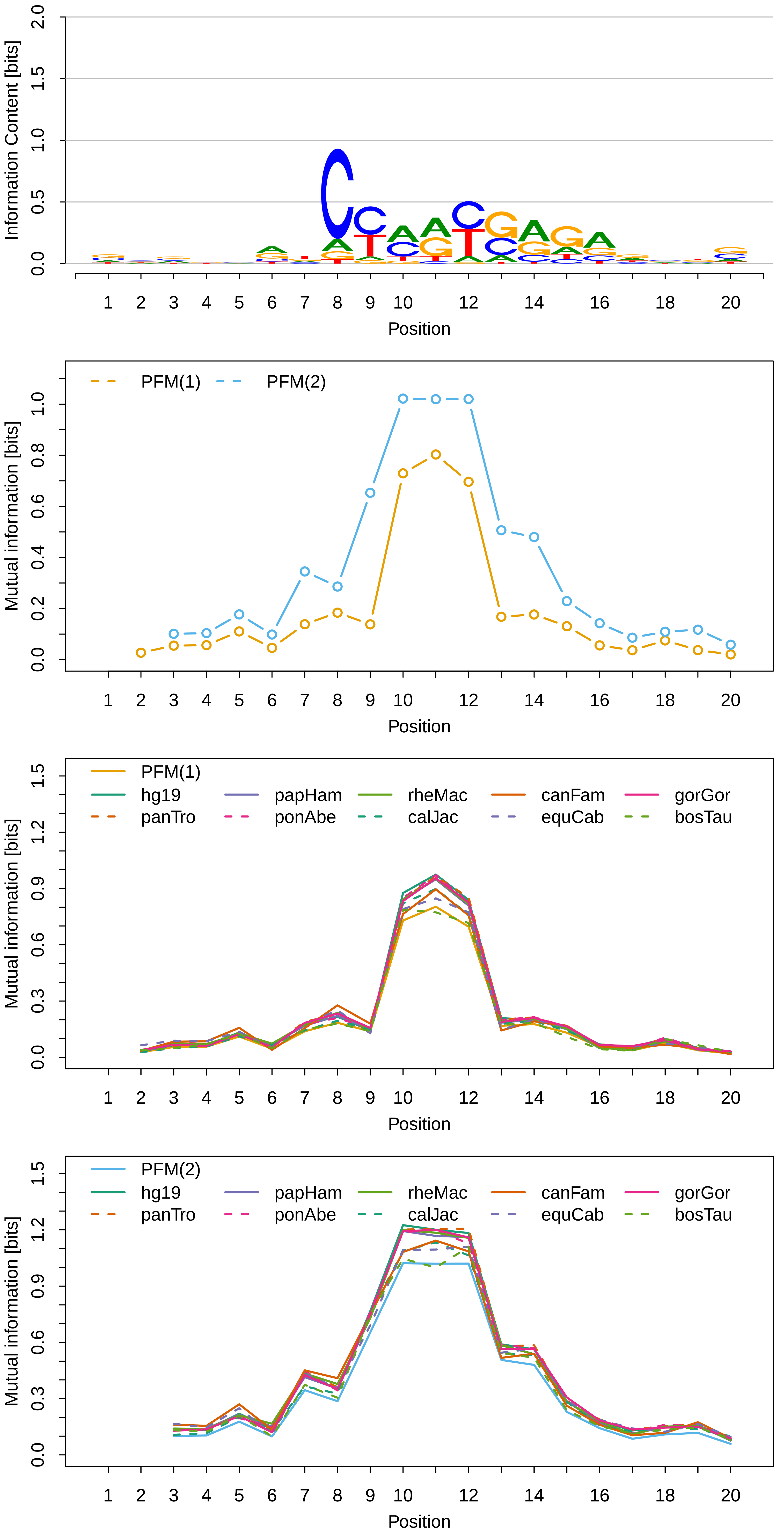
Information Content [bits]
Position
PFM(1)
PFM(2)
Mutual information [bits]
Position
PFM(1)
hg19
papHam
rheMac
canFam
gorGor
panTro
ponAbe
calJac
equCab
bosTau
Mutual information [bits]
Position
PFM(2)
hg19
papHam
rheMac
canFam
gorGor
panTro
ponAbe
calJac
equCab
bosTau
Mutual information [bits]
Position

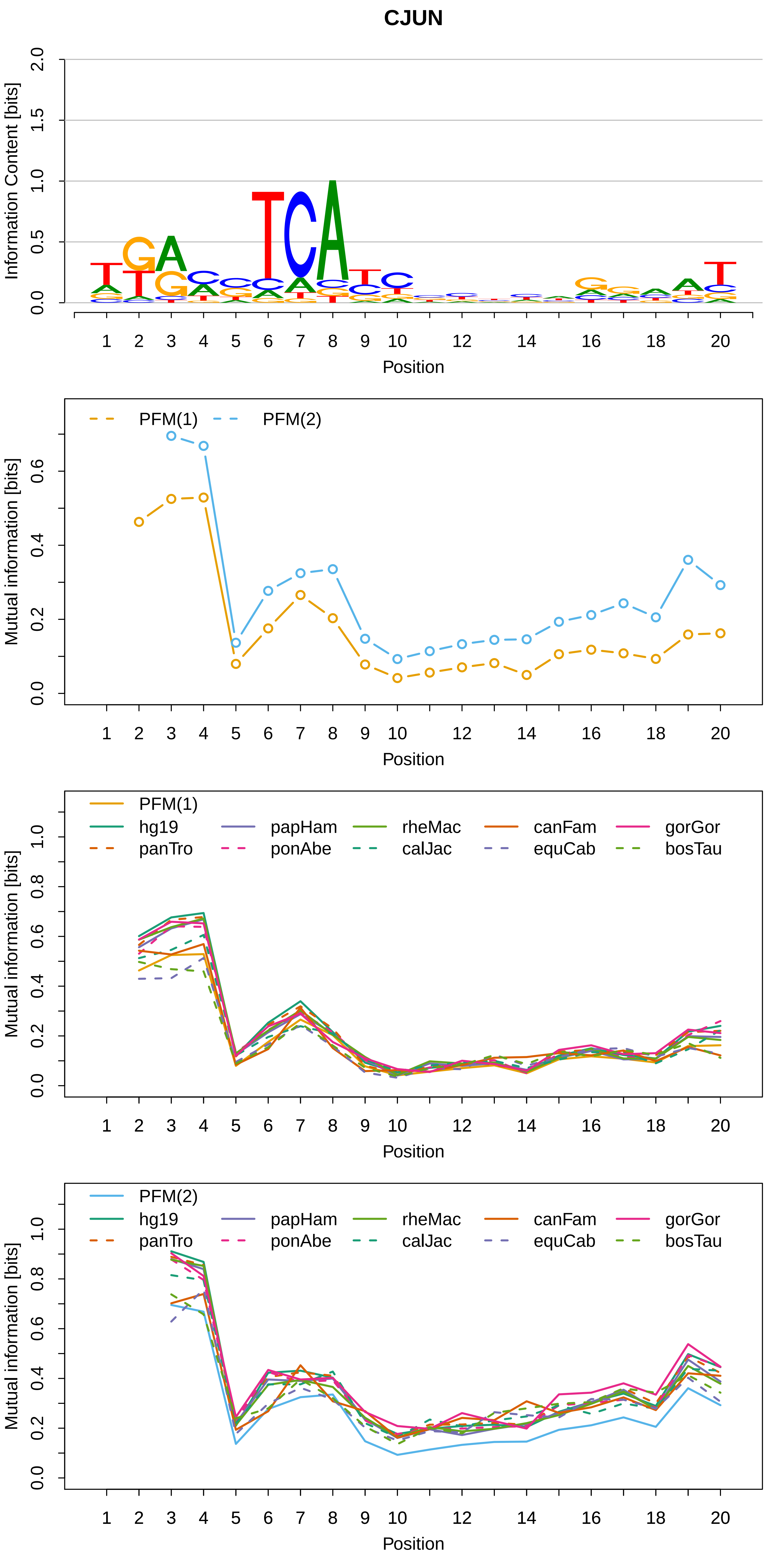

CJUN
Information Content [bits]
0.0
0.5
1.0
1.5
2.0
1
2
3
4
5
6
7
8
9
10
12
14
16
18
20
Position
PFM(1)
PFM(2)
Mutual information [bits]
0.0
0.2
0.4
0.6
Position
PFM(1)
hg19
panTro
papHam
ponAbe
rheMac
calJac
canFam
equCab
gorGor
bosTau
Mutual information [bits]
0.0
0.2
0.4
0.6
0.8
1.0
Position
PFM(2)
hg19
panTro
papHam
ponAbe
rheMac
calJac
canFam
equCab
gorGor
bosTau
Mutual information [bits]
Position

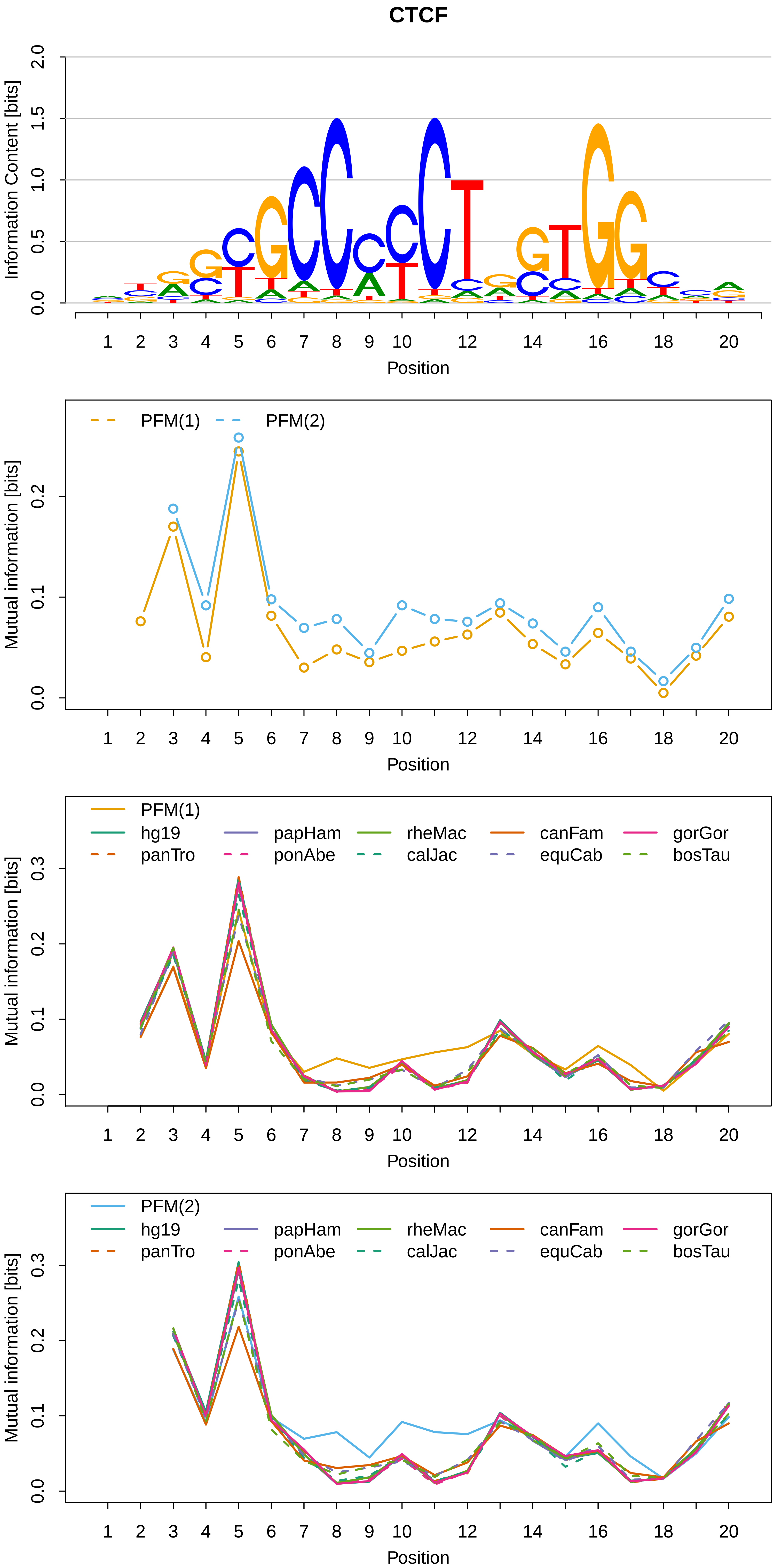
CTCF
Information Content [bits]
0.0
0.5
1.0
1.5
2.0
1 2 3 4 5 6 7 8 9 10 12 14 16 18 20
Position
PFM(1)
PFM(2)
Mutual information [bits]
0.0
0.1
0.2
1 2 3 4 5 6 7 8 9 10 12 14 16 18 20
Position
PFM(1)
hg19
papHam
rheMac
canFam
gorGor
panTro
ponAbe
calJac
equCab
bosTau
Mutual information [bits]
0.0
0.1
0.2
0.3
1 2 3 4 5 6 7 8 9 10 12 14 16 18 20
Position
PFM(2)
hg19
papHam
rheMac
canFam
gorGor
panTro
ponAbe
calJac
equCab
bosTau
Mutual information [bits]
0.0
0.1
0.2
0.3
1 2 3 4 5 6 7 8 9 10 12 14 16 18 20
Position

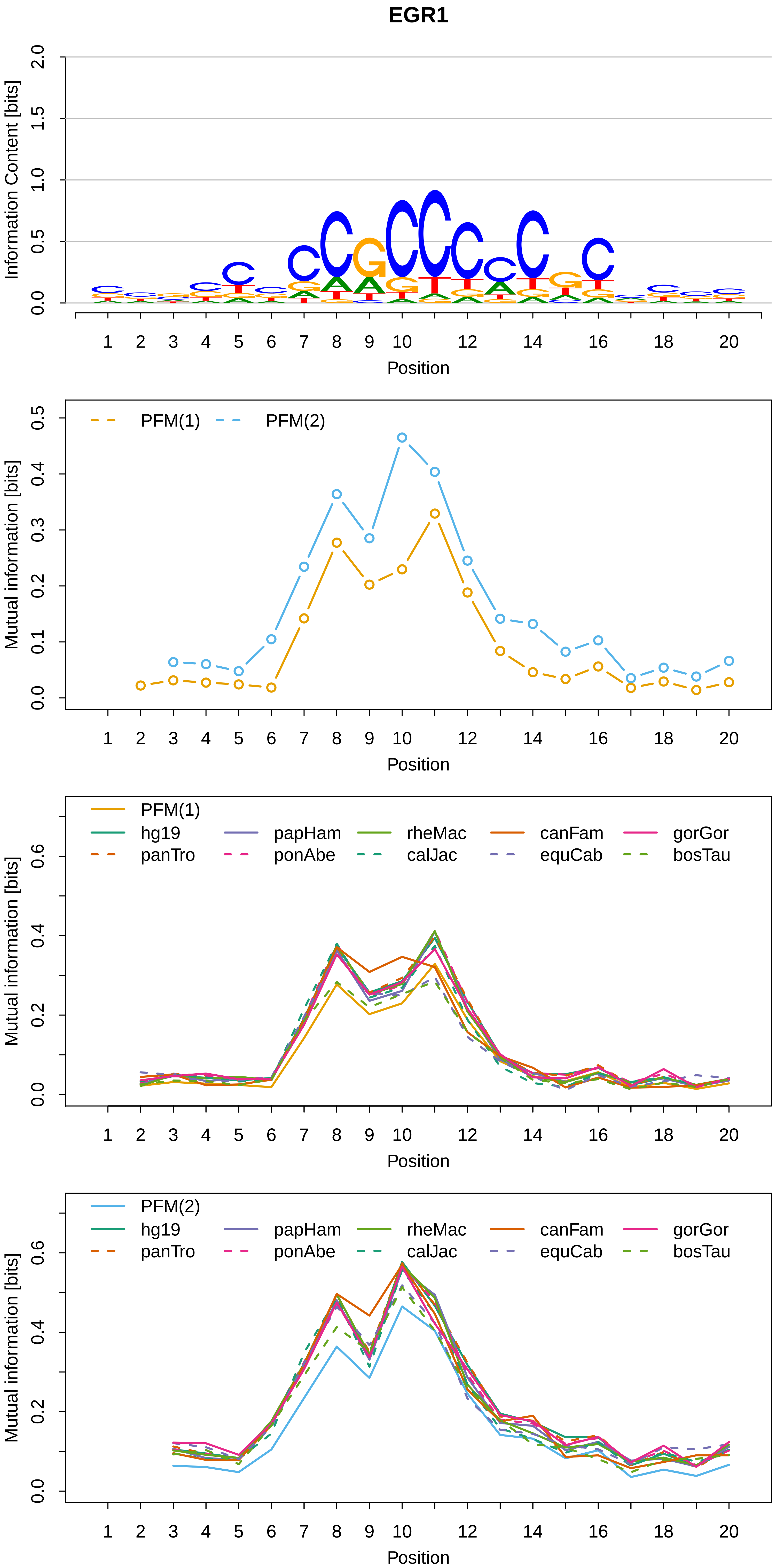
EGR1
Information Content [bits]
0.0
0.5
1.0
1.5
2.0
1 2 3 4 5 6 7 8 9 10 12 14 16 18 20
Position
PFM(1)
PFM(2)
Mutual information [bits]
0.0
0.1
0.2
0.3
0.4
0.5
1 2 3 4 5 6 7 8 9 10 12 14 16 18 20
Position
PFM(1)
hg19
papHam
rheMac
canFam
gorGor
panTro
ponAbe
calJac
equCab
bosTau
Mutual information [bits]
0.0
0.2
0.4
0.6
1 2 3 4 5 6 7 8 9 10 12 14 16 18 20
Position
PFM(2)
hg19
papHam
rheMac
canFam
gorGor
panTro
ponAbe
calJac
equCab
bosTau
Mutual information [bits]
0.0
0.2
0.4
0.6
1 2 3 4 5 6 7 8 9 10 12 14 16 18 20
Position

FOSL1

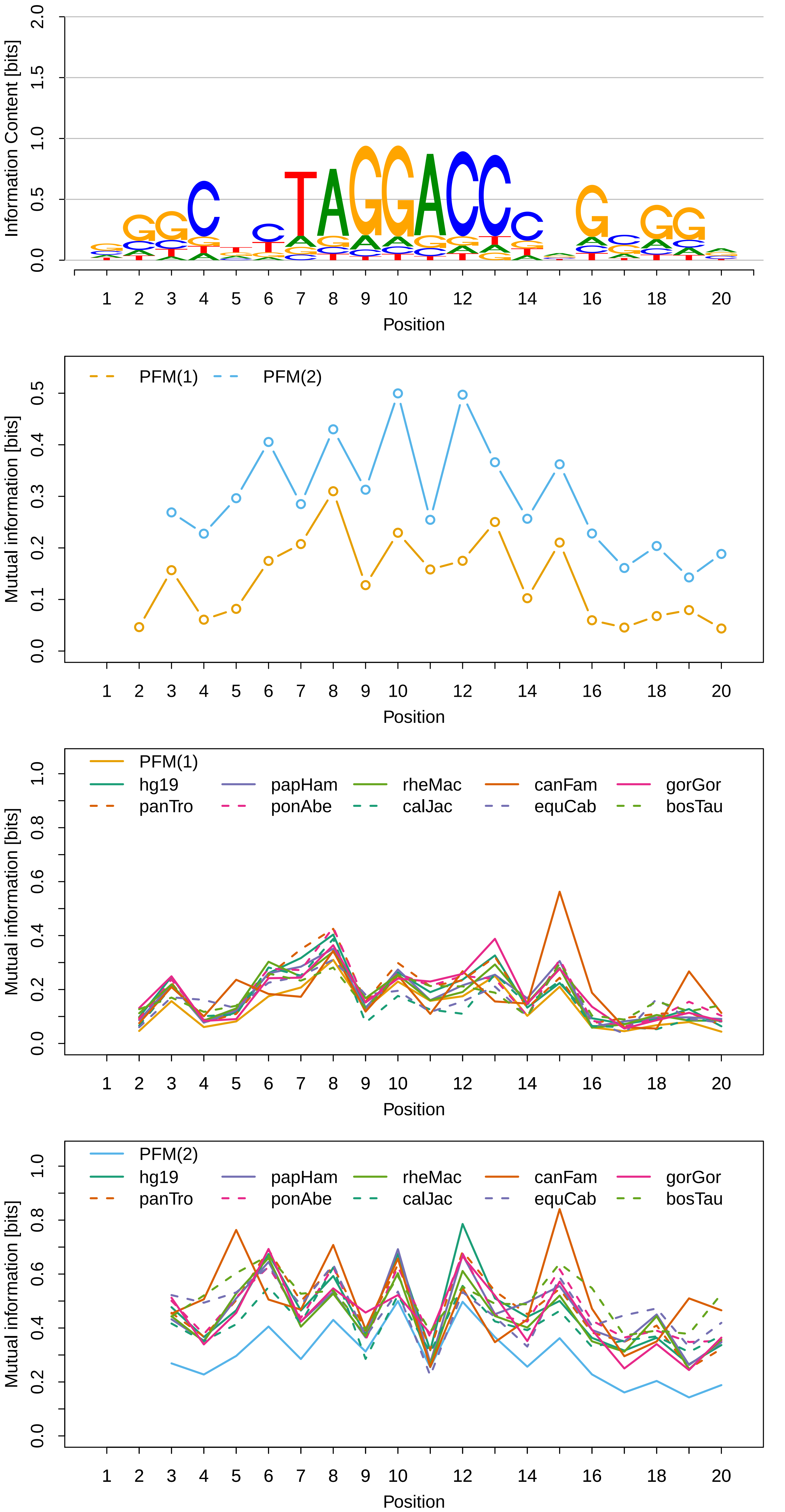

Information Content [bits]
Position
PFM(1)
PFM(2)
Mutual information [bits]
Position
PFM(1)
hg19
papHam
rheMac
canFam
gorGor
panTro
ponAbe
calJac
equCab
bosTau
Mutual information [bits]
Position
PFM(2)
hg19
papHam
rheMac
canFam
gorGor
panTro
ponAbe
calJac
equCab
bosTau
Mutual information [bits]
Position

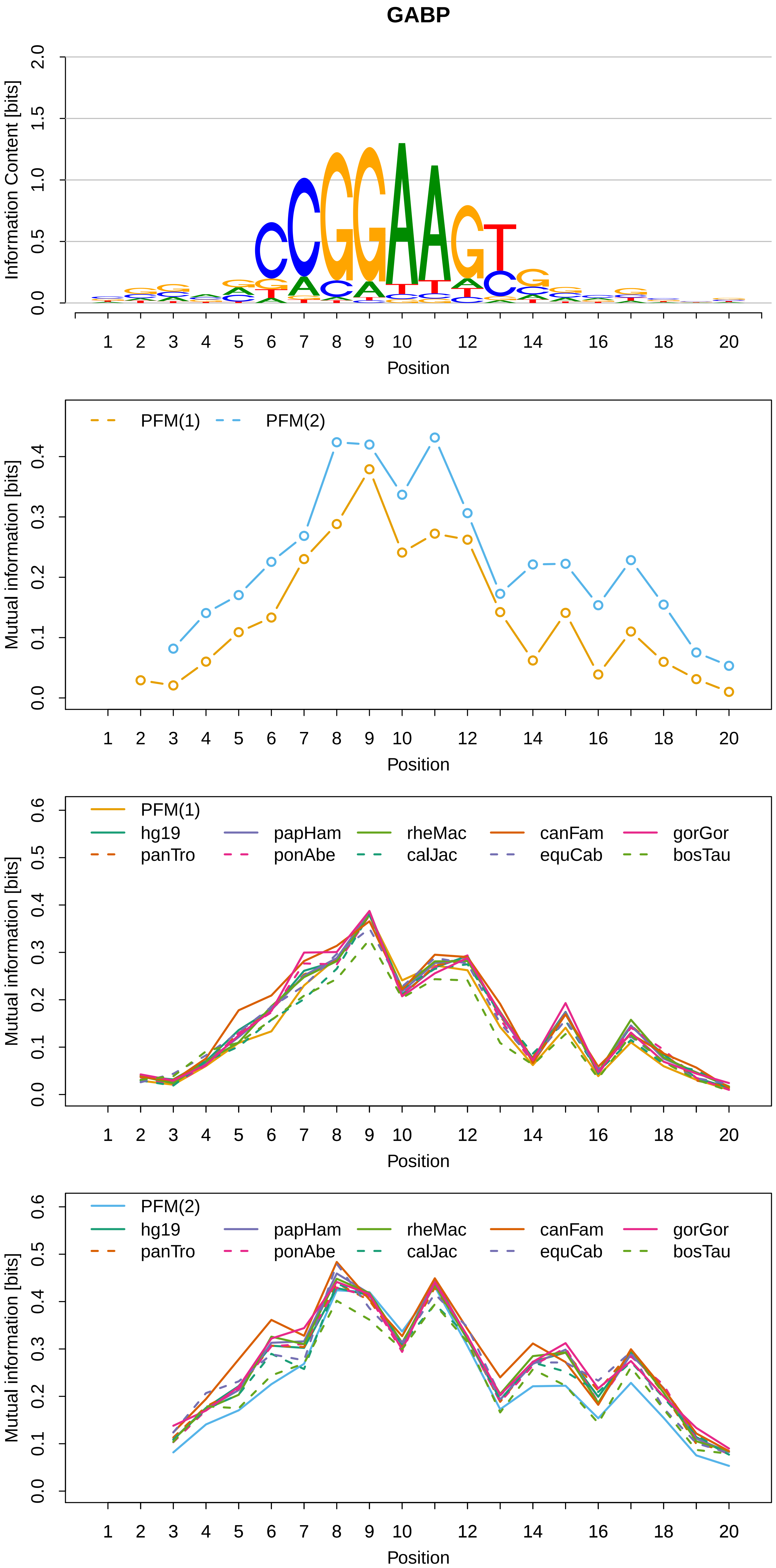

GABP
Information Content [bits]
Position
PFM(1)
PFM(2)
Mutual information [bits]
hg19
panTro
papHam
ponAbe
rheMac
calJac
canFam
equCab
gorGor
bosTau

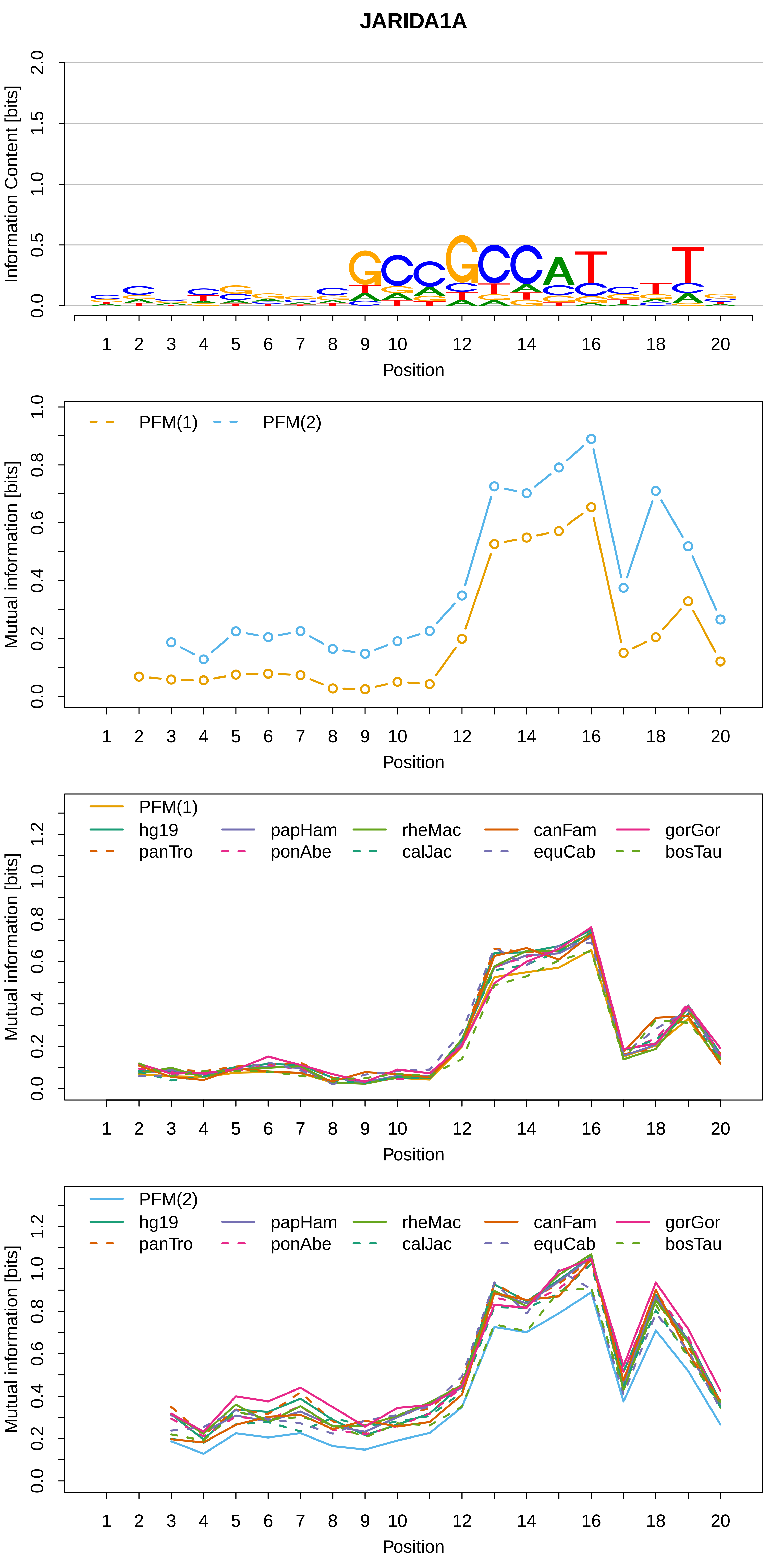
JARIDA1A
Information Content [bits]
Position
Mutual information [bits]
PFM(1)
PFM(2)
Position
Mutual information [bits]
PFM(1)
hg19
panTro
papHam
ponAbe
rheMac
calJac
canFam
equCab
gorGor
bosTau
Position
Mutual information [bits]
PFM(2)
hg19
panTro
papHam
ponAbe
rheMac
calJac
canFam
equCab
gorGor
bosTau
Position

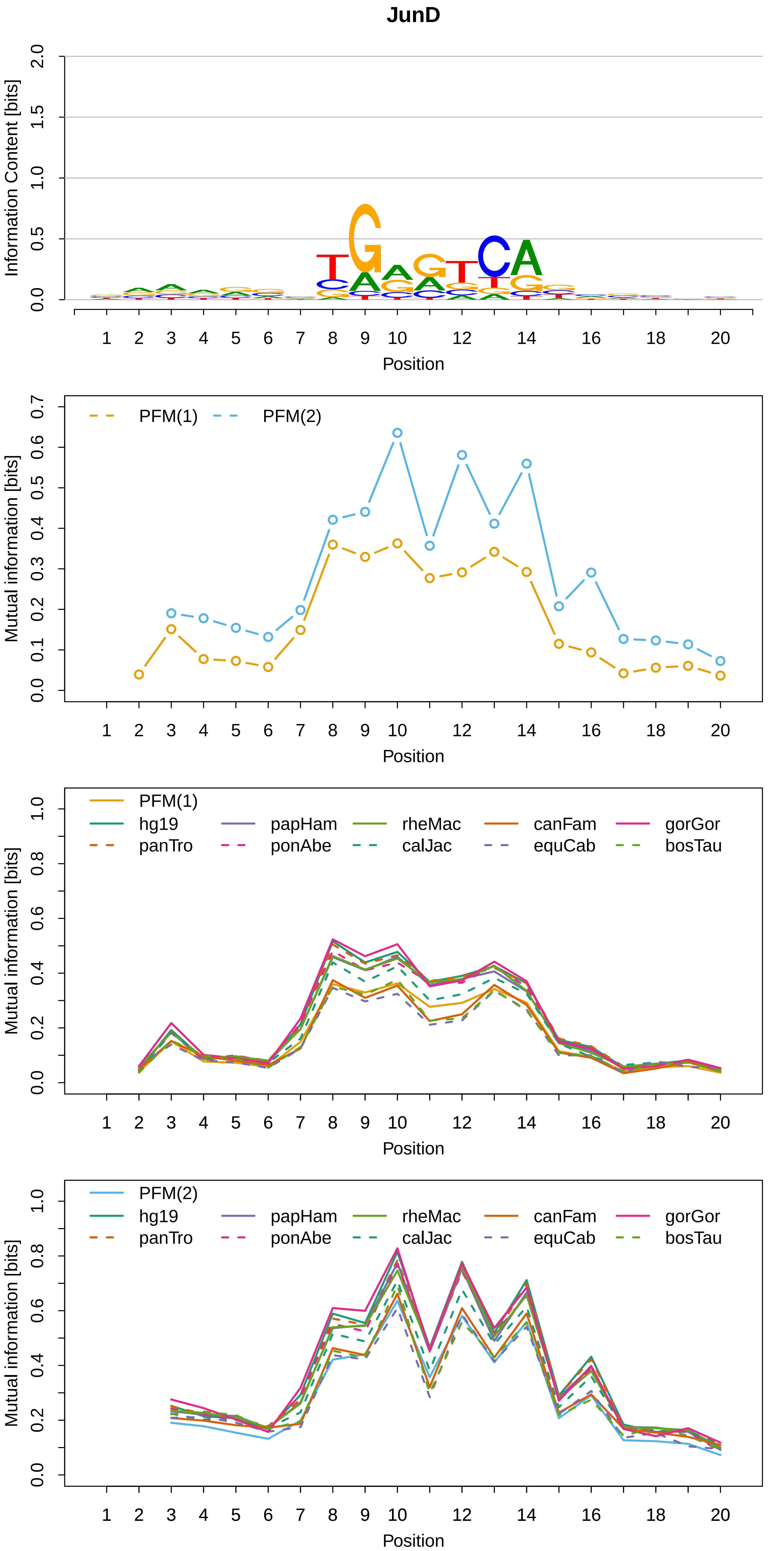
JunD
Information Content [bits]
Position
PFM(1)
PFM(2)
Mutual information [bits]
Position
PFM(1)
hg19
panTro
papHam
ponAbe
rheMac
calJac
canFam
equCab
gorGor
bosTau
Mutual information [bits]
Position
PFM(2)
hg19
panTro
papHam
ponAbe
rheMac
calJac
canFam
equCab
gorGor
bosTau
Mutual information [bits]
Position

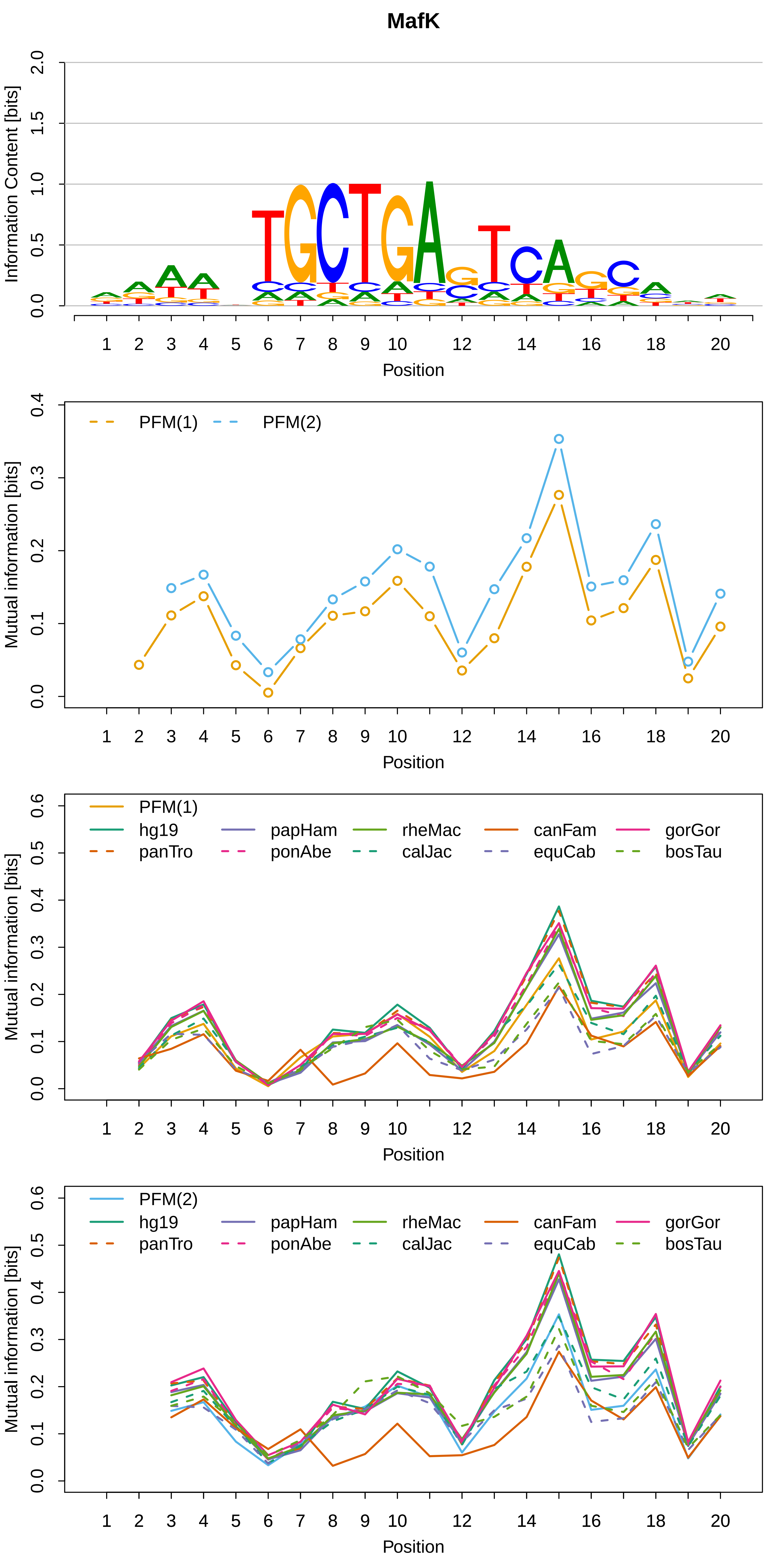

MafK
Information Content [bits]
Mutual information [bits]
Position
PFM(1)
PFM(2)
hg19
panTro
papHam
ponAbe
rheMac
calJac
canFam
equCab
gorGor
bosTau

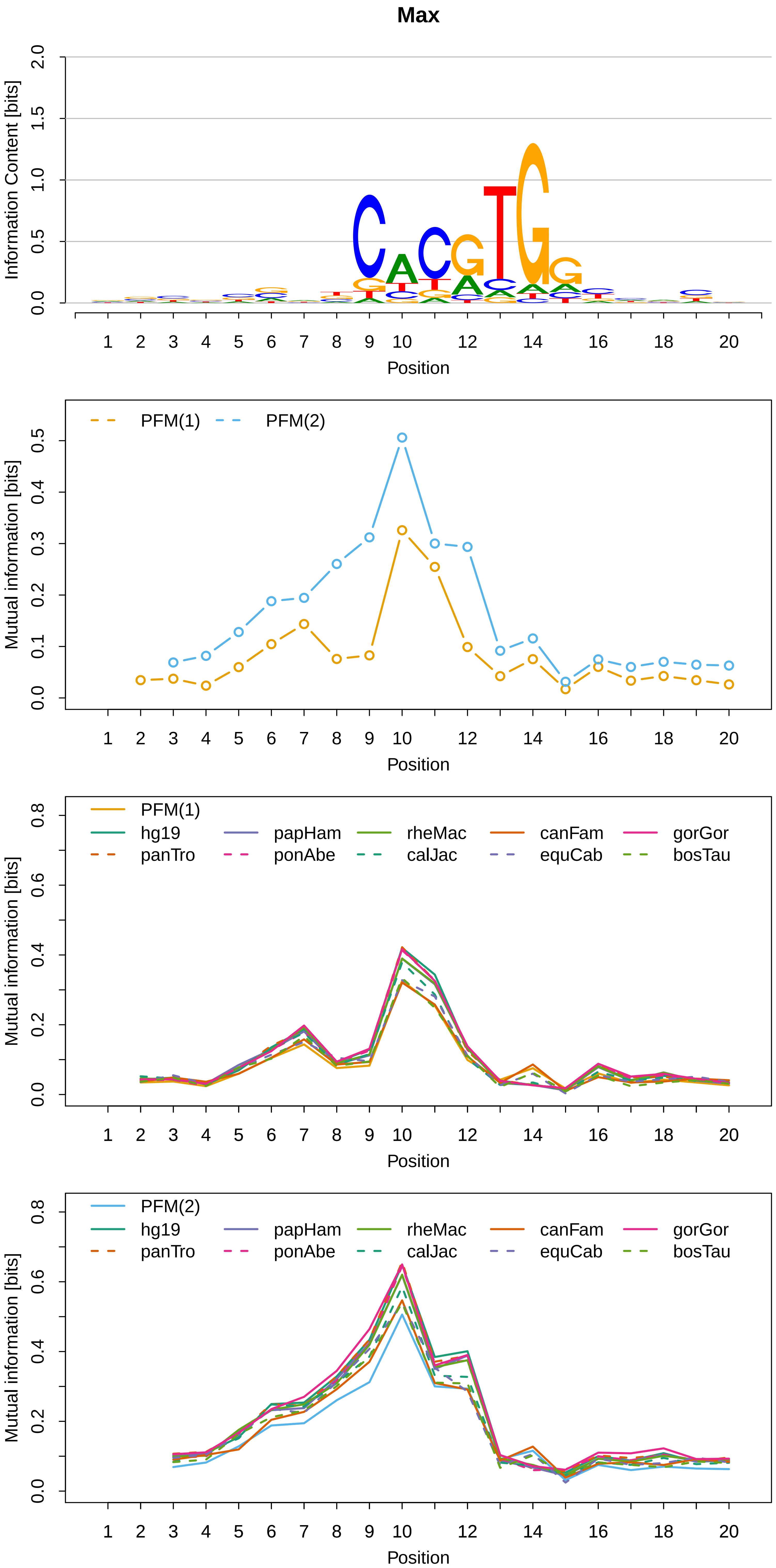
Max
Information Content [bits]
Position
PFM(1)
PFM(2)
Mutual information [bits]
hg19
panTro
papHam
ponAbe
rheMac
calJac
canFam
equCab
gorGor
bosTau

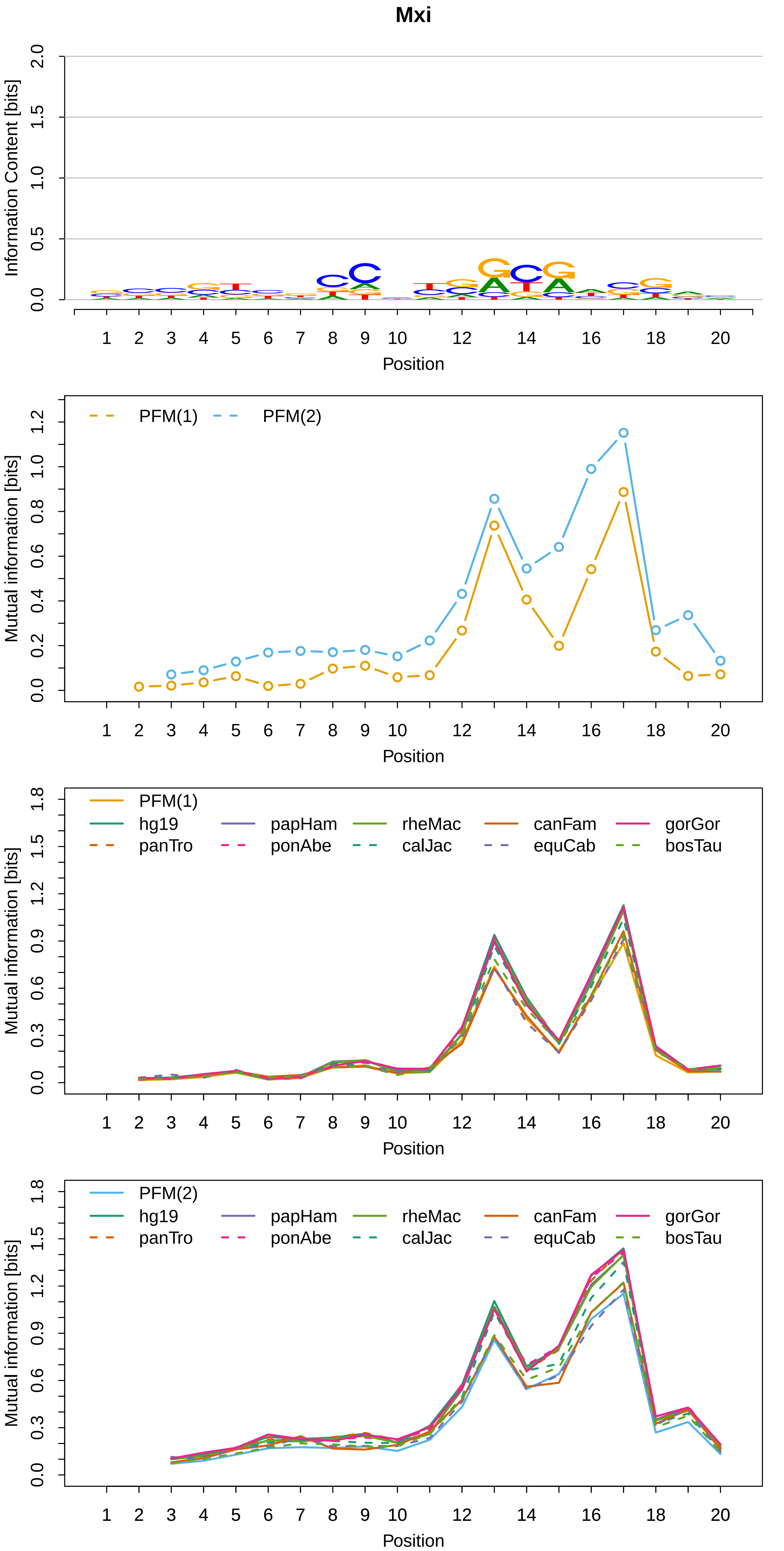

Mxi
Information Content [bits]
Position
Mutual information [bits]
PFM(1)
PFM(2)
hg19
panTro
papHam
ponAbe
rheMac
calJac
canFam
equCab
gorGor
bosTau

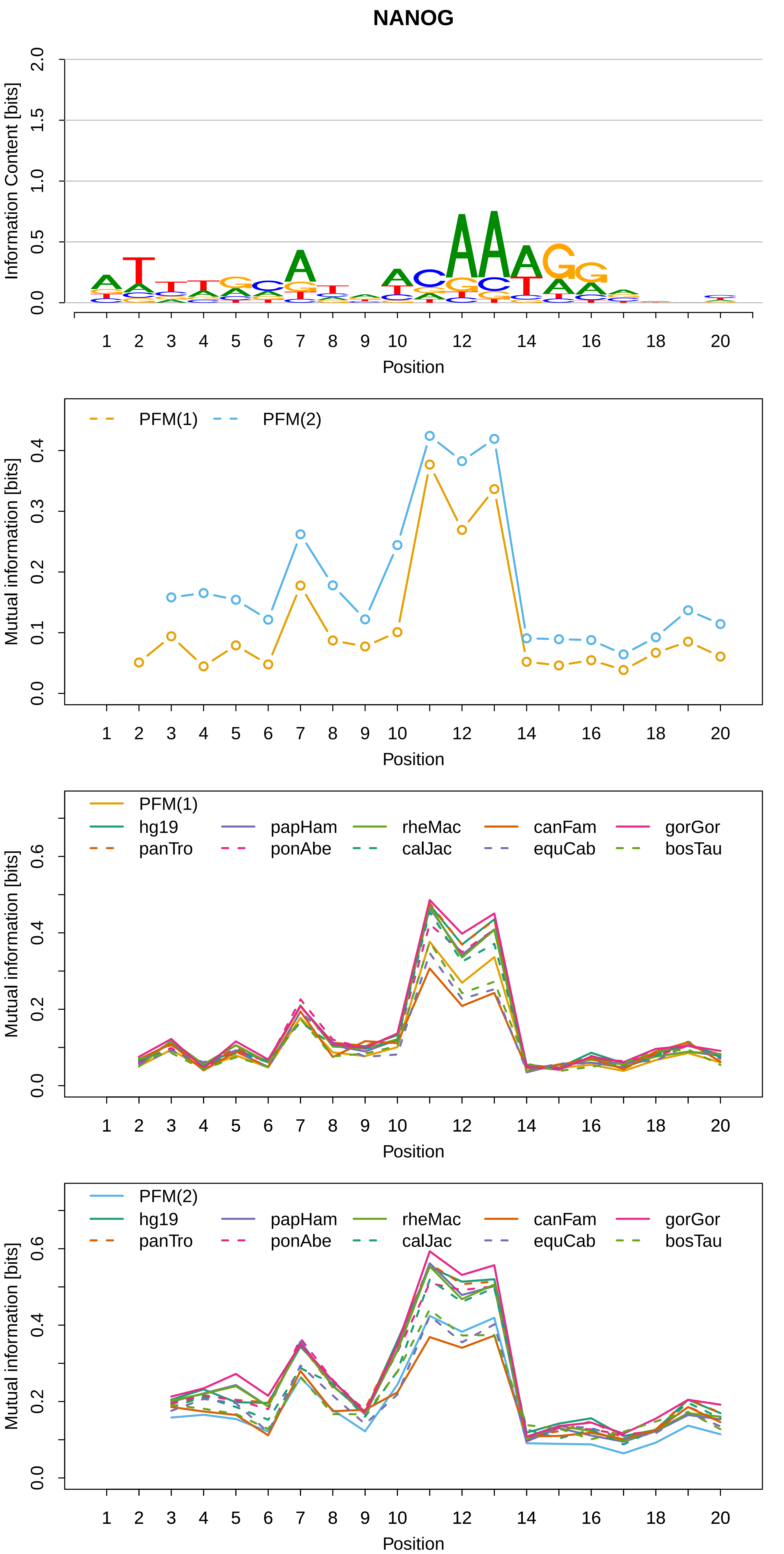
NANOG
Information Content [bits]
Position
PFM(1)
PFM(2)
Mutual information [bits]
hg19
panTro
papHam
ponAbe
rheMac
calJac
canFam
equCab
gorGor
bosTau

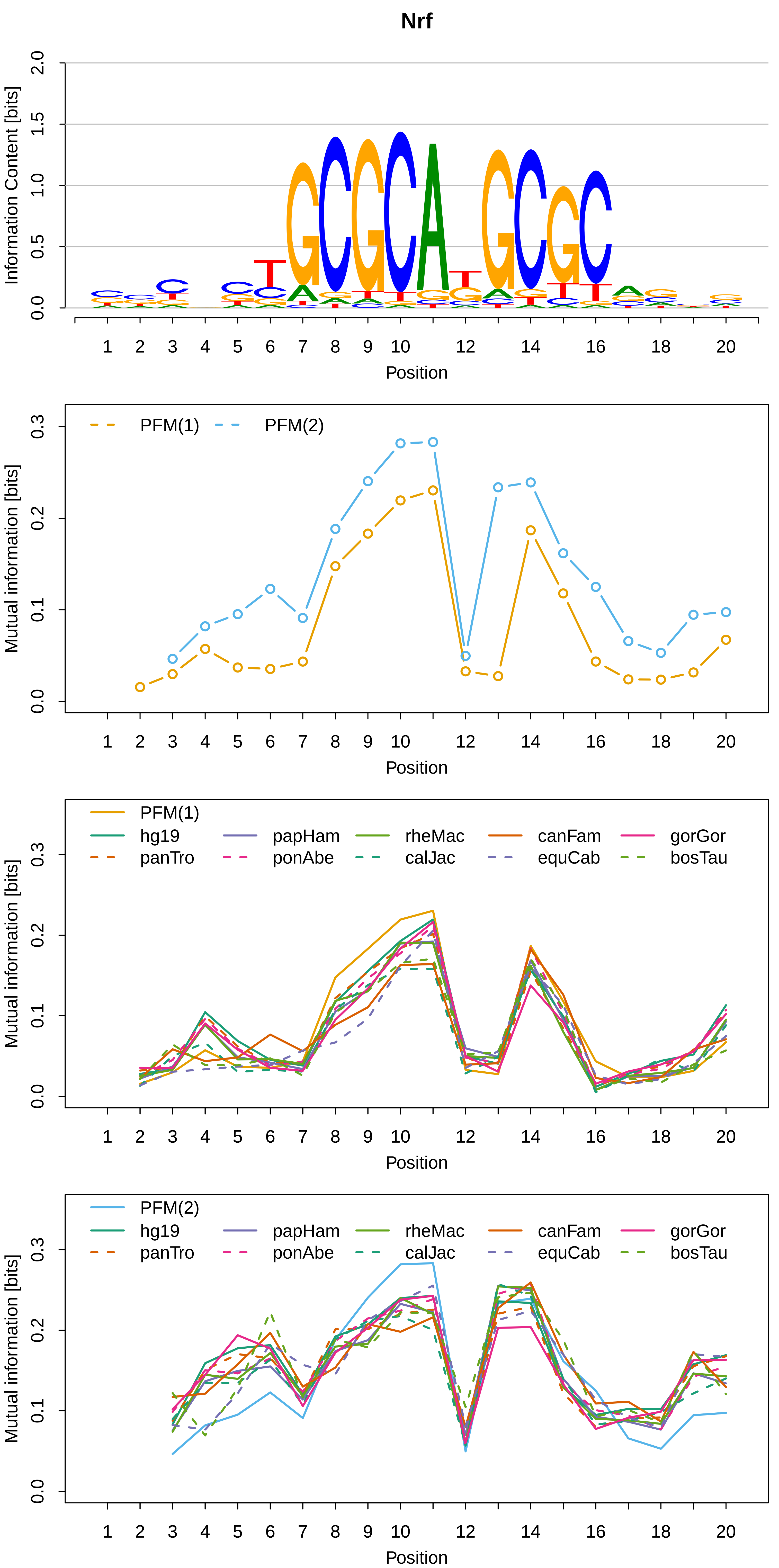
Nrf
Information Content [bits]
0.0
0.5
1.0
1.5
2.0
1 2 3 4 5 6 7 8 9 10 12 14 16 18 20
Position
Mutual information [bits]
0.0
0.1
0.2
0.3
PFM(1)
PFM(2)
1 2 3 4 5 6 7 8 9 10 12 14 16 18 20
Position
Mutual information [bits]
PFM(1)
hg19
panTro
papHam
ponAbe
rheMac
calJac
canFam
equCab
gorGor
bosTau
1 2 3 4 5 6 7 8 9 10 12 14 16 18 20
Position
Mutual information [bits]
PFM(2)
hg19
panTro
papHam
ponAbe
rheMac
calJac
canFam
equCab
gorGor
bosTau
1 2 3 4 5 6 7 8 9 10 12 14 16 18 20
Position

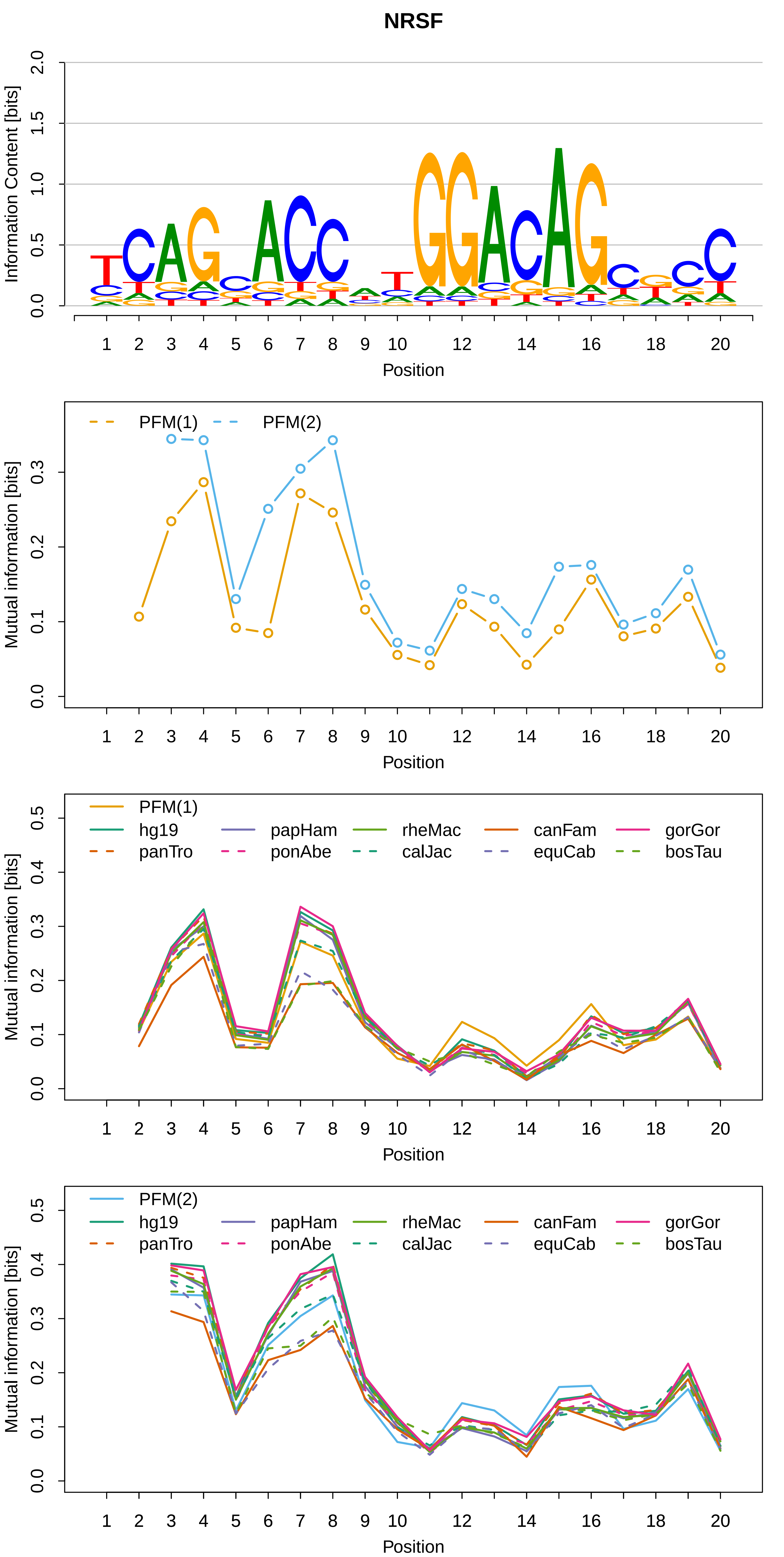

NRSF
Information Content [bits]
0.0
0.5
1.0
1.5
2.0
1
2
3
4
5
6
7
8
9
10
12
14
16
18
20
Position
PFM(1)
PFM(2)
Mutual information [bits]
0.0
0.1
0.2
0.3
Position
PFM(1)
hg19
panTro
papHam
ponAbe
rheMac
calJac
canFam
equCab
gorGor
bosTau
Mutual information [bits]
0.0
0.1
0.2
0.3
0.4
0.5
Position
PFM(2)
hg19
panTro
papHam
ponAbe
rheMac
calJac
canFam
equCab
gorGor
bosTau
Mutual information [bits]
Position

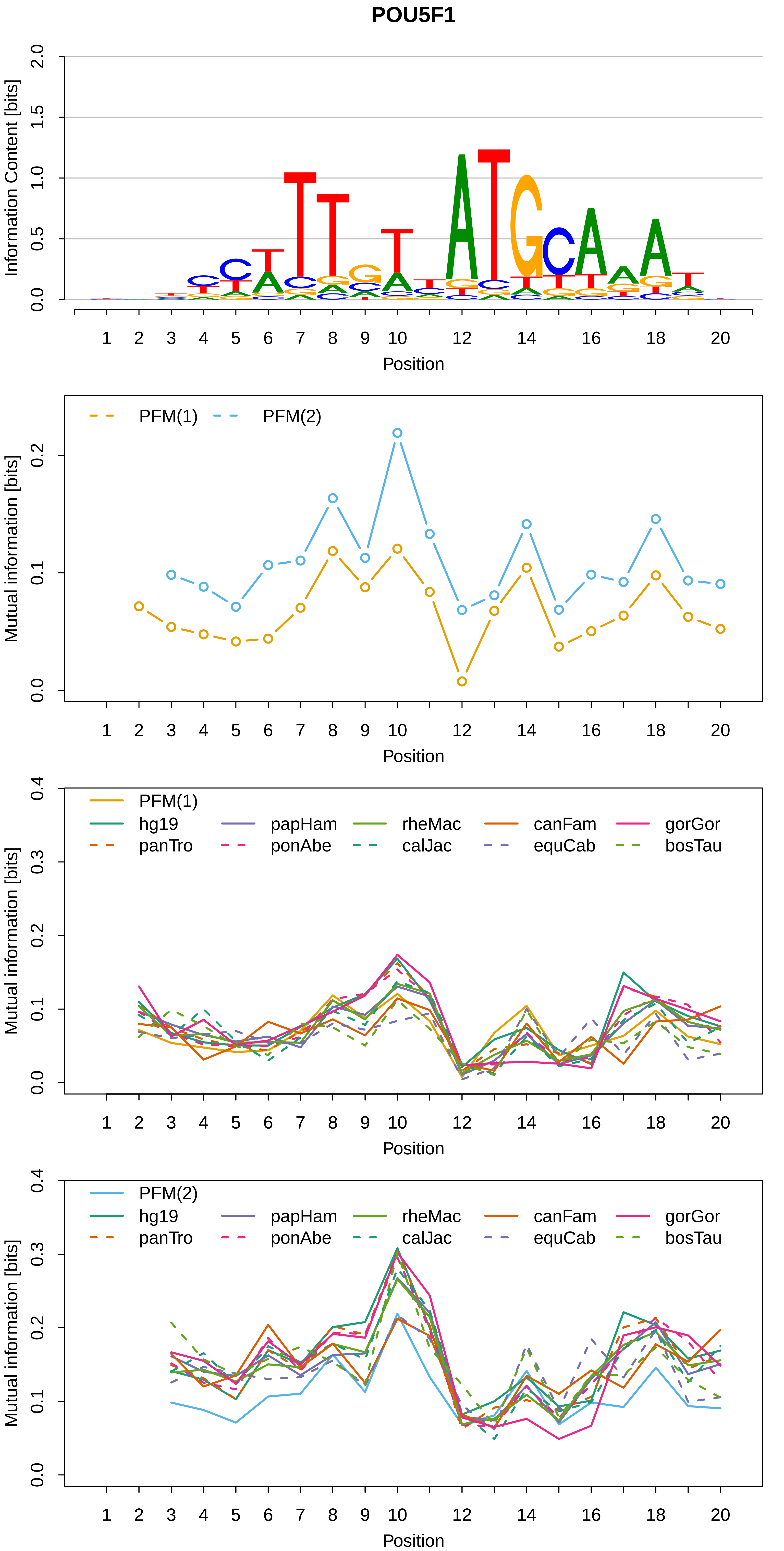

POU5F1
Information Content [bits]
Position
Mutual information [bits]
PFM(1)
PFM(2)
hg19
panTro
papHam
ponAbe
rheMac
calJac
canFam
equCab
gorGor
bosTau

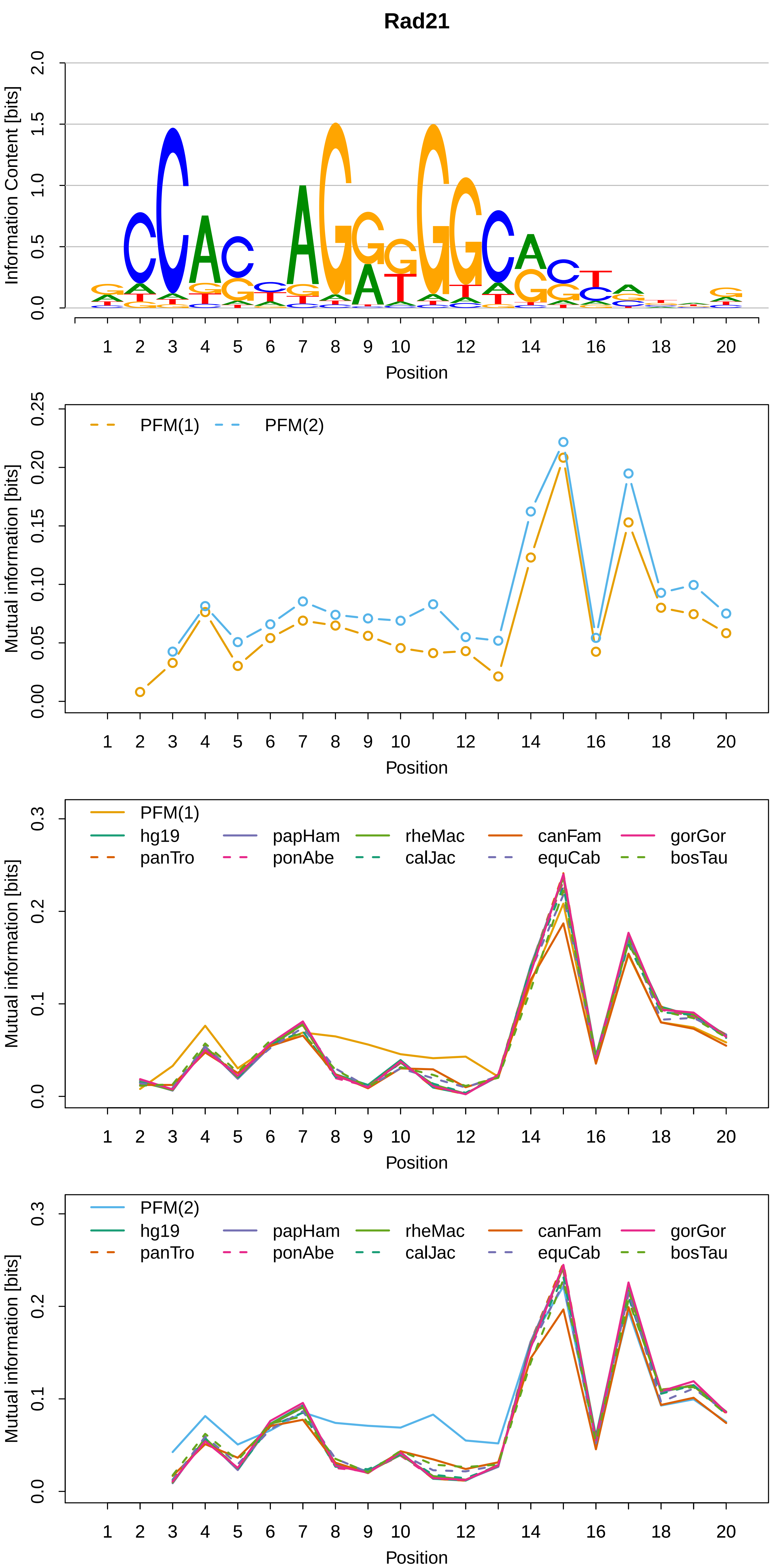

Rad21
Information Content [bits]
Position
Mutual information [bits]
PFM(1)
PFM(2)
hg19
panTro
papHam
ponAbe
rheMac
calJac
canFam
equCab
gorGor
bosTau

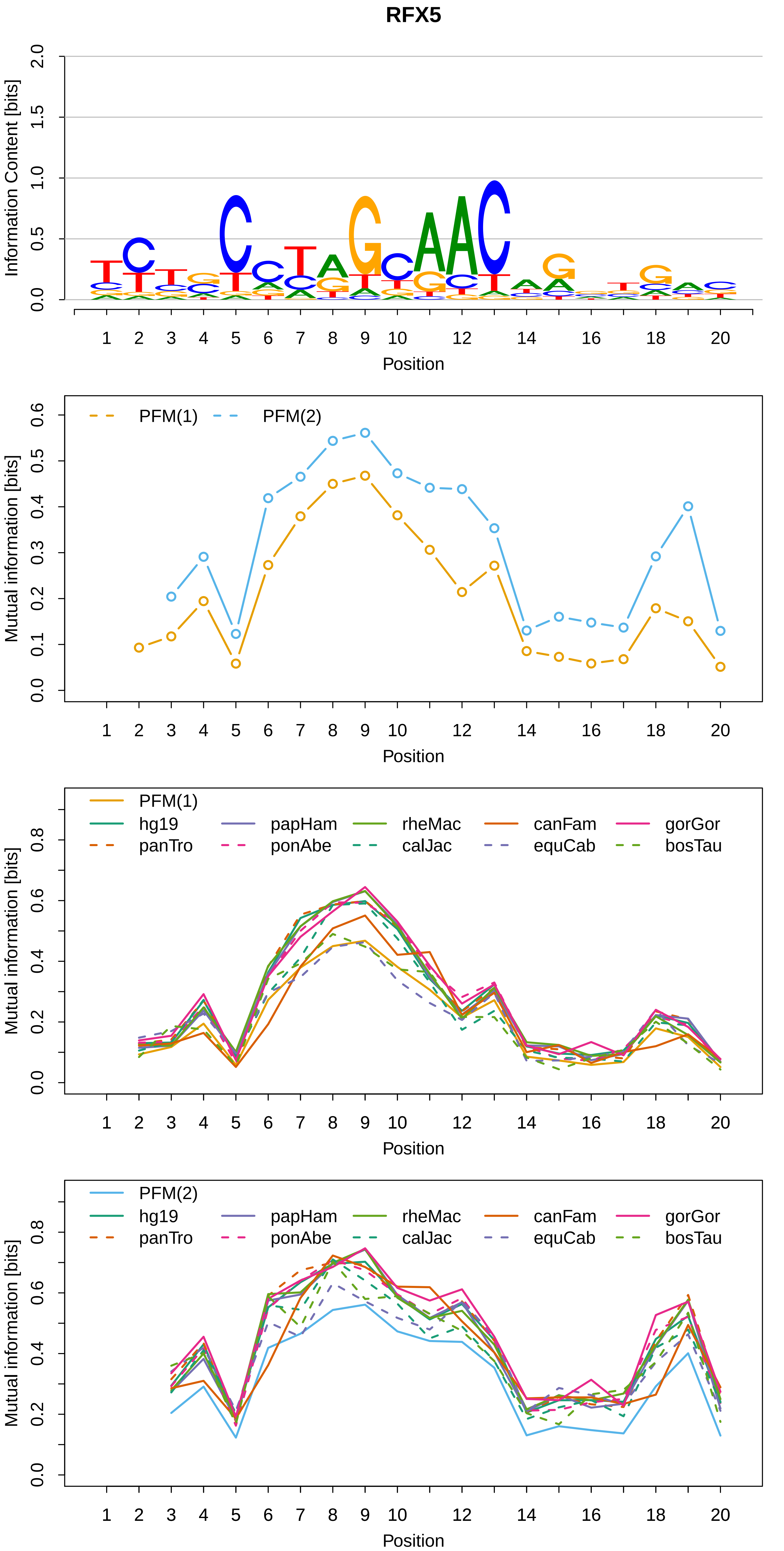
RFX5
Information Content [bits]
Position
Mutual information [bits]
PFM(1)
PFM(2)
hg19
panTro
papHam
ponAbe
rheMac
calJac
canFam
equCab
gorGor
bosTau

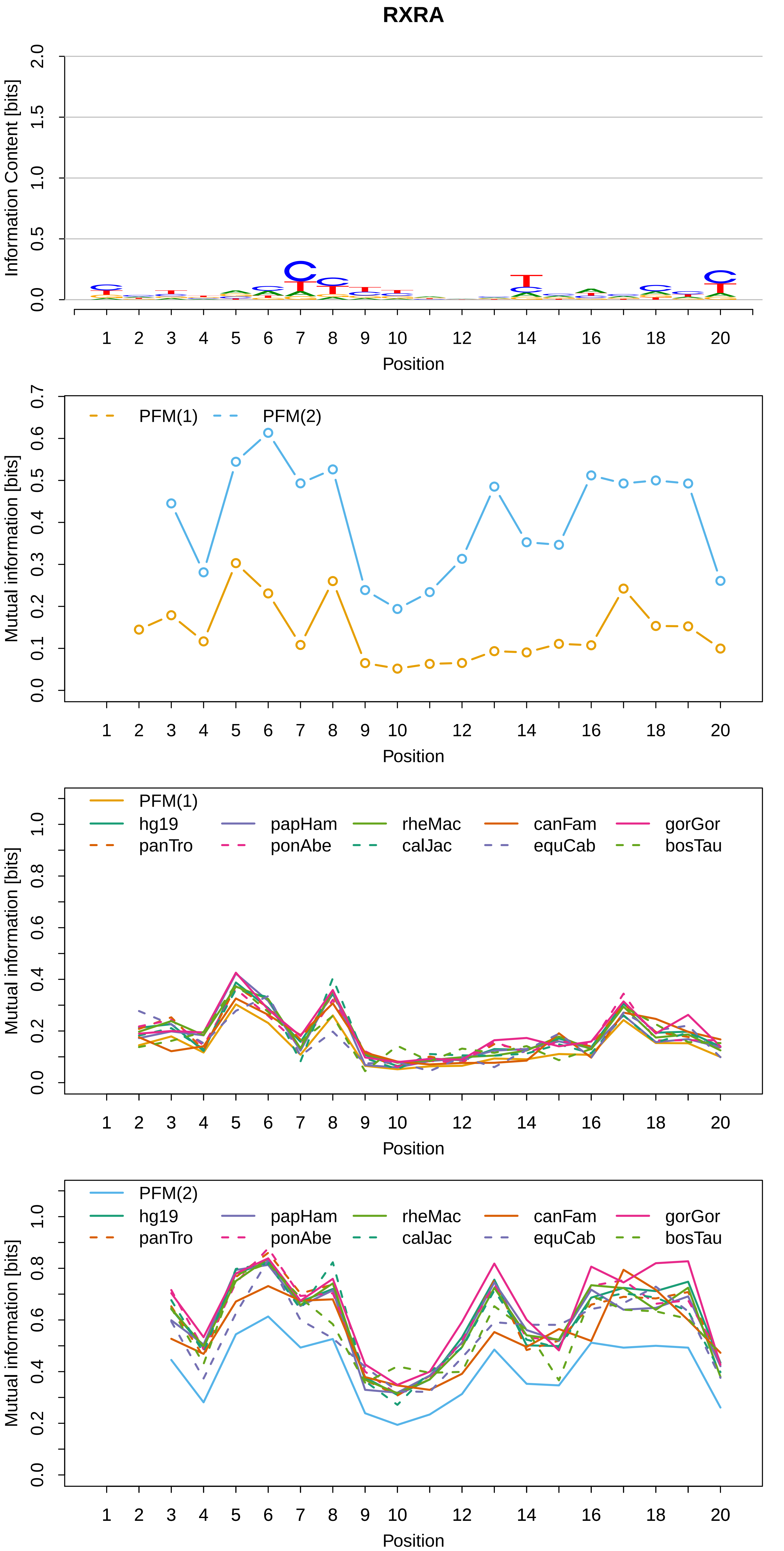

RXRA
Information Content [bits]
Position
Mutual information [bits]
PFM(1)
PFM(2)
hg19
panTro
papHam
ponAbe
rheMac
calJac
canFam
equCab
gorGor
bosTau

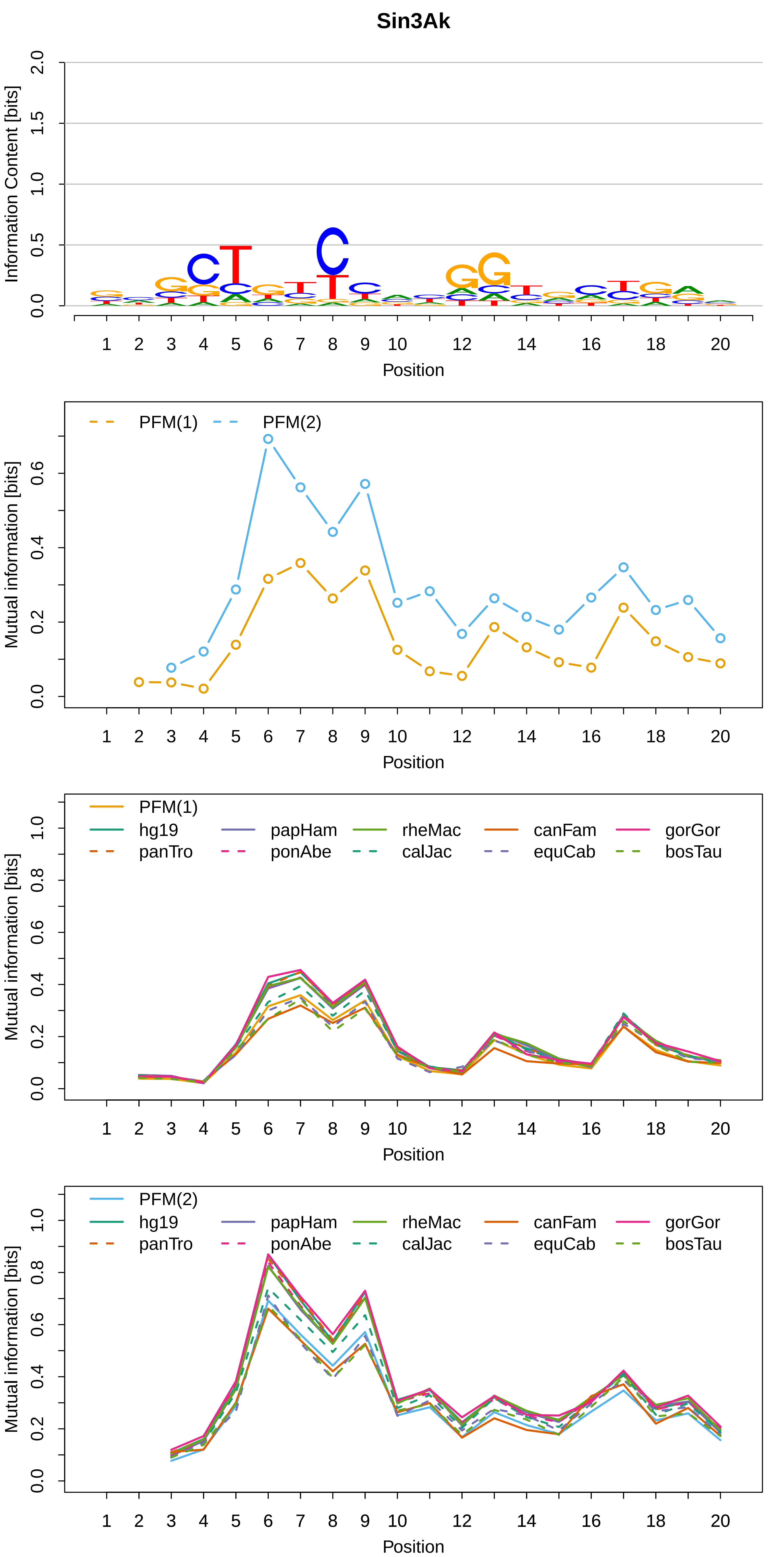

Sin3Ak
Information Content [bits]
Position
Mutual information [bits]
PFM(1)
PFM(2)
hg19
panTro
papHam
ponAbe
rheMac
calJac
canFam
equCab
gorGor
bosTau

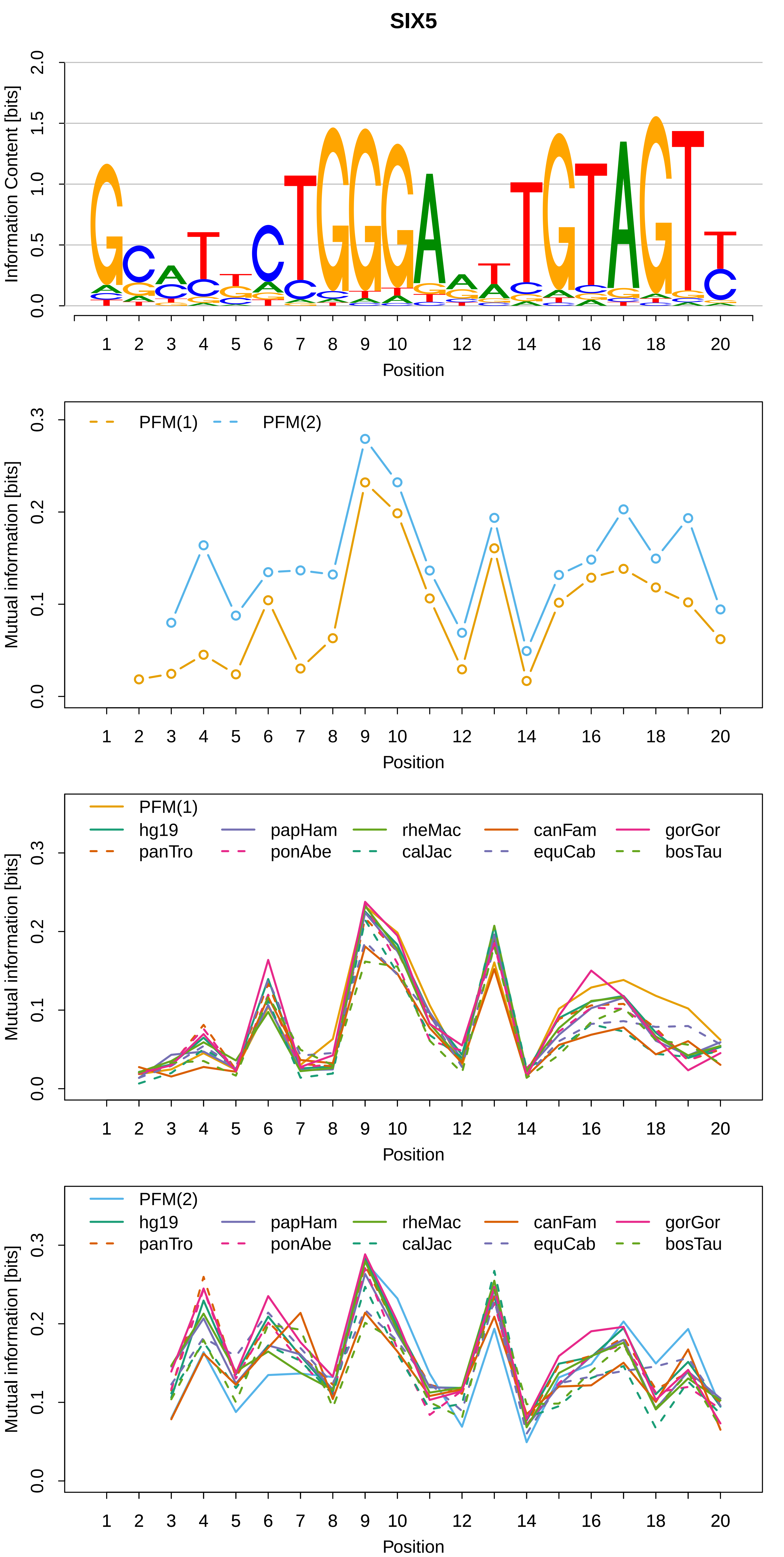
SIX5
Information Content [bits]
Position
Mutual information [bits]
PFM(1)
PFM(2)
hg19
panTro
papHam
ponAbe
rheMac
calJac
canFam
equCab
gorGor
bosTau

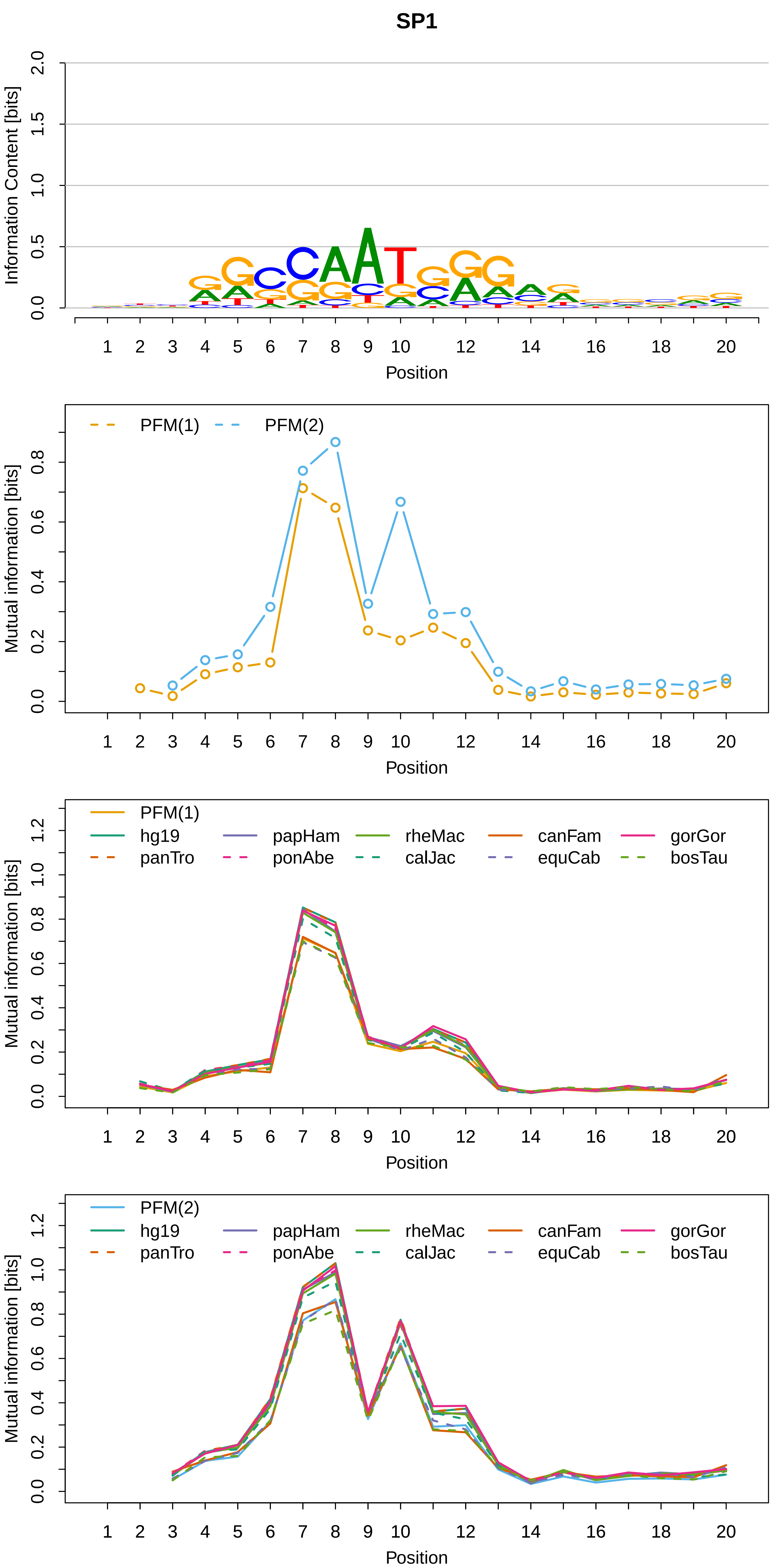

SP1
Information Content [bits]
Position
PFM(1)
PFM(2)
Mutual information [bits]
hg19
panTro
papHam
ponAbe
rheMac
calJac
canFam
equCab
gorGor
bosTau

SP2

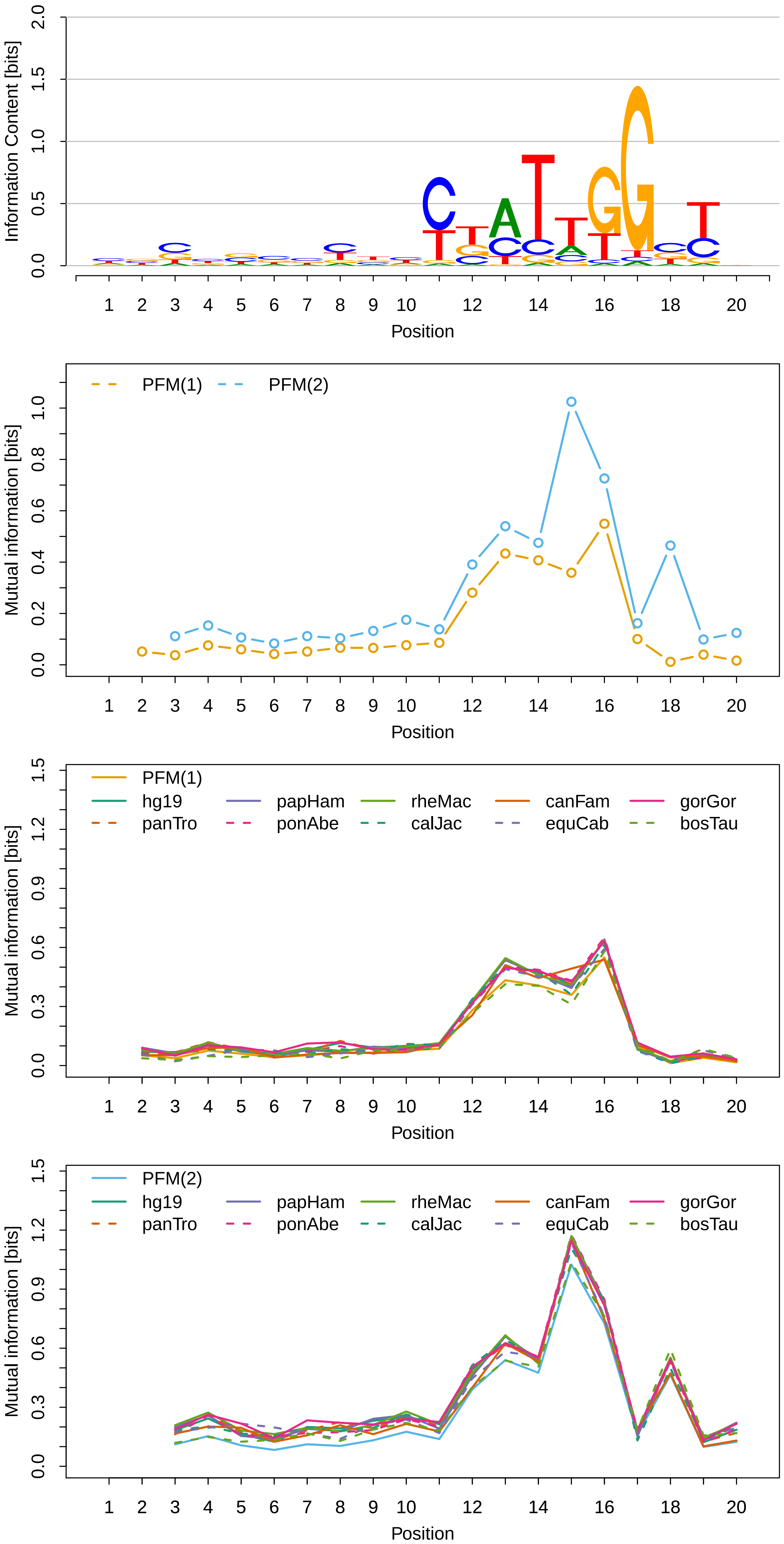
Information Content [bits]
Position
Mutual information [bits]
PFM(1)
PFM(2)
hg19
panTro
papHam
ponAbe
rheMac
calJac
canFam
equCab
gorGor
bosTau

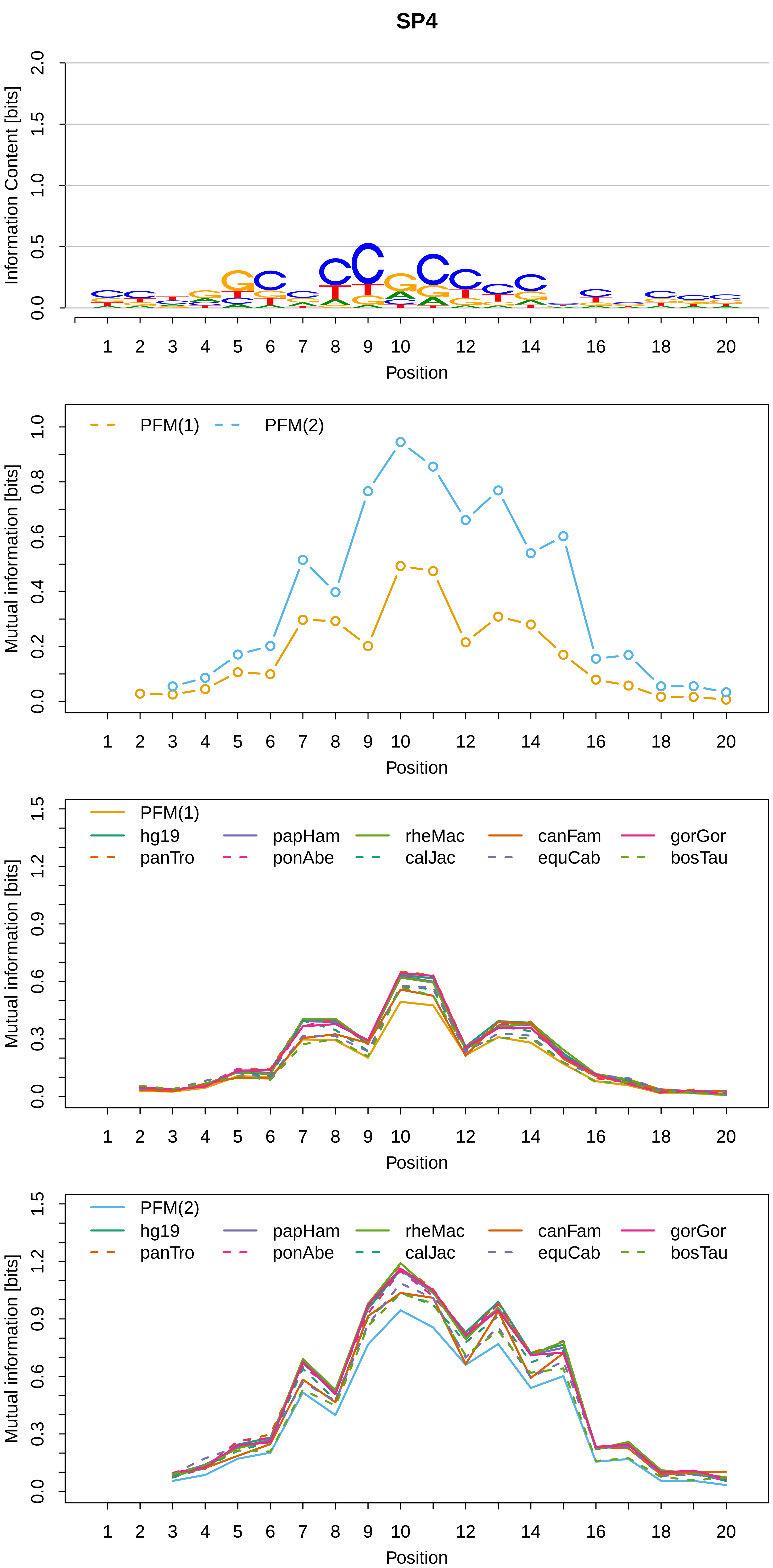
SP4
Information Content [bits]
Position
Mutual information [bits]
PFM(1)
PFM(2)
hg19
panTro
papHam
ponAbe
rheMac
calJac
canFam
equCab
gorGor
bosTau

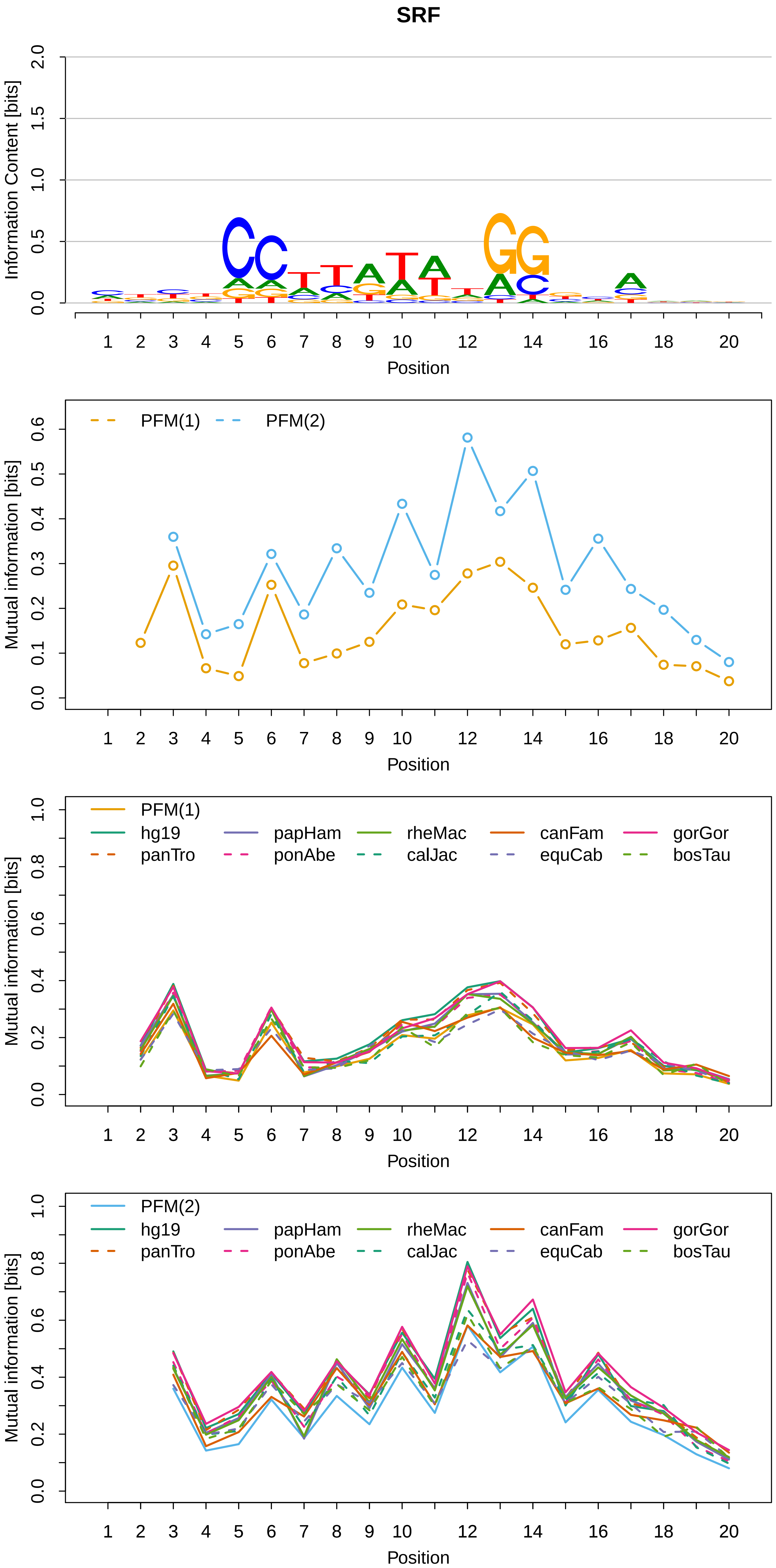

SRF
Information Content [bits]
0.0
0.5
1.0
1.5
2.0
Position
1
2
3
4
5
6
7
8
9
10
12
14
16
18
20
Mutual information [bits]
PFM(1)
PFM(2)
0.0
0.1
0.2
0.3
0.4
0.5
0.6
hg19
panTro
papHam
ponAbe
rheMac
calJac
canFam
equCab
gorGor
bosTau
0.2
0.4
0.6
0.8
1.0

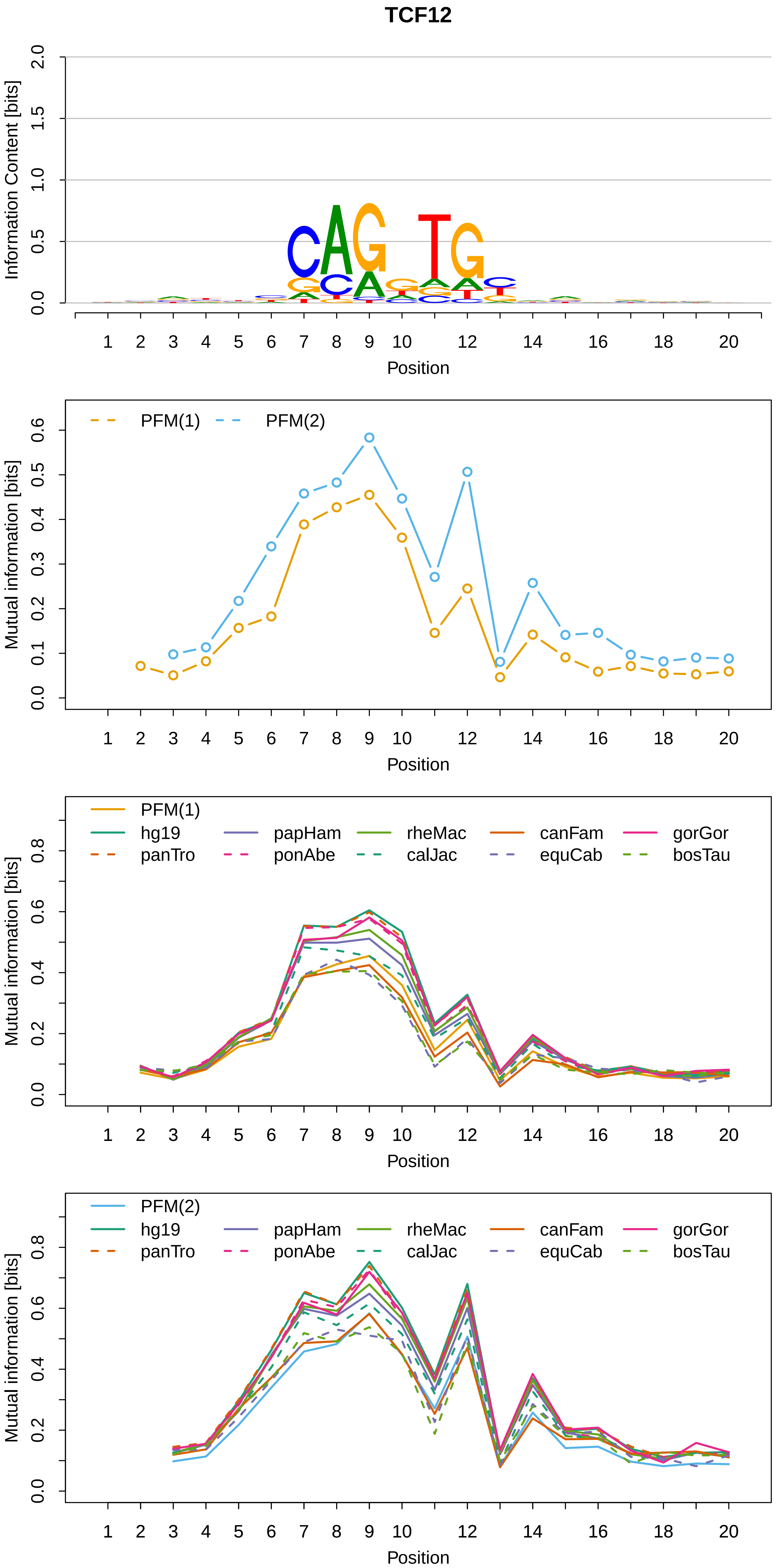

TCF12
Information Content [bits]
Position
PFM(1)
PFM(2)
Mutual information [bits]
hg19
panTro
papHam
ponAbe
rheMac
calJac
canFam
equCab
gorGor
bosTau

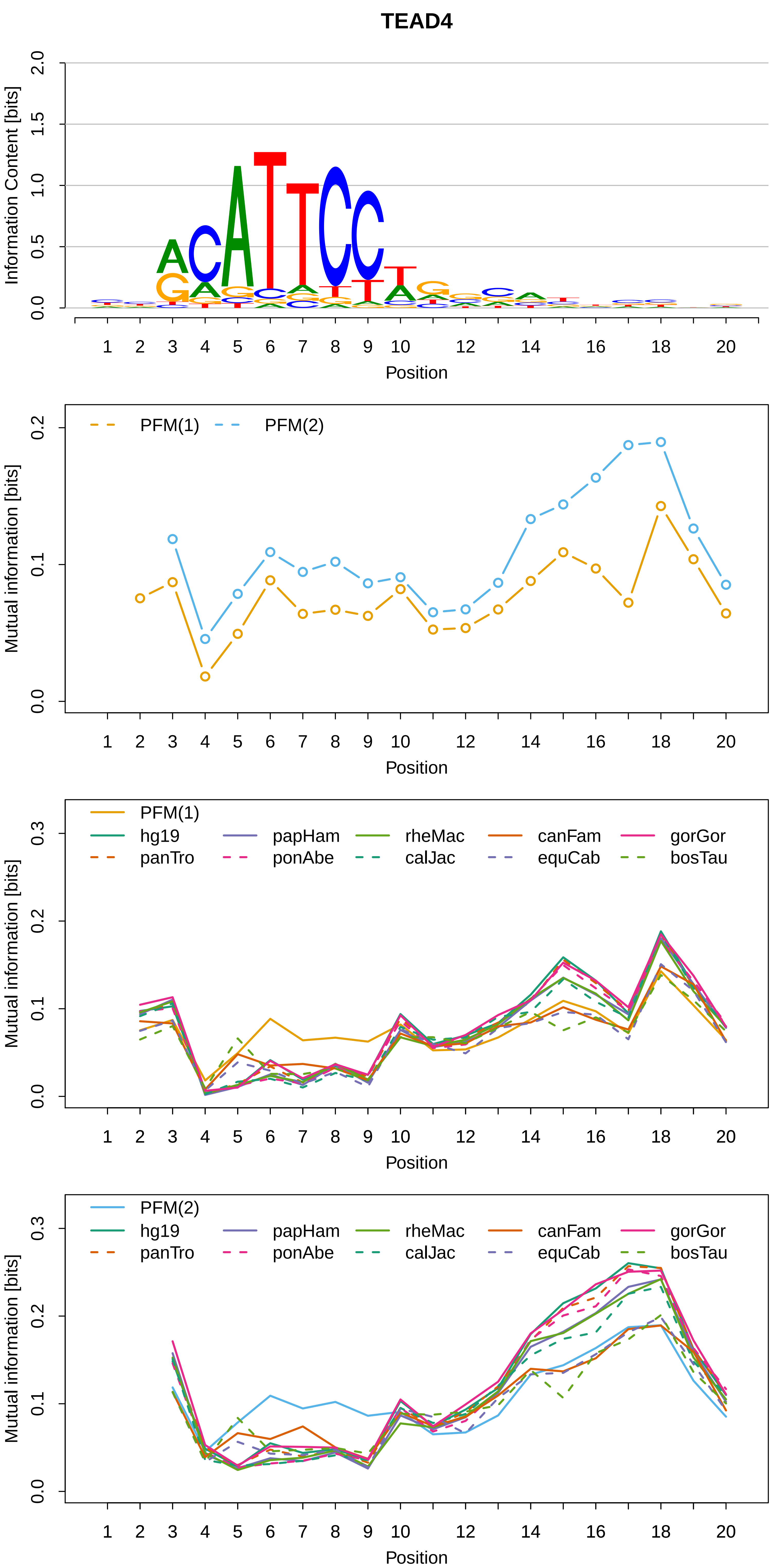

TEAD4
Information Content [bits]
Position
Mutual information [bits]
PFM(1)
PFM(2)
hg19
panTro
papHam
ponAbe
rheMac
calJac
canFam
equCab
gorGor
bosTau

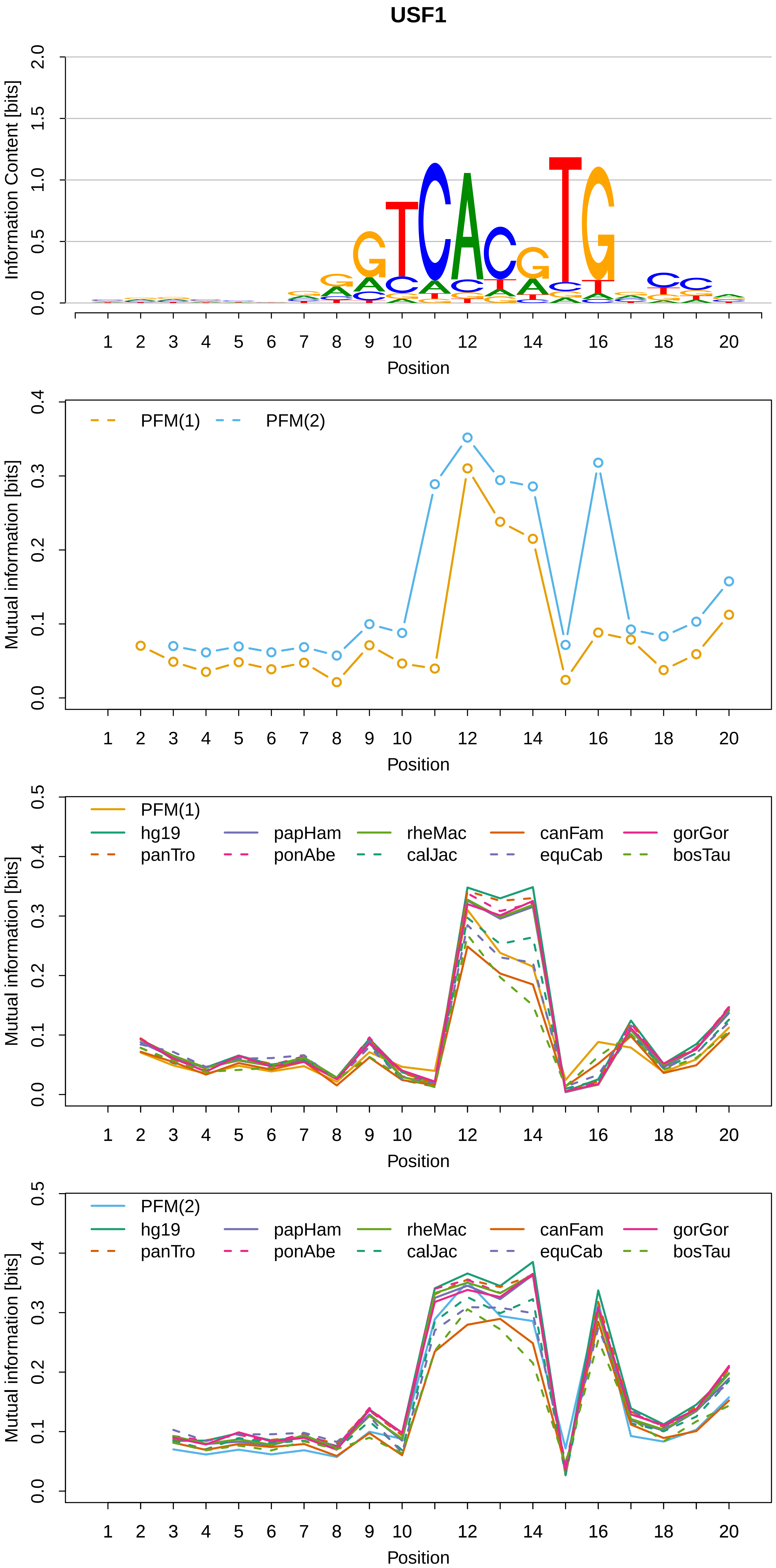

USF1
Information Content [bits]
Position
Mutual information [bits]
PFM(1)
PFM(2)
hg19
panTro
papHam
ponAbe
rheMac
calJac
canFam
equCab
gorGor
bosTau

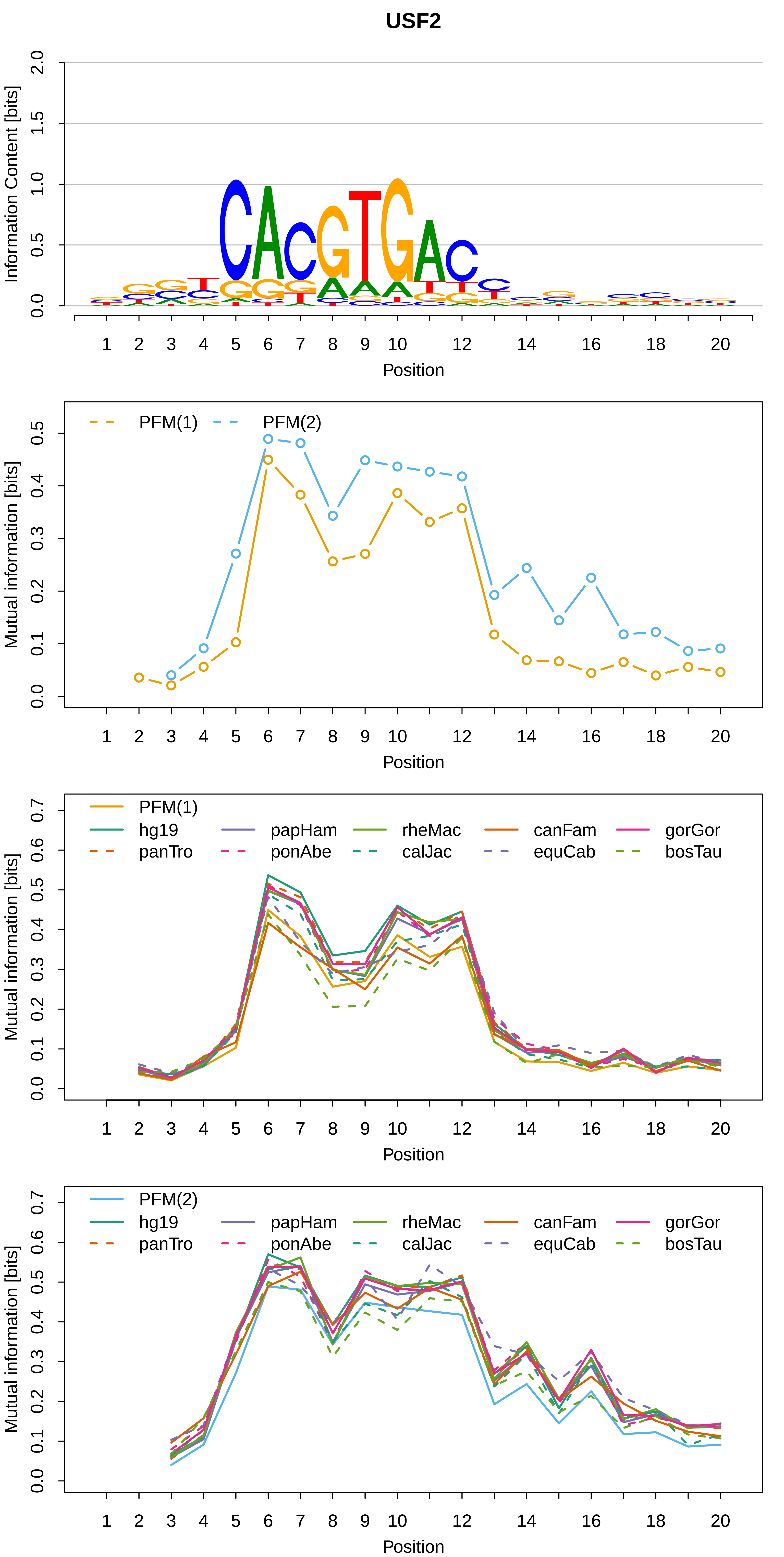

USF2
Information Content [bits]
Position
PFM(1)
PFM(2)
Mutual information [bits]
hg19
panTro
papHam
ponAbe
rheMac
calJac
canFam
equCab
gorGor
bosTau

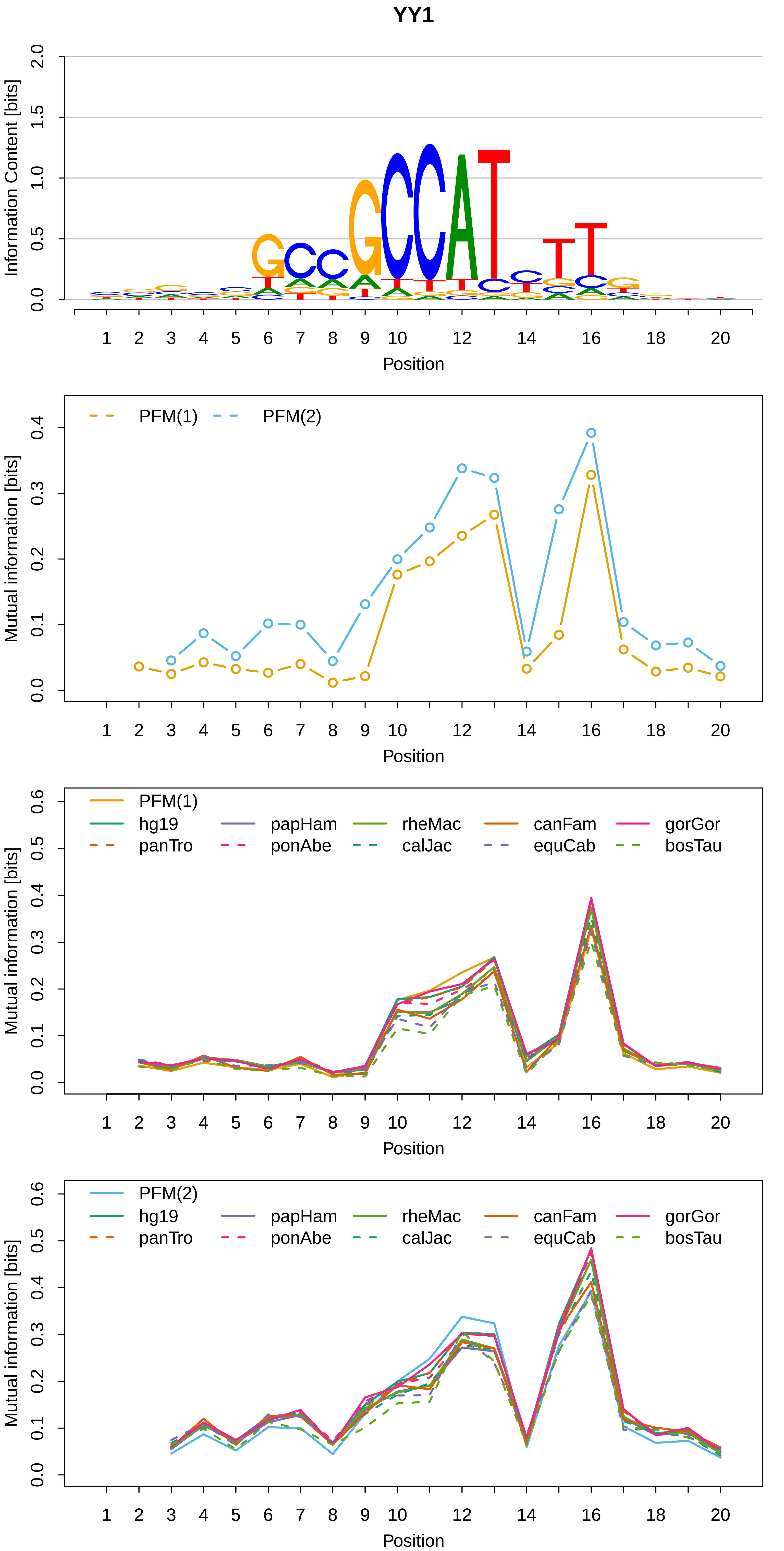
YY1
Information Content [bits]
Position
Mutual information [bits]
PFM(1)
PFM(2)
hg19
panTro
papHam
ponAbe
rheMac
calJac
canFam
equCab
gorGor
bosTau

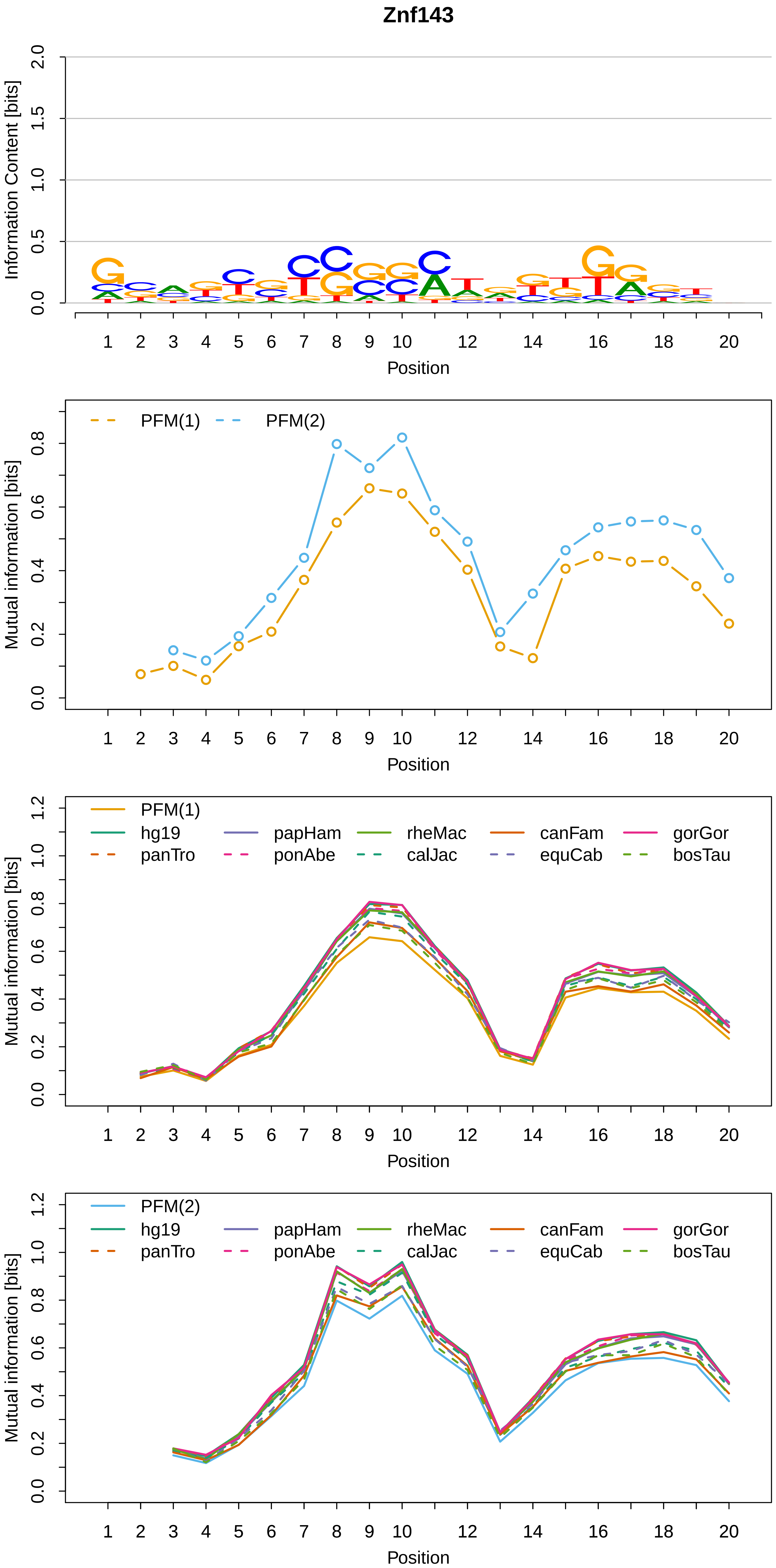

Znf143
Information Content [bits]
0.0
0.5
1.0
1.5
2.0
1 2 3 4 5 6 7 8 9 10 12 14 16 18 20
Position
PFM(1)
PFM(2)
Mutual information [bits]
0.0 0.2 0.4 0.6 0.8
1 2 3 4 5 6 7 8 9 10 12 14 16 18 20
Position
PFM(1)
hg19
papHam
rheMac
canFam
gorGor
panTro
ponAbe
calJac
equCab
bosTau
Mutual information [bits]
0.0 0.2 0.4 0.6 0.8 1.0 1.2
1 2 3 4 5 6 7 8 9 10 12 14 16 18 20
Position
PFM(2)
hg19
papHam
rheMac
canFam
gorGor
panTro
ponAbe
calJac
equCab
bosTau
Mutual information [bits]
0.0 0.2 0.4 0.6 0.8 1.0 1.2
1 2 3 4 5 6 7 8 9 10 12 14 16 18 20
Position
