# Supplementary figures and images for: Combining phylogenetic footprinting with motif models incorporating intra-motif dependencies
Source: BMC Bioinformatics. 2017 Mar 1;18:141. doi: 10.1186/s12859-017-1495-1 (PMC5333389; doi:10.1186/s12859-017-1495-1)

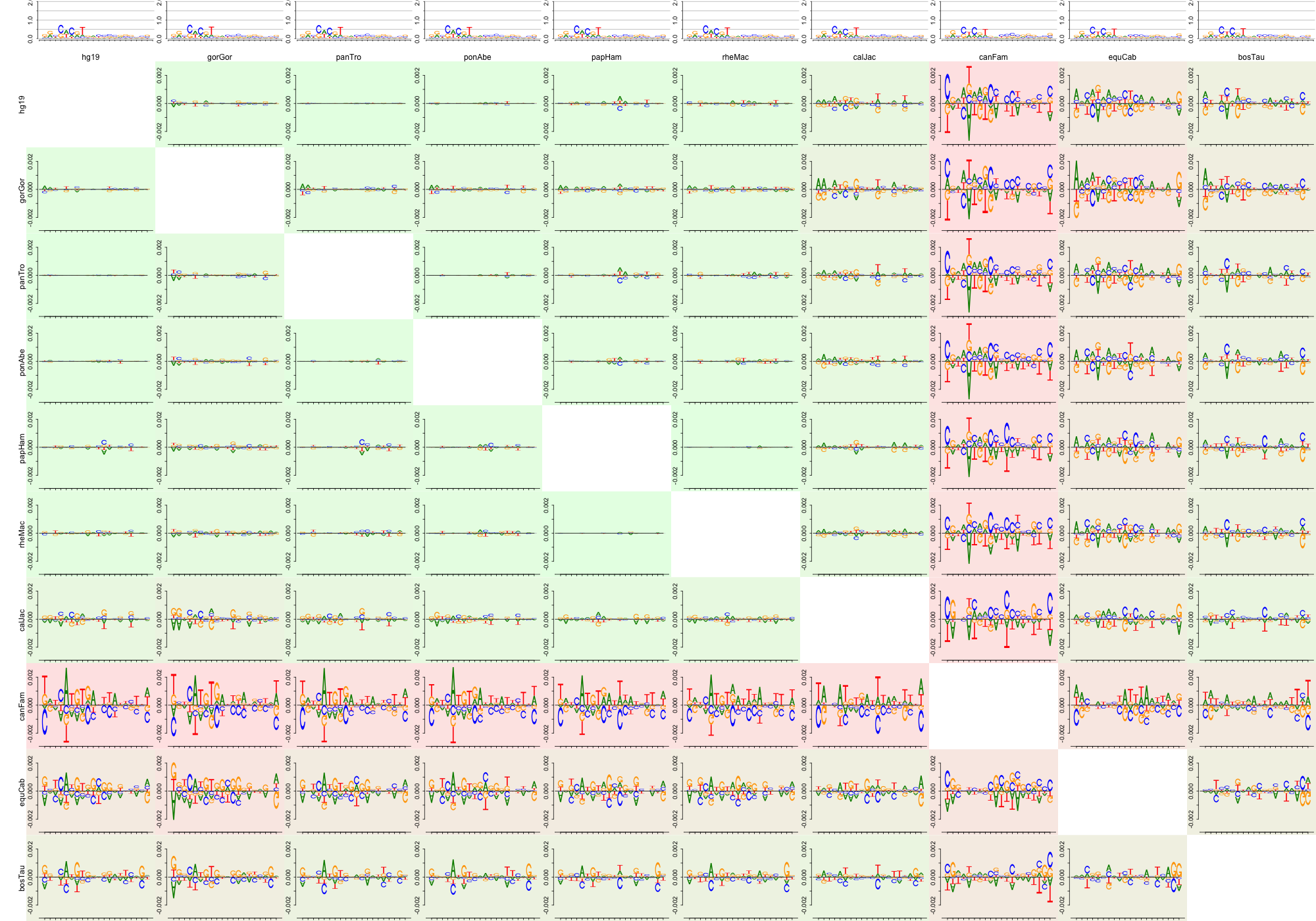

Supplement: Supplementary file 4 — Tables of difference logos. The file contains for each of the 35 TFs a 10×10 table of difference logos for a pair-wise visual comparison of species-specific motifs. (ZIP 26112 kb) [file 12859_2017_1495_MOESM4_ESM.zip › ATF3.png]

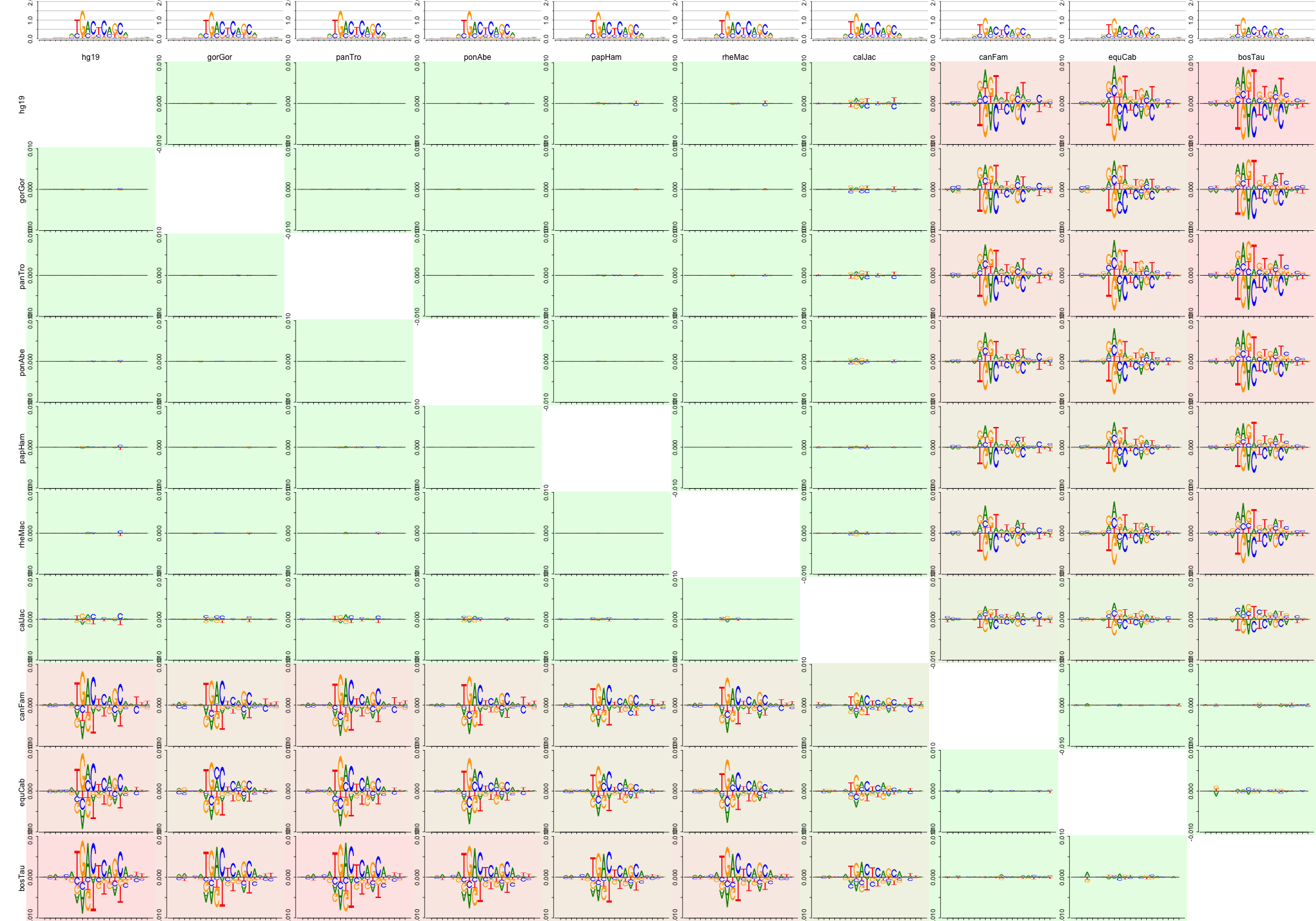

Supplement: Supplementary file 4 — Tables of difference logos. The file contains for each of the 35 TFs a 10×10 table of difference logos for a pair-wise visual comparison of species-specific motifs. (ZIP 26112 kb) [file 12859_2017_1495_MOESM4_ESM.zip › Bach1.png]

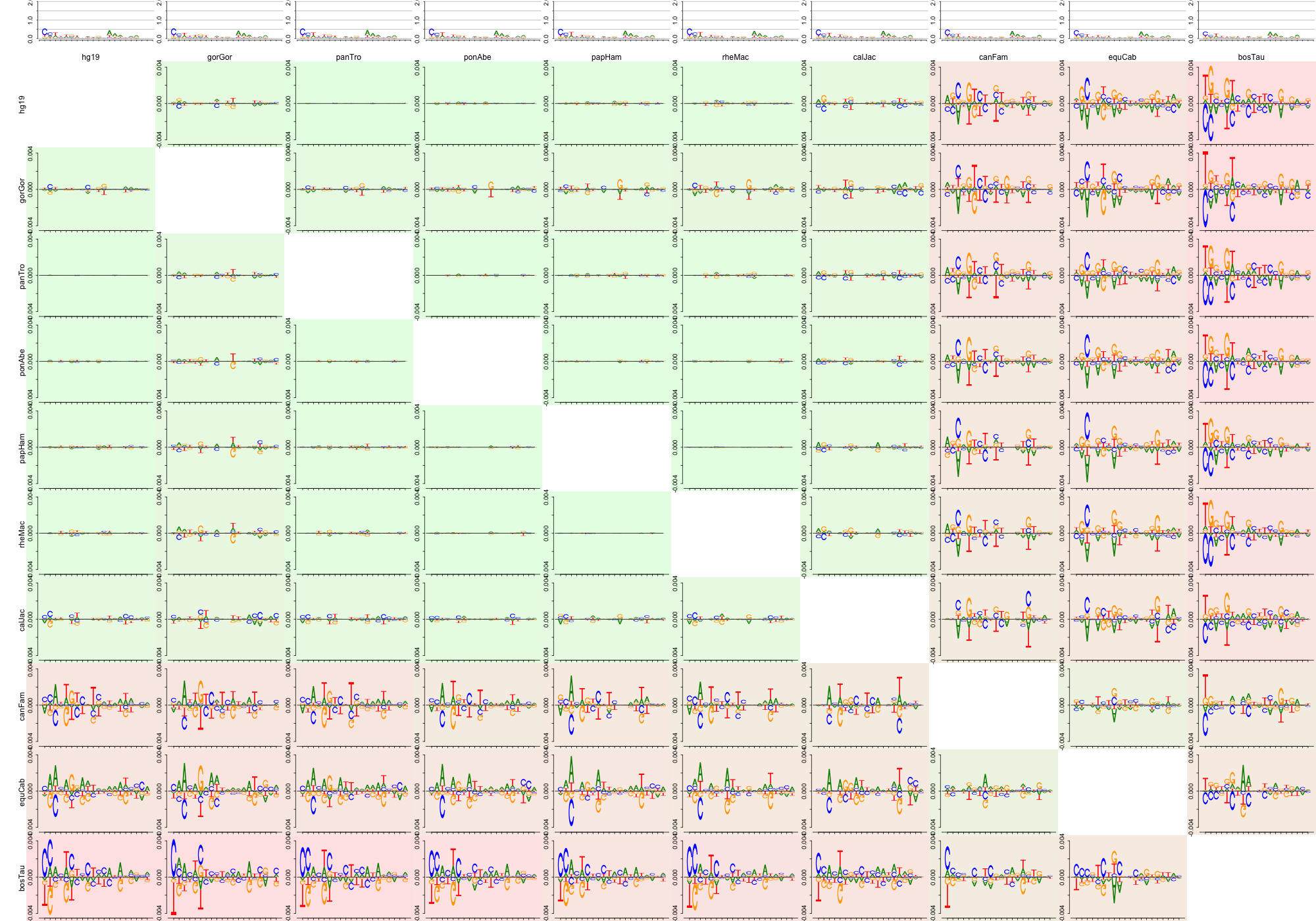

Supplement: Supplementary file 4 — Tables of difference logos. The file contains for each of the 35 TFs a 10×10 table of difference logos for a pair-wise visual comparison of species-specific motifs. (ZIP 26112 kb) [file 12859_2017_1495_MOESM4_ESM.zip › BCL11A.png]

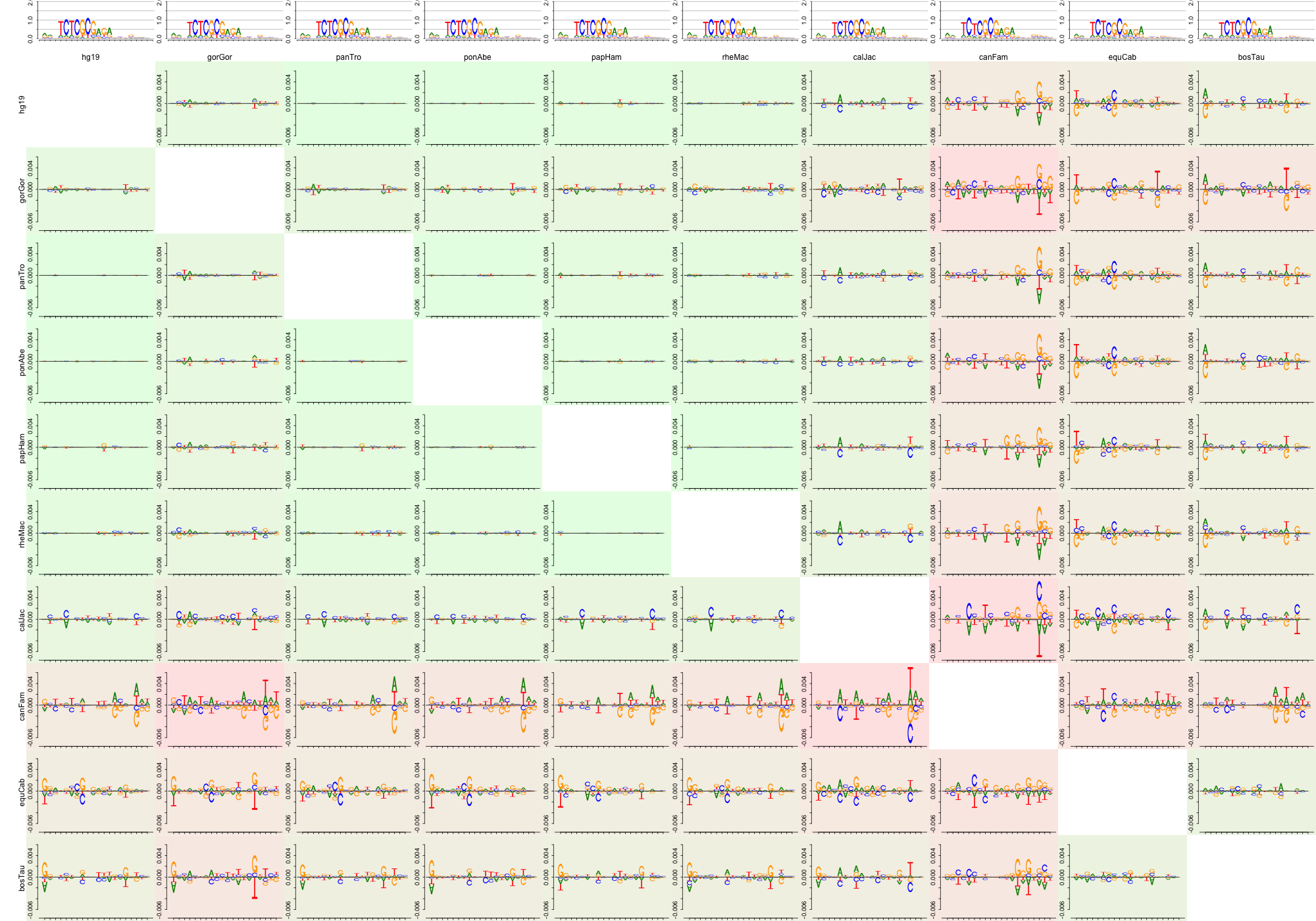

Supplement: Supplementary file 4 — Tables of difference logos. The file contains for each of the 35 TFs a 10×10 table of difference logos for a pair-wise visual comparison of species-specific motifs. (ZIP 26112 kb) [file 12859_2017_1495_MOESM4_ESM.zip › BRCA1.png]

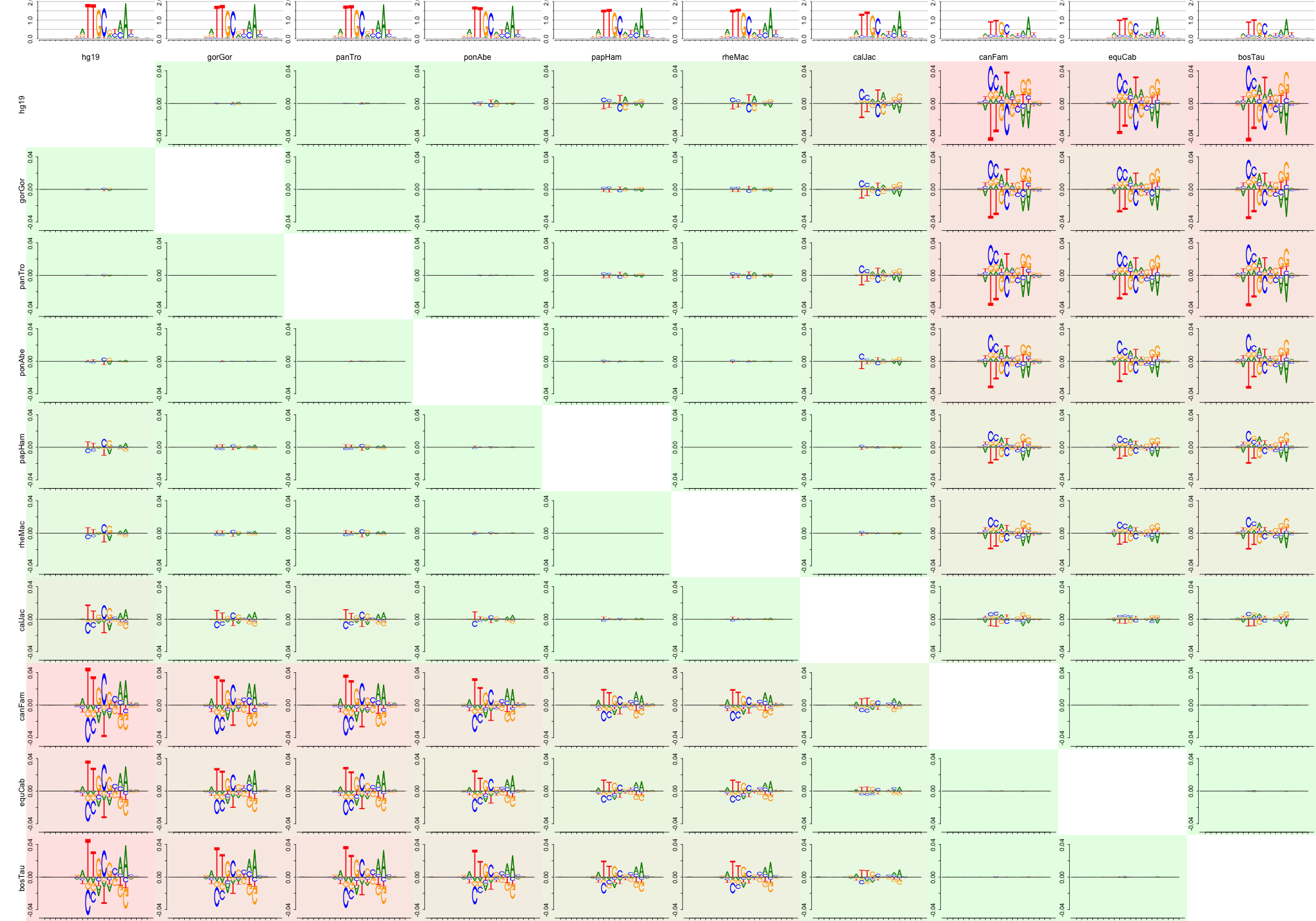

Supplement: Supplementary file 4 — Tables of difference logos. The file contains for each of the 35 TFs a 10×10 table of difference logos for a pair-wise visual comparison of species-specific motifs. (ZIP 26112 kb) [file 12859_2017_1495_MOESM4_ESM.zip › CEBPB.png]

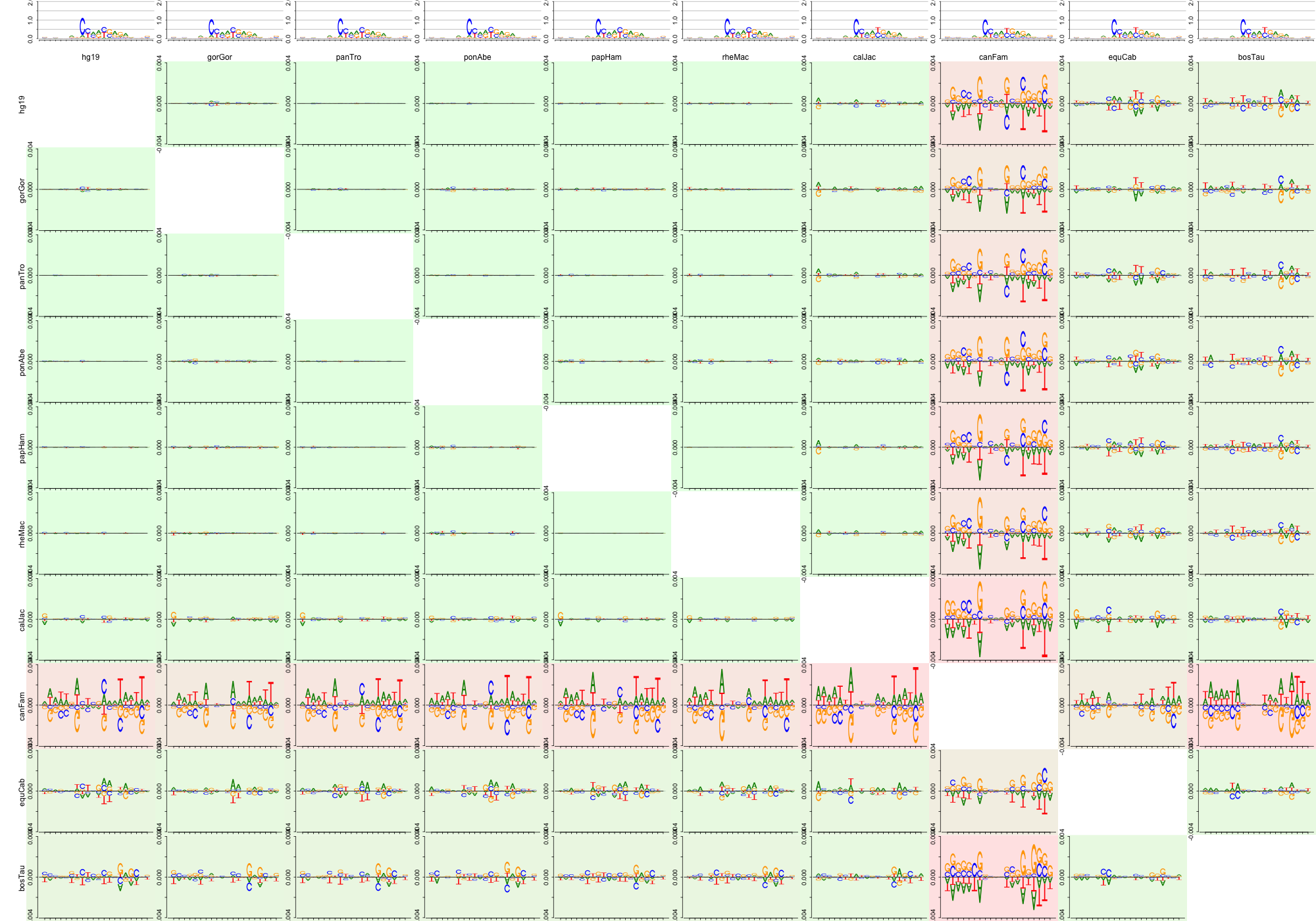

Supplement: Supplementary file 4 — Tables of difference logos. The file contains for each of the 35 TFs a 10×10 table of difference logos for a pair-wise visual comparison of species-specific motifs. (ZIP 26112 kb) [file 12859_2017_1495_MOESM4_ESM.zip › CHD2.png]

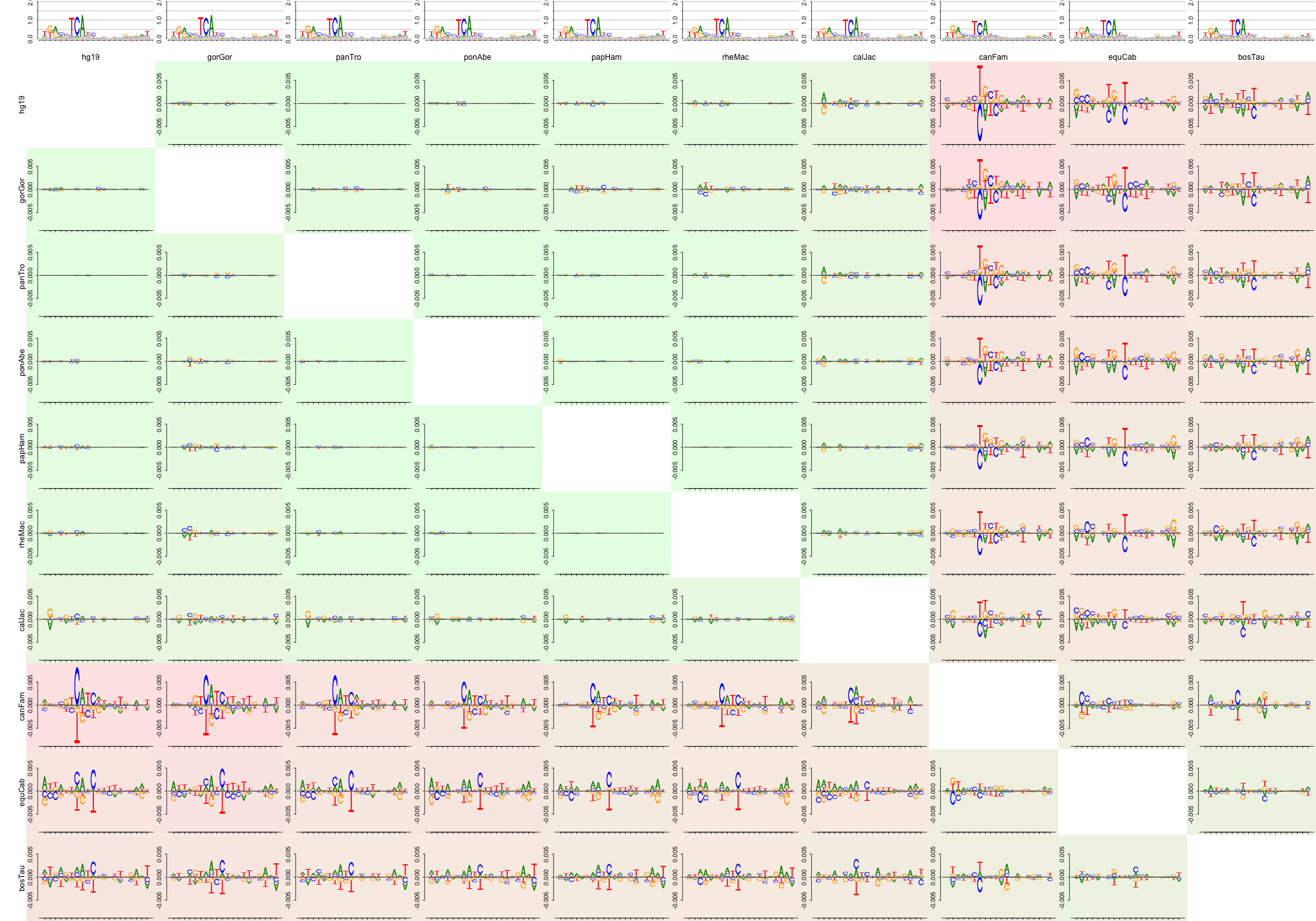

Supplement: Supplementary file 4 — Tables of difference logos. The file contains for each of the 35 TFs a 10×10 table of difference logos for a pair-wise visual comparison of species-specific motifs. (ZIP 26112 kb) [file 12859_2017_1495_MOESM4_ESM.zip › CJUN.png]

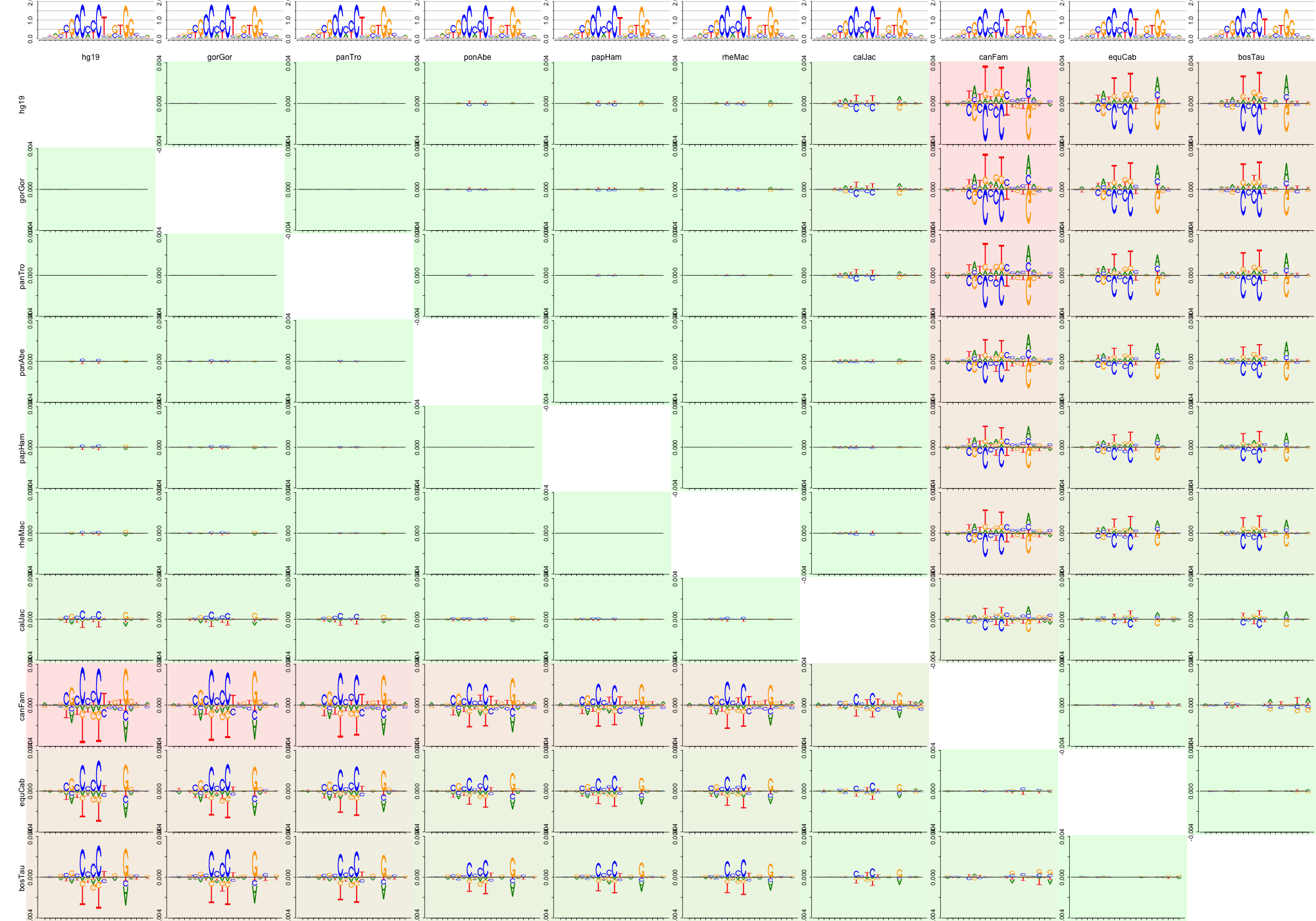

Supplement: Supplementary file 4 — Tables of difference logos. The file contains for each of the 35 TFs a 10×10 table of difference logos for a pair-wise visual comparison of species-specific motifs. (ZIP 26112 kb) [file 12859_2017_1495_MOESM4_ESM.zip › CTCF.png]

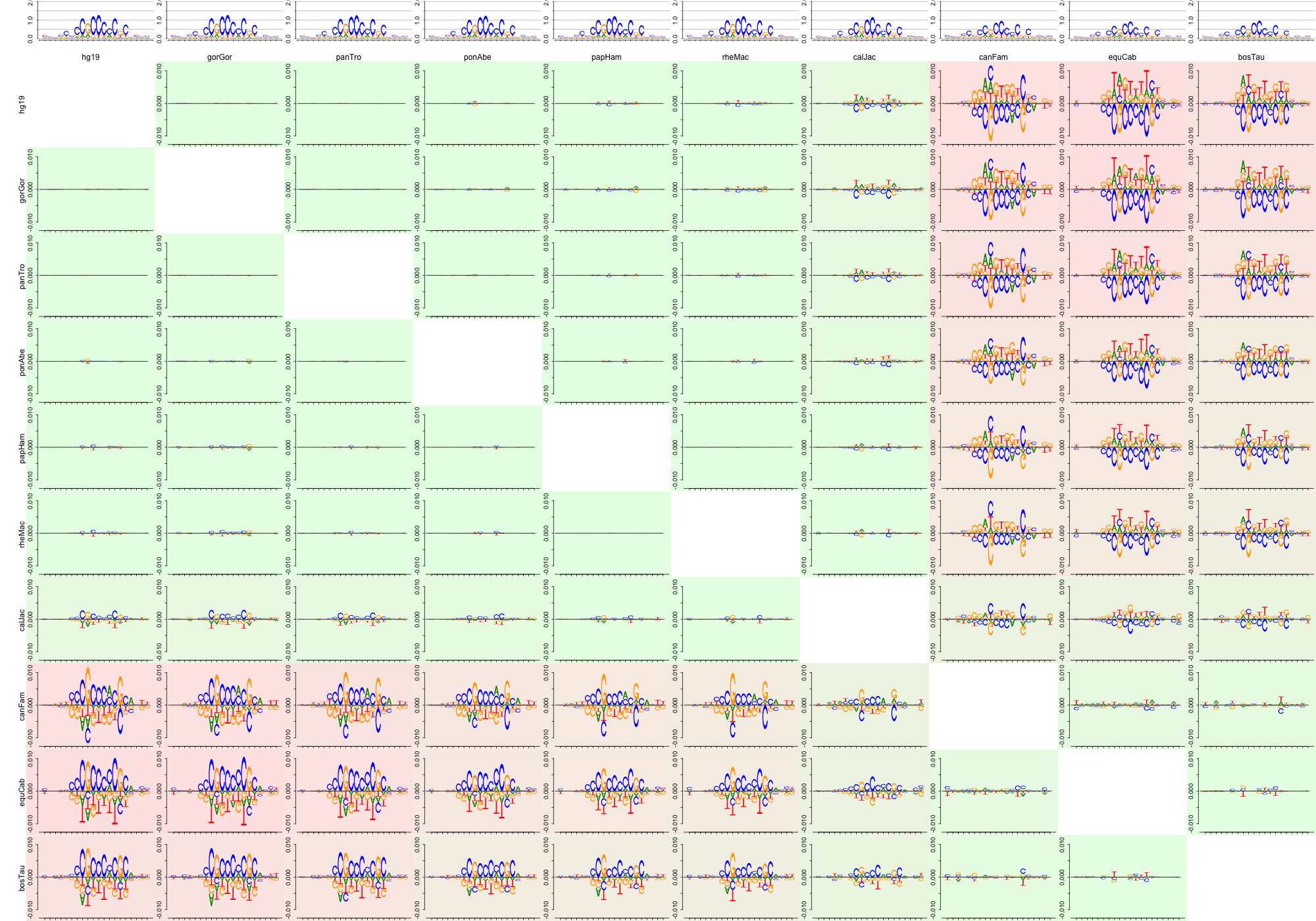

Supplement: Supplementary file 4 — Tables of difference logos. The file contains for each of the 35 TFs a 10×10 table of difference logos for a pair-wise visual comparison of species-specific motifs. (ZIP 26112 kb) [file 12859_2017_1495_MOESM4_ESM.zip › EGR1.png]

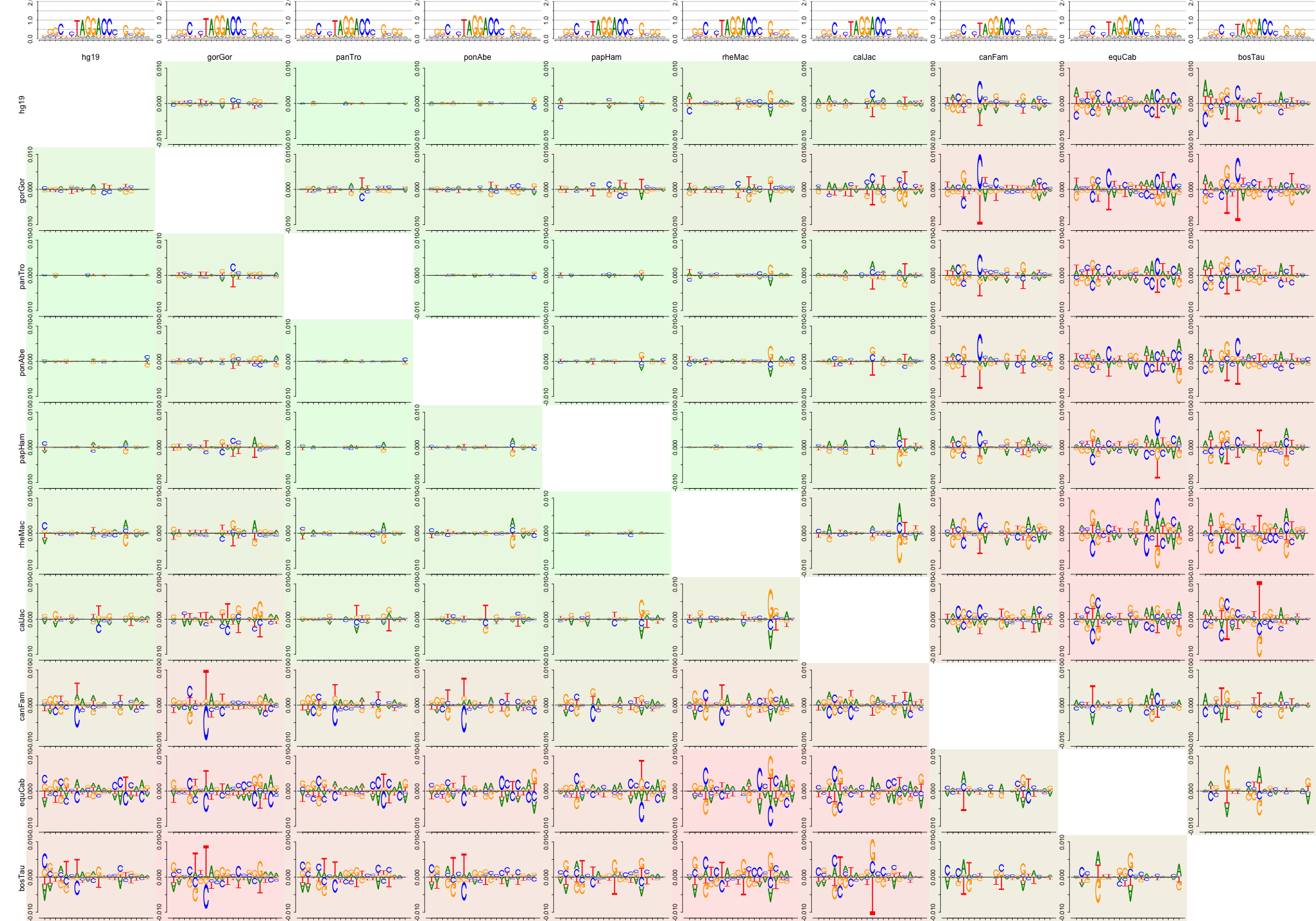

Supplement: Supplementary file 4 — Tables of difference logos. The file contains for each of the 35 TFs a 10×10 table of difference logos for a pair-wise visual comparison of species-specific motifs. (ZIP 26112 kb) [file 12859_2017_1495_MOESM4_ESM.zip › FOSL1.png]

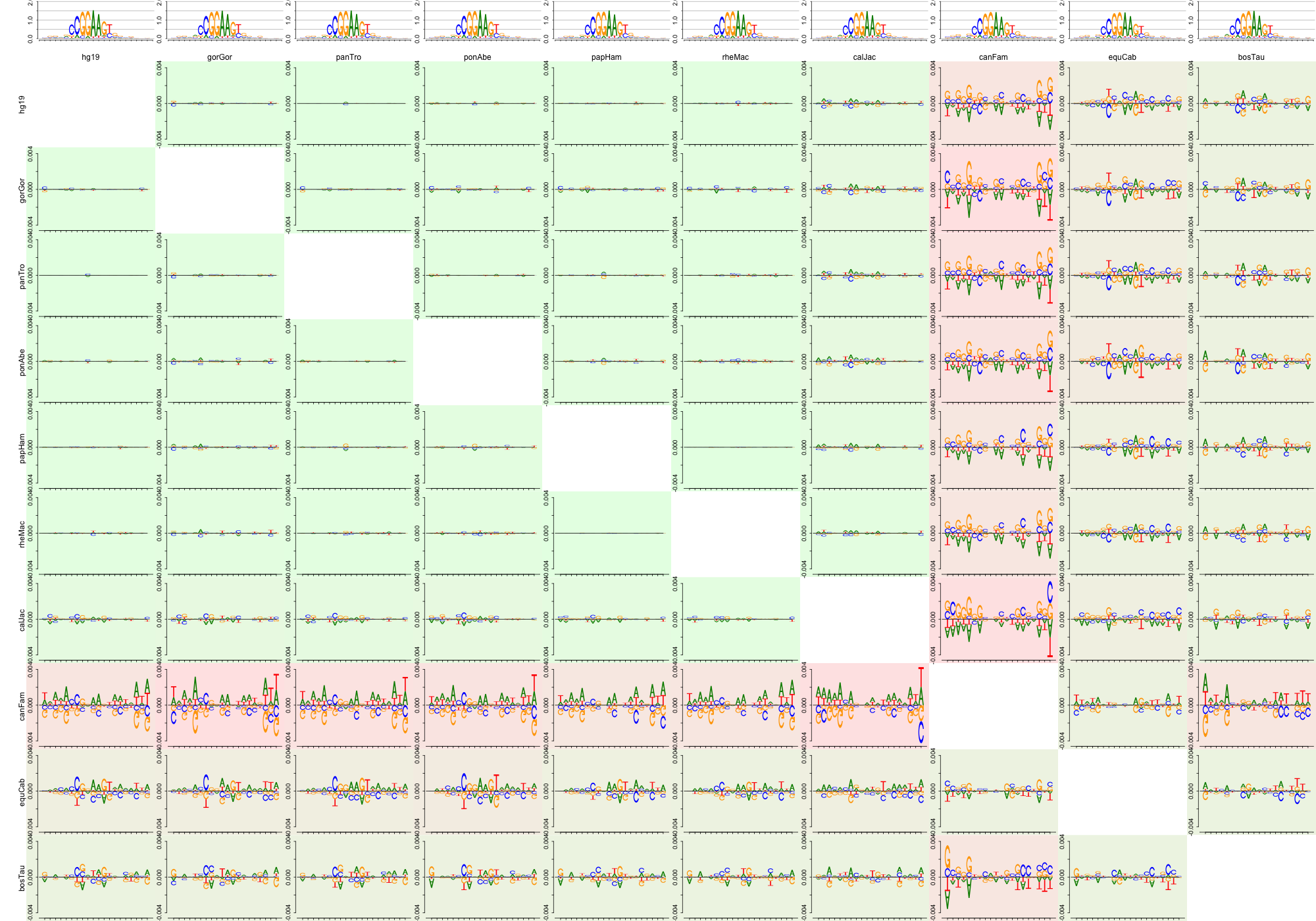

Supplement: Supplementary file 4 — Tables of difference logos. The file contains for each of the 35 TFs a 10×10 table of difference logos for a pair-wise visual comparison of species-specific motifs. (ZIP 26112 kb) [file 12859_2017_1495_MOESM4_ESM.zip › GABP.png]

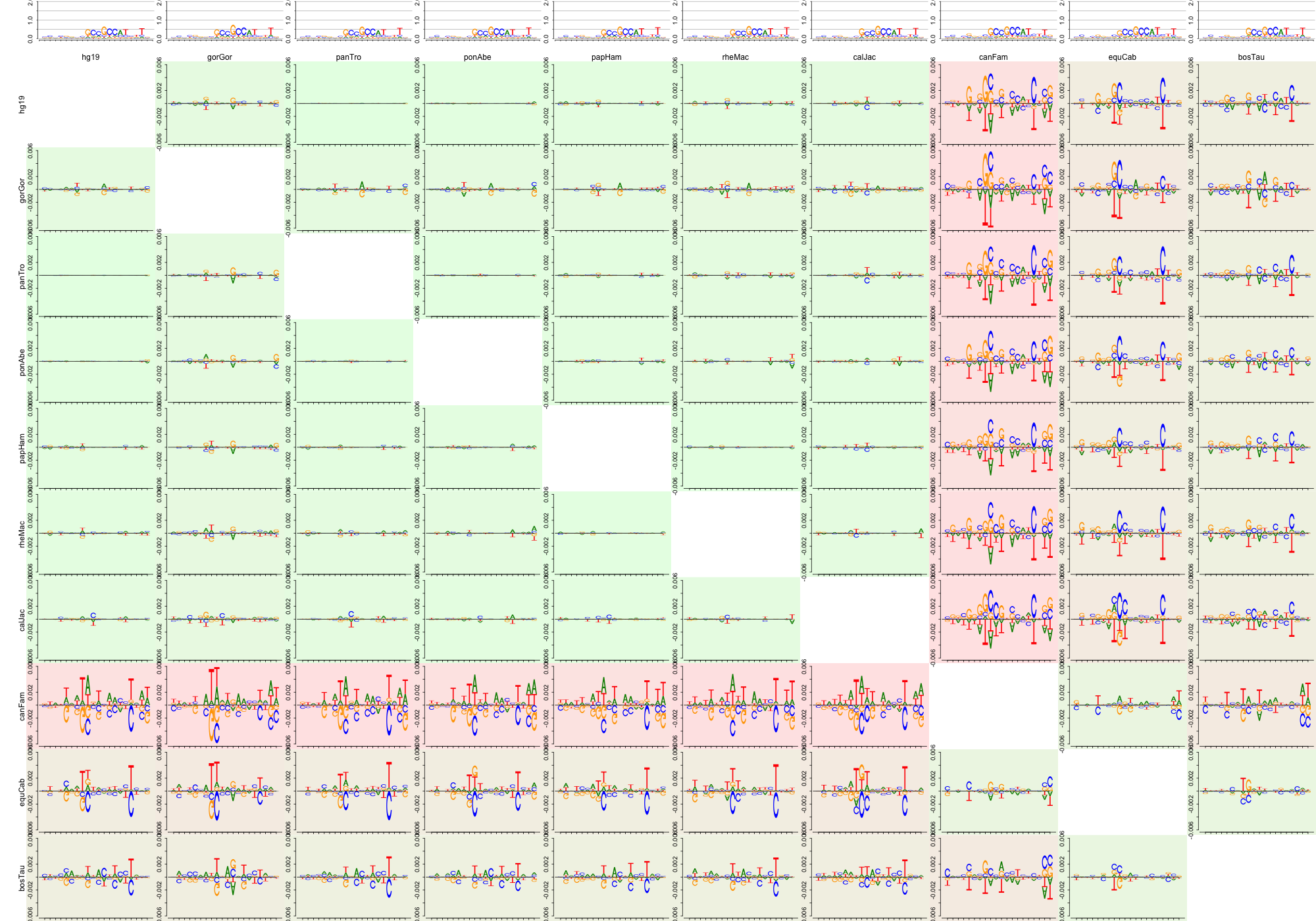

Supplement: Supplementary file 4 — Tables of difference logos. The file contains for each of the 35 TFs a 10×10 table of difference logos for a pair-wise visual comparison of species-specific motifs. (ZIP 26112 kb) [file 12859_2017_1495_MOESM4_ESM.zip › JARIDA1A.png]

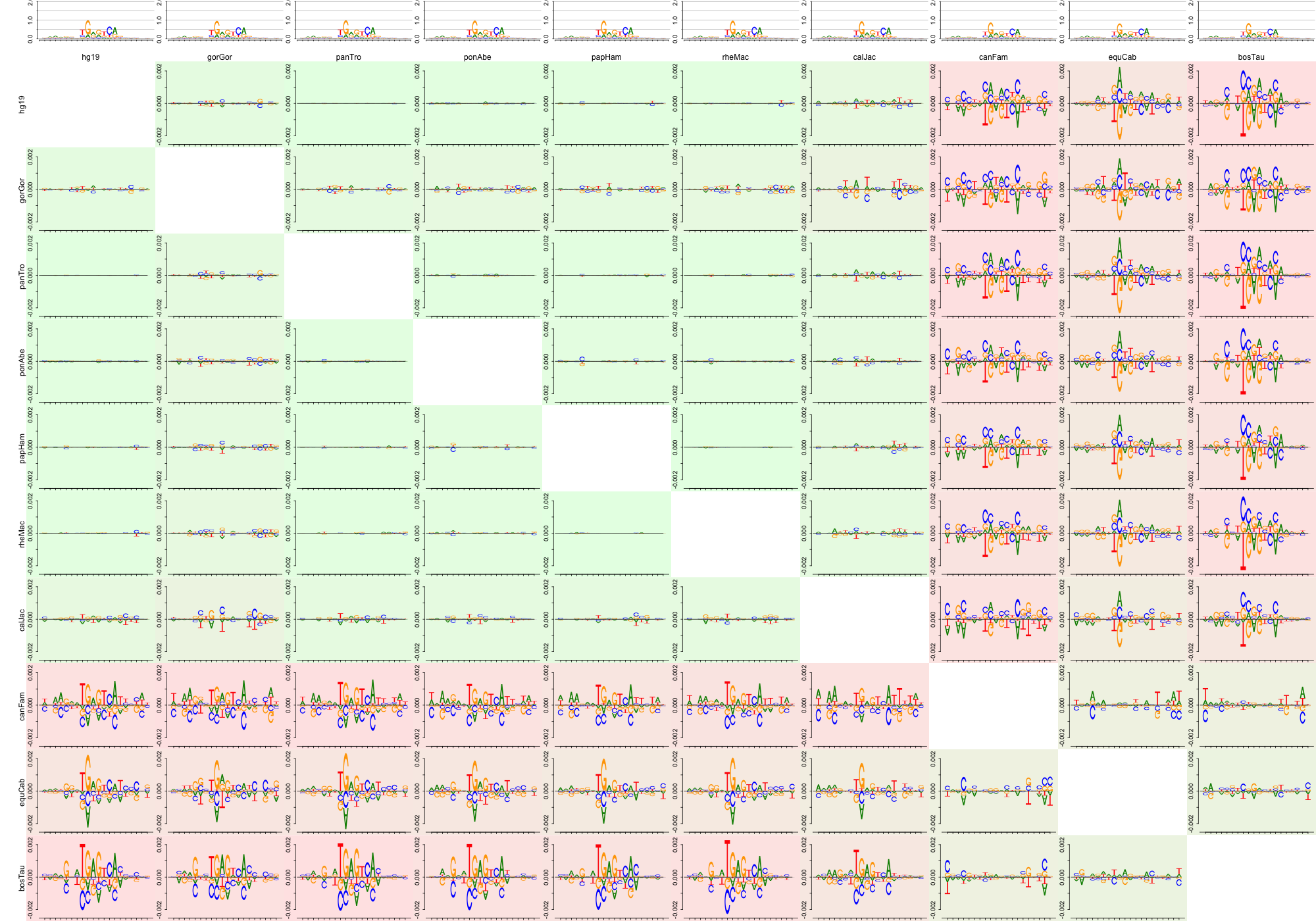

Supplement: Supplementary file 4 — Tables of difference logos. The file contains for each of the 35 TFs a 10×10 table of difference logos for a pair-wise visual comparison of species-specific motifs. (ZIP 26112 kb) [file 12859_2017_1495_MOESM4_ESM.zip › JunD.png]

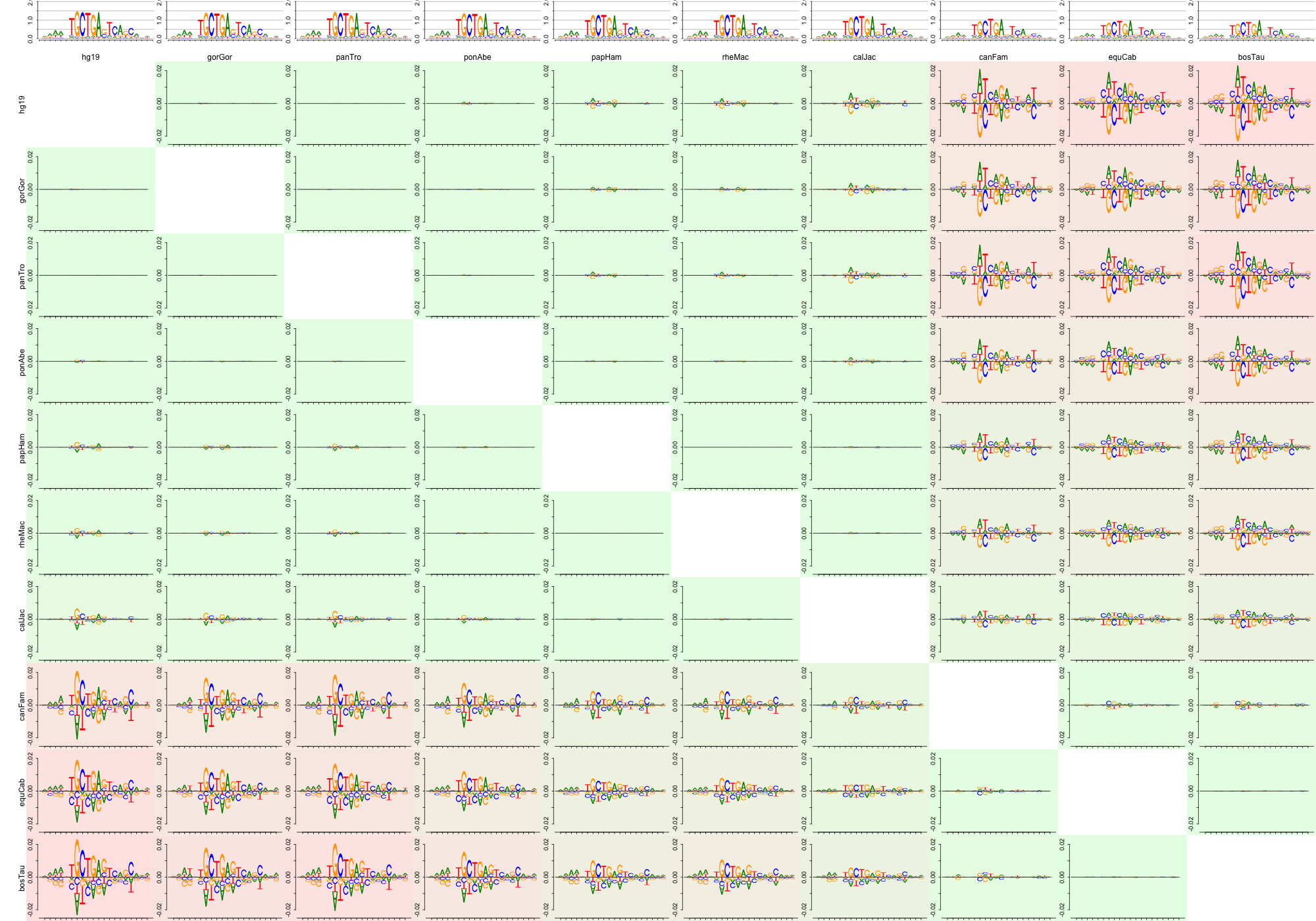

Supplement: Supplementary file 4 — Tables of difference logos. The file contains for each of the 35 TFs a 10×10 table of difference logos for a pair-wise visual comparison of species-specific motifs. (ZIP 26112 kb) [file 12859_2017_1495_MOESM4_ESM.zip › MafK.png]

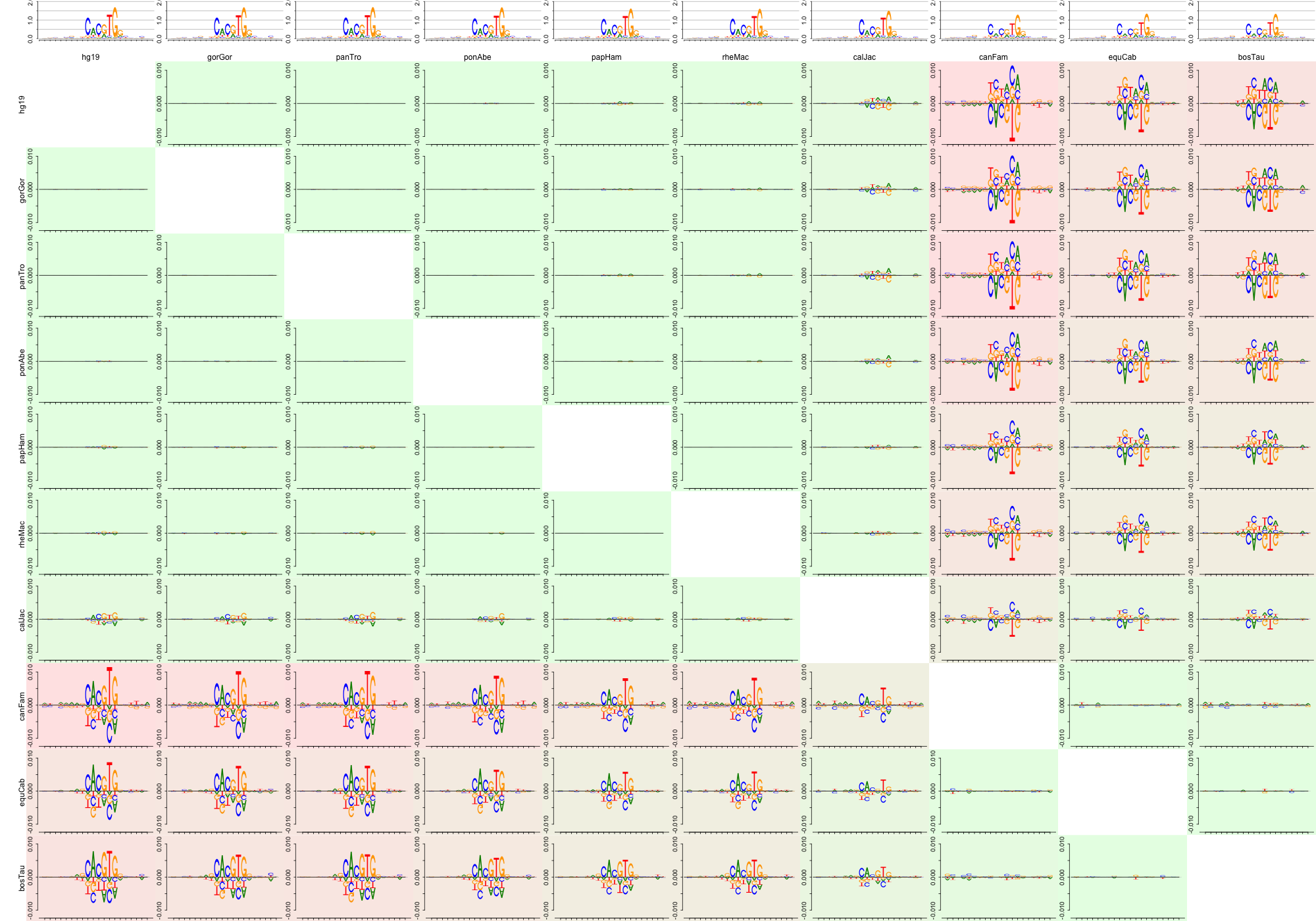

Supplement: Supplementary file 4 — Tables of difference logos. The file contains for each of the 35 TFs a 10×10 table of difference logos for a pair-wise visual comparison of species-specific motifs. (ZIP 26112 kb) [file 12859_2017_1495_MOESM4_ESM.zip › Max.png]

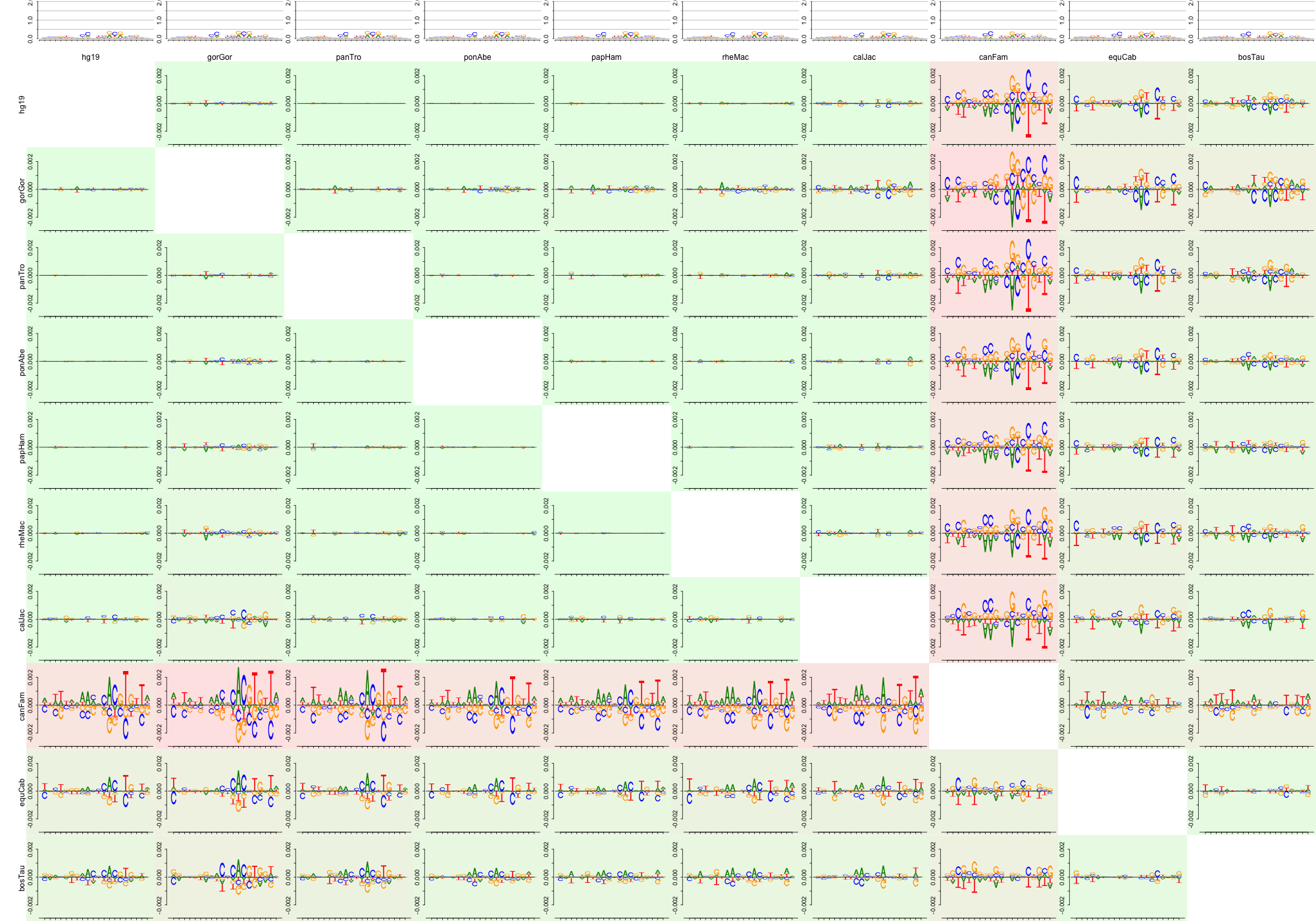

Supplement: Supplementary file 4 — Tables of difference logos. The file contains for each of the 35 TFs a 10×10 table of difference logos for a pair-wise visual comparison of species-specific motifs. (ZIP 26112 kb) [file 12859_2017_1495_MOESM4_ESM.zip › Mxi.png]

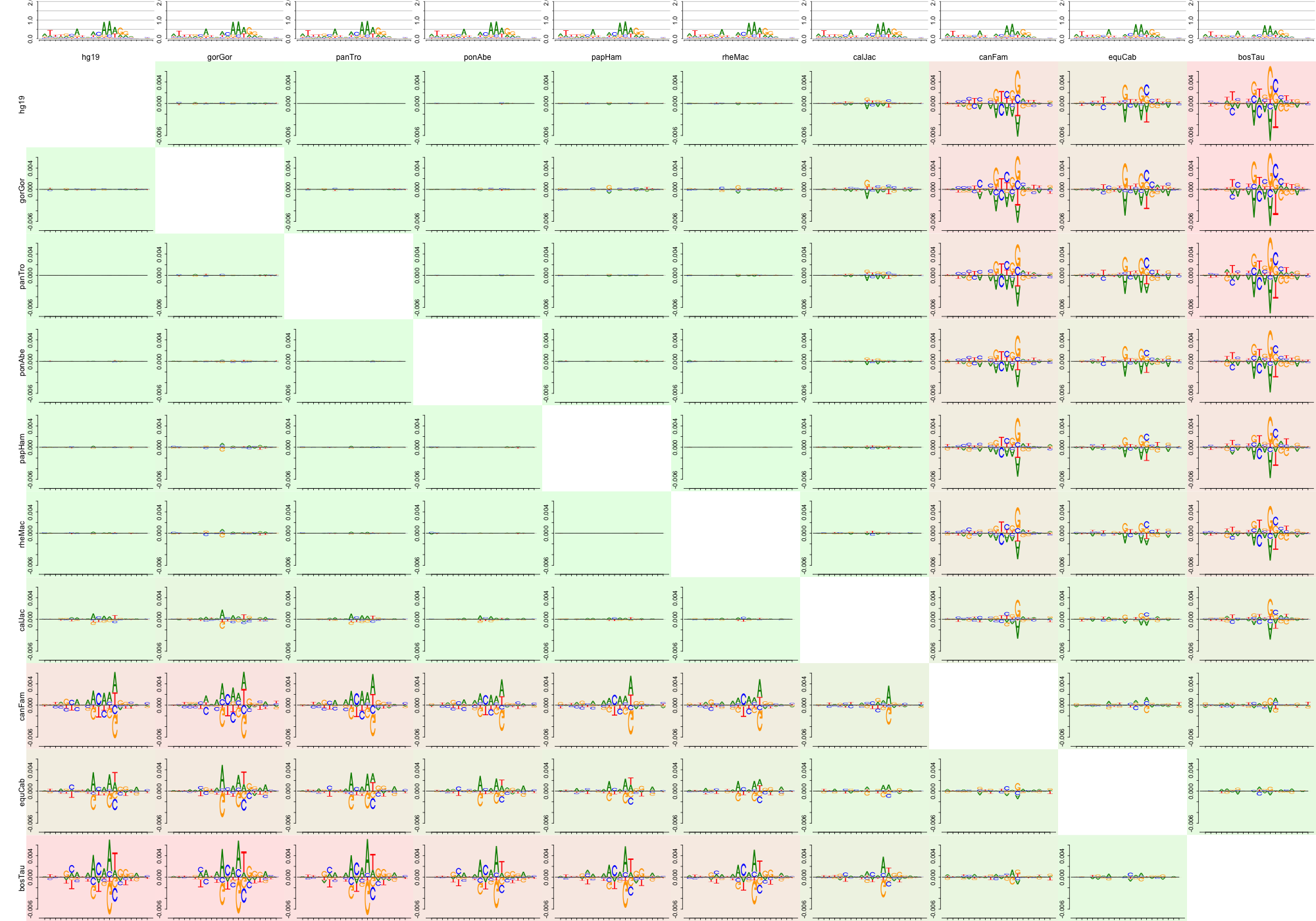

Supplement: Supplementary file 4 — Tables of difference logos. The file contains for each of the 35 TFs a 10×10 table of difference logos for a pair-wise visual comparison of species-specific motifs. (ZIP 26112 kb) [file 12859_2017_1495_MOESM4_ESM.zip › NANOG.png]

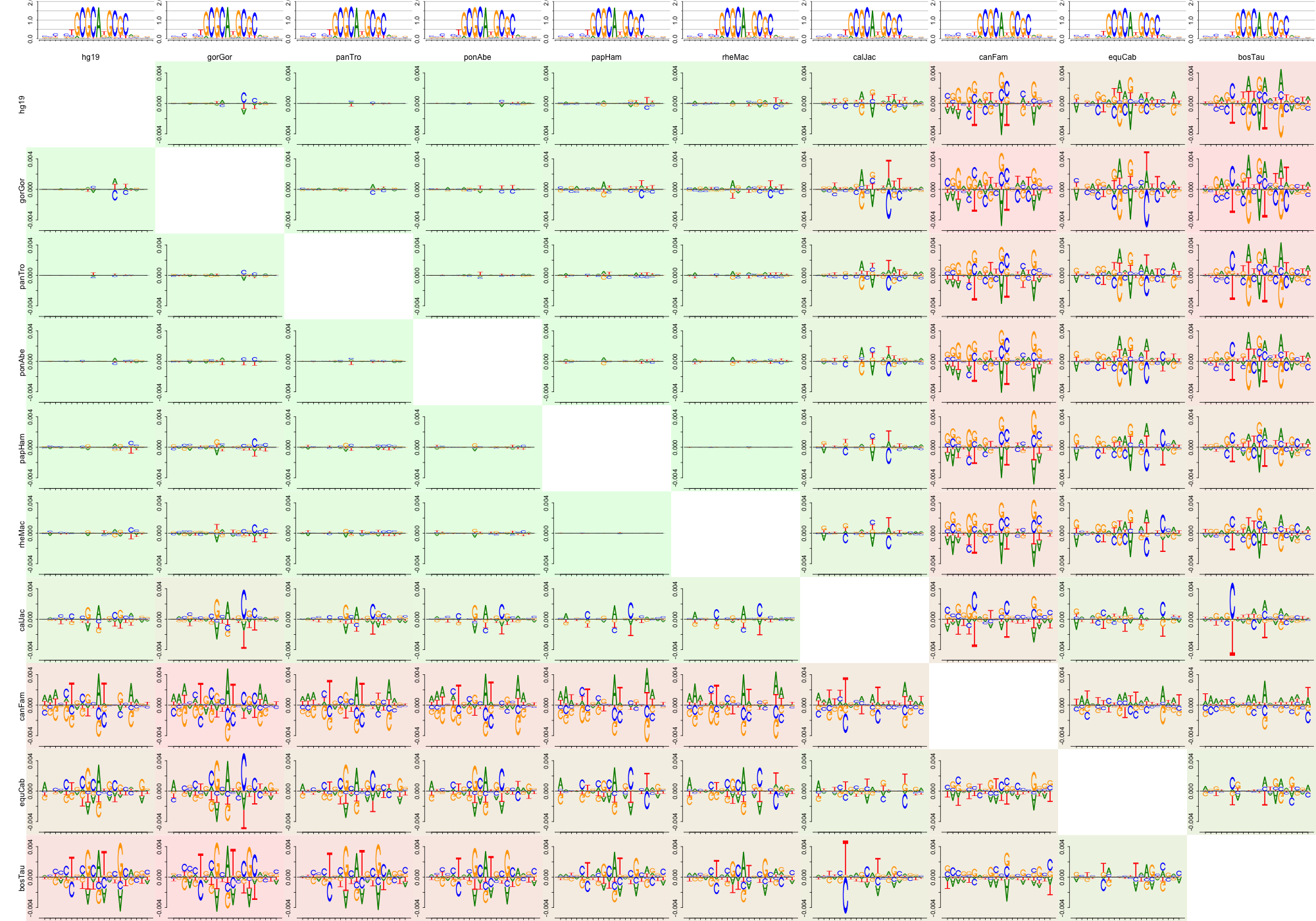

Supplement: Supplementary file 4 — Tables of difference logos. The file contains for each of the 35 TFs a 10×10 table of difference logos for a pair-wise visual comparison of species-specific motifs. (ZIP 26112 kb) [file 12859_2017_1495_MOESM4_ESM.zip › Nrf.png]

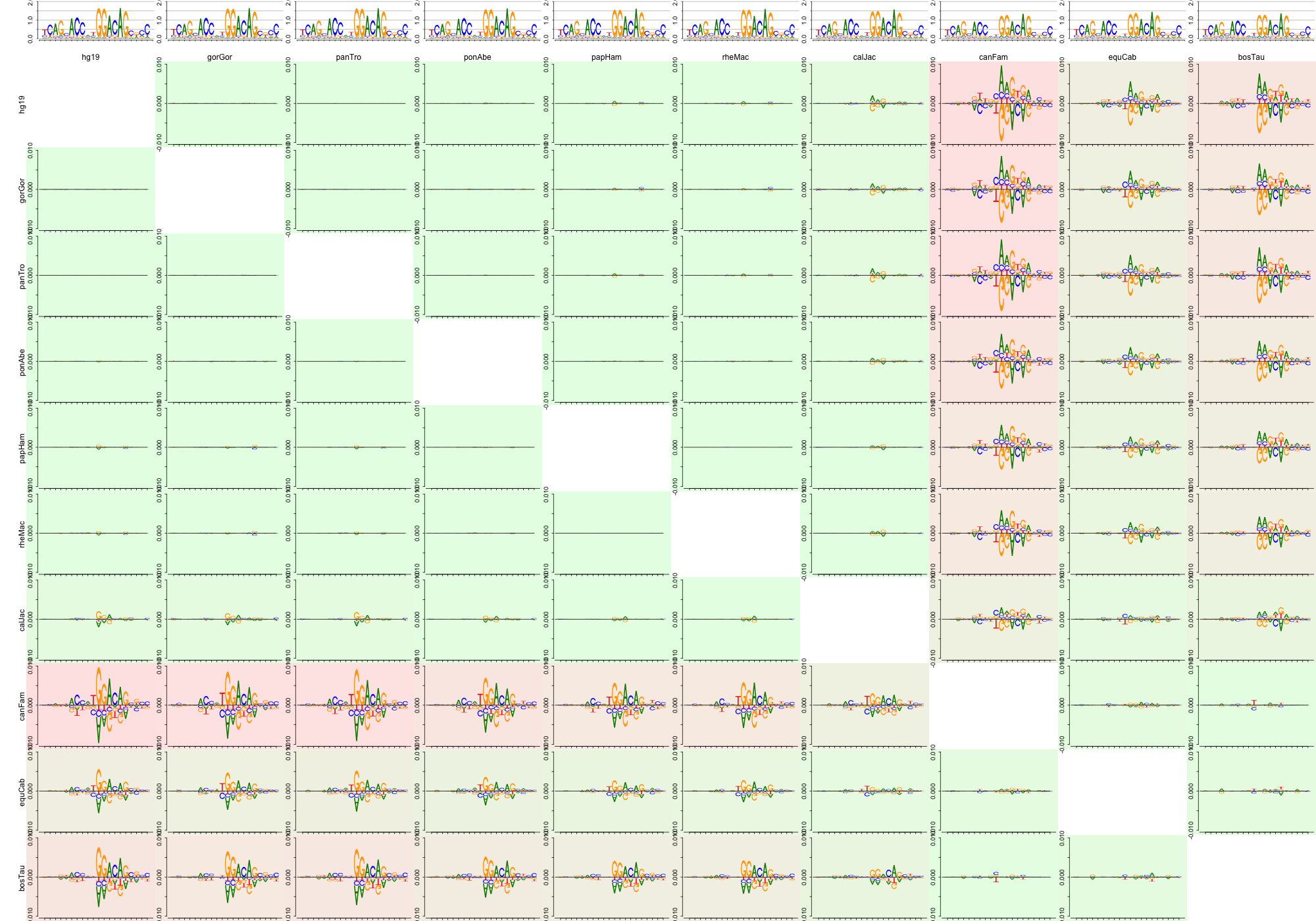

Supplement: Supplementary file 4 — Tables of difference logos. The file contains for each of the 35 TFs a 10×10 table of difference logos for a pair-wise visual comparison of species-specific motifs. (ZIP 26112 kb) [file 12859_2017_1495_MOESM4_ESM.zip › NRSF.png]

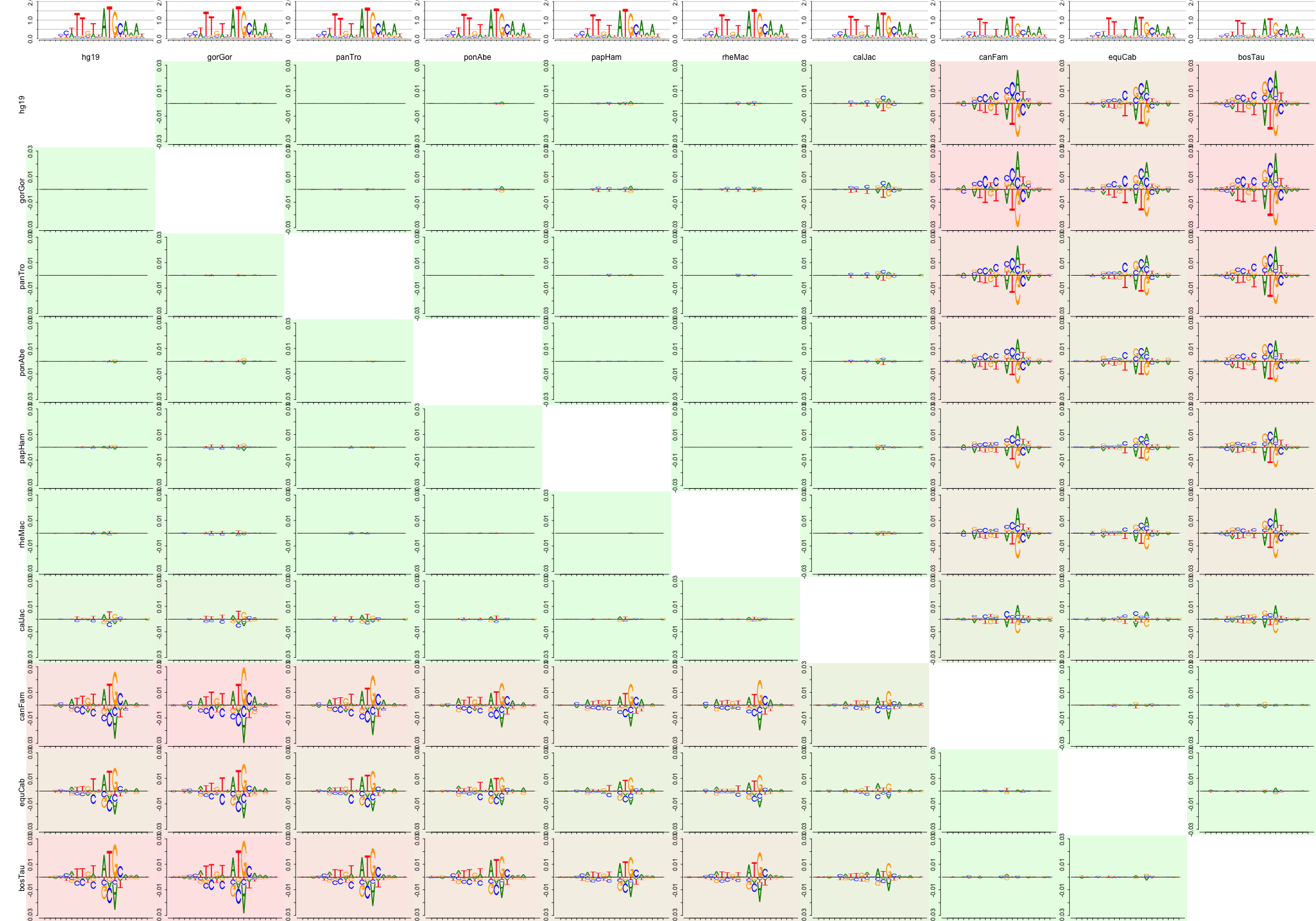

Supplement: Supplementary file 4 — Tables of difference logos. The file contains for each of the 35 TFs a 10×10 table of difference logos for a pair-wise visual comparison of species-specific motifs. (ZIP 26112 kb) [file 12859_2017_1495_MOESM4_ESM.zip › POU5F1.png]

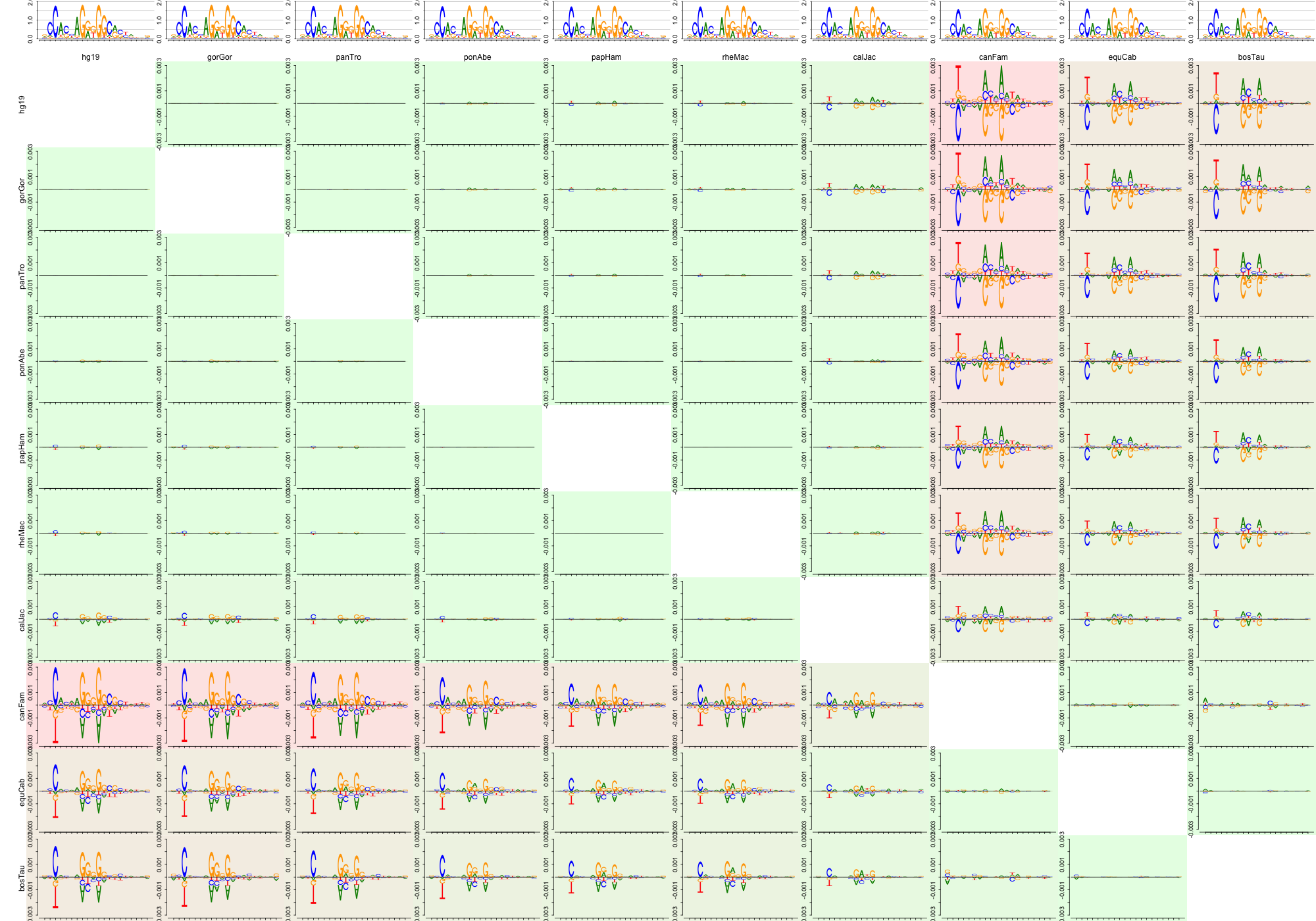

Supplement: Supplementary file 4 — Tables of difference logos. The file contains for each of the 35 TFs a 10×10 table of difference logos for a pair-wise visual comparison of species-specific motifs. (ZIP 26112 kb) [file 12859_2017_1495_MOESM4_ESM.zip › Rad21.png]

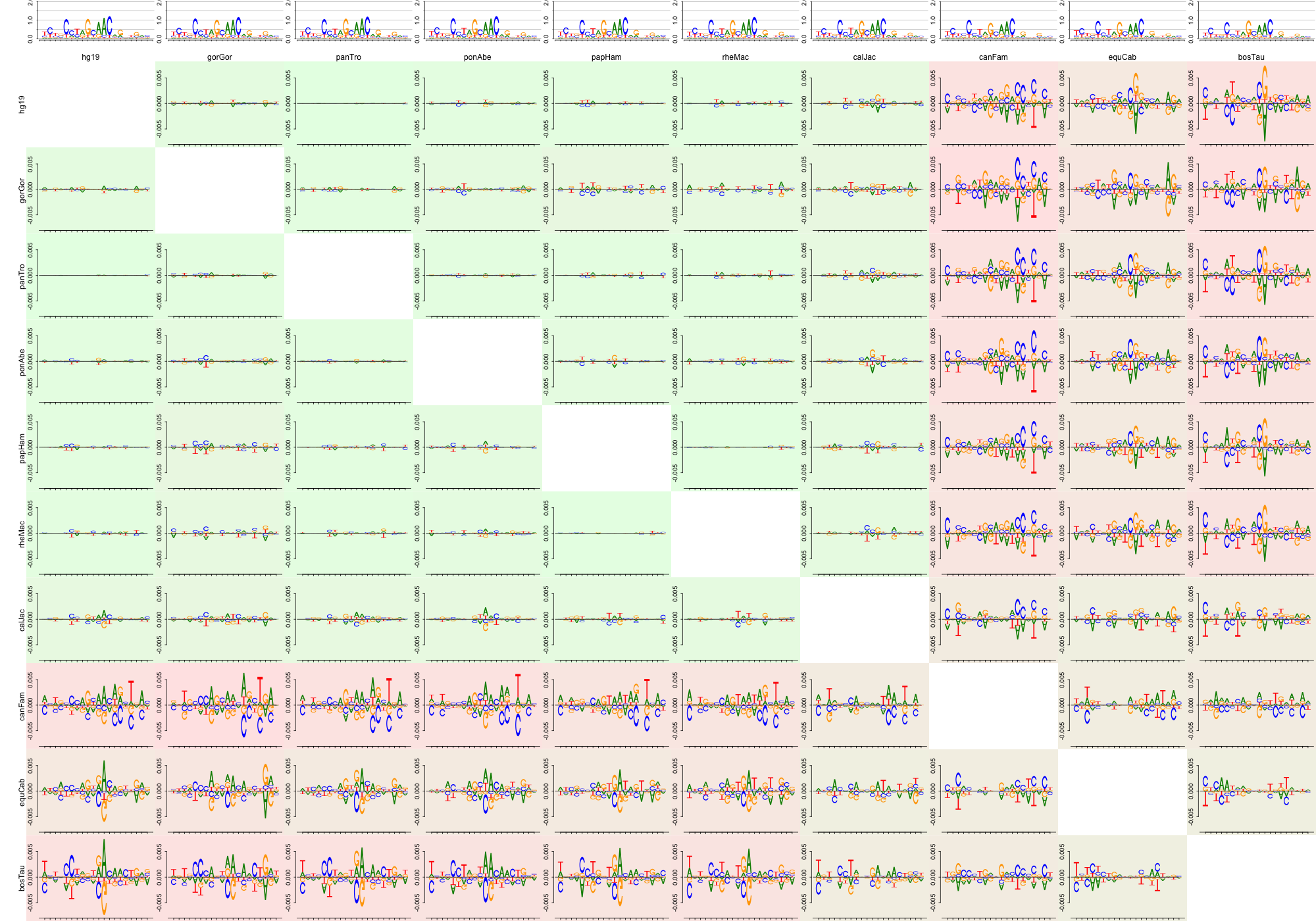

Supplement: Supplementary file 4 — Tables of difference logos. The file contains for each of the 35 TFs a 10×10 table of difference logos for a pair-wise visual comparison of species-specific motifs. (ZIP 26112 kb) [file 12859_2017_1495_MOESM4_ESM.zip › RFX5.png]

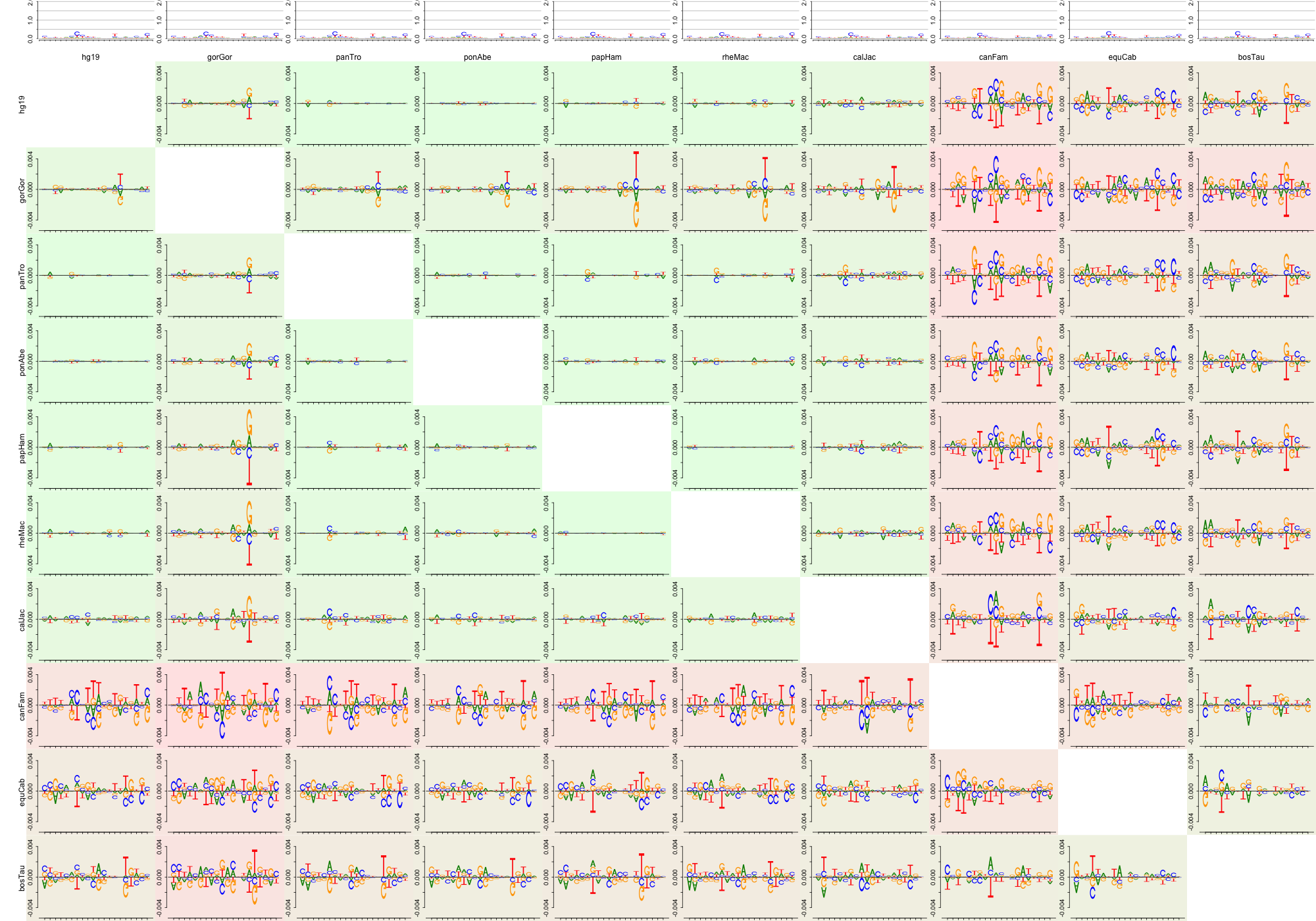

Supplement: Supplementary file 4 — Tables of difference logos. The file contains for each of the 35 TFs a 10×10 table of difference logos for a pair-wise visual comparison of species-specific motifs. (ZIP 26112 kb) [file 12859_2017_1495_MOESM4_ESM.zip › RXRA.png]

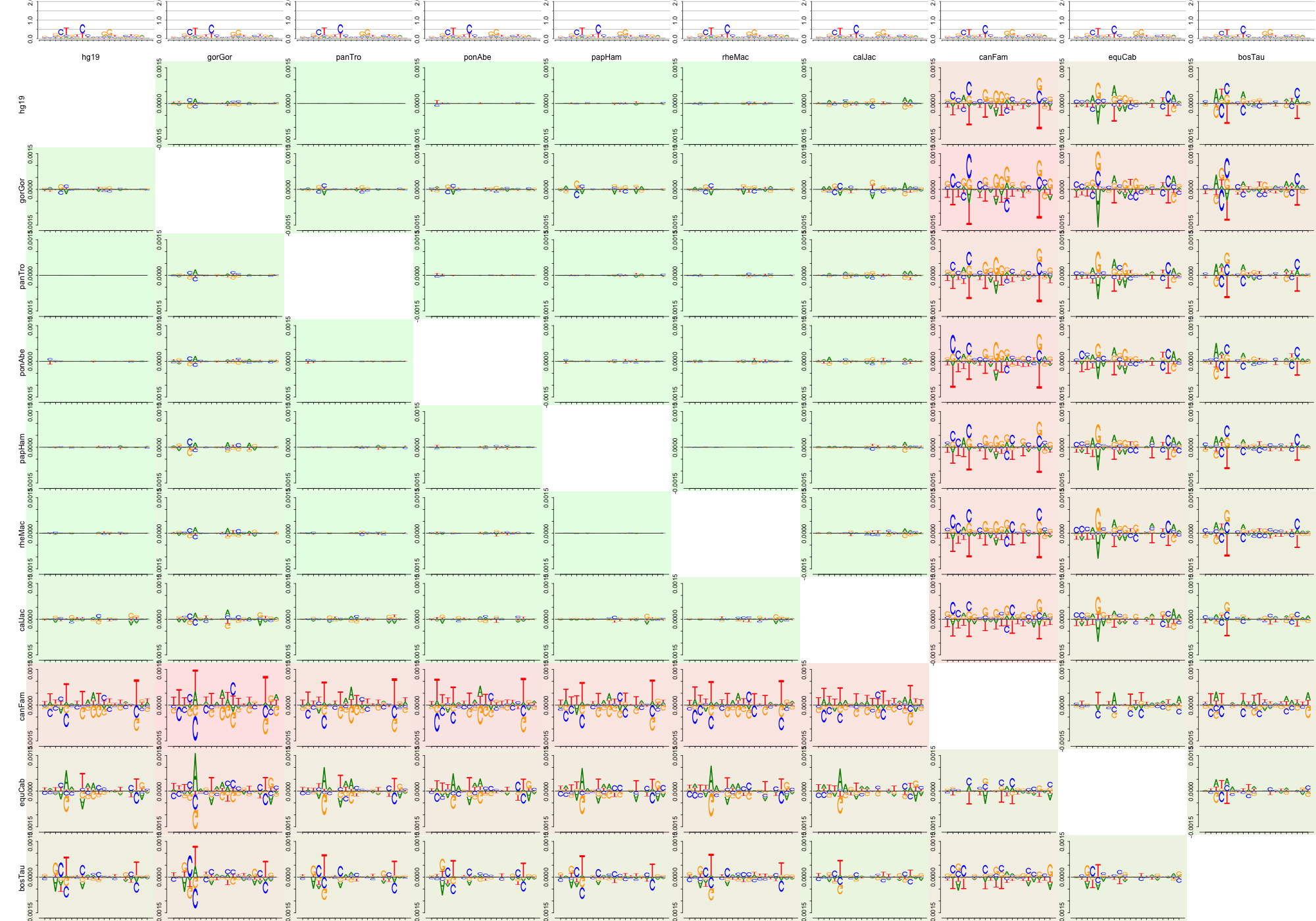

Supplement: Supplementary file 4 — Tables of difference logos. The file contains for each of the 35 TFs a 10×10 table of difference logos for a pair-wise visual comparison of species-specific motifs. (ZIP 26112 kb) [file 12859_2017_1495_MOESM4_ESM.zip › Sin3Ak.png]

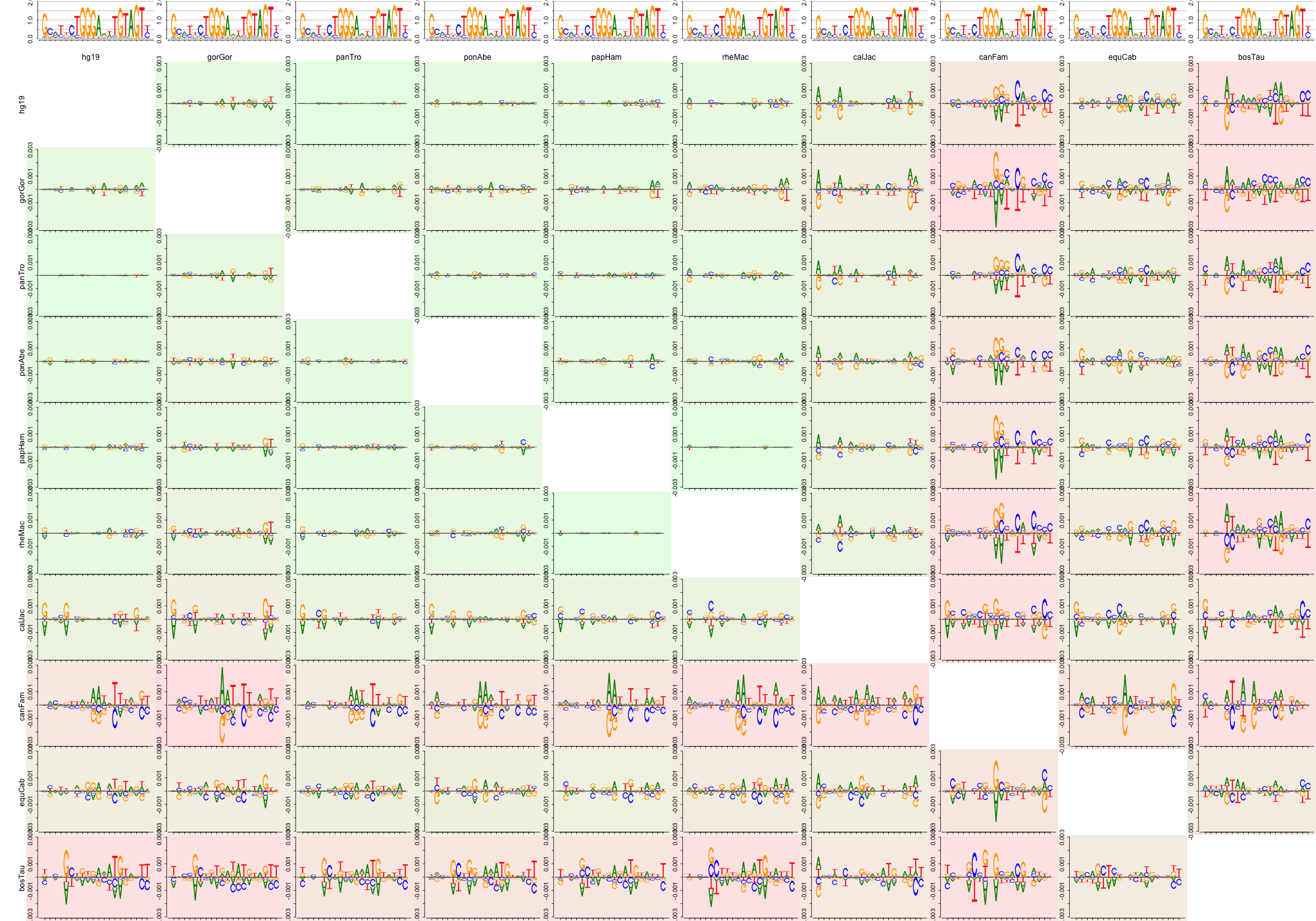

Supplement: Supplementary file 4 — Tables of difference logos. The file contains for each of the 35 TFs a 10×10 table of difference logos for a pair-wise visual comparison of species-specific motifs. (ZIP 26112 kb) [file 12859_2017_1495_MOESM4_ESM.zip › SIX5.png]

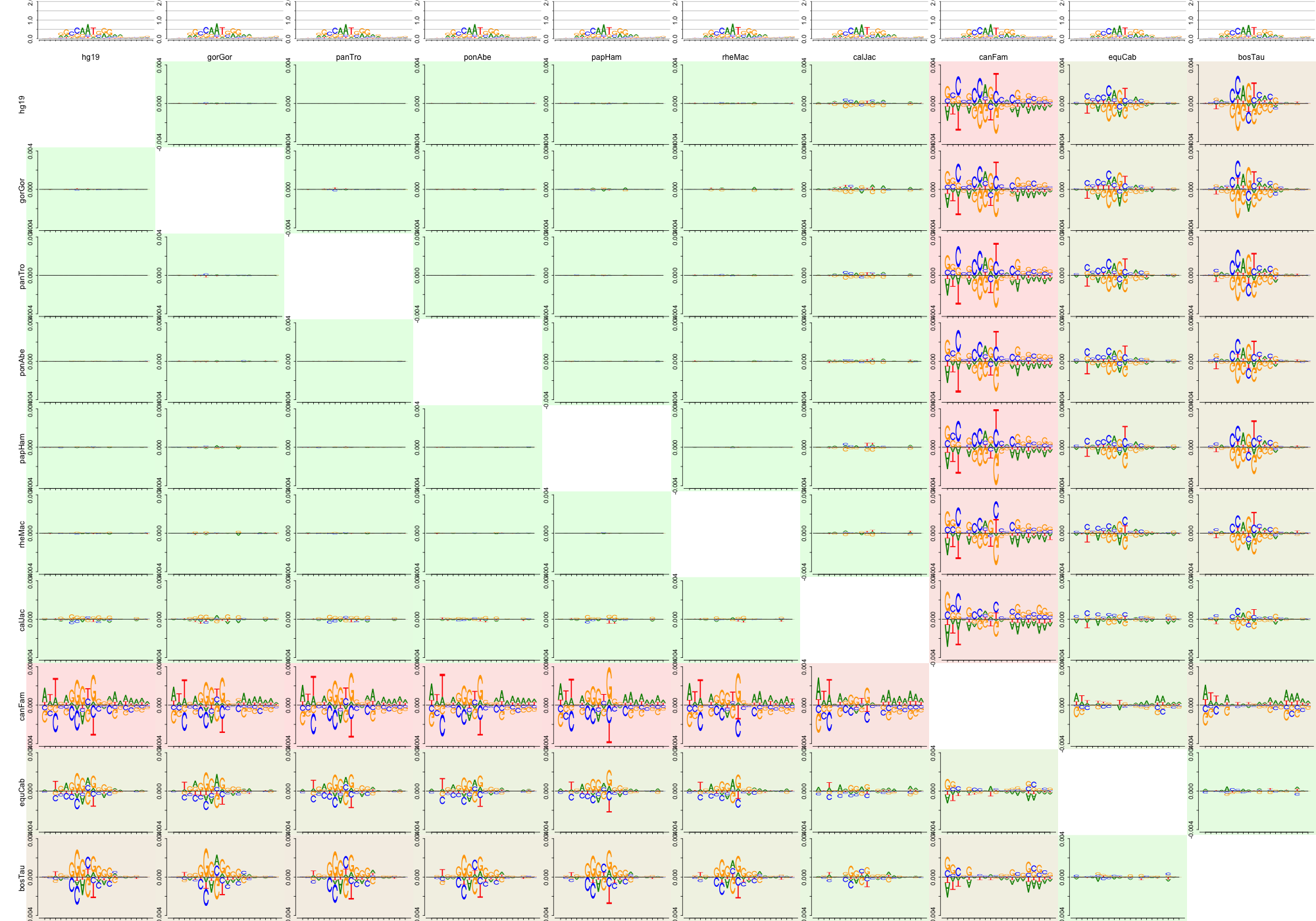

Supplement: Supplementary file 4 — Tables of difference logos. The file contains for each of the 35 TFs a 10×10 table of difference logos for a pair-wise visual comparison of species-specific motifs. (ZIP 26112 kb) [file 12859_2017_1495_MOESM4_ESM.zip › SP1.png]

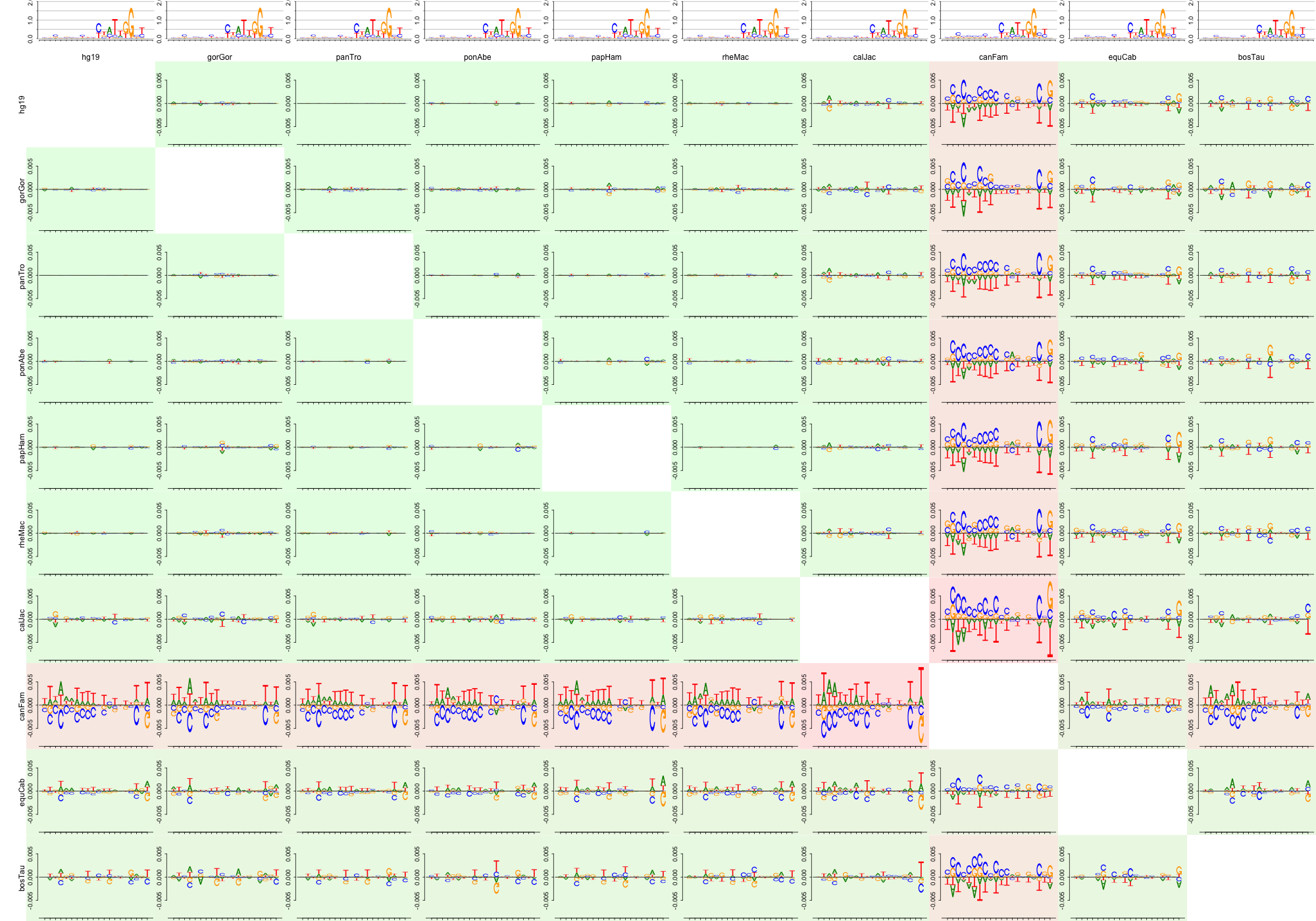

Supplement: Supplementary file 4 — Tables of difference logos. The file contains for each of the 35 TFs a 10×10 table of difference logos for a pair-wise visual comparison of species-specific motifs. (ZIP 26112 kb) [file 12859_2017_1495_MOESM4_ESM.zip › SP2.png]

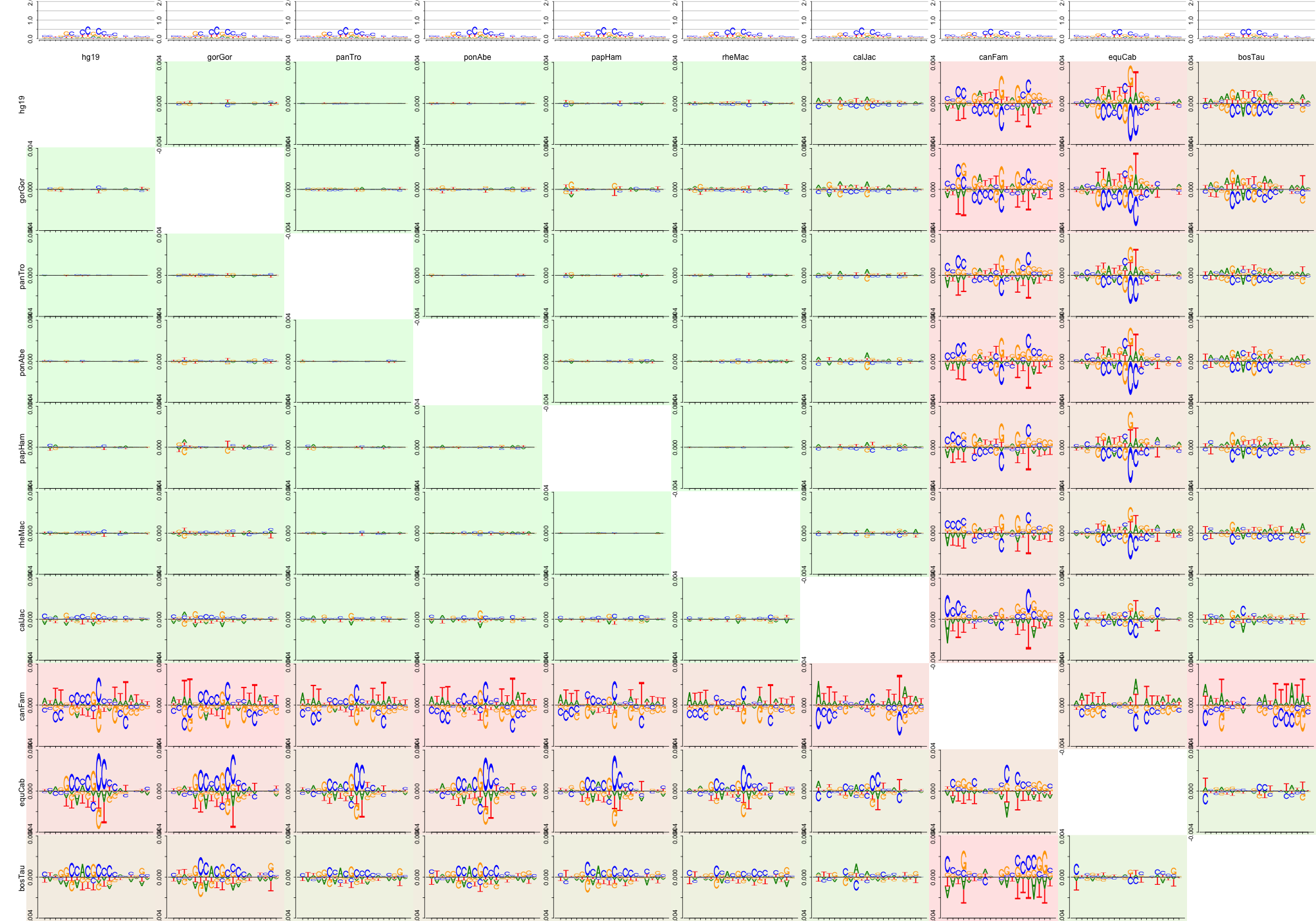

Supplement: Supplementary file 4 — Tables of difference logos. The file contains for each of the 35 TFs a 10×10 table of difference logos for a pair-wise visual comparison of species-specific motifs. (ZIP 26112 kb) [file 12859_2017_1495_MOESM4_ESM.zip › SP4.png]

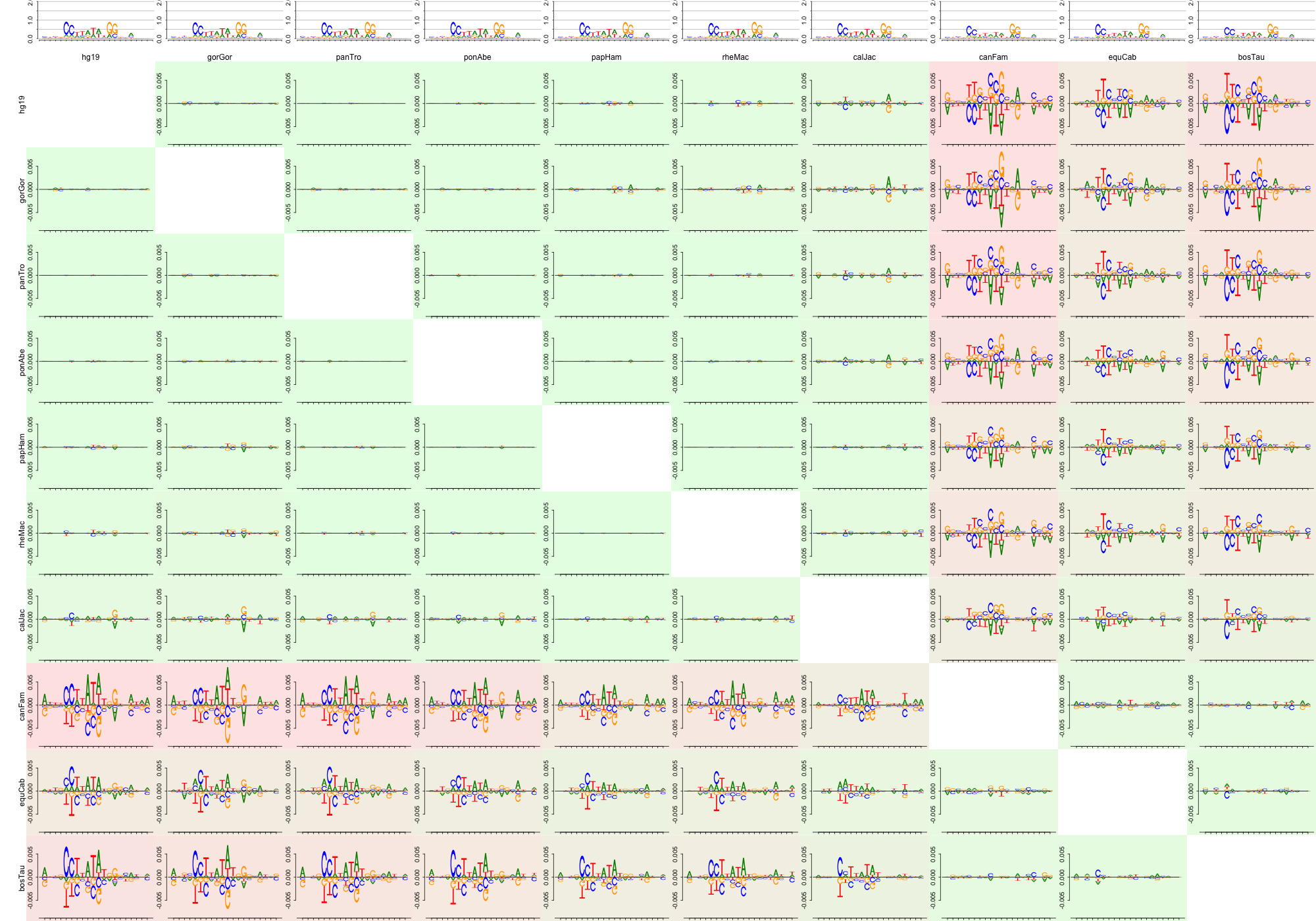

Supplement: Supplementary file 4 — Tables of difference logos. The file contains for each of the 35 TFs a 10×10 table of difference logos for a pair-wise visual comparison of species-specific motifs. (ZIP 26112 kb) [file 12859_2017_1495_MOESM4_ESM.zip › SRF.png]

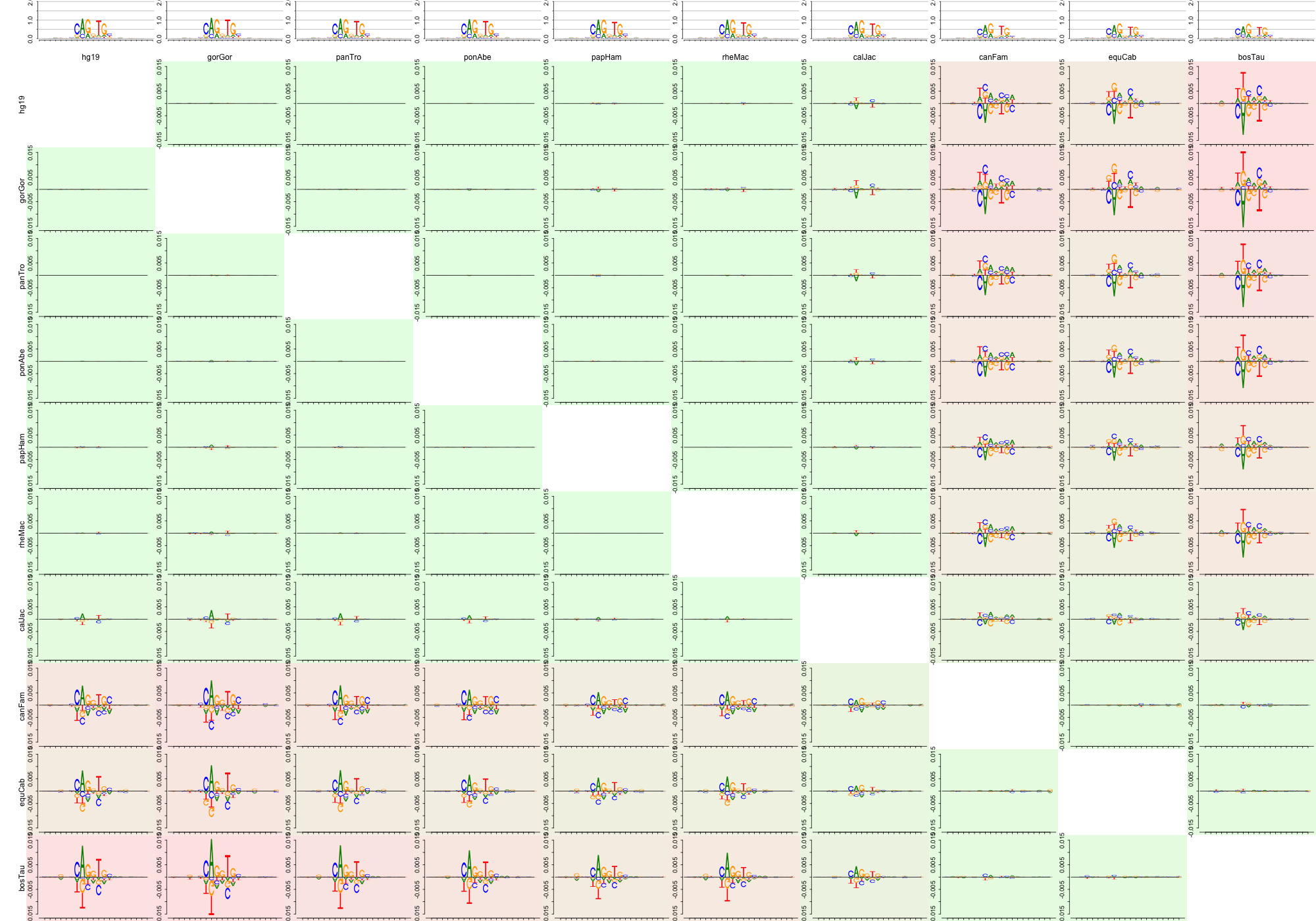

Supplement: Supplementary file 4 — Tables of difference logos. The file contains for each of the 35 TFs a 10×10 table of difference logos for a pair-wise visual comparison of species-specific motifs. (ZIP 26112 kb) [file 12859_2017_1495_MOESM4_ESM.zip › TCF12.png]

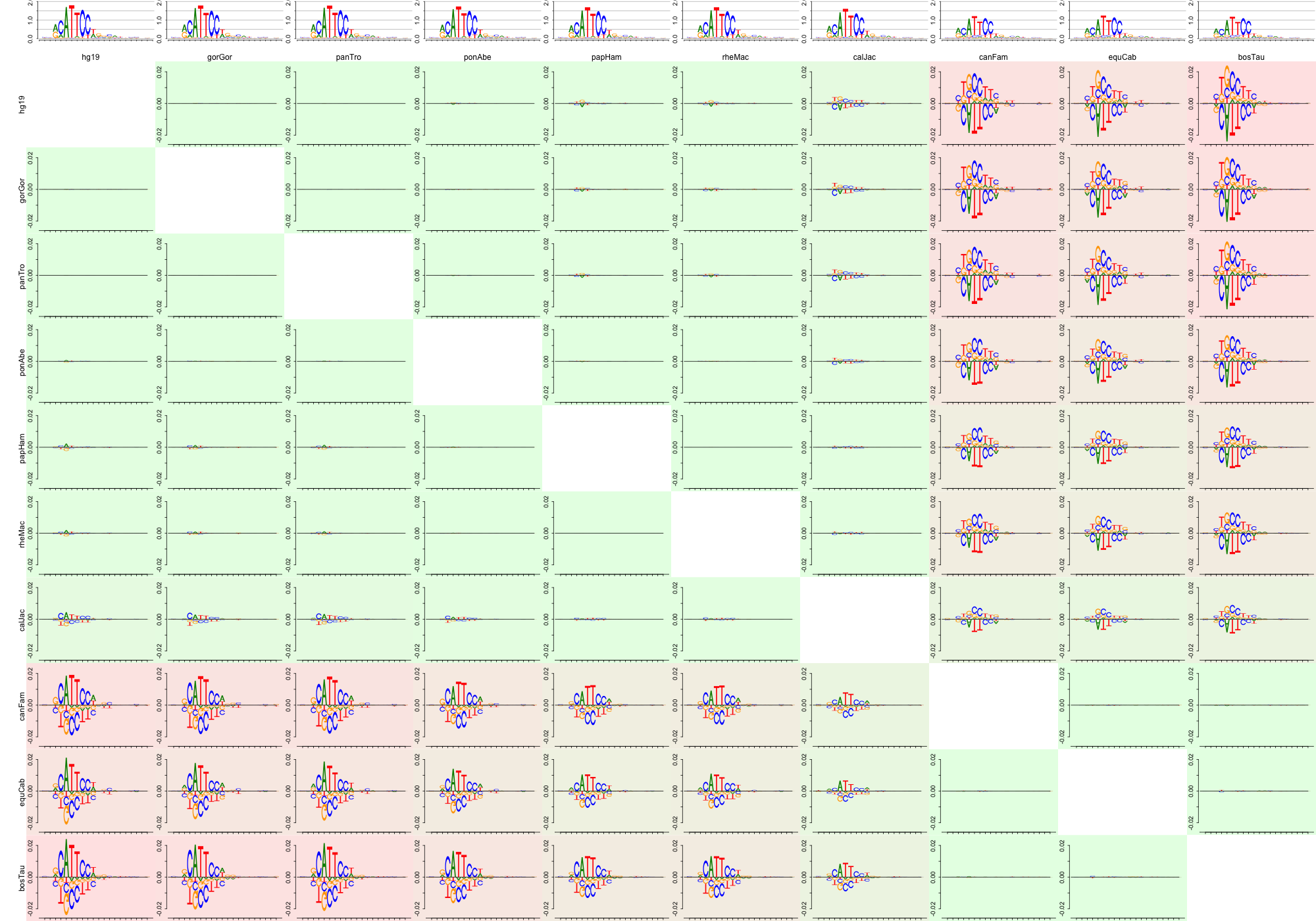

Supplement: Supplementary file 4 — Tables of difference logos. The file contains for each of the 35 TFs a 10×10 table of difference logos for a pair-wise visual comparison of species-specific motifs. (ZIP 26112 kb) [file 12859_2017_1495_MOESM4_ESM.zip › TEAD4.png]

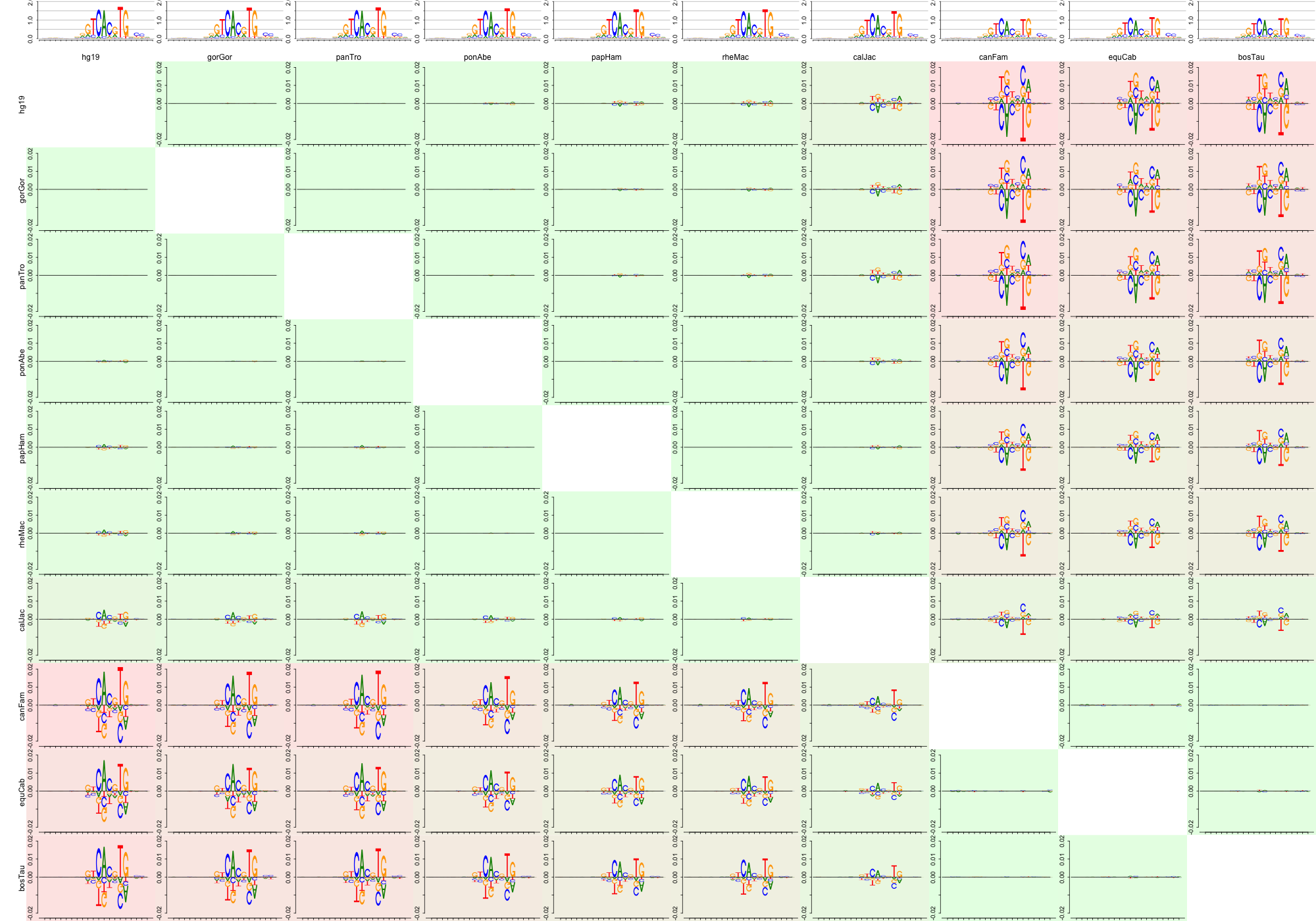

Supplement: Supplementary file 4 — Tables of difference logos. The file contains for each of the 35 TFs a 10×10 table of difference logos for a pair-wise visual comparison of species-specific motifs. (ZIP 26112 kb) [file 12859_2017_1495_MOESM4_ESM.zip › USF1.png]

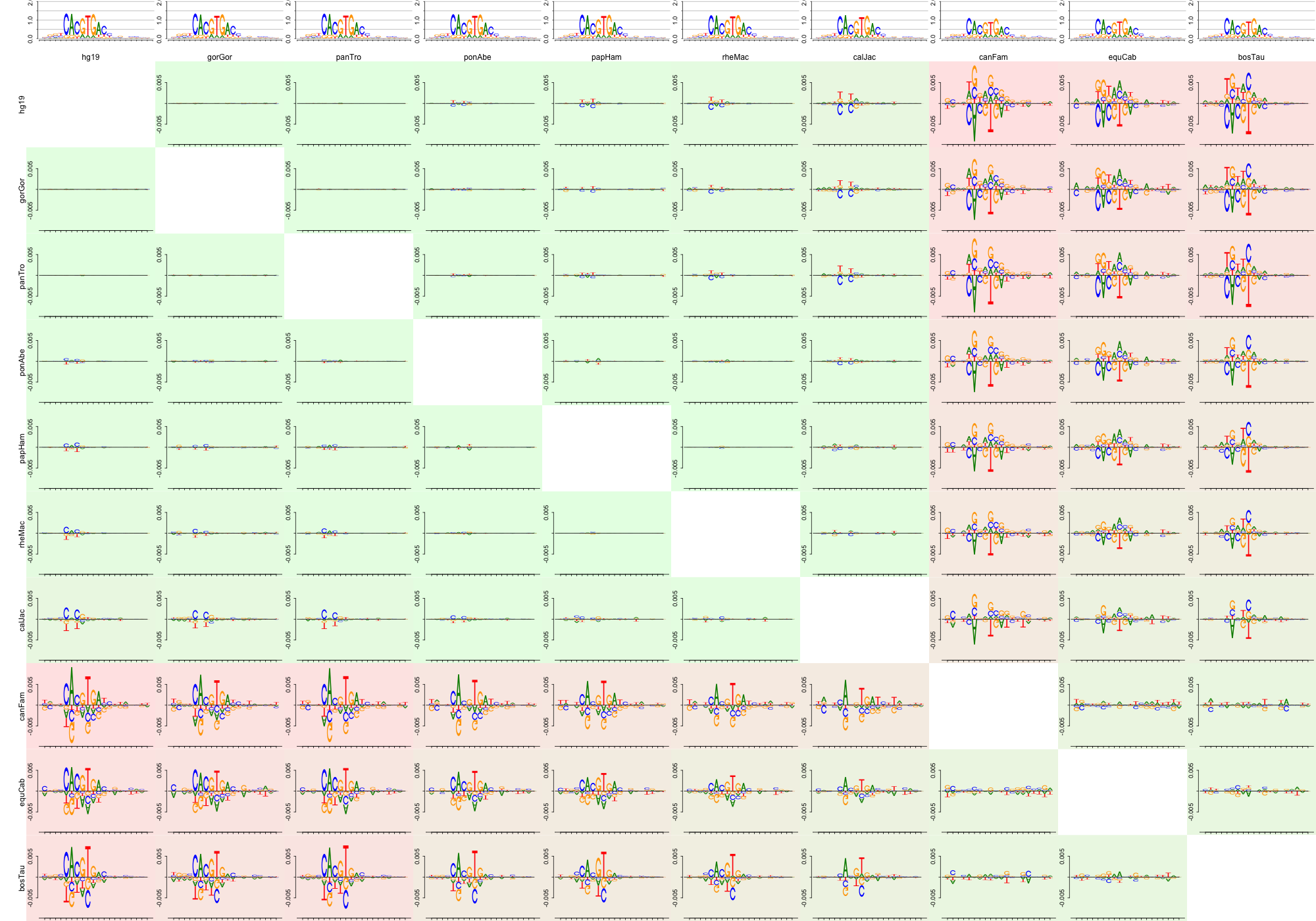

Supplement: Supplementary file 4 — Tables of difference logos. The file contains for each of the 35 TFs a 10×10 table of difference logos for a pair-wise visual comparison of species-specific motifs. (ZIP 26112 kb) [file 12859_2017_1495_MOESM4_ESM.zip › USF2.png]

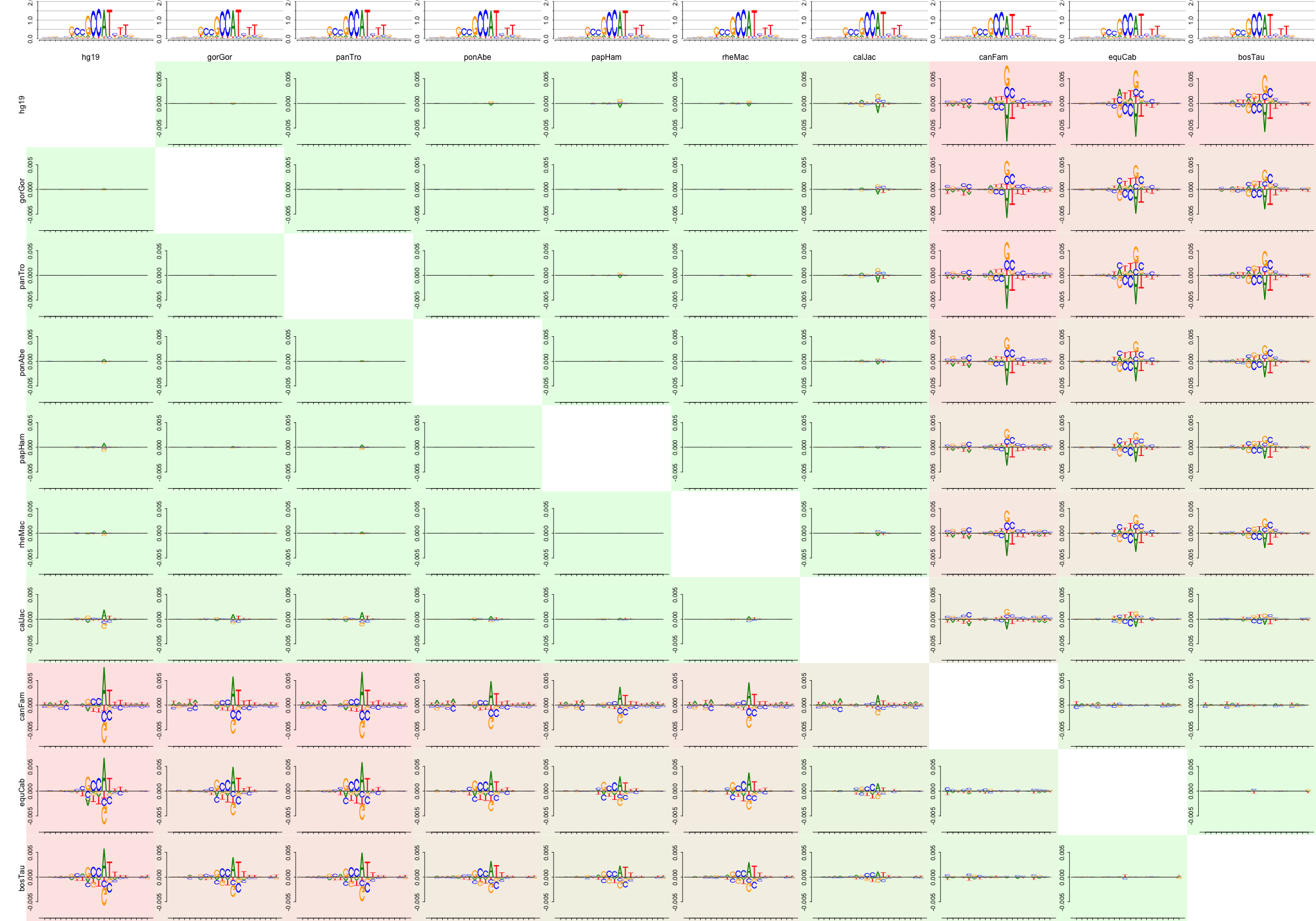

Supplement: Supplementary file 4 — Tables of difference logos. The file contains for each of the 35 TFs a 10×10 table of difference logos for a pair-wise visual comparison of species-specific motifs. (ZIP 26112 kb) [file 12859_2017_1495_MOESM4_ESM.zip › YY1.png]

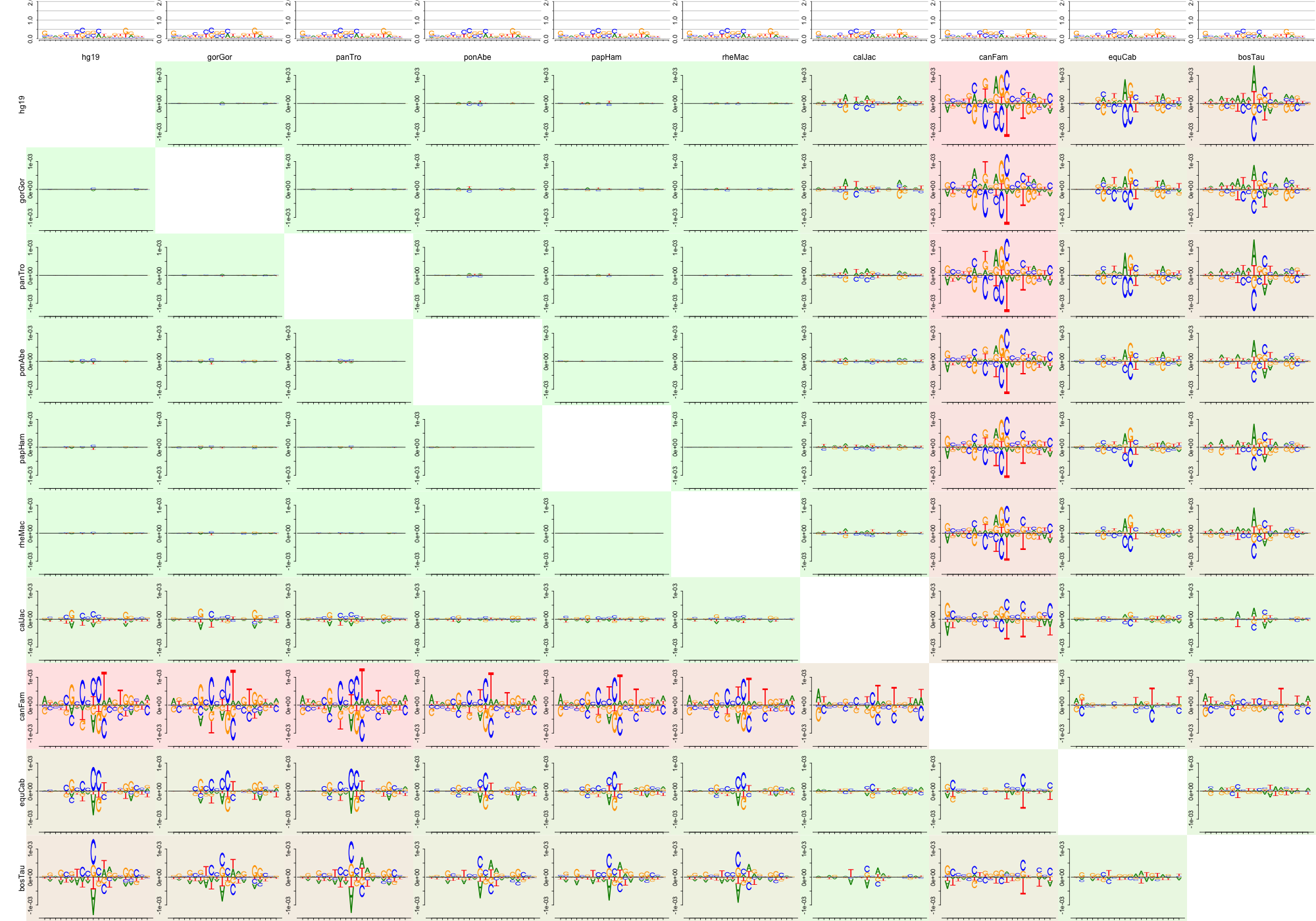

Supplement: Supplementary file 4 — Tables of difference logos. The file contains for each of the 35 TFs a 10×10 table of difference logos for a pair-wise visual comparison of species-specific motifs. (ZIP 26112 kb) [file 12859_2017_1495_MOESM4_ESM.zip › Znf143.png]
