# Supplementary material for: Combining phylogenetic footprinting with motif models incorporating intra-motif dependencies
Source: BMC Bioinformatics. 2017 Mar 1;18:141. doi: 10.1186/s12859-017-1495-1 (PMC5333389; doi:10.1186/s12859-017-1495-1)

ATF3

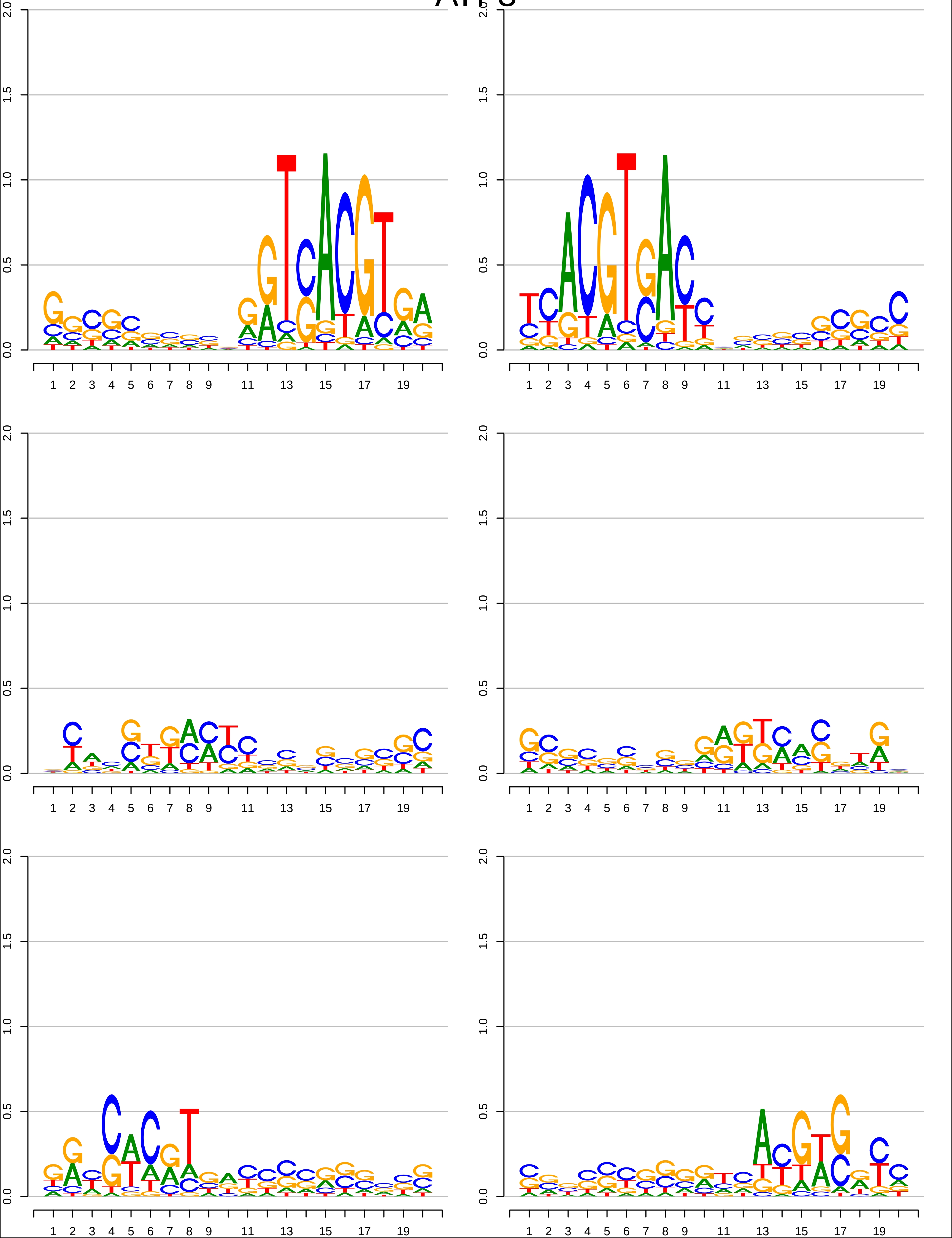

Bach1

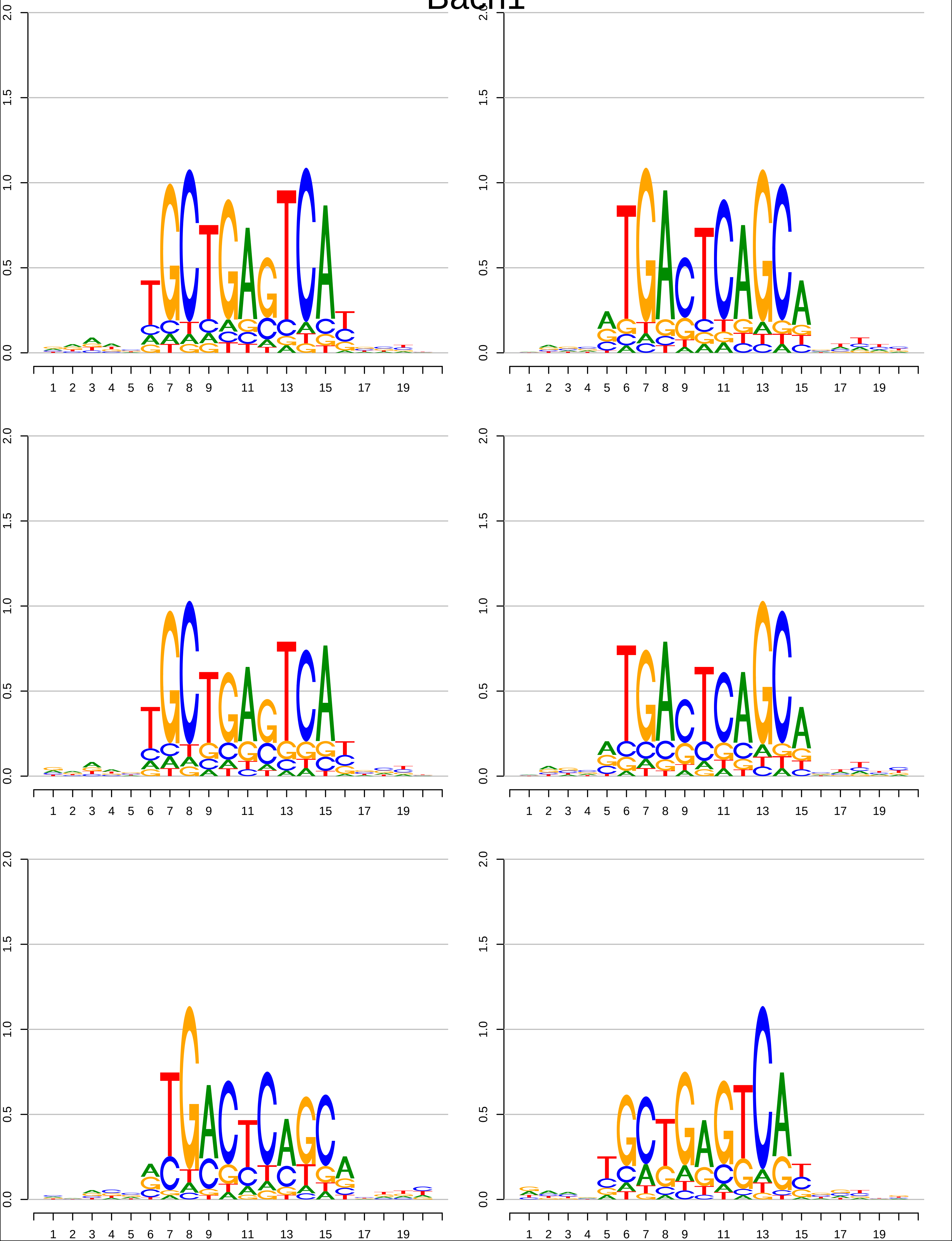

BCL11A

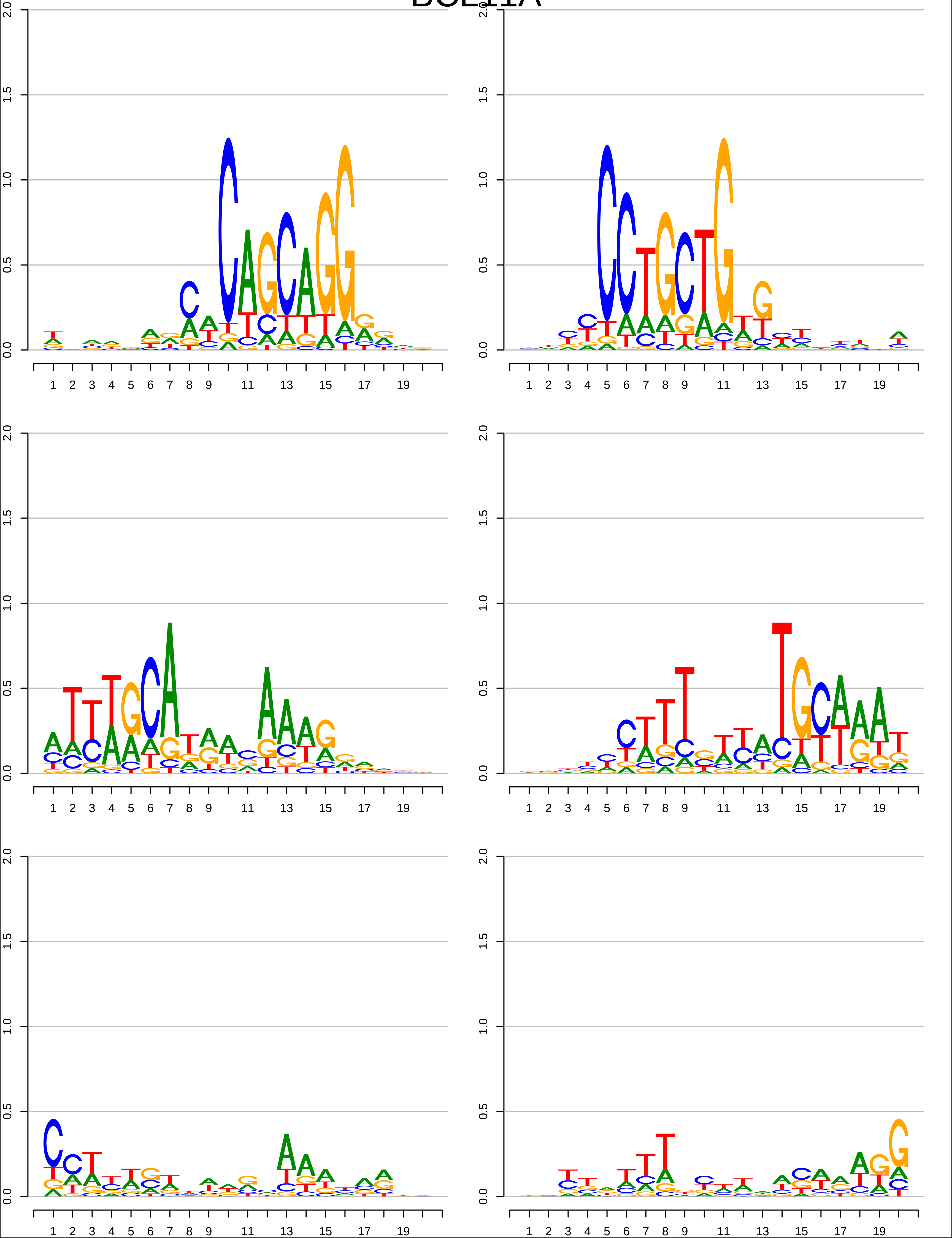

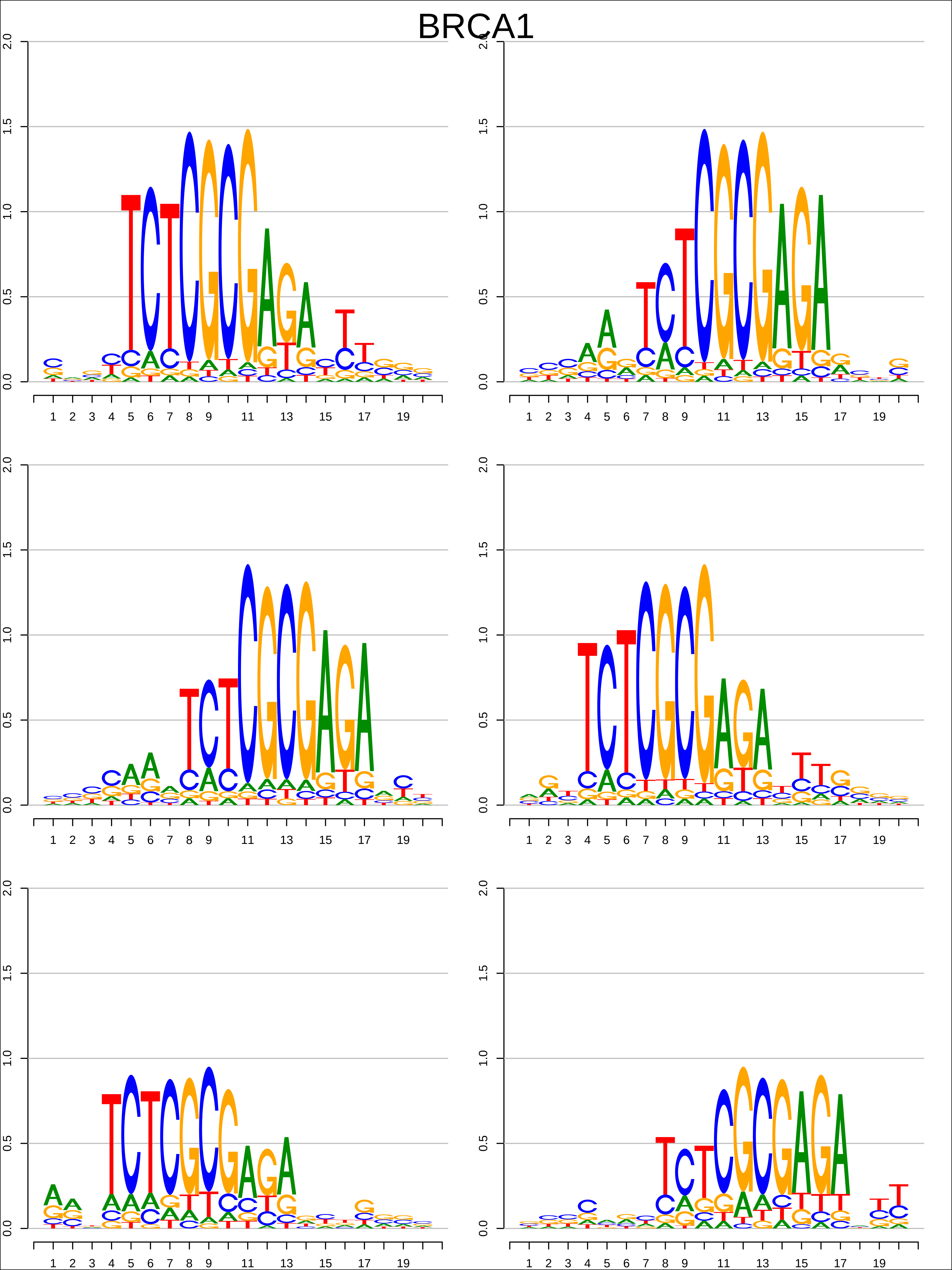
BRCA1

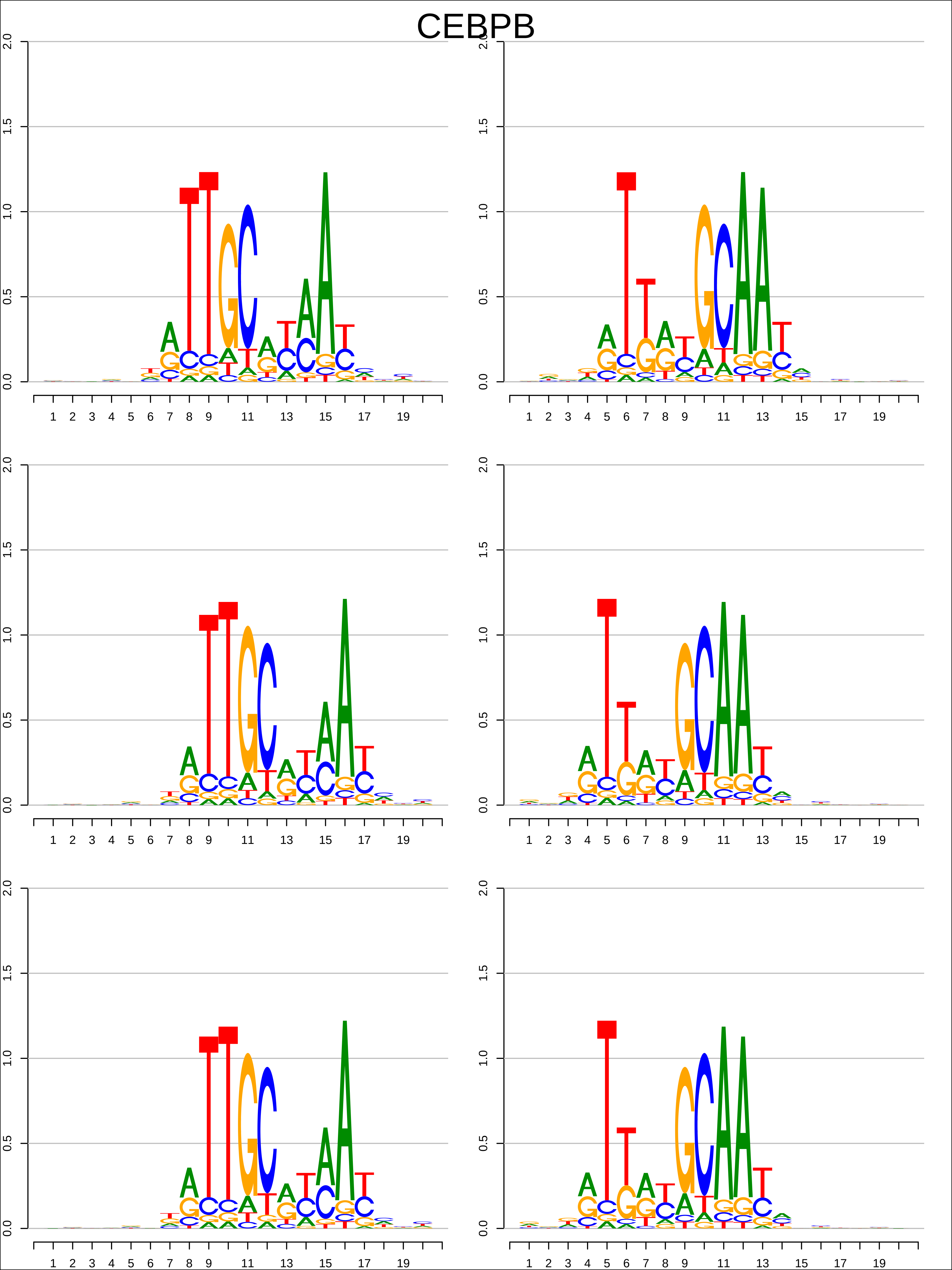
CEBPB

CHD2

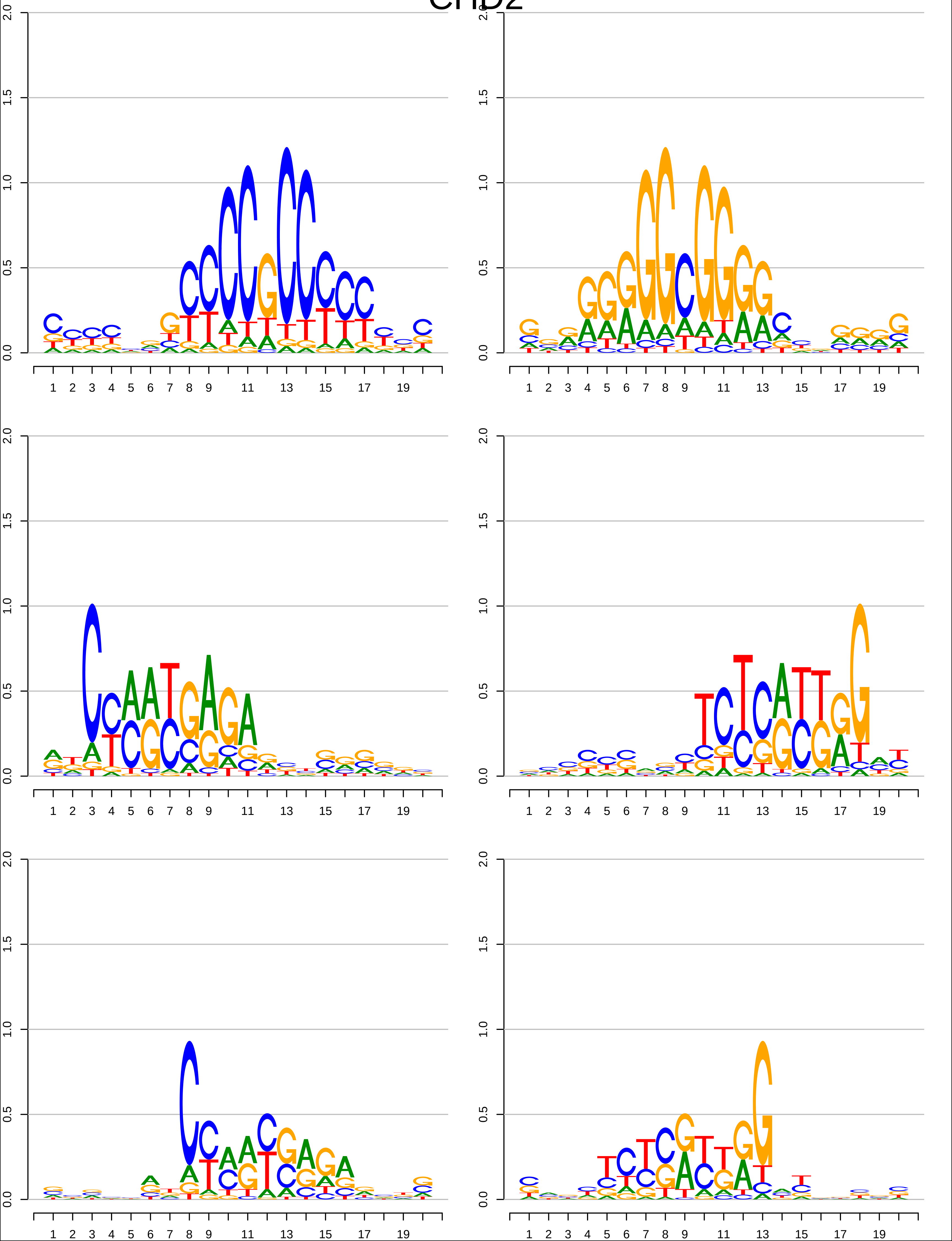

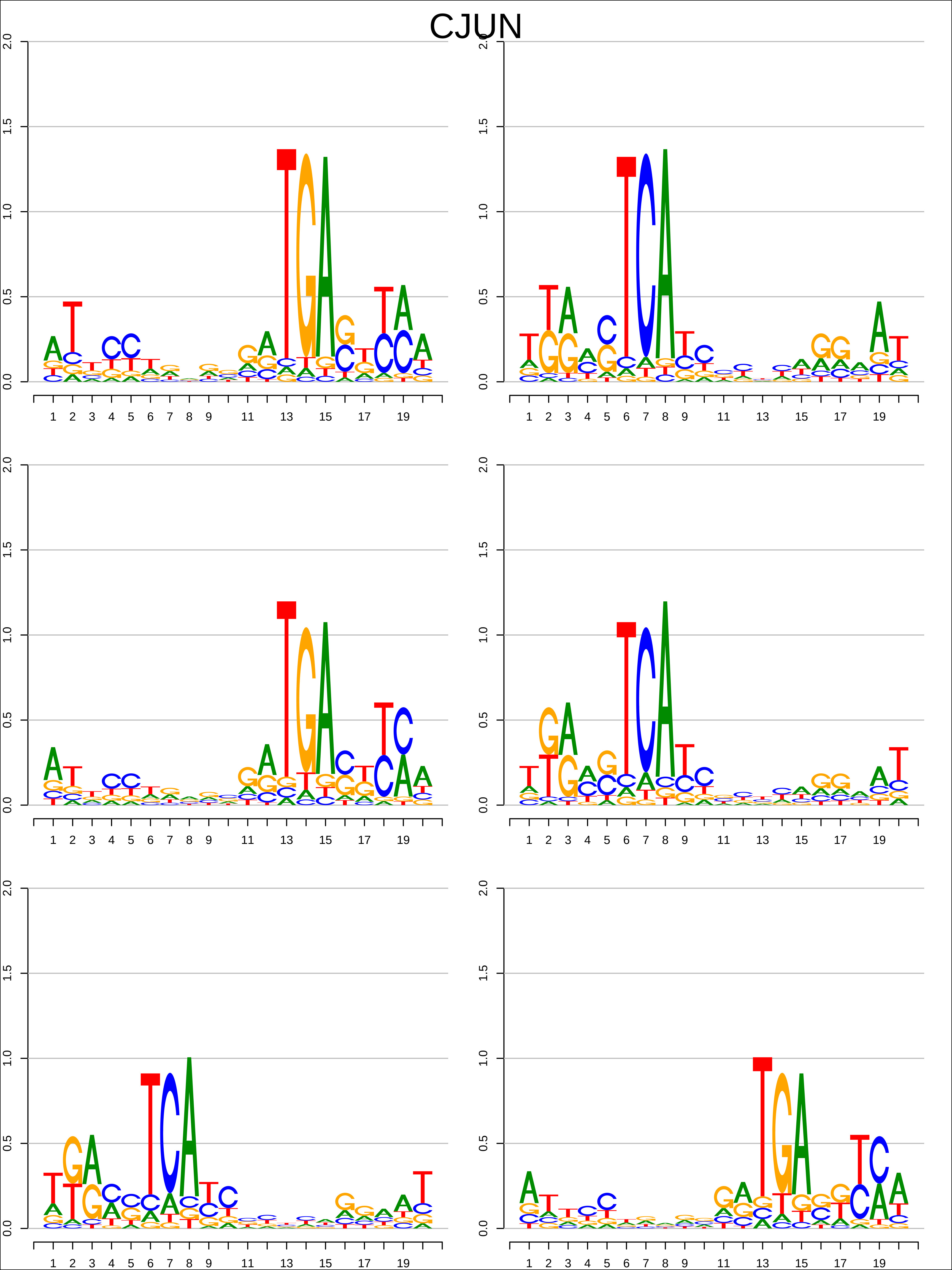
CJUN

CTCF

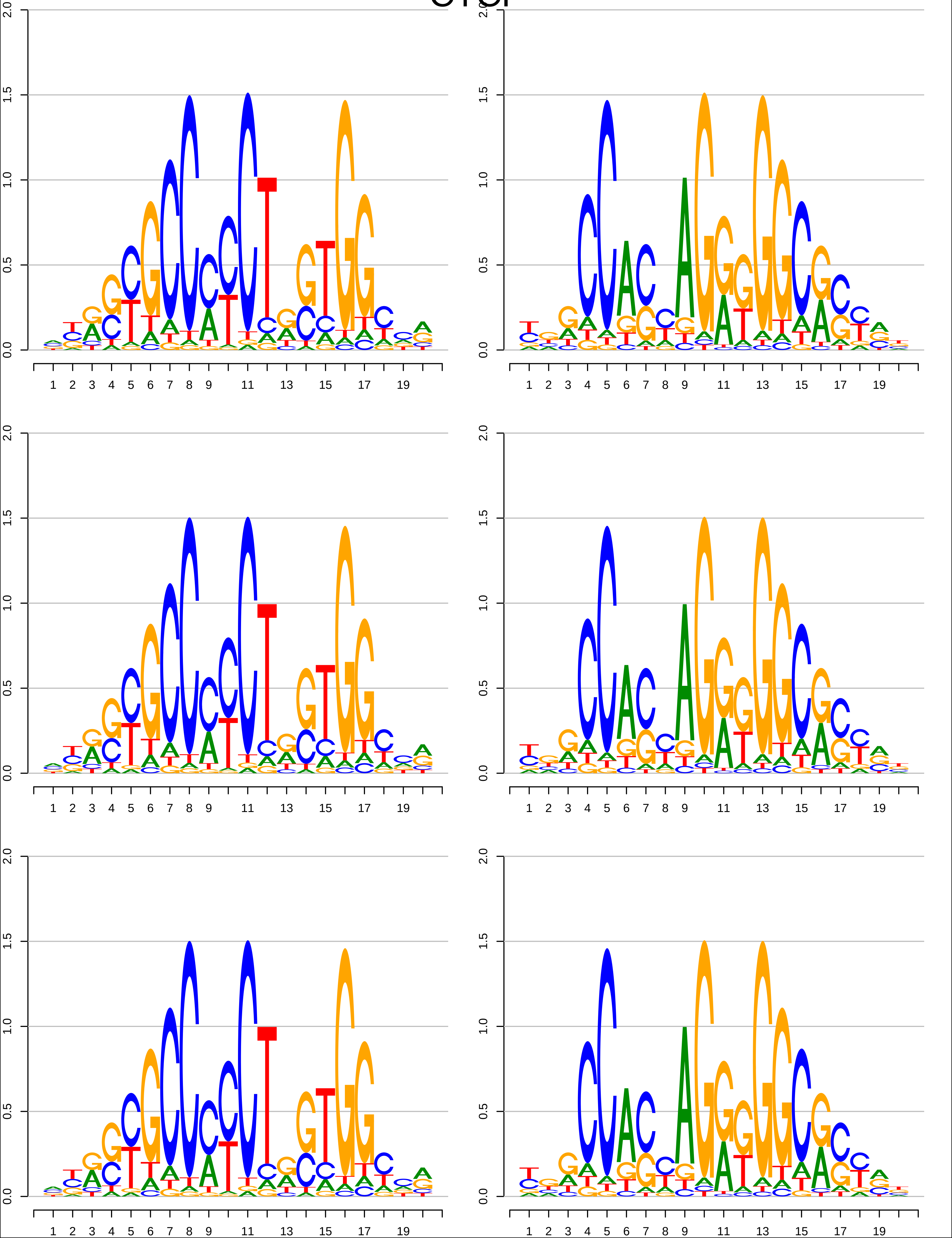

EGR1

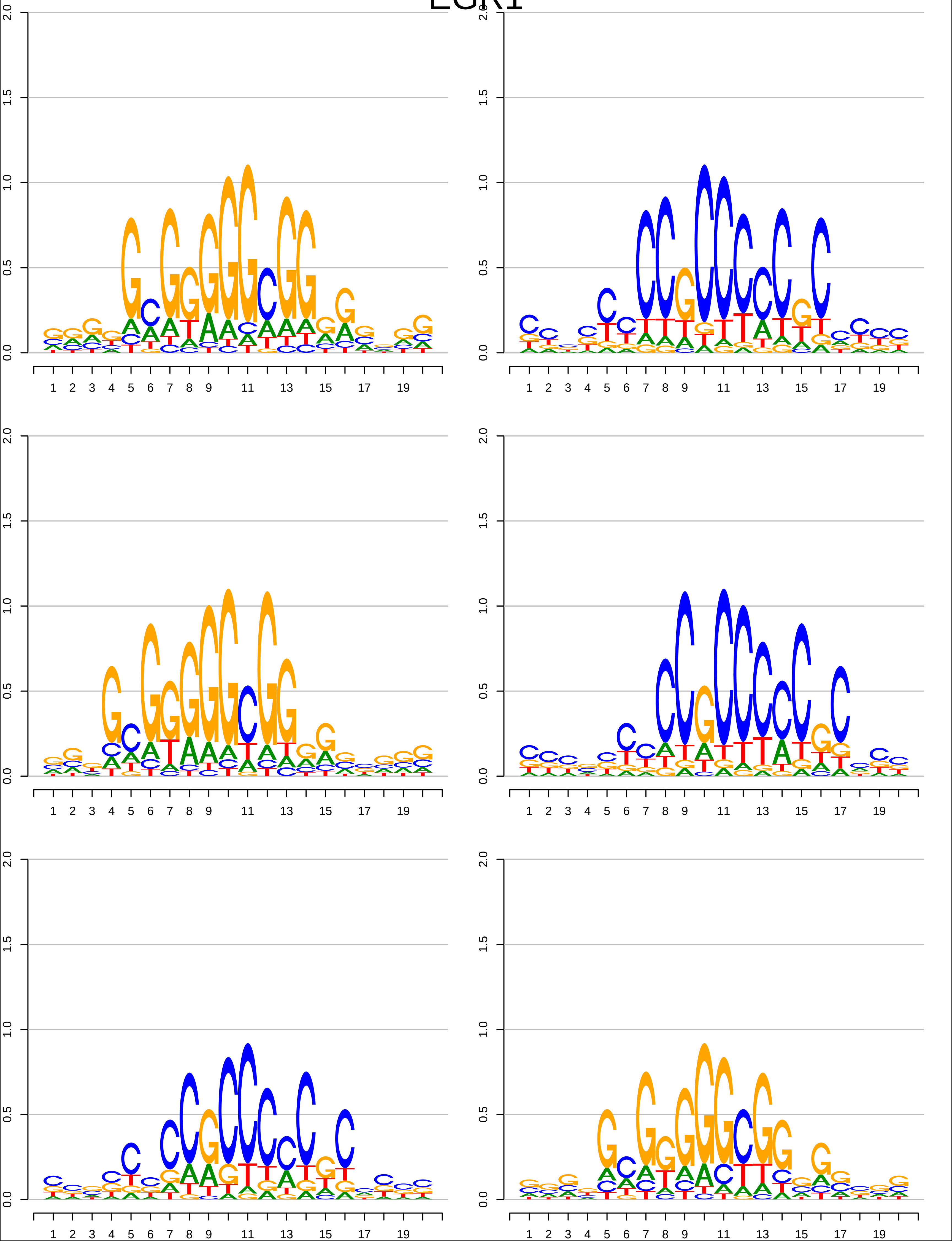

FOSL1

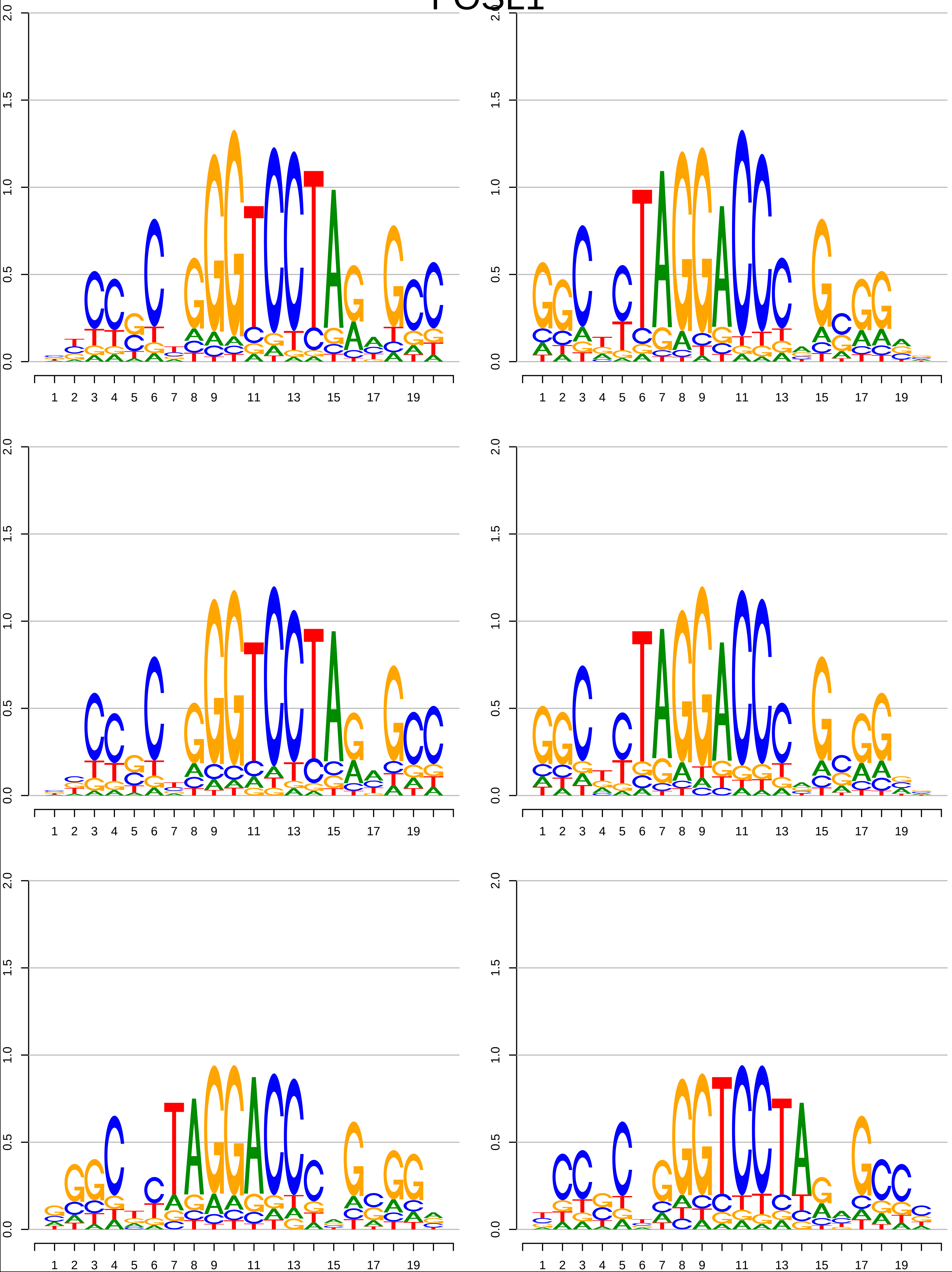

GABP

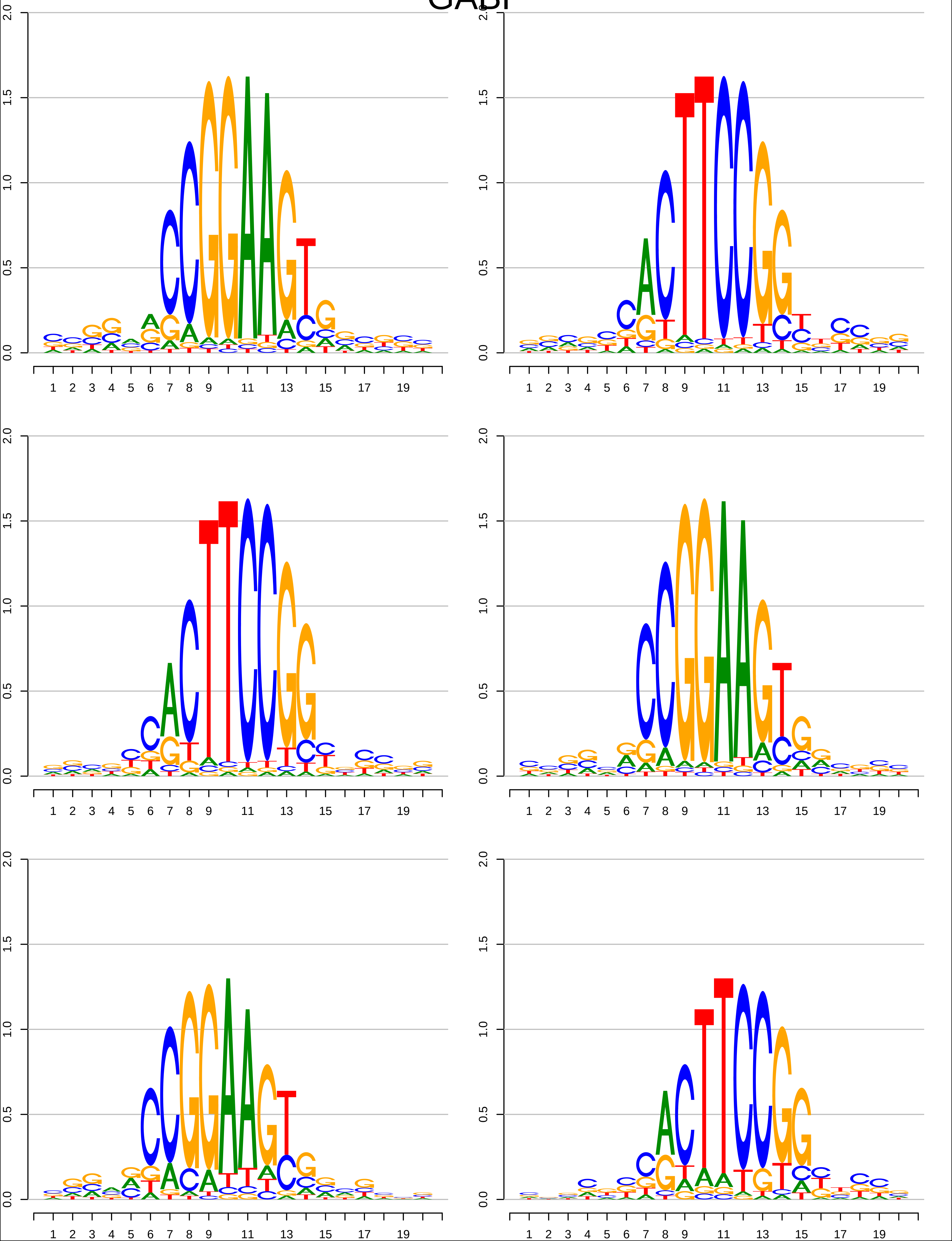

JARIDA1A

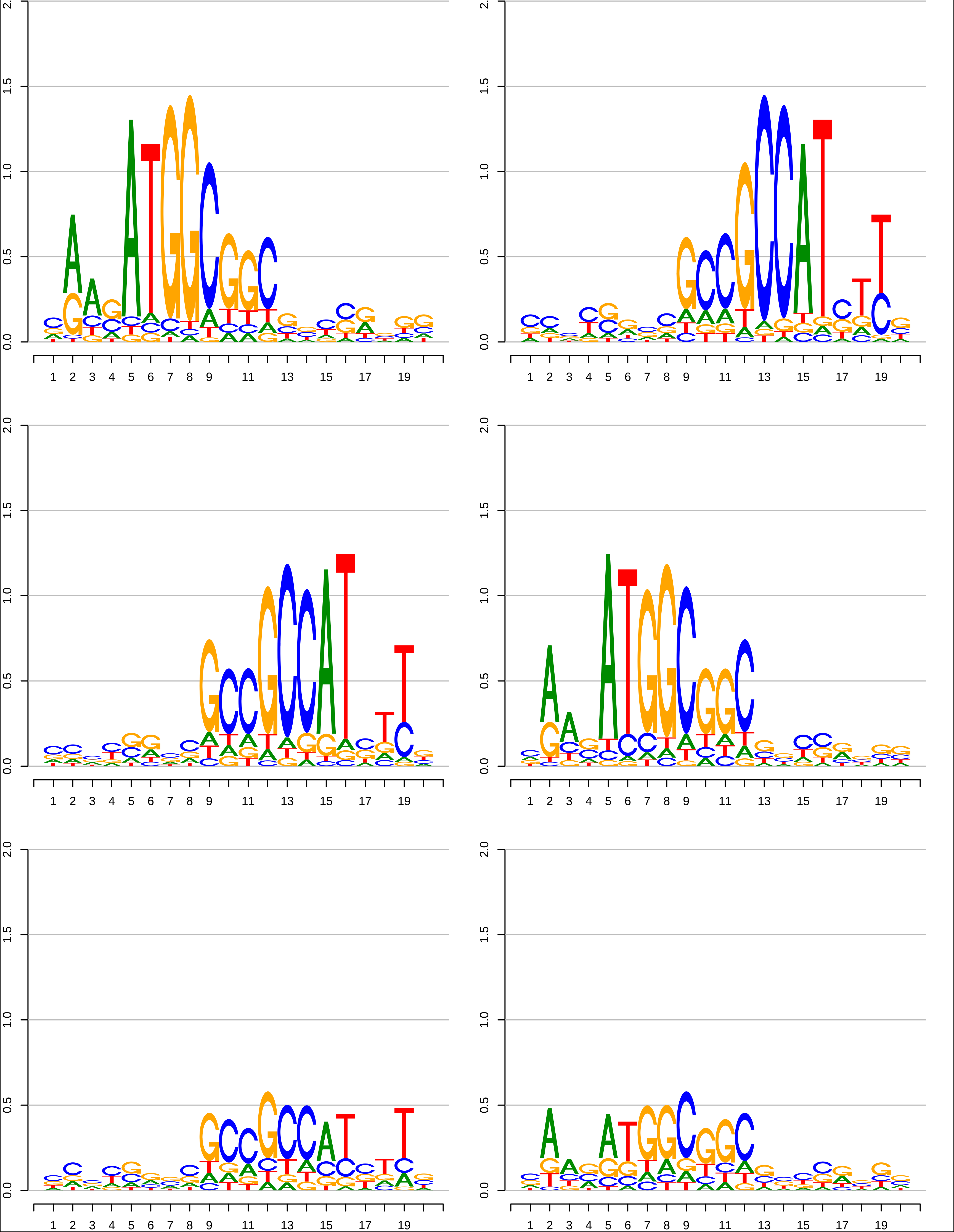

JunD

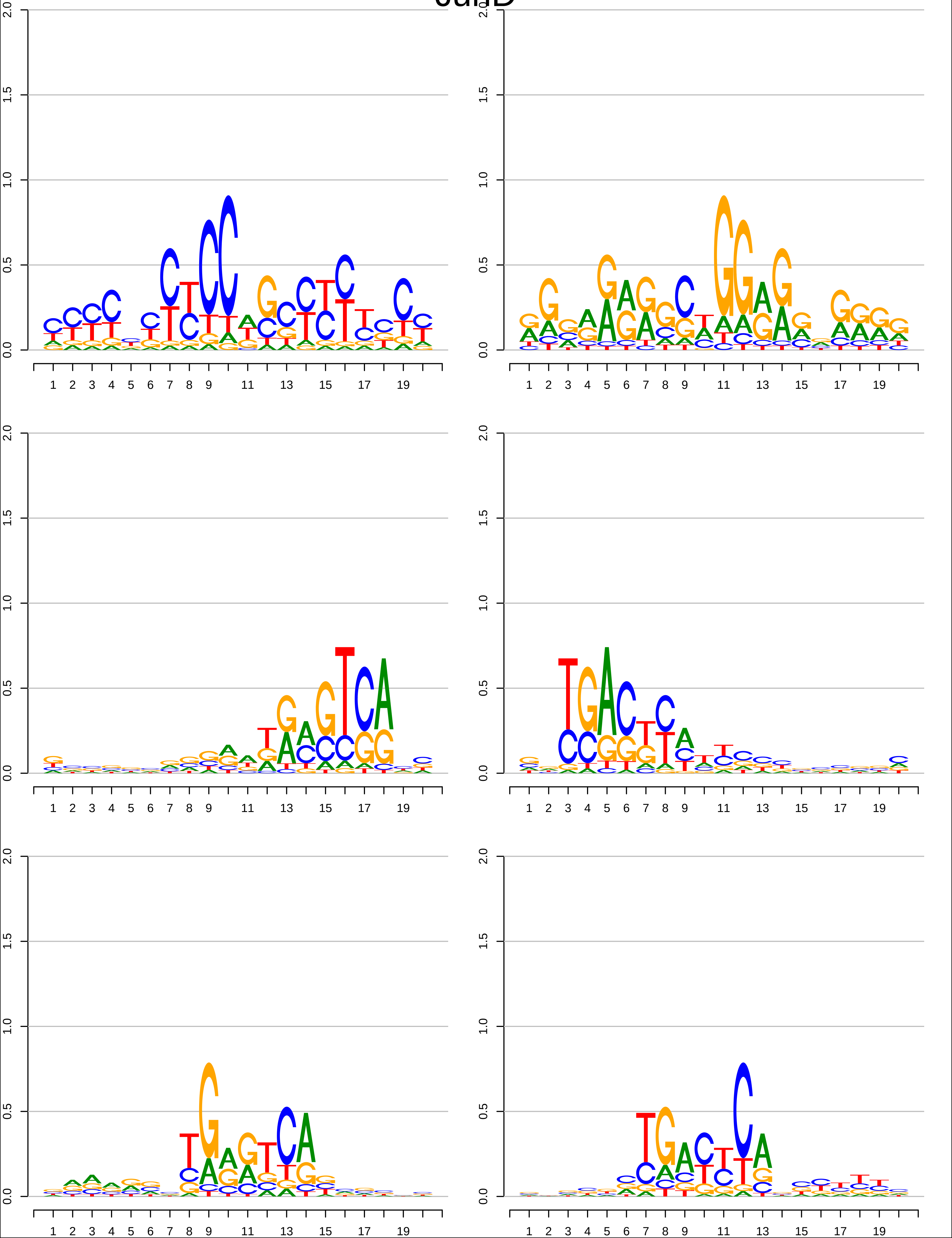

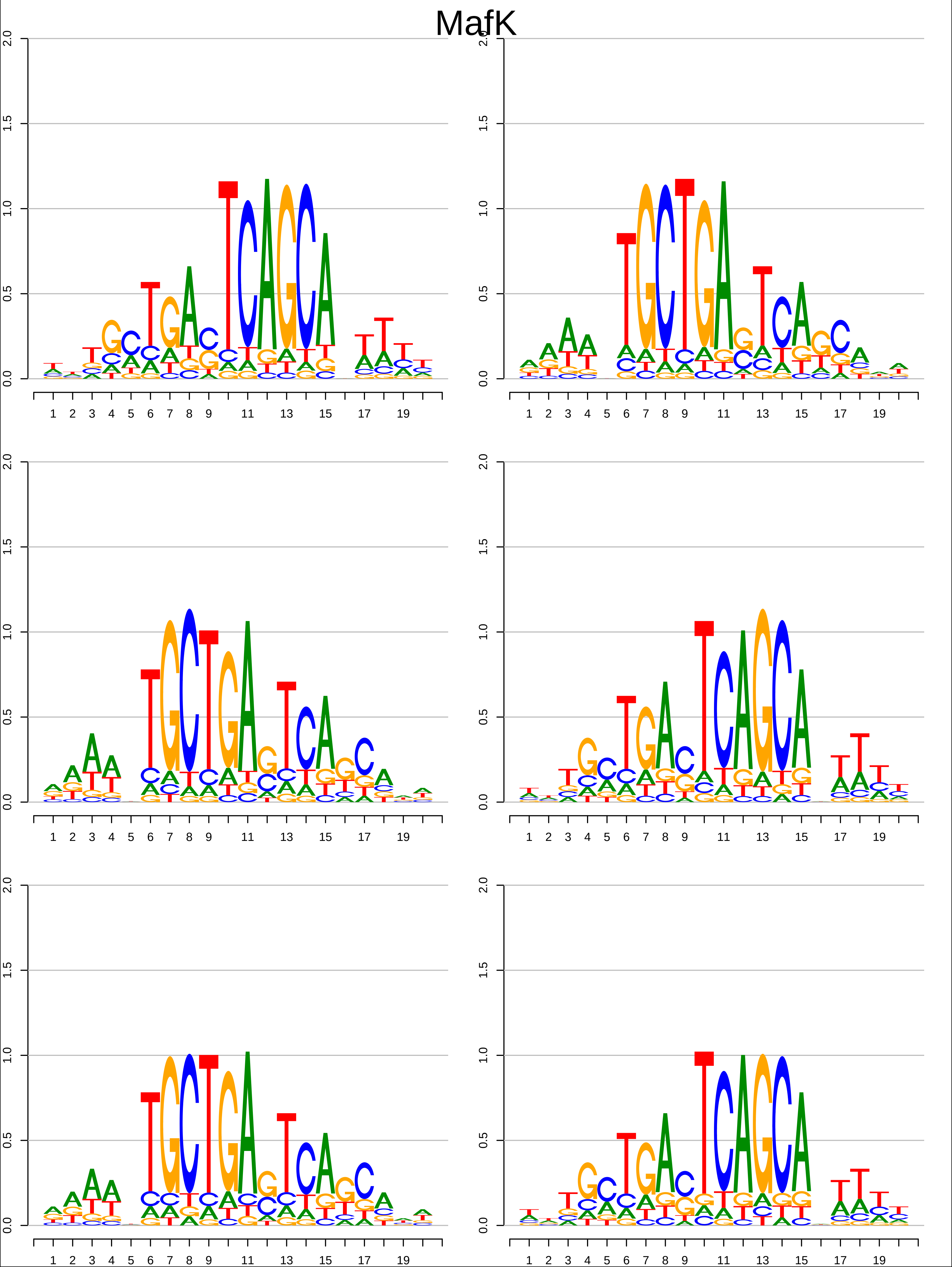
MafK

Max

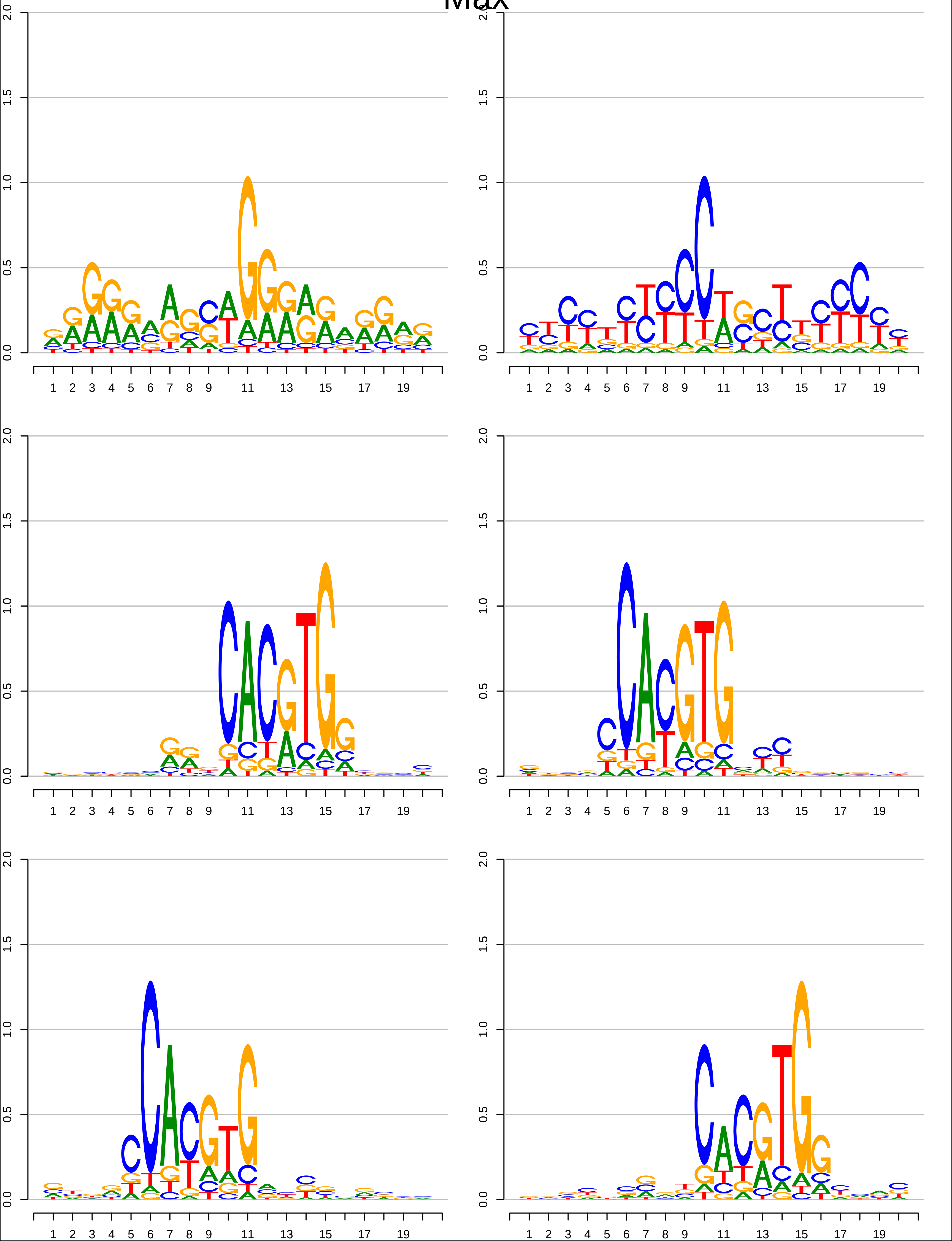

Mxi

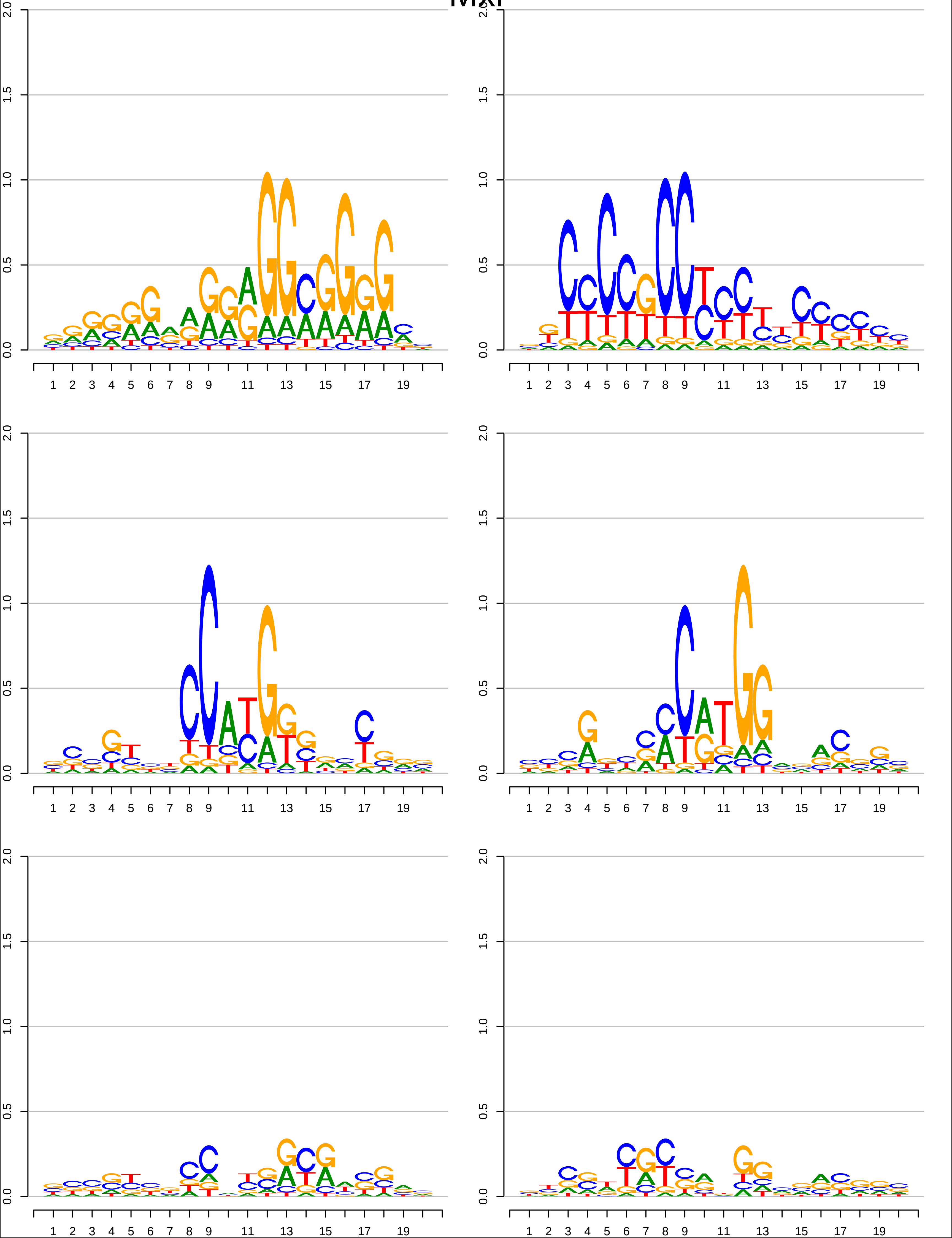

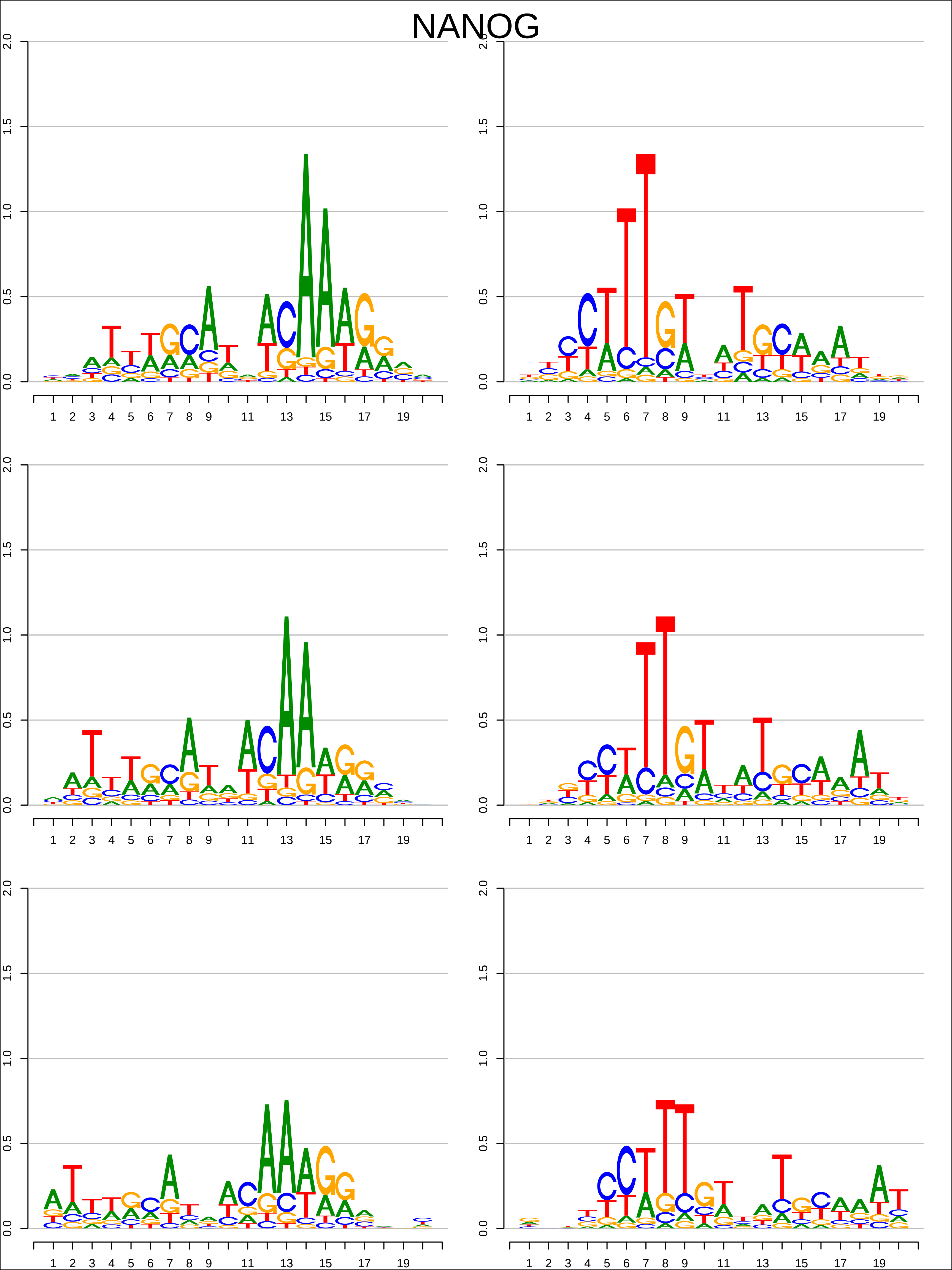
NANOG

Nrf

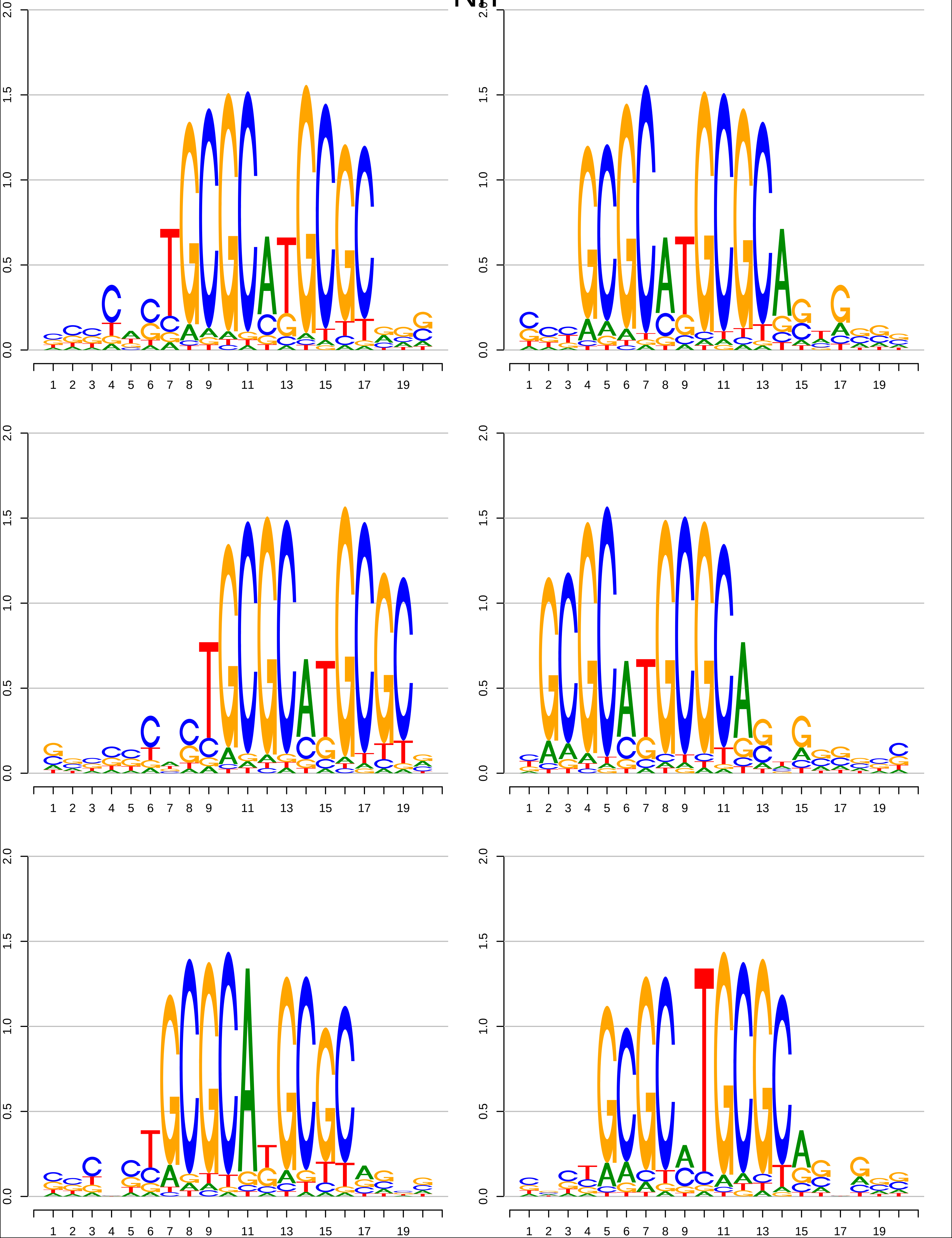

NRSF

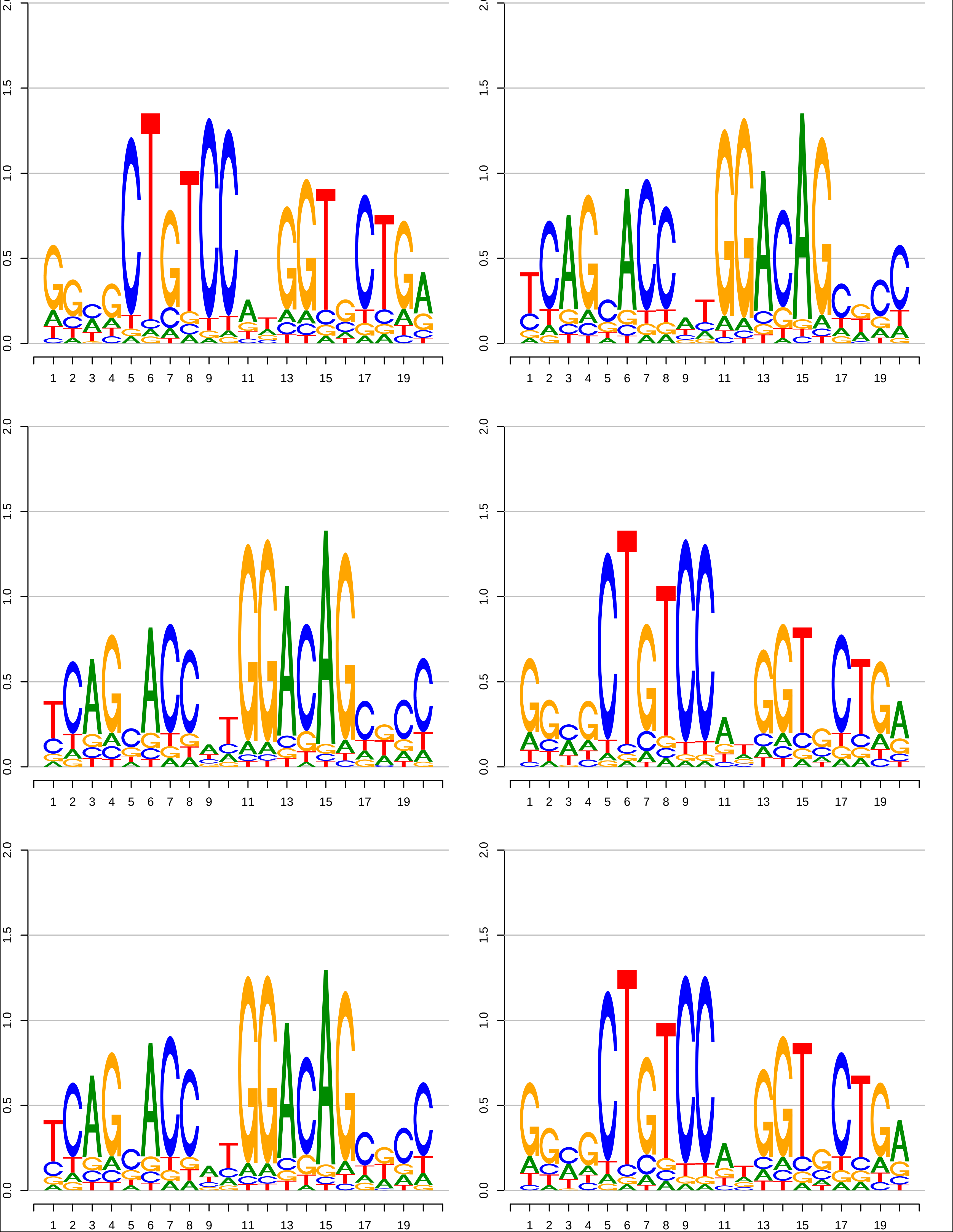

POU5F1

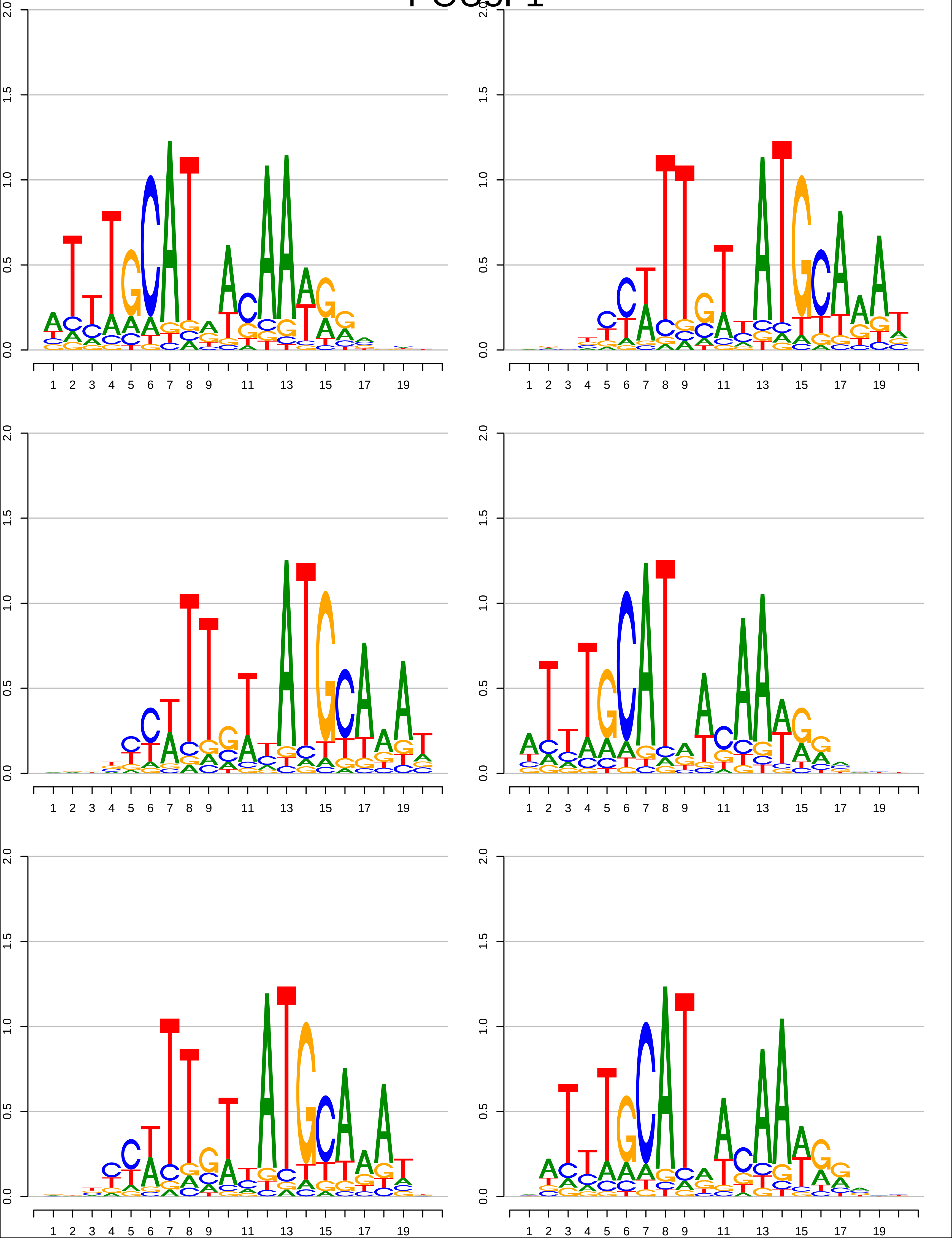

Rad21

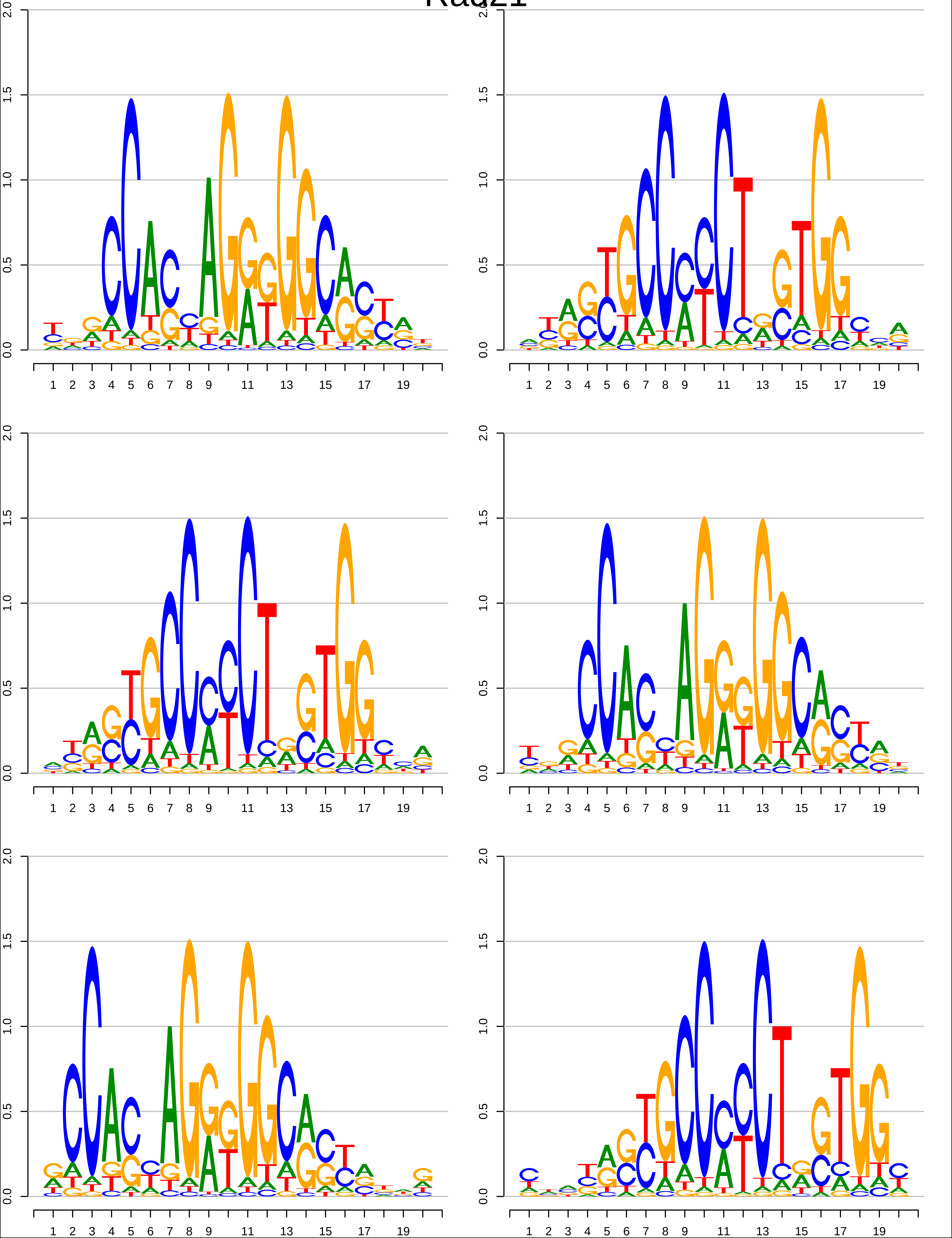

RFX5

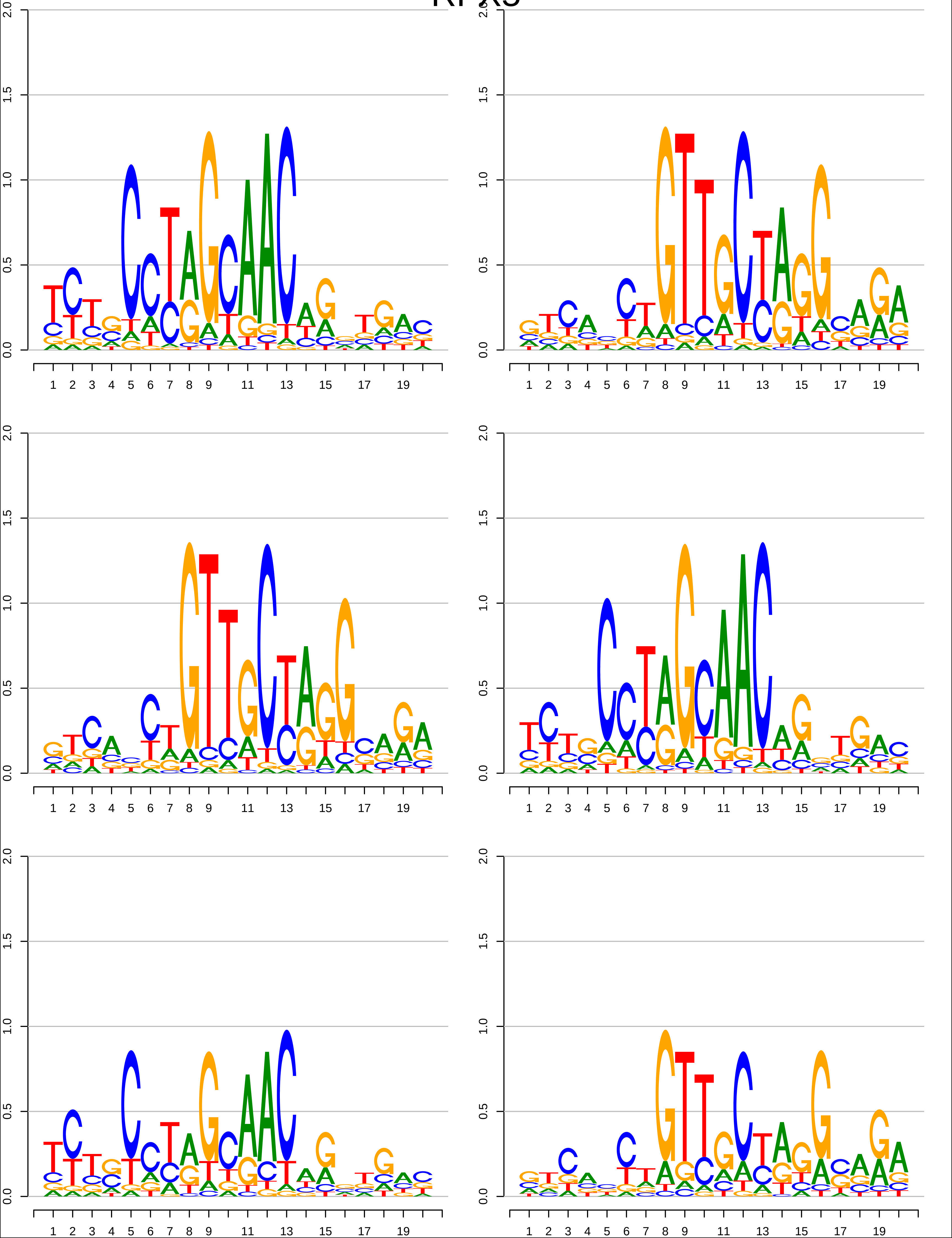

RXRA

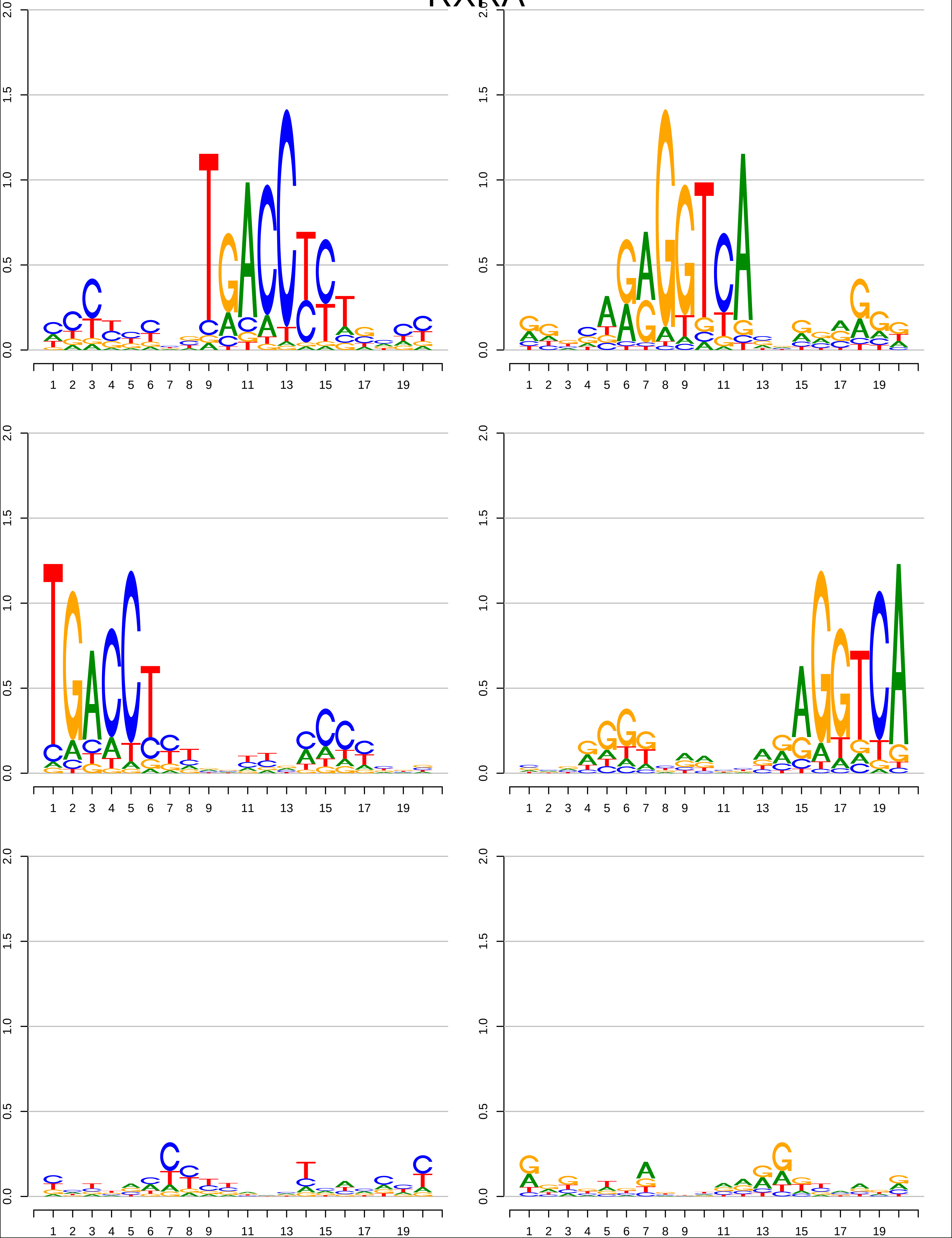

Sin3Ak

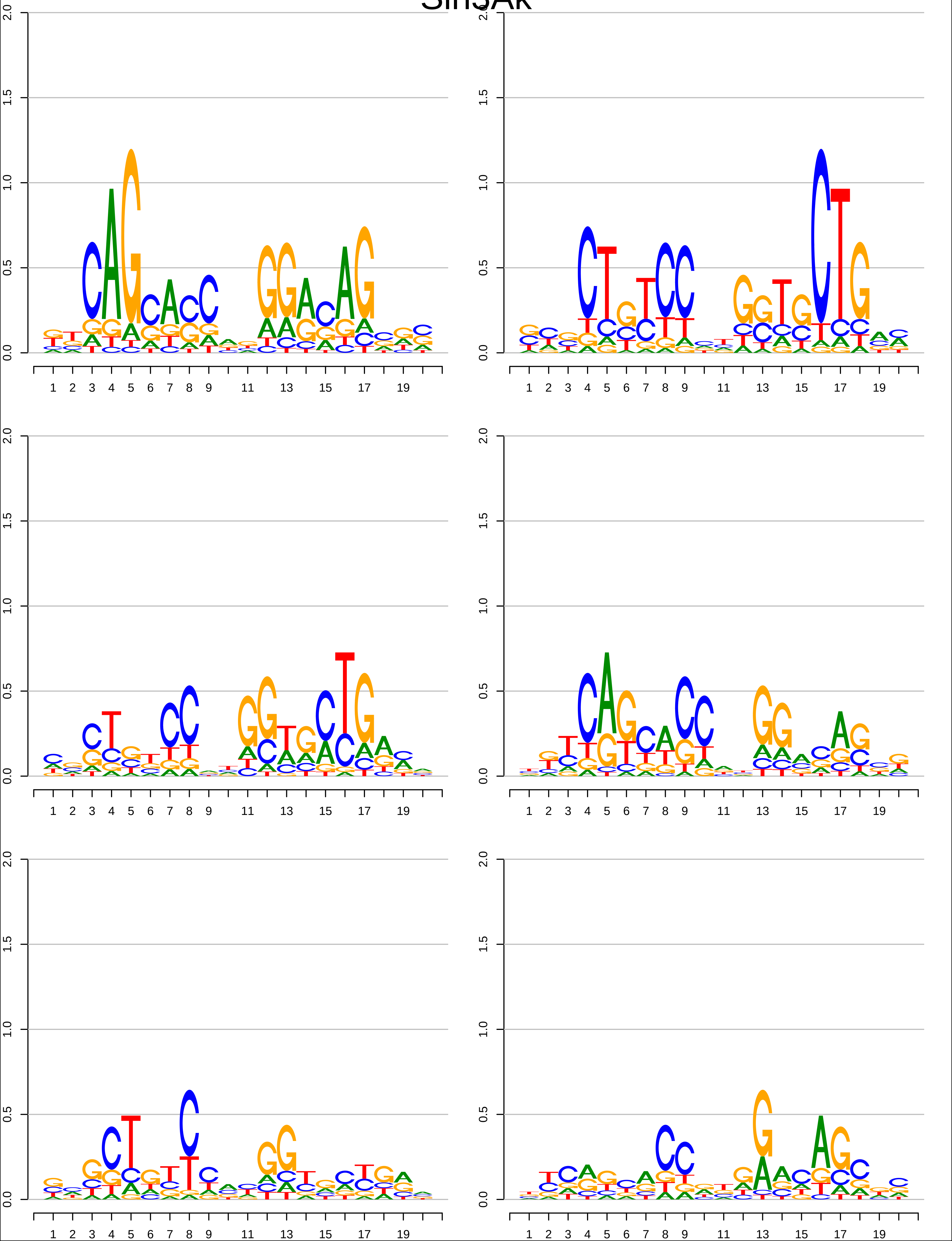

SIX5

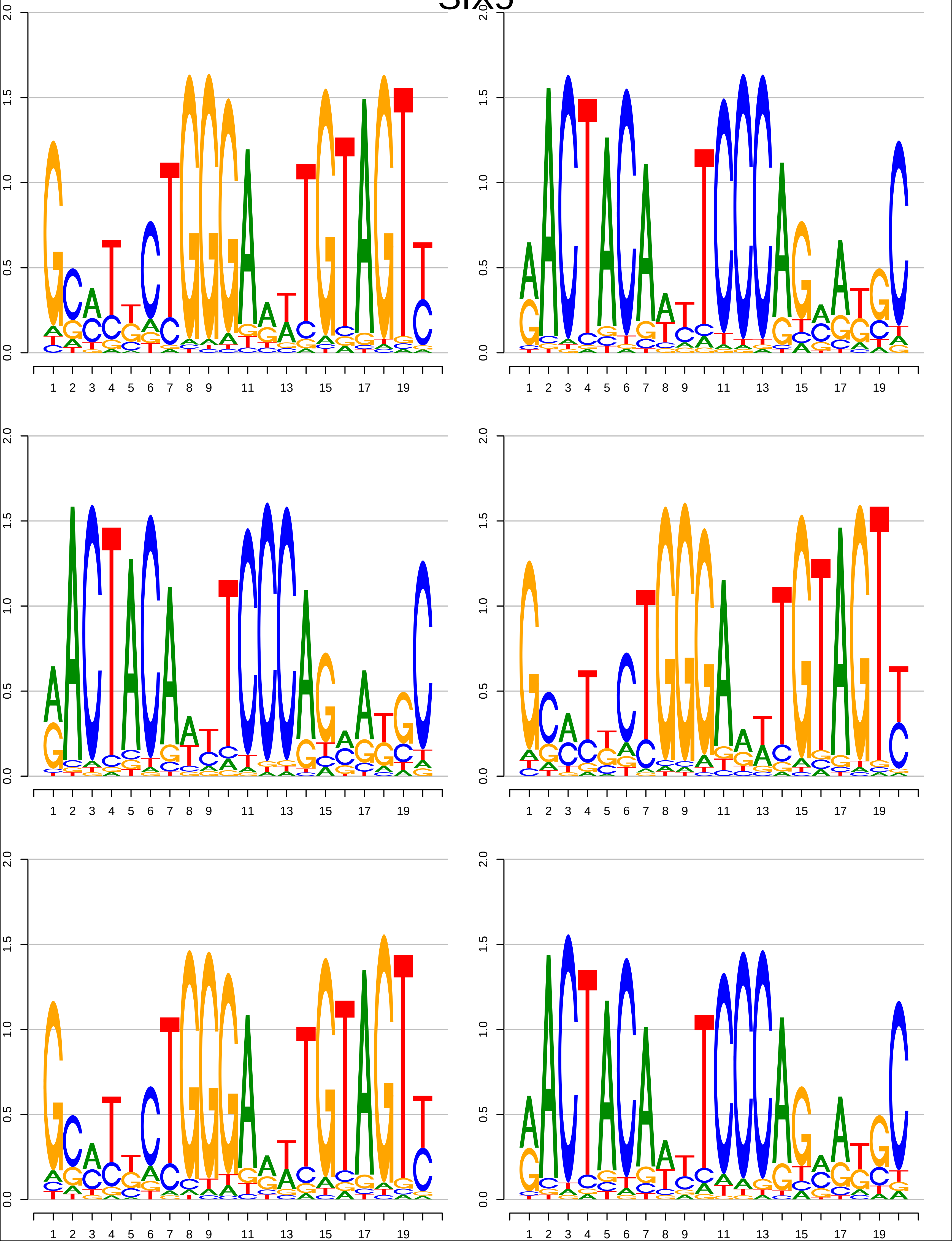

SP1

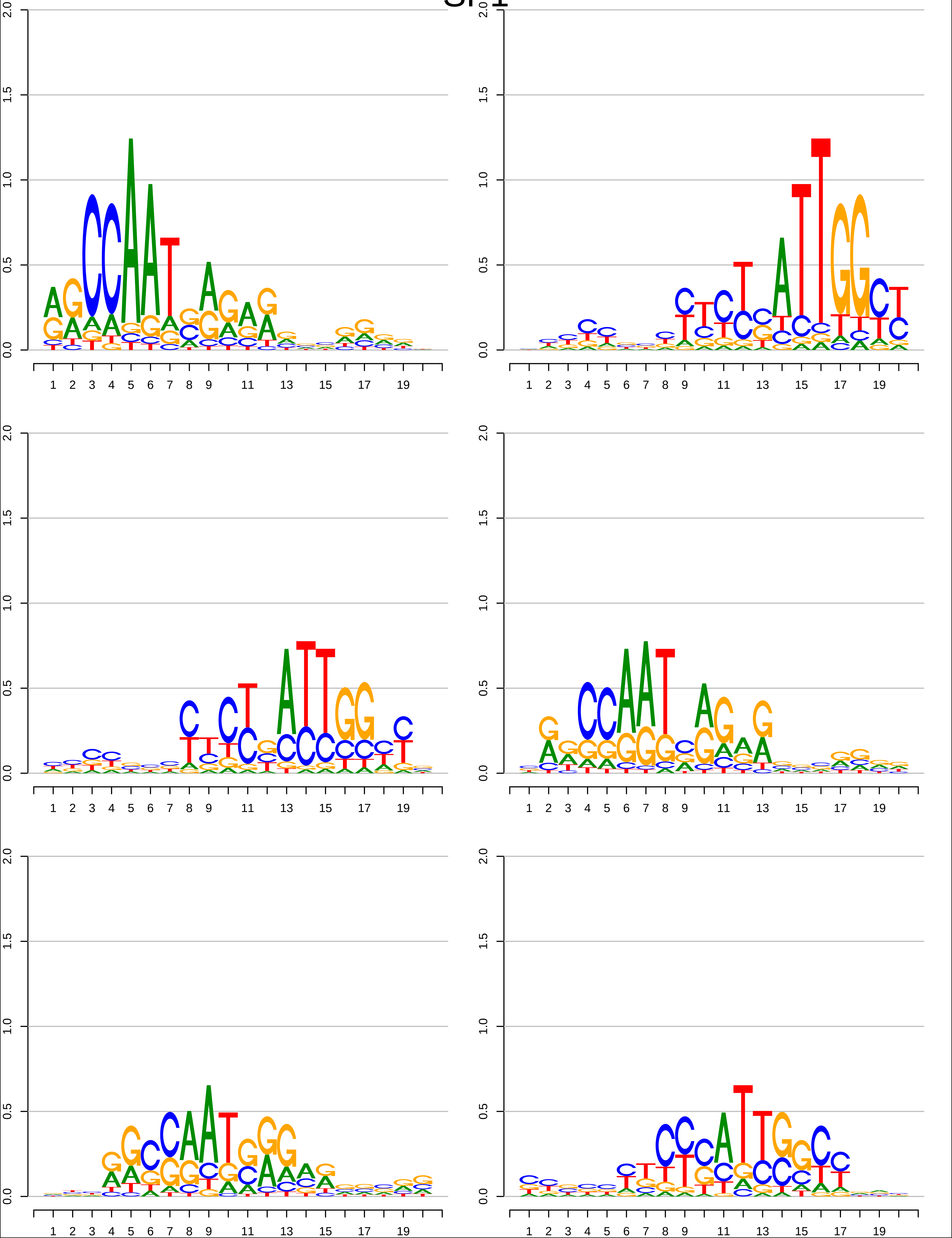

SP2

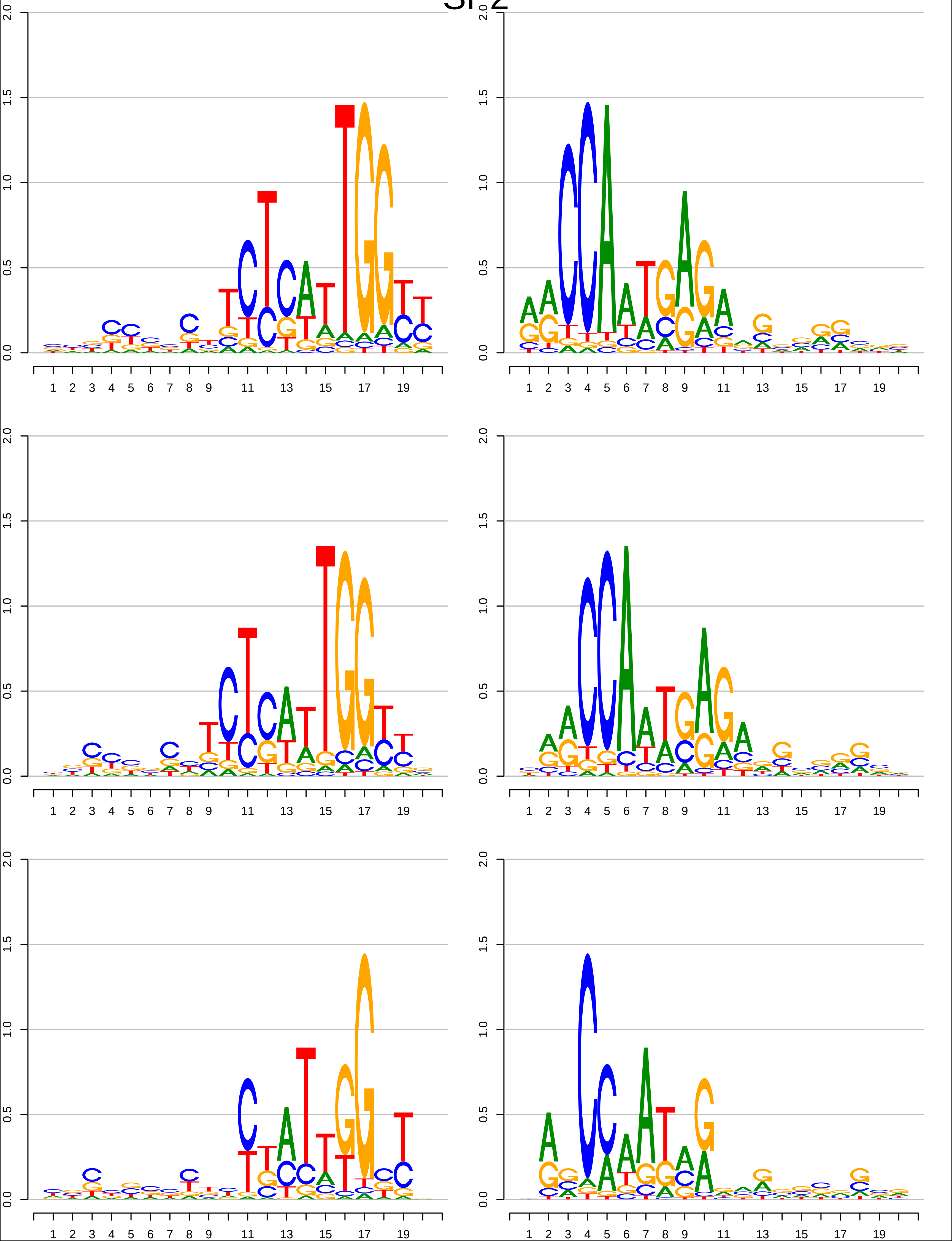

SP4

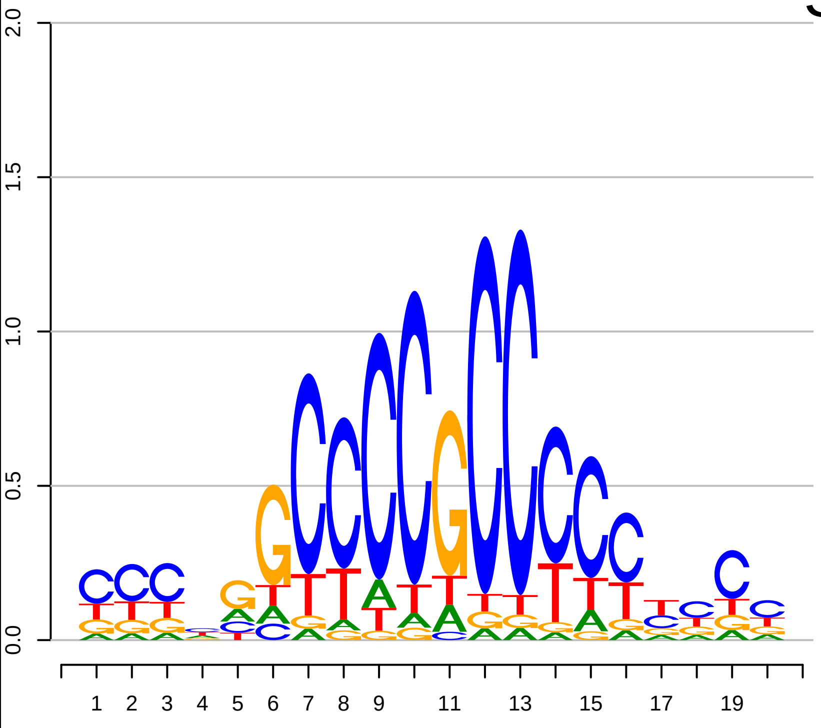

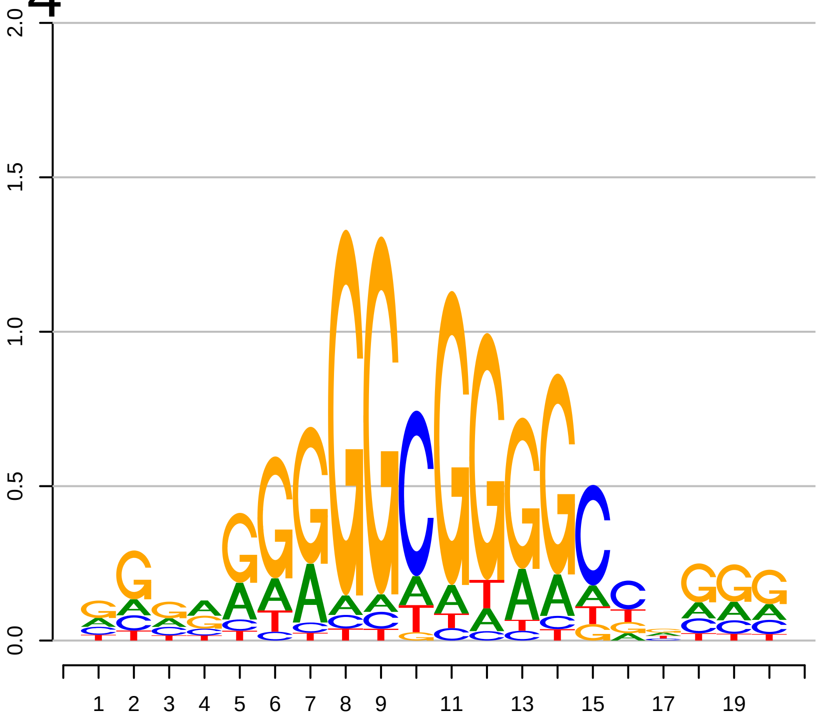

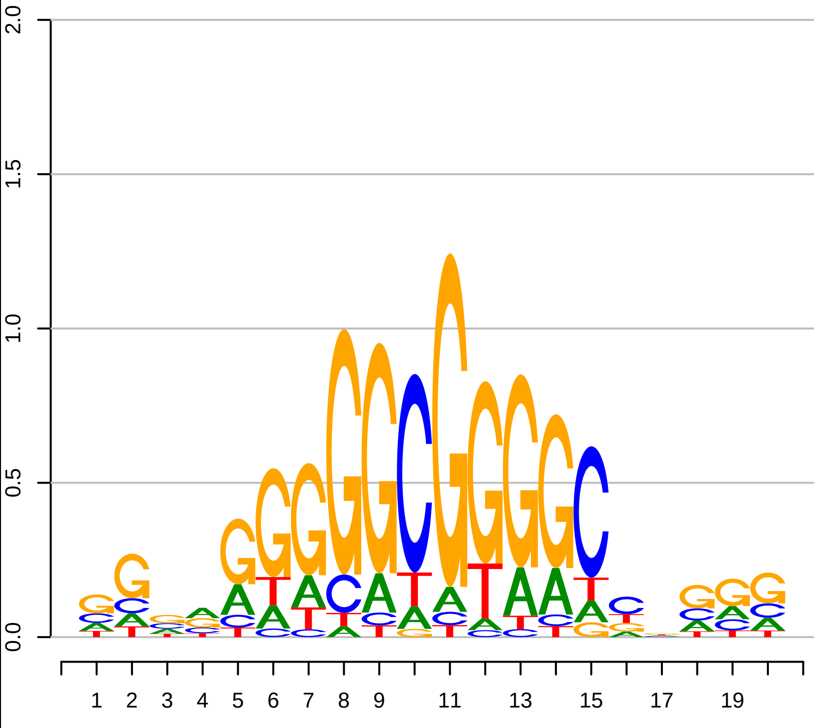

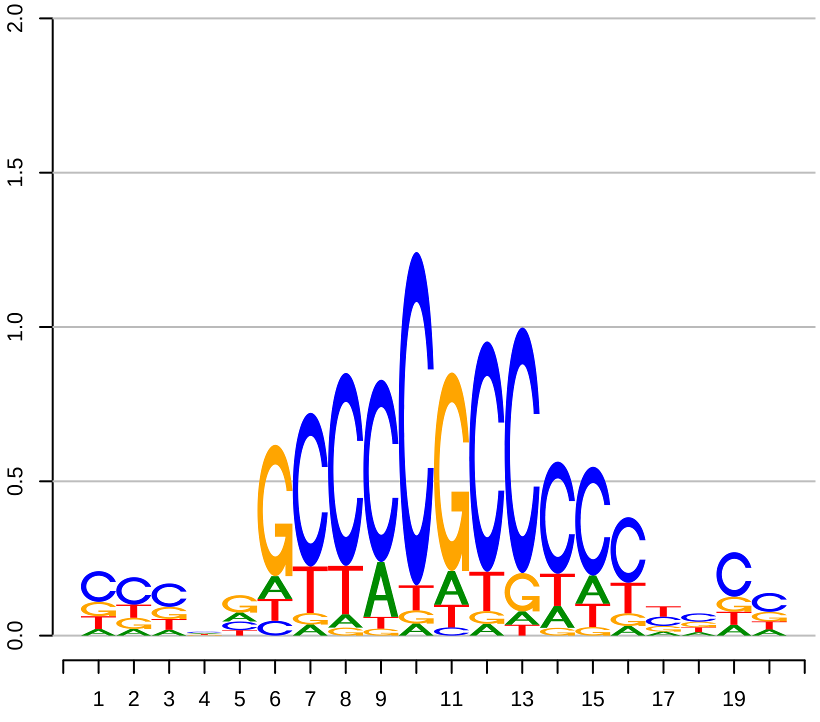

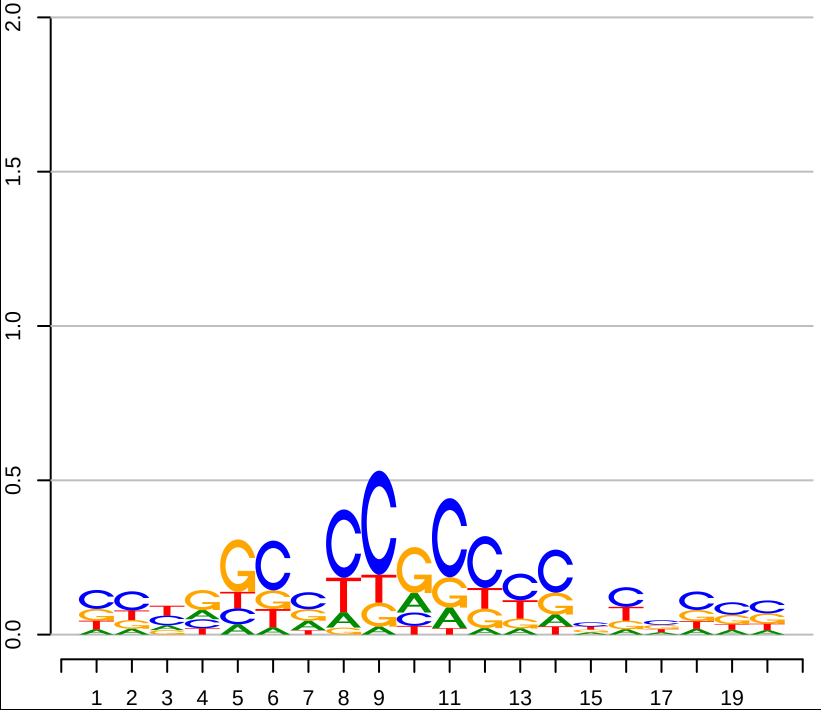

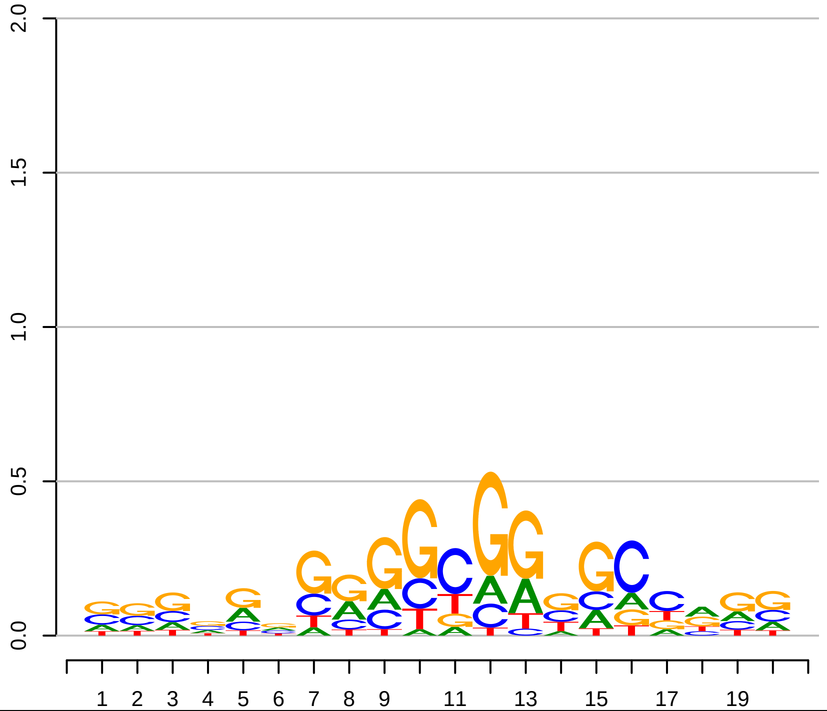

SRF

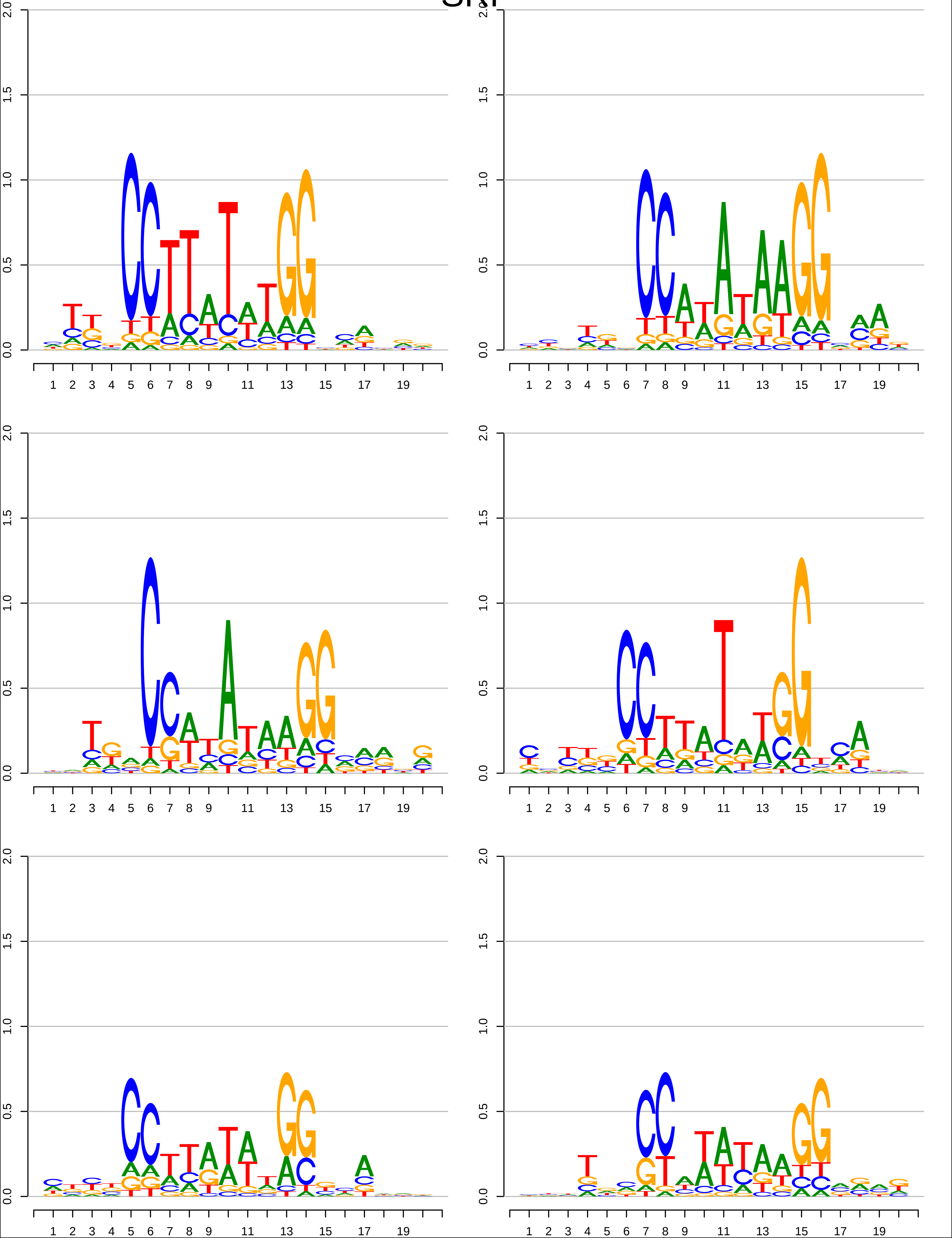

TCF12

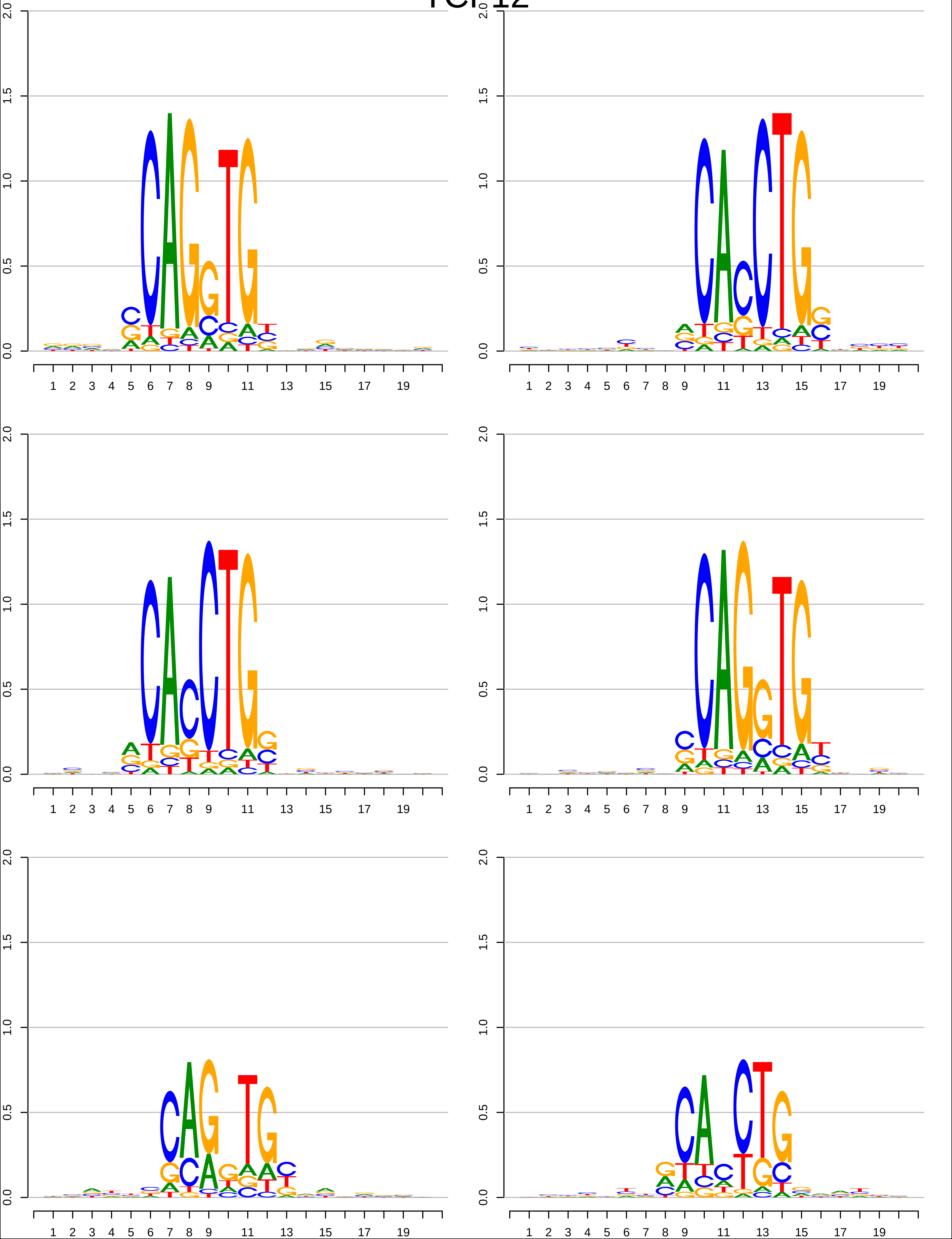

TEAD4

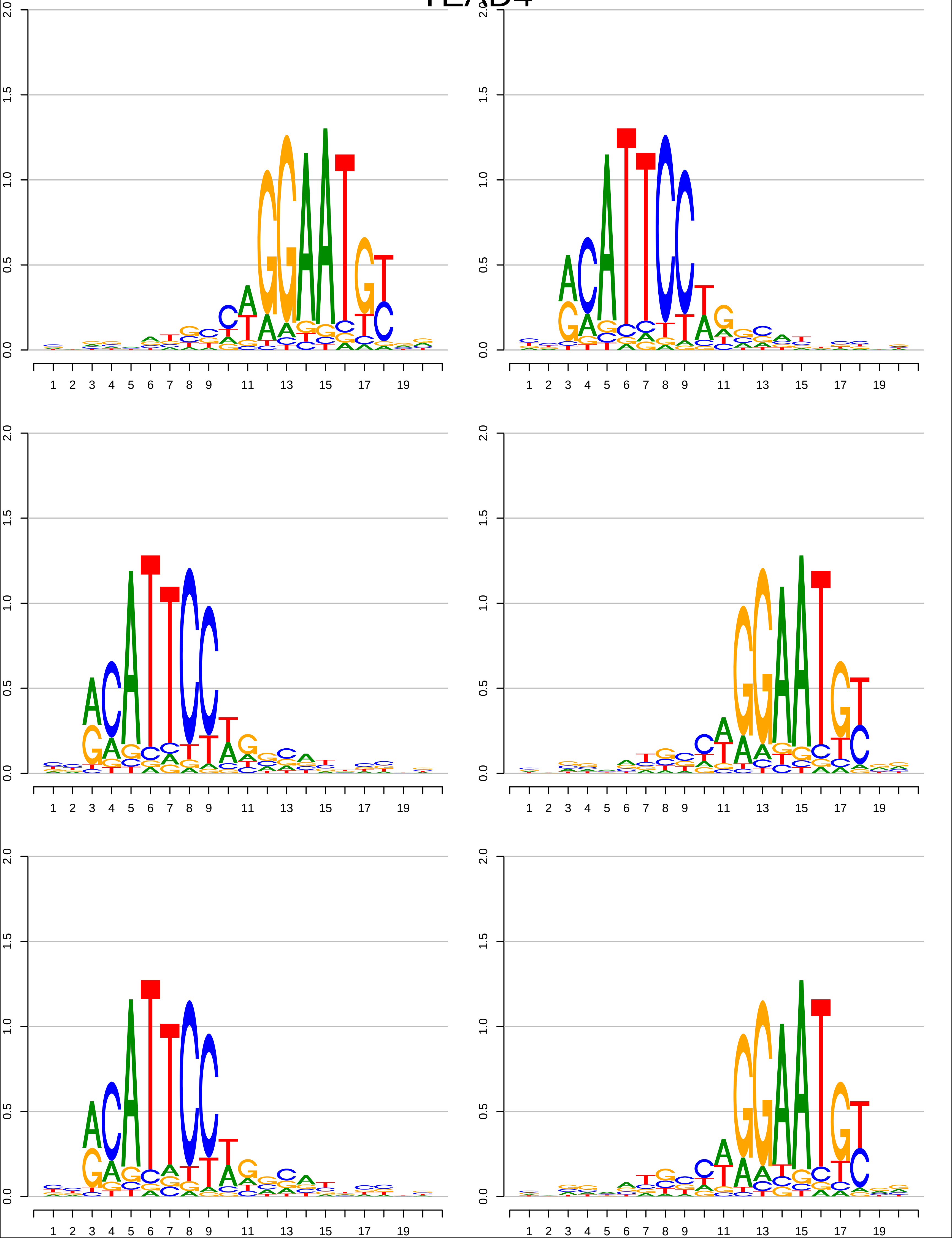

USF1

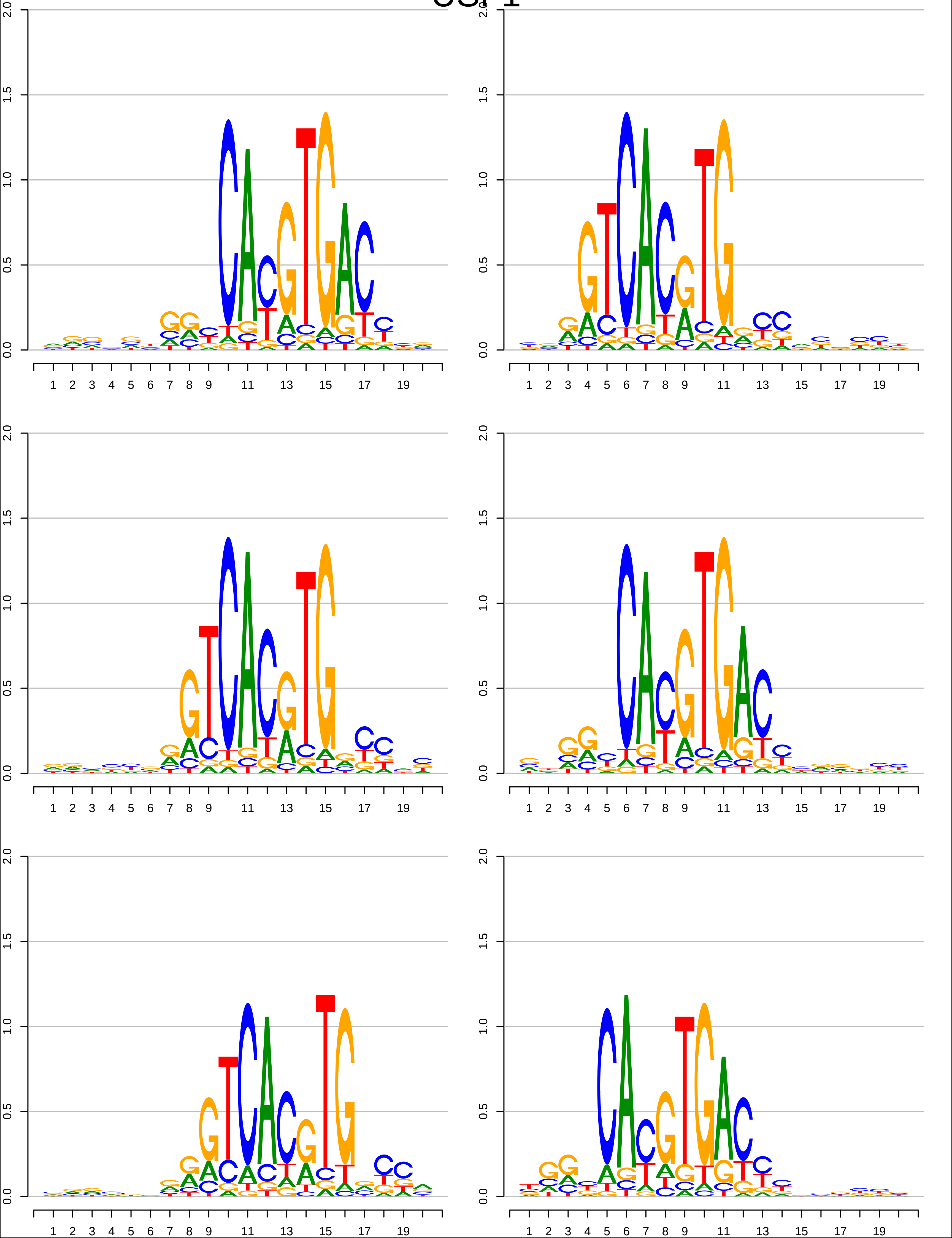

USF2

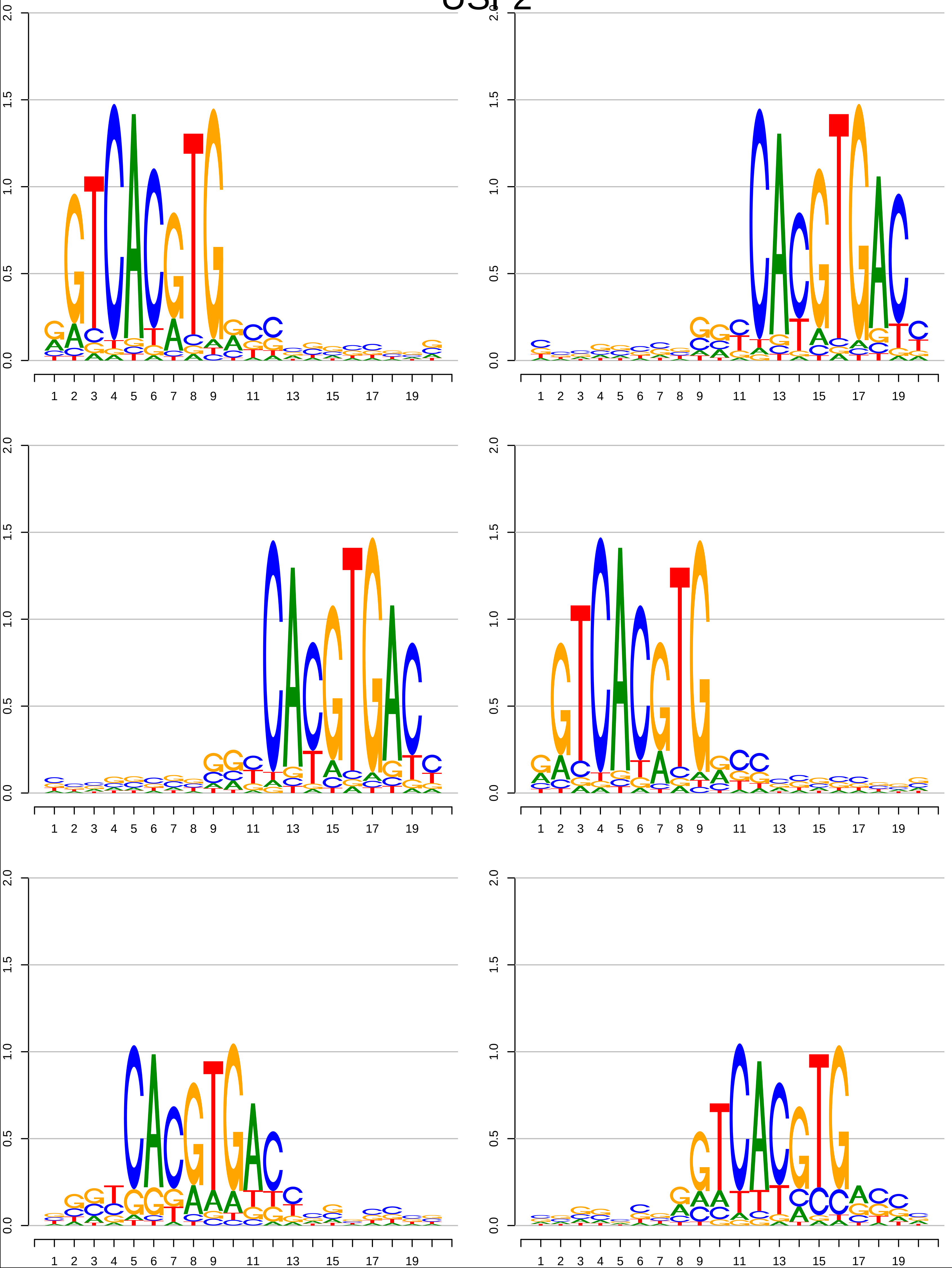

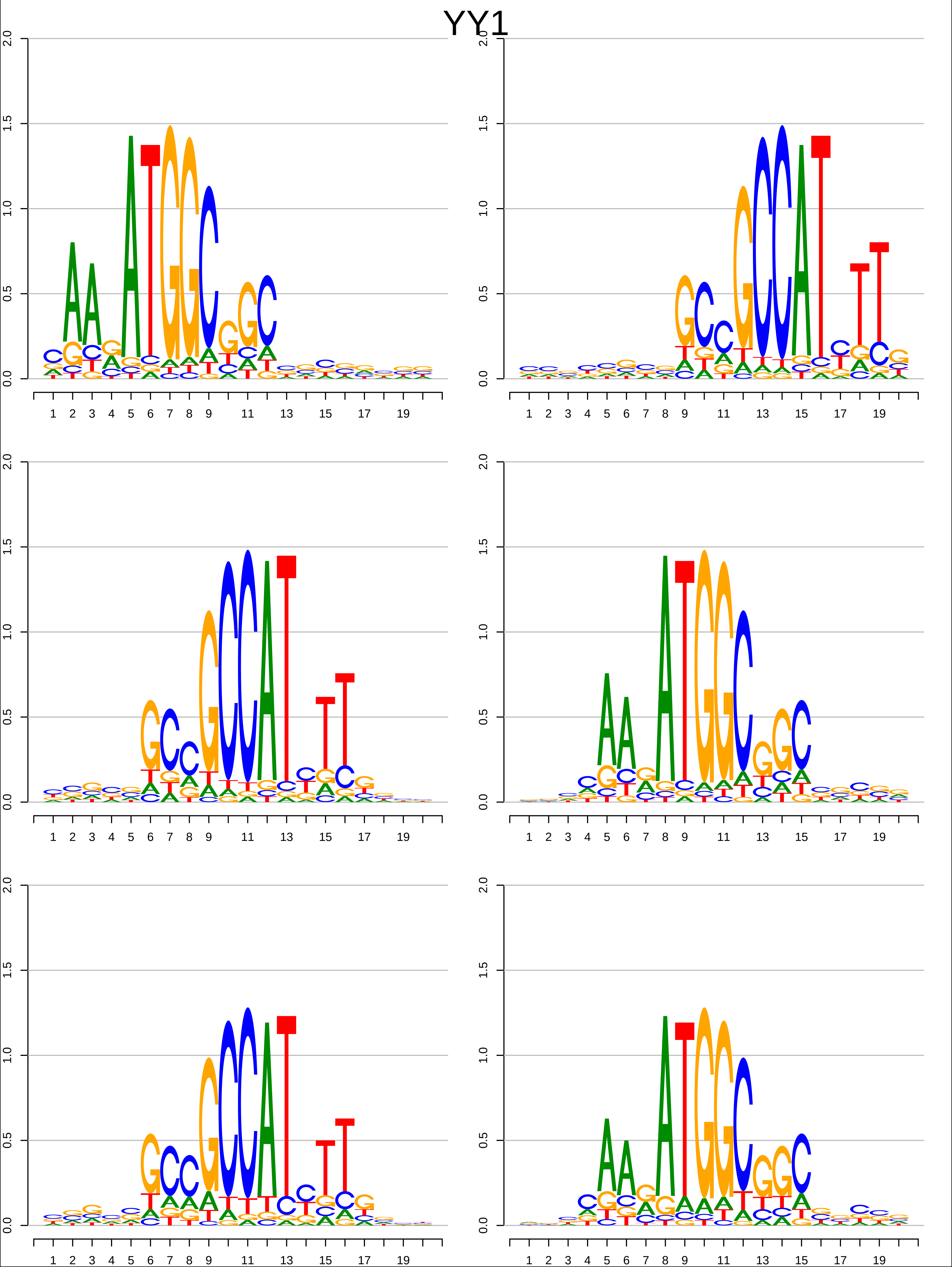
YY1

Znf143

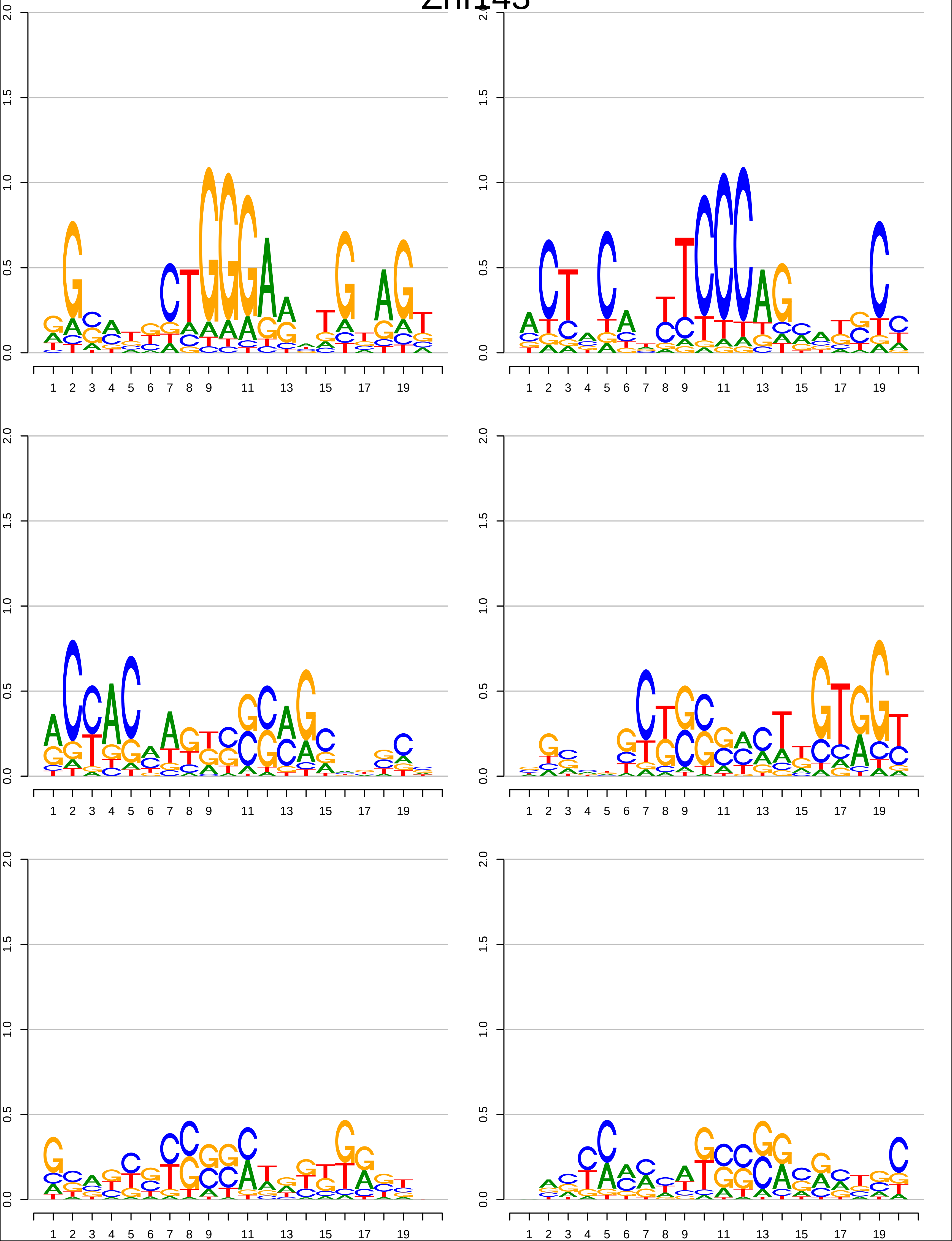

Supplement: Supplementary file 5 — Sequence logos of predicted binding sites. The file contains sequence logos and their reverse complements of predicted binding sites inferred using the PFM(0), the PFM(1), and the PFM(2) for each of the 35 TFs. (PDF 11776 kb) [file 12859_2017_1495_MOESM5_ESM.pdf]
