# Supplementary material for: Combining phylogenetic footprinting with motif models incorporating intra-motif dependencies
Source: BMC Bioinformatics. 2017 Mar 1;18:141. doi: 10.1186/s12859-017-1495-1 (PMC5333389; doi:10.1186/s12859-017-1495-1)
Supplement: Supplementary file 6 — ROC curves. The pdf file comprises for each TF one plot that shows the 25 ROC curves and one plot that shows the 25 PR curves from the 25–fold stratified repeated random sub-sampling validation procedure described in Methods 3. (PDF 2611.2 kb) [file 12859_2017_1495_MOESM6_ESM.pdf]

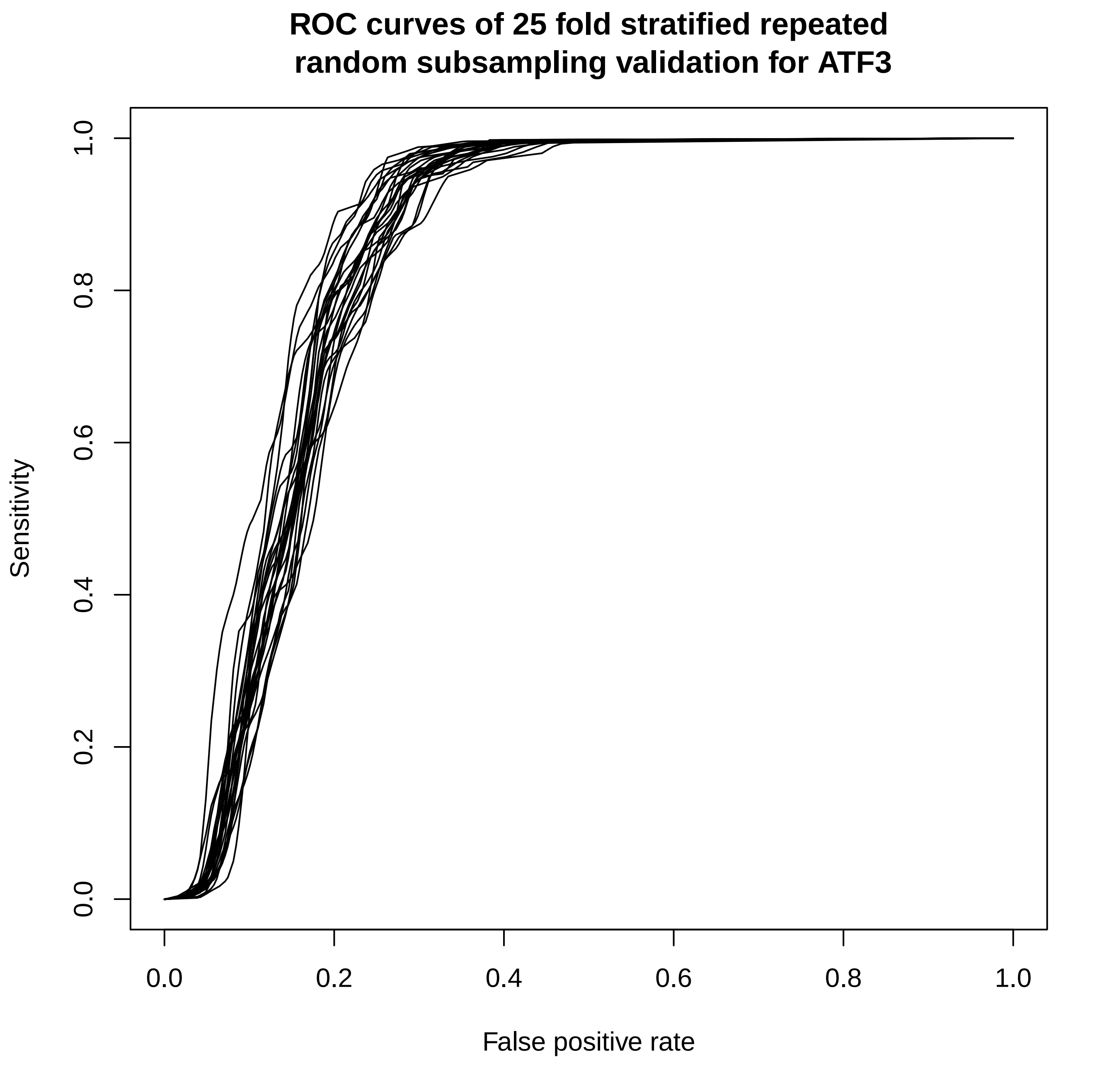
ROC curves of 25 fold stratified repeated
random subsampling validation for ATF3
Sensitivity
0.0
0.2
0.4
0.6
0.8
1.0
False positive rate
0.0
0.2
0.4
0.6
0.8
1.0

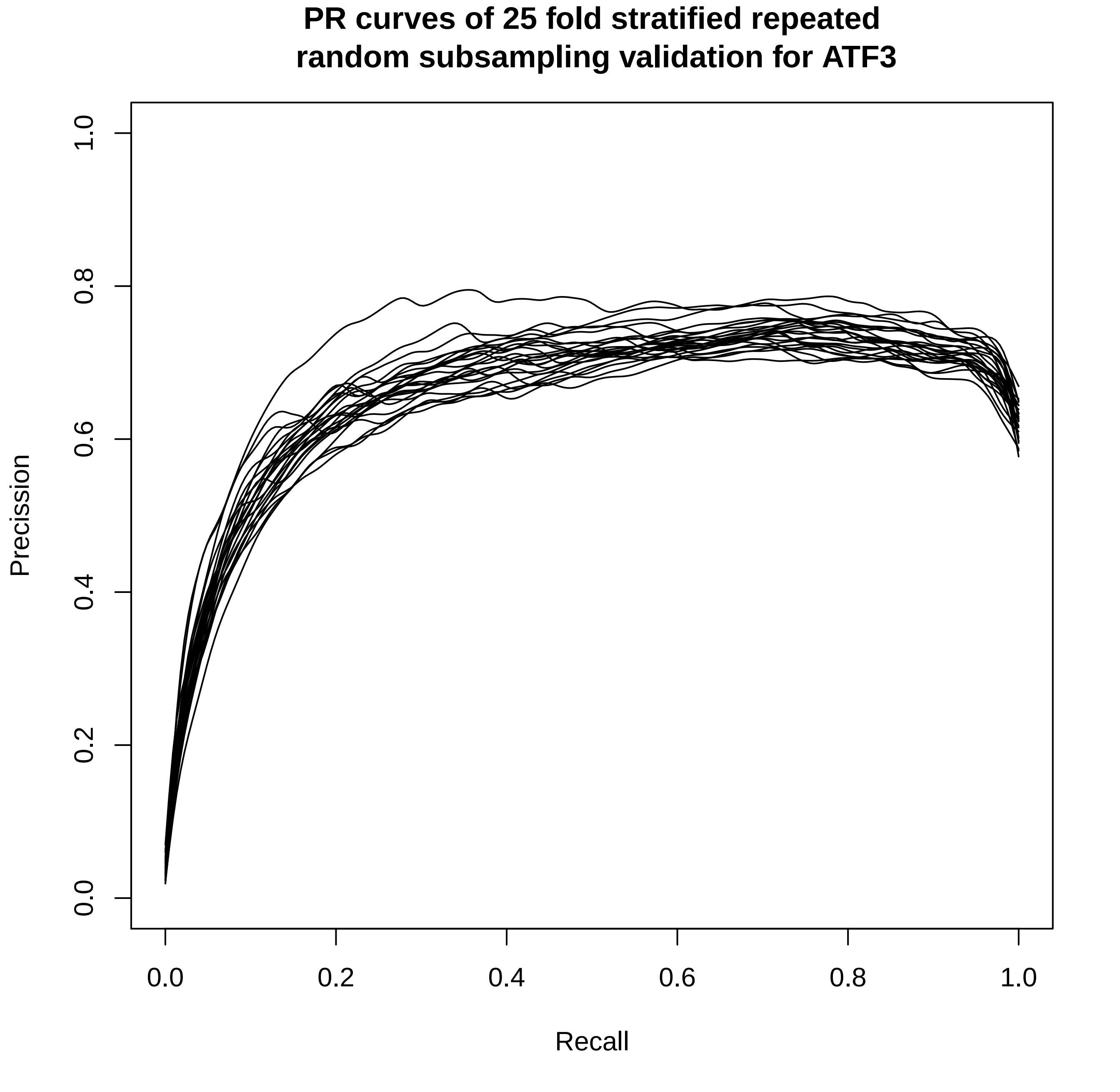
PR curves of 25 fold stratified repeated
random subsampling validation for ATF3
Precission
Recall
0.0
0.2
0.4
0.6
0.8
1.0
0.0
0.2
0.4
0.6
0.8
1.0

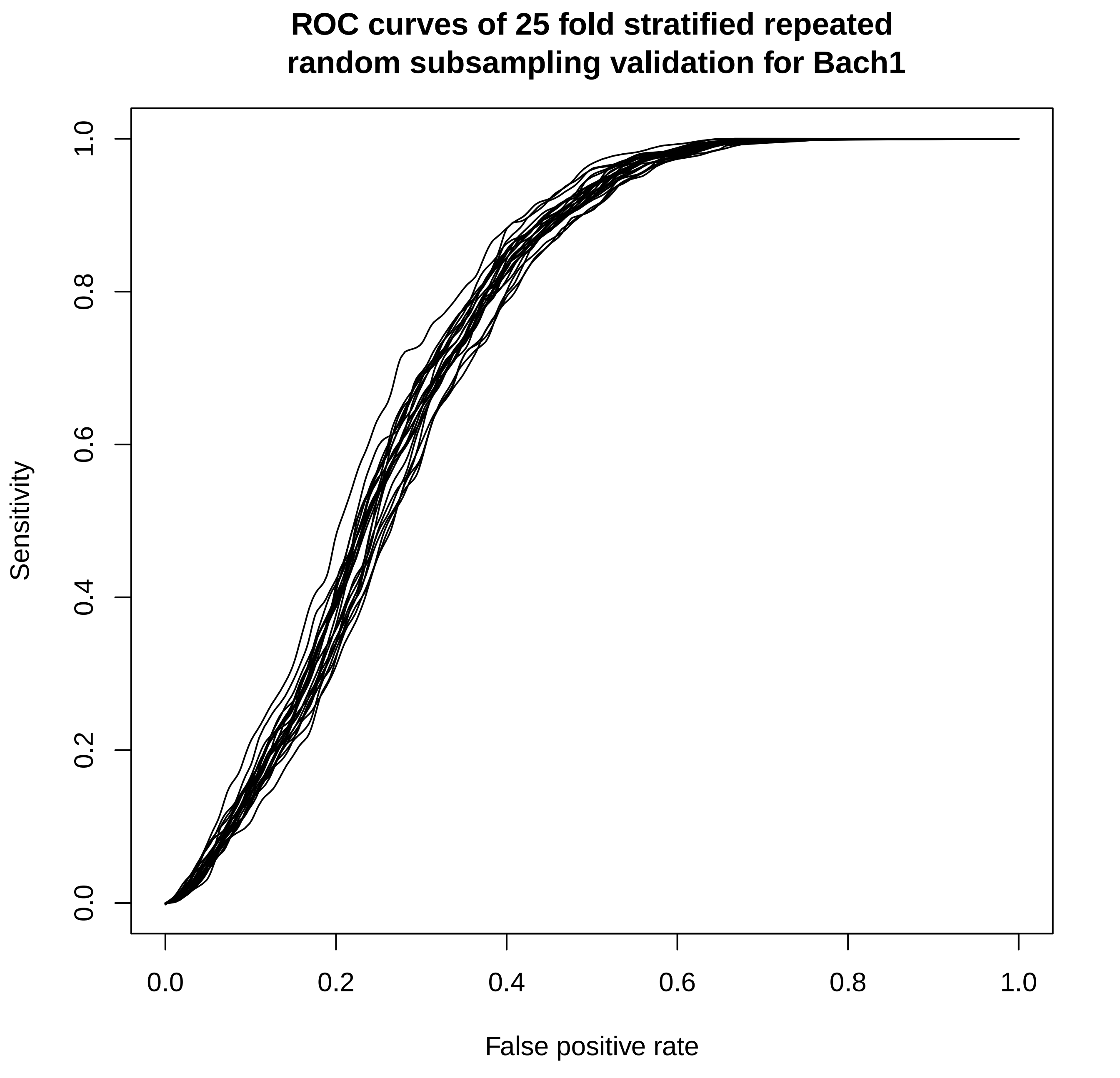

ROC curves of 25 fold stratified repeated random subsampling validation for Bach1
Sensitivity
False positive rate
0.0
0.2
0.4
0.6
0.8
1.0

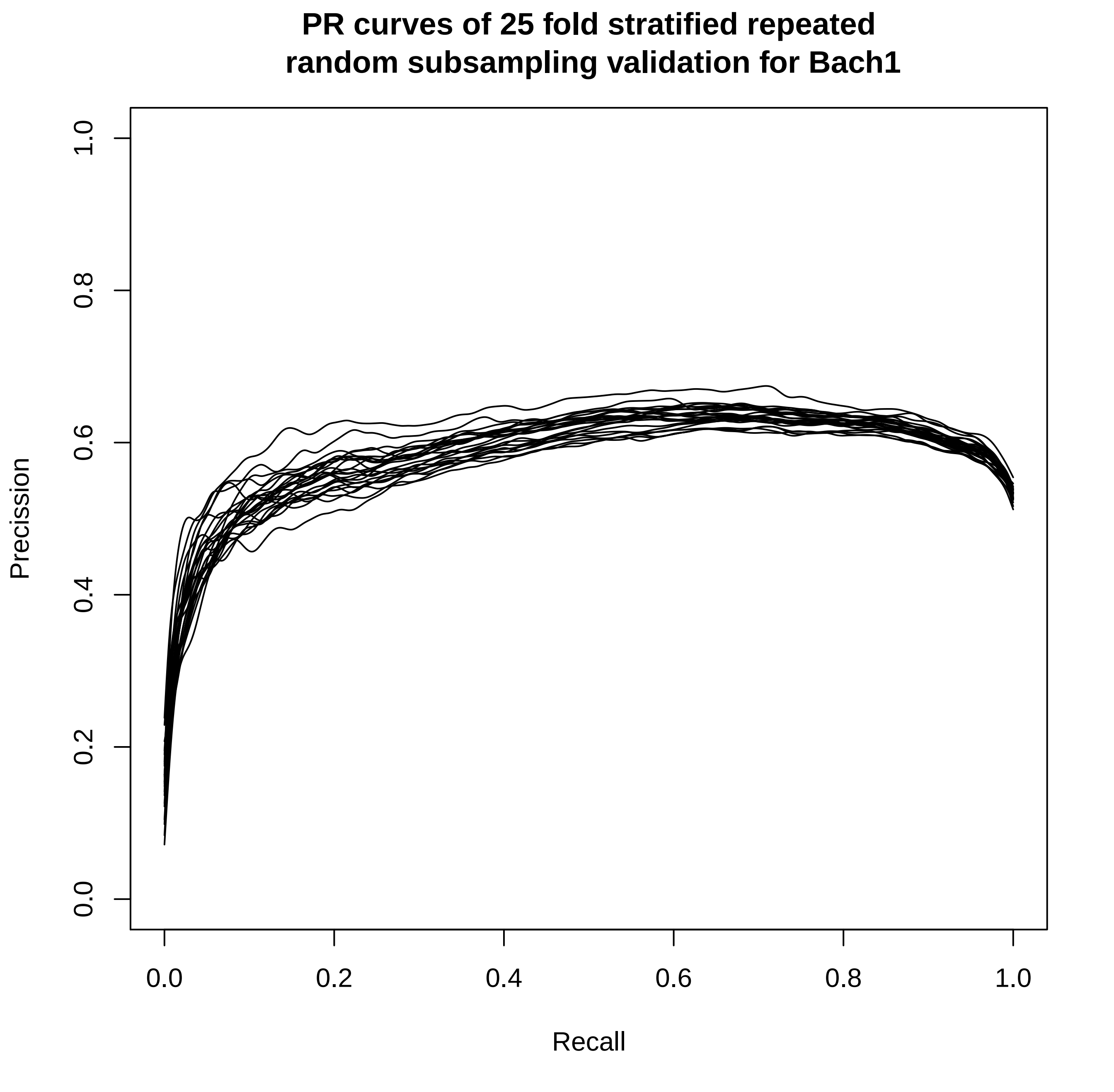
PR curves of 25 fold stratified repeated
random subsampling validation for Bach1
Precission
Recall
0.0
0.2
0.4
0.6
0.8
1.0
0.0
0.2
0.4
0.6
0.8
1.0

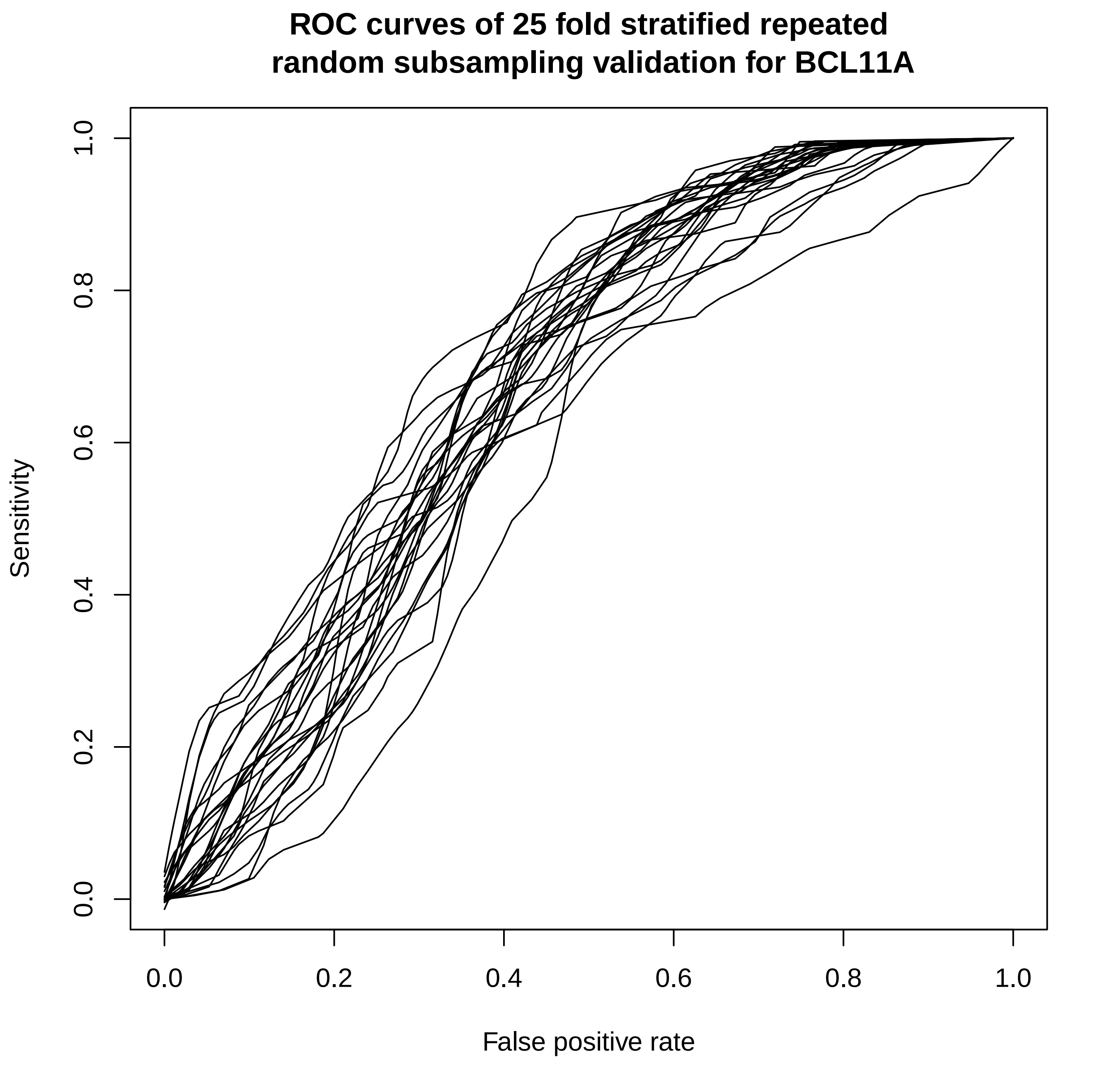
ROC curves of 25 fold stratified repeated
random subsampling validation for BCL11A
Sensitivity
0.0
0.2
0.4
0.6
0.8
1.0
0.0
0.2
0.4
0.6
0.8
1.0
False positive rate

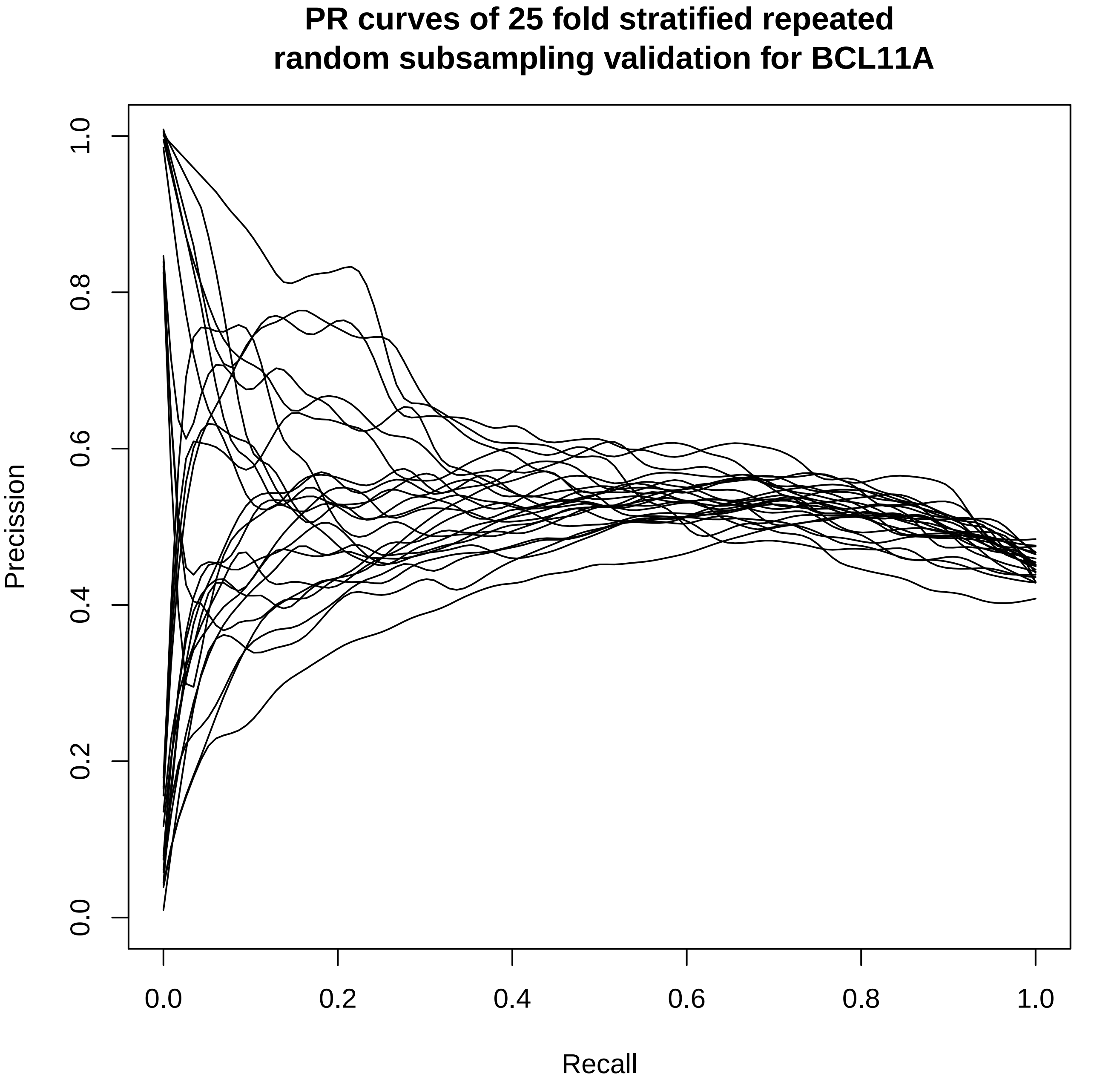
PR curves of 25 fold stratified repeated
random subsampling validation for BCL11A
Precission
Recall
0.0
0.2
0.4
0.6
0.8
1.0

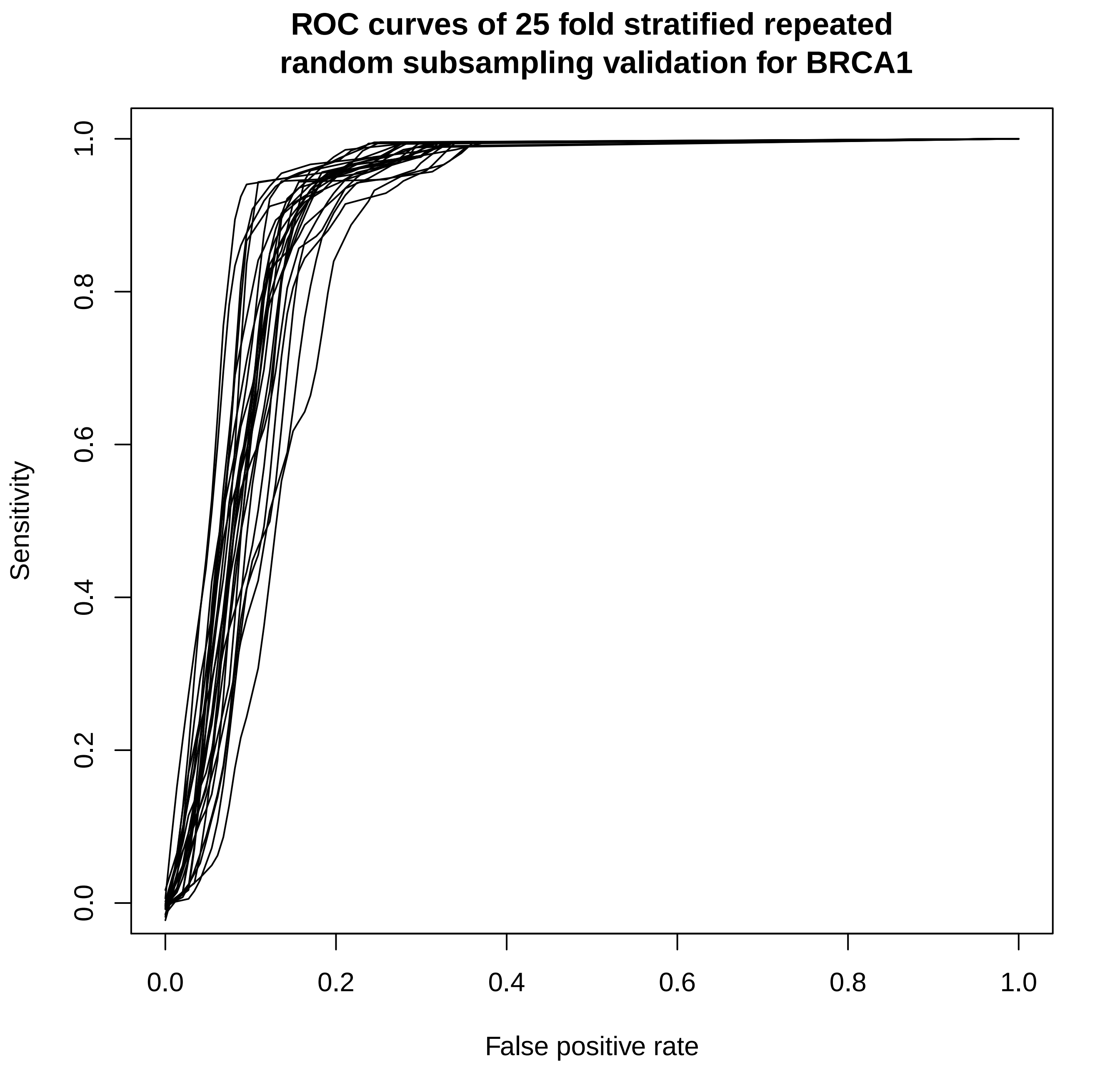

ROC curves of 25 fold stratified repeated random subsampling validation for BRCA1
Sensitivity
0.0
0.2
0.4
0.6
0.8
1.0
False positive rate

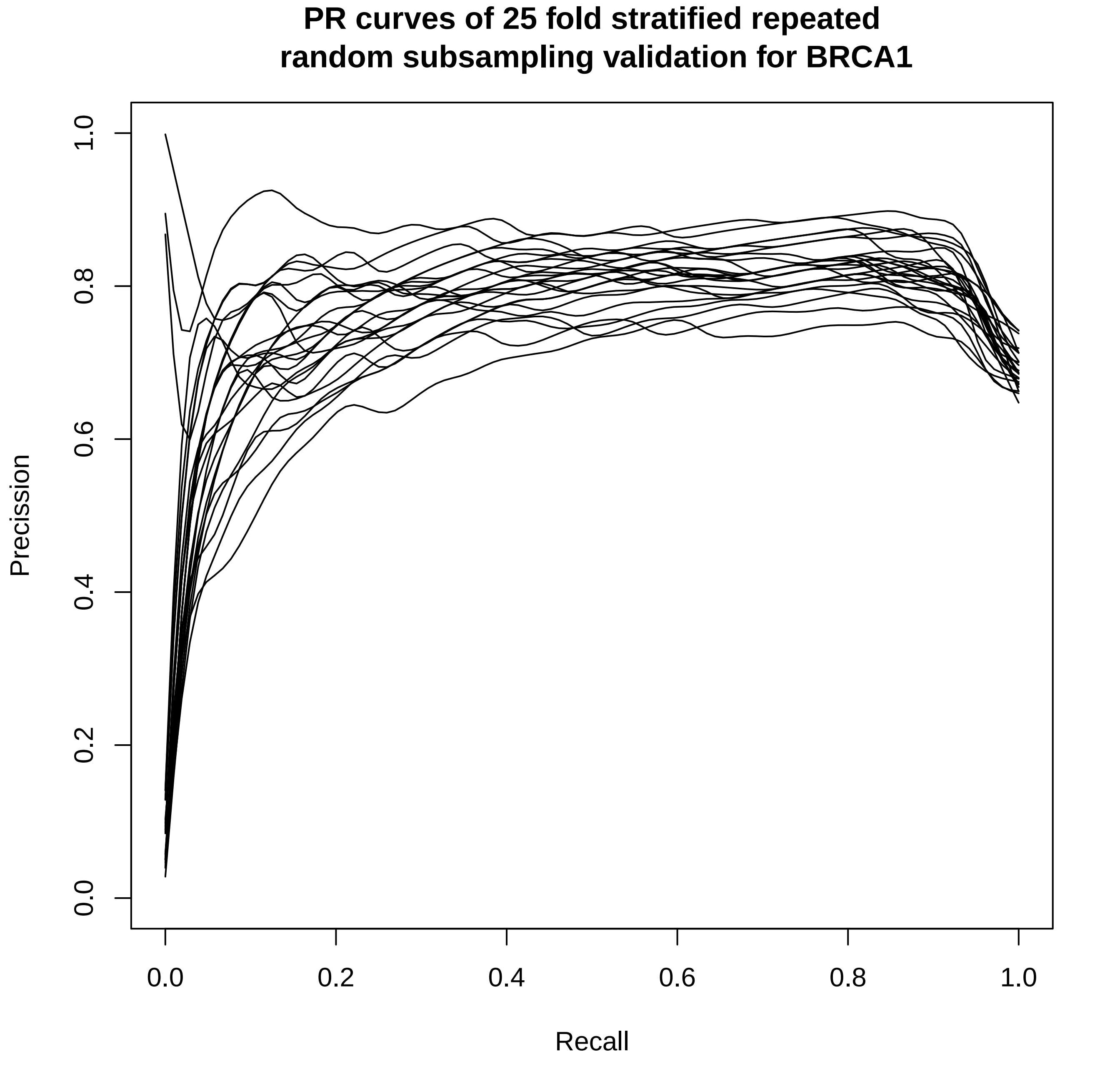
PR curves of 25 fold stratified repeated random subsampling validation for BRCA1
Precission
Recall
0.0
0.2
0.4
0.6
0.8
1.0

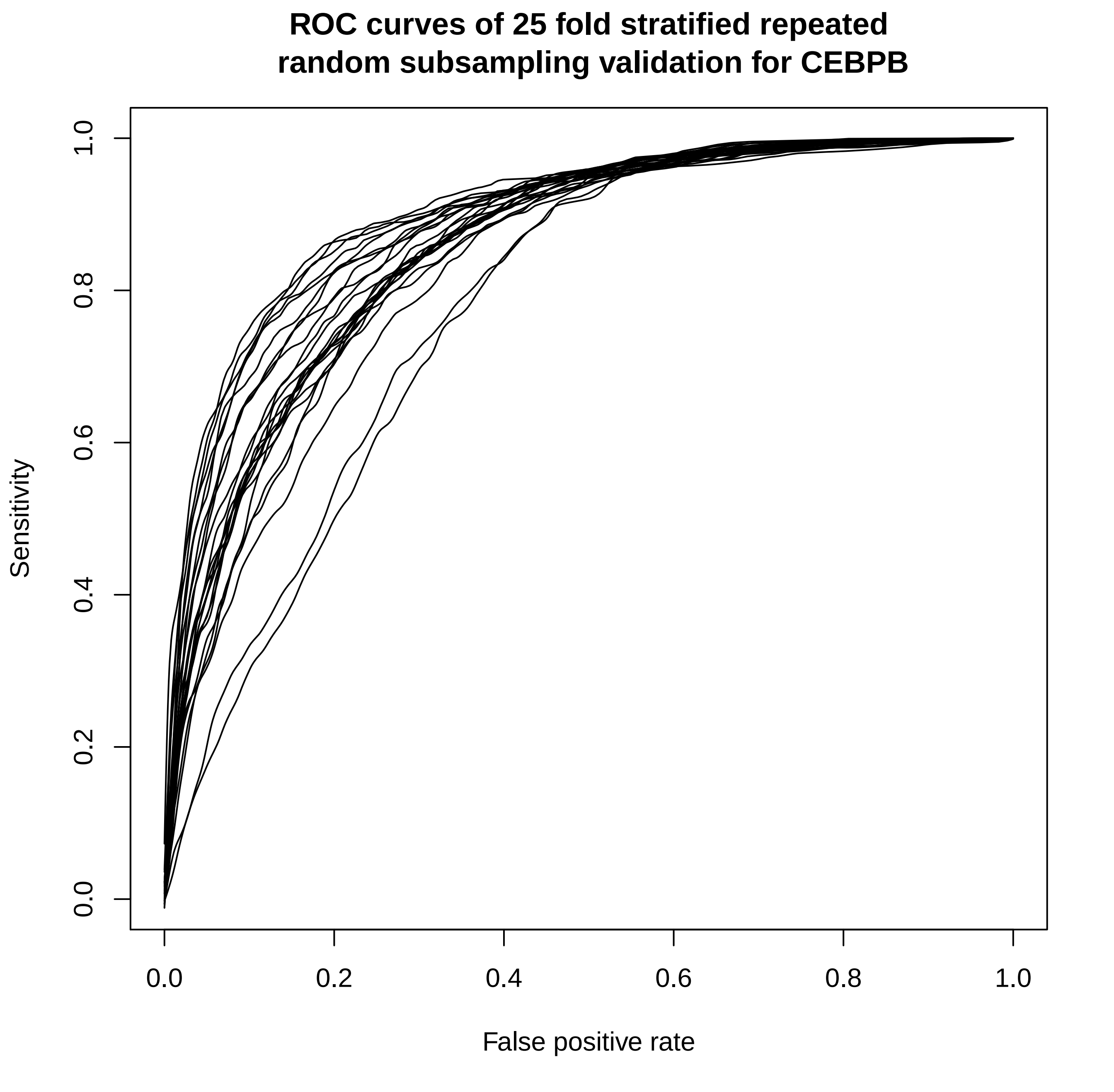
ROC curves of 25 fold stratified repeated
random subsampling validation for CEBPB
Sensitivity
0.0
0.2
0.4
0.6
0.8
1.0
0.0
0.2
0.4
0.6
0.8
1.0
False positive rate

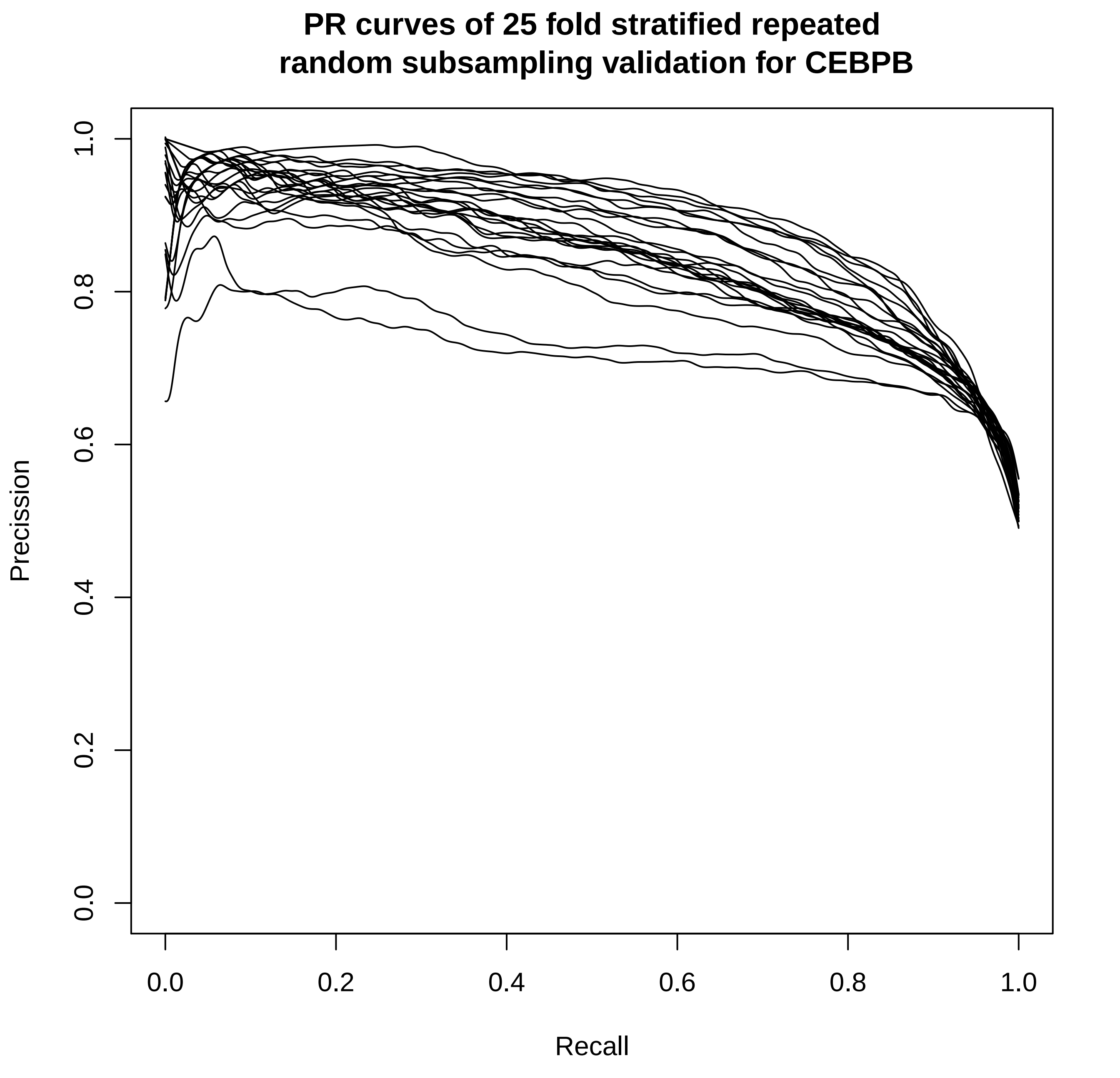
PR curves of 25 fold stratified repeated
random subsampling validation for CEBPB
Precission
Recall
0.0
0.2
0.4
0.6
0.8
1.0
0.0
0.2
0.4
0.6
0.8
1.0

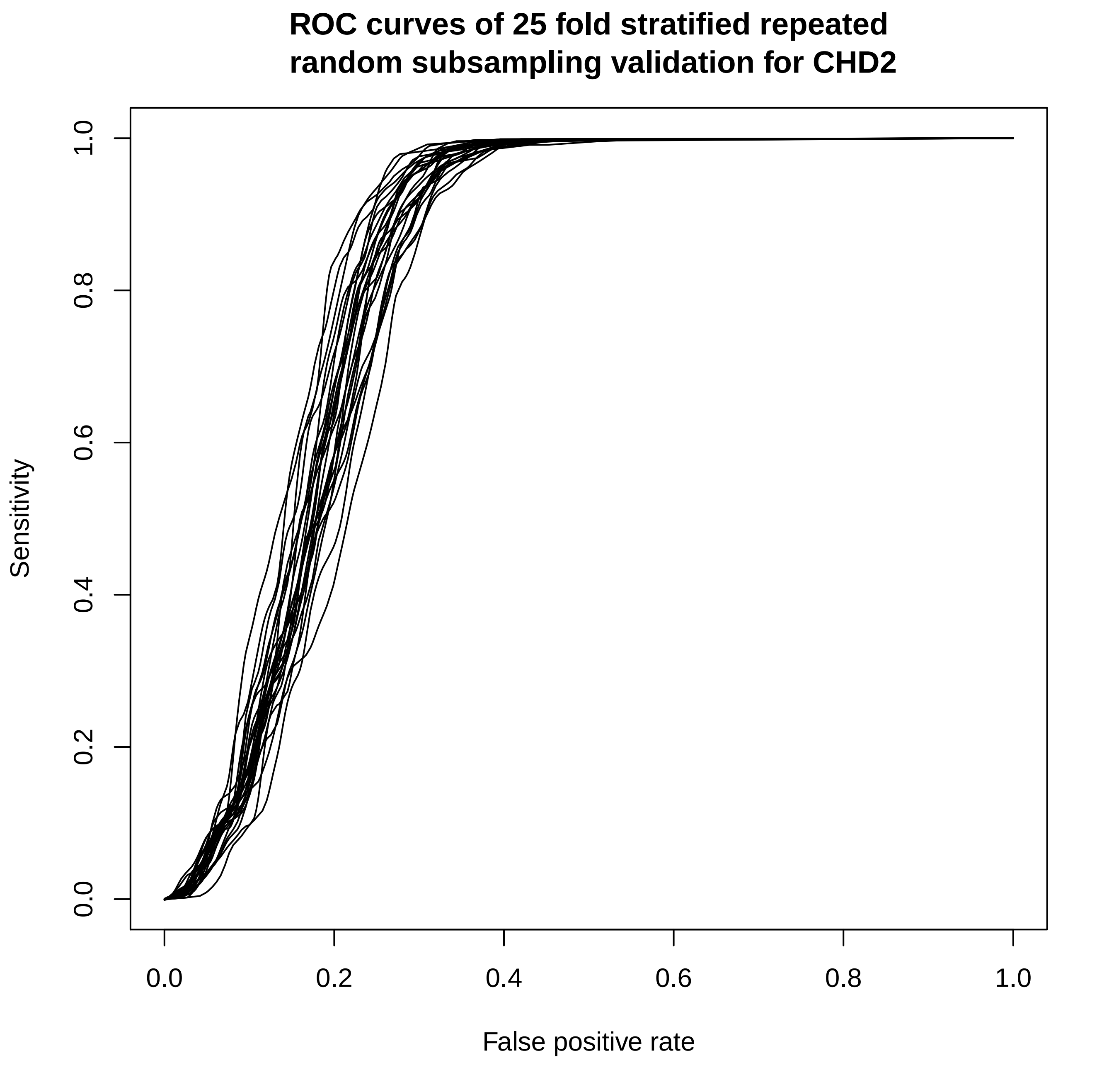

ROC curves of 25 fold stratified repeated
random subsampling validation for CHD2
Sensitivity
False positive rate
0.0
0.2
0.4
0.6
0.8
1.0

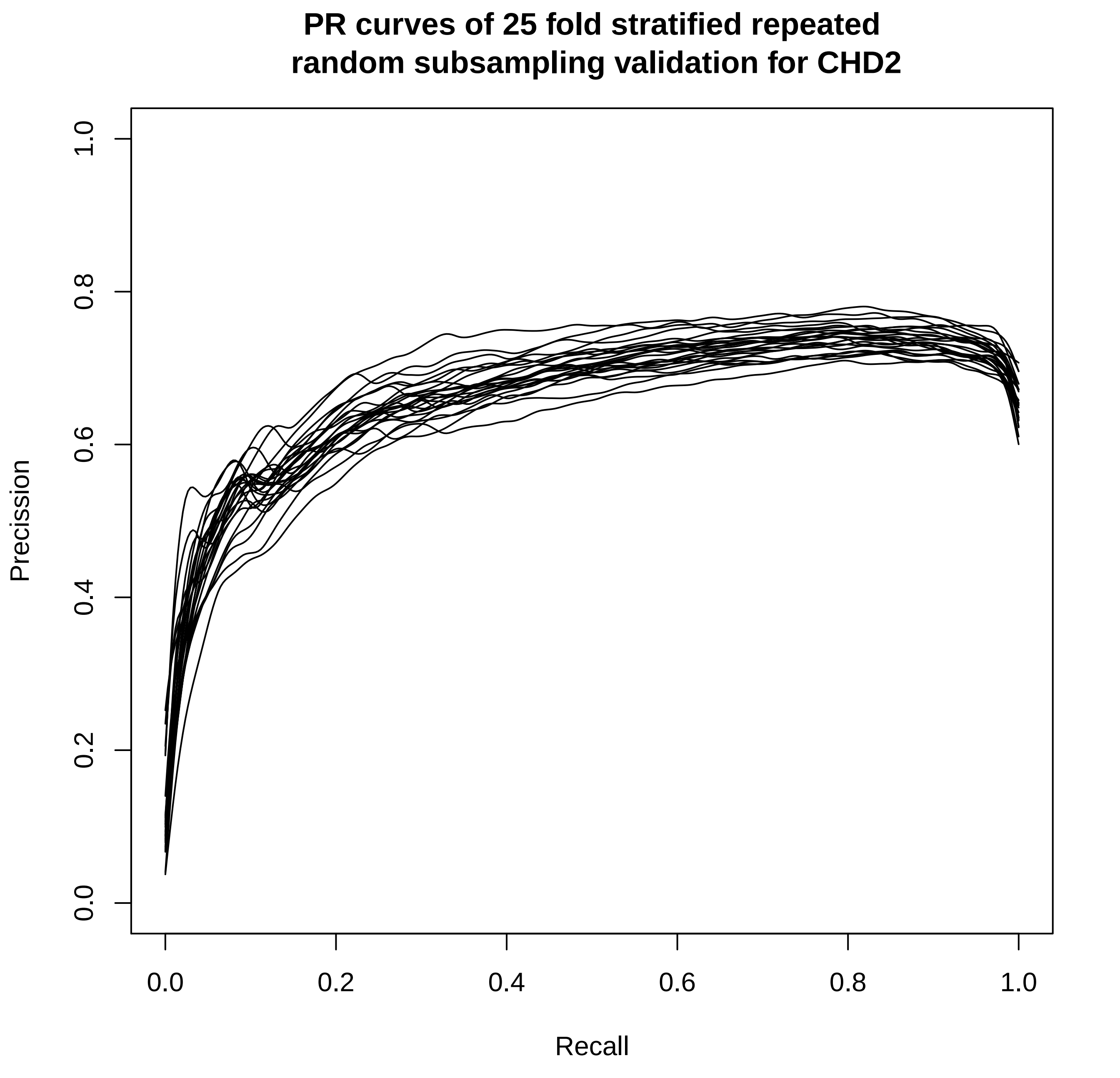
PR curves of 25 fold stratified repeated
random subsampling validation for CHD2
Precission
Recall
0.0
0.2
0.4
0.6
0.8
1.0

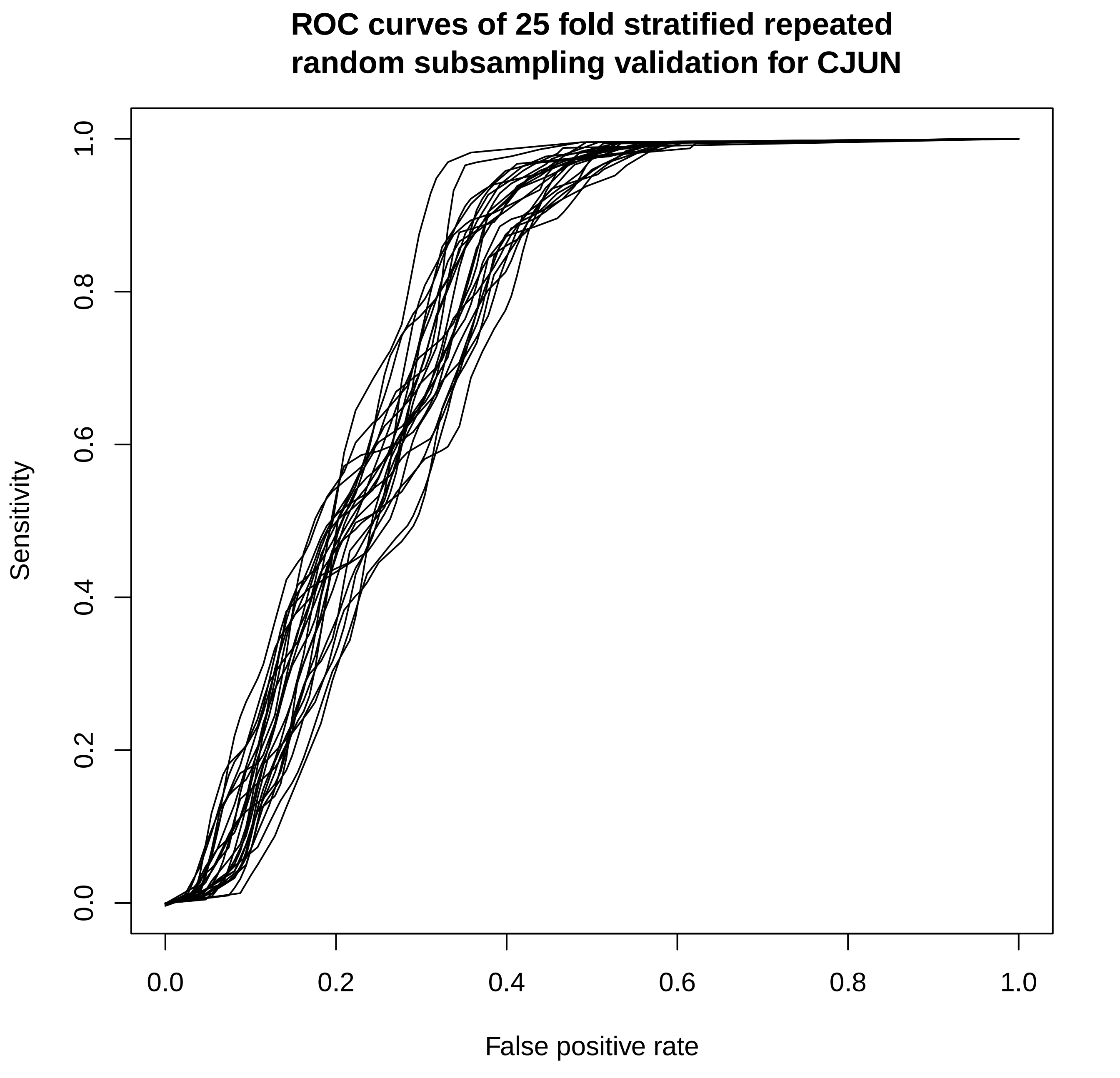
ROC curves of 25 fold stratified repeated random subsampling validation for CJUN
Sensitivity
0.0
0.2
0.4
0.6
0.8
1.0
False positive rate
0.0
0.2
0.4
0.6
0.8
1.0

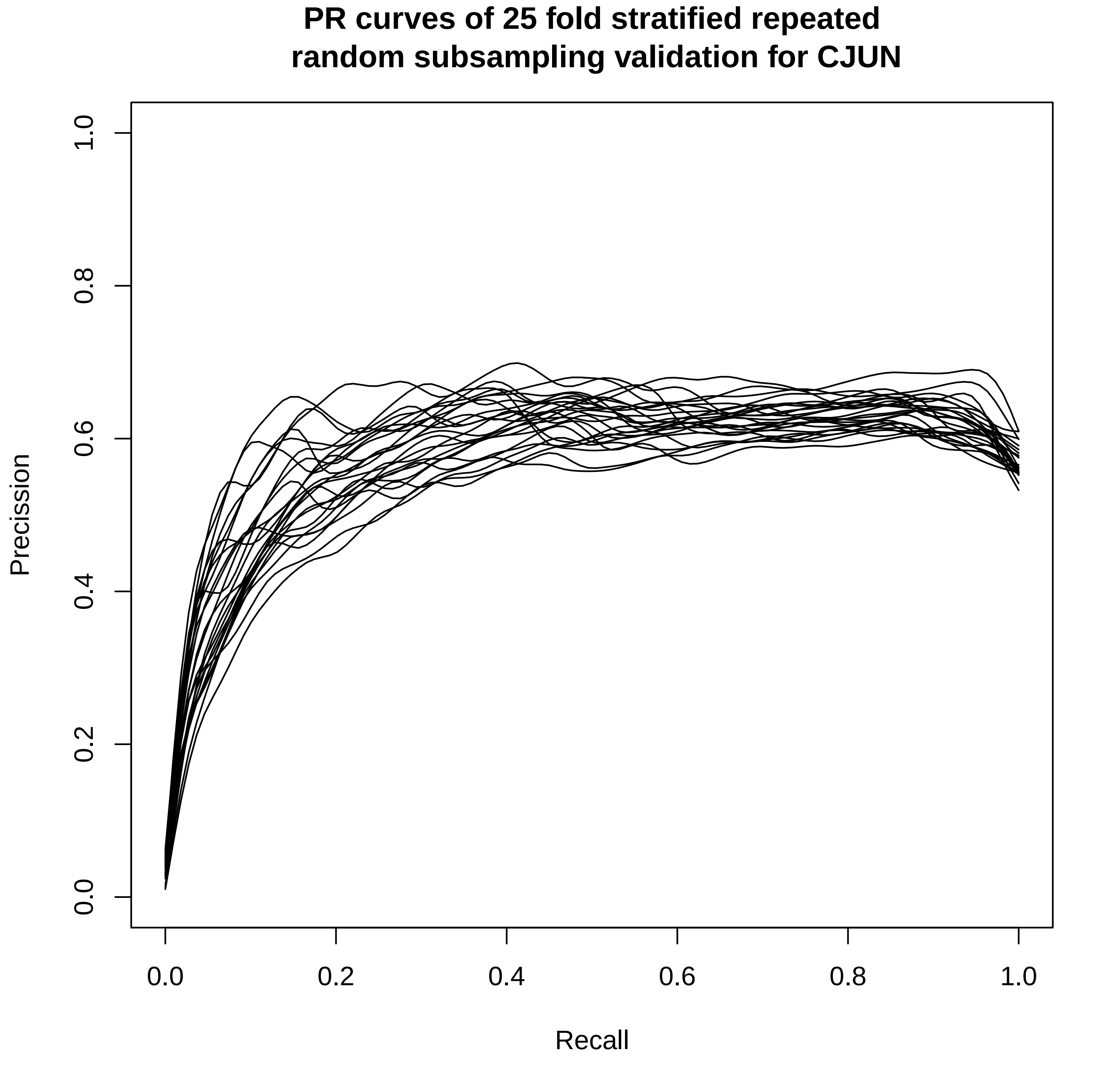

PR curves of 25 fold stratified repeated random subsampling validation for CJUN
Precission
Recall
0.0
0.2
0.4
0.6
0.8
1.0

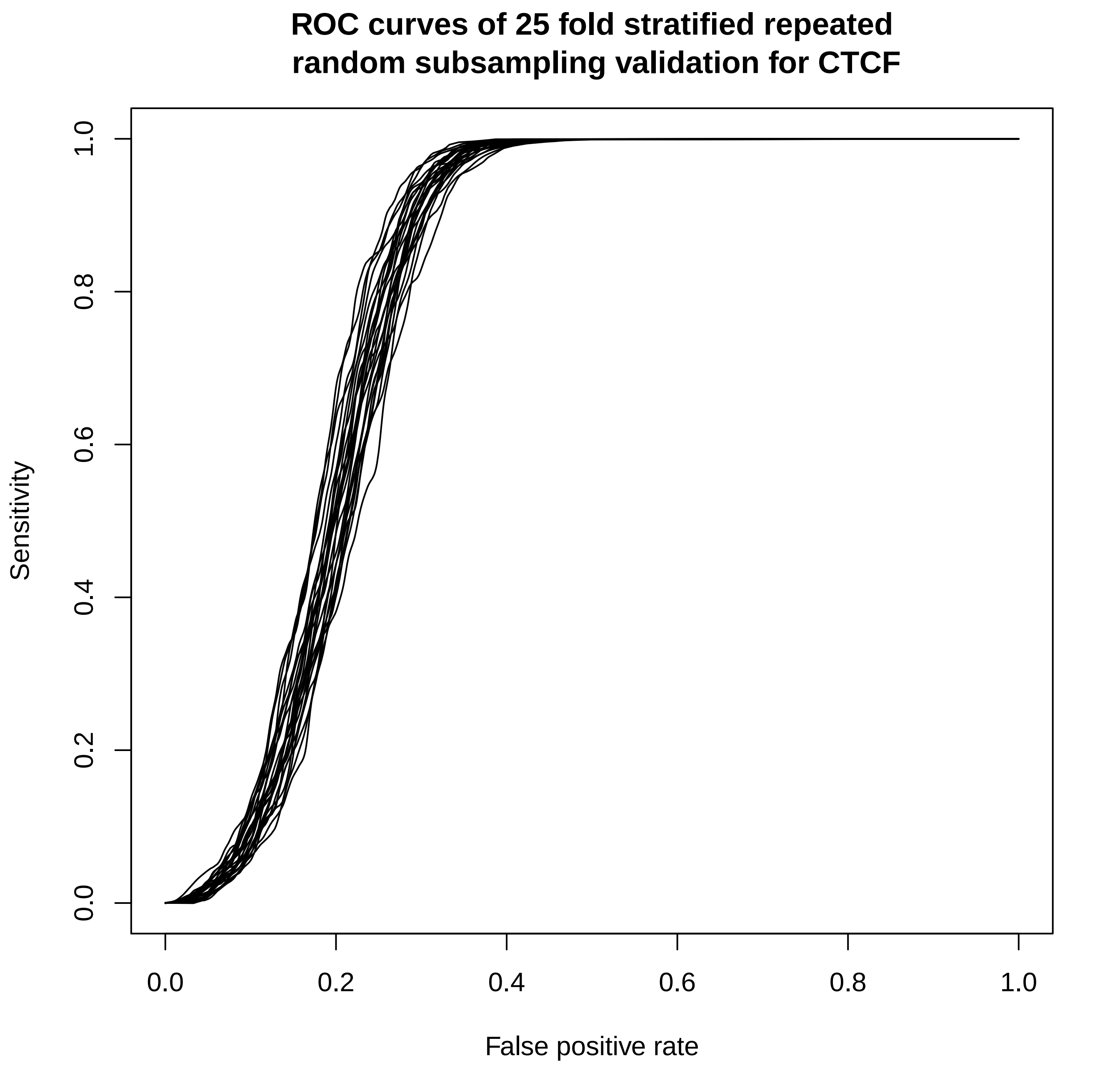

ROC curves of 25 fold stratified repeated
random subsampling validation for CTCF
Sensitivity
0.0
0.2
0.4
0.6
0.8
1.0
False positive rate
0.0
0.2
0.4
0.6
0.8
1.0

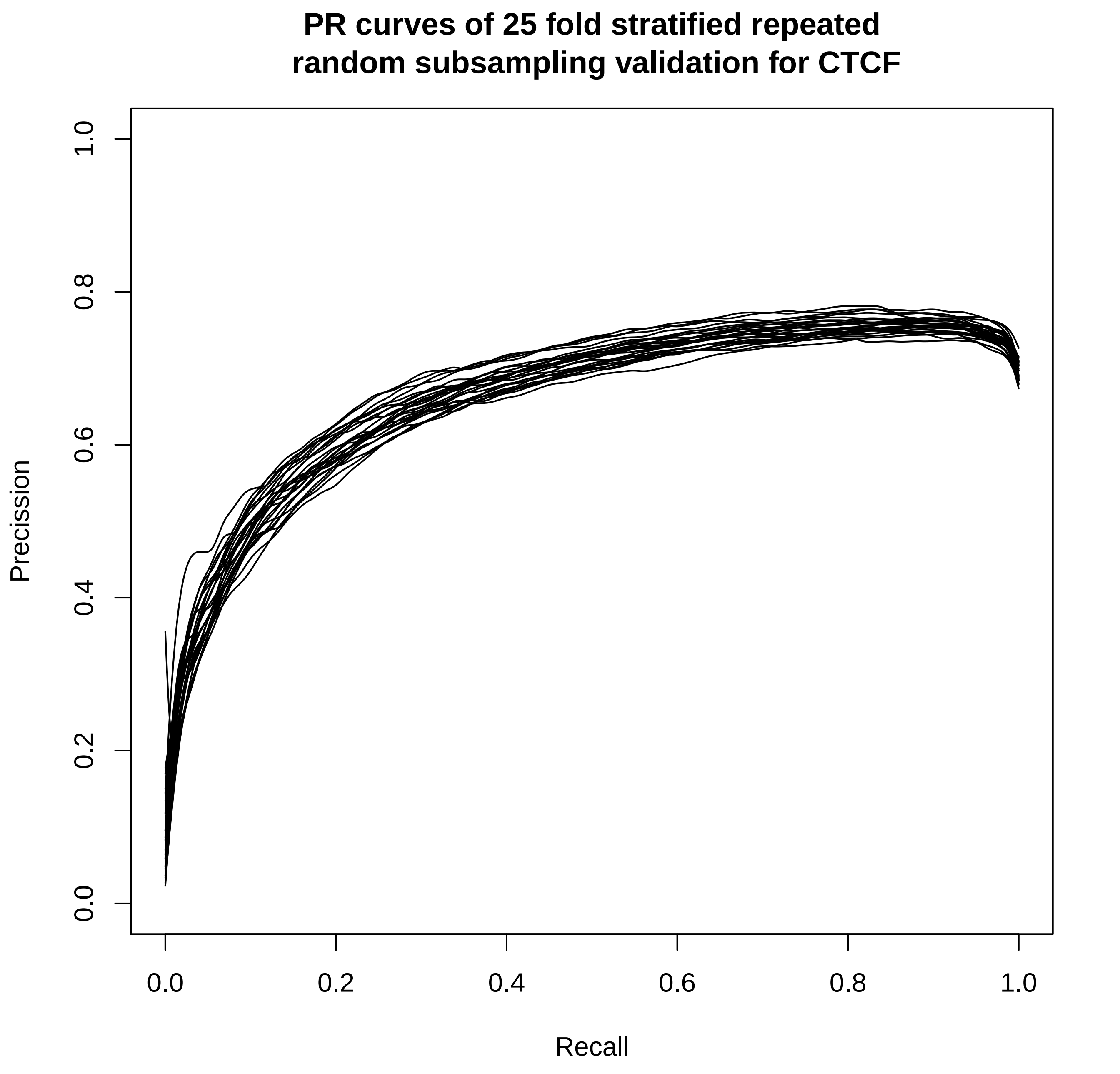
PR curves of 25 fold stratified repeated
random subsampling validation for CTCF
Precission
Recall
0.0
0.2
0.4
0.6
0.8
1.0

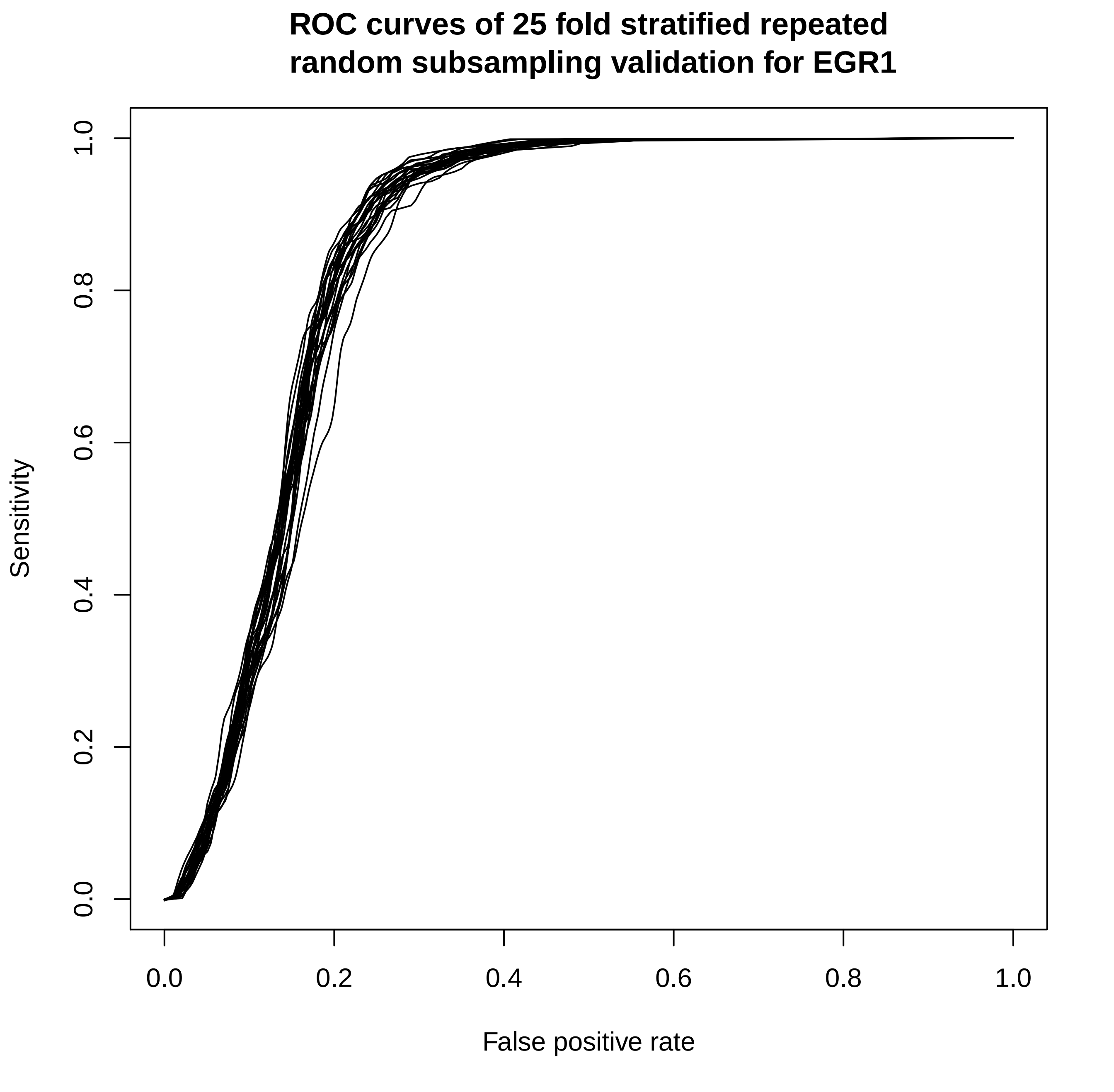
ROC curves of 25 fold stratified repeated random subsampling validation for EGR1
Sensitivity
0.0
0.2
0.4
0.6
0.8
1.0
False positive rate
0.0
0.2
0.4
0.6
0.8
1.0

PR curves of 25 fold stratified repeated random subsampling validation for EGR1

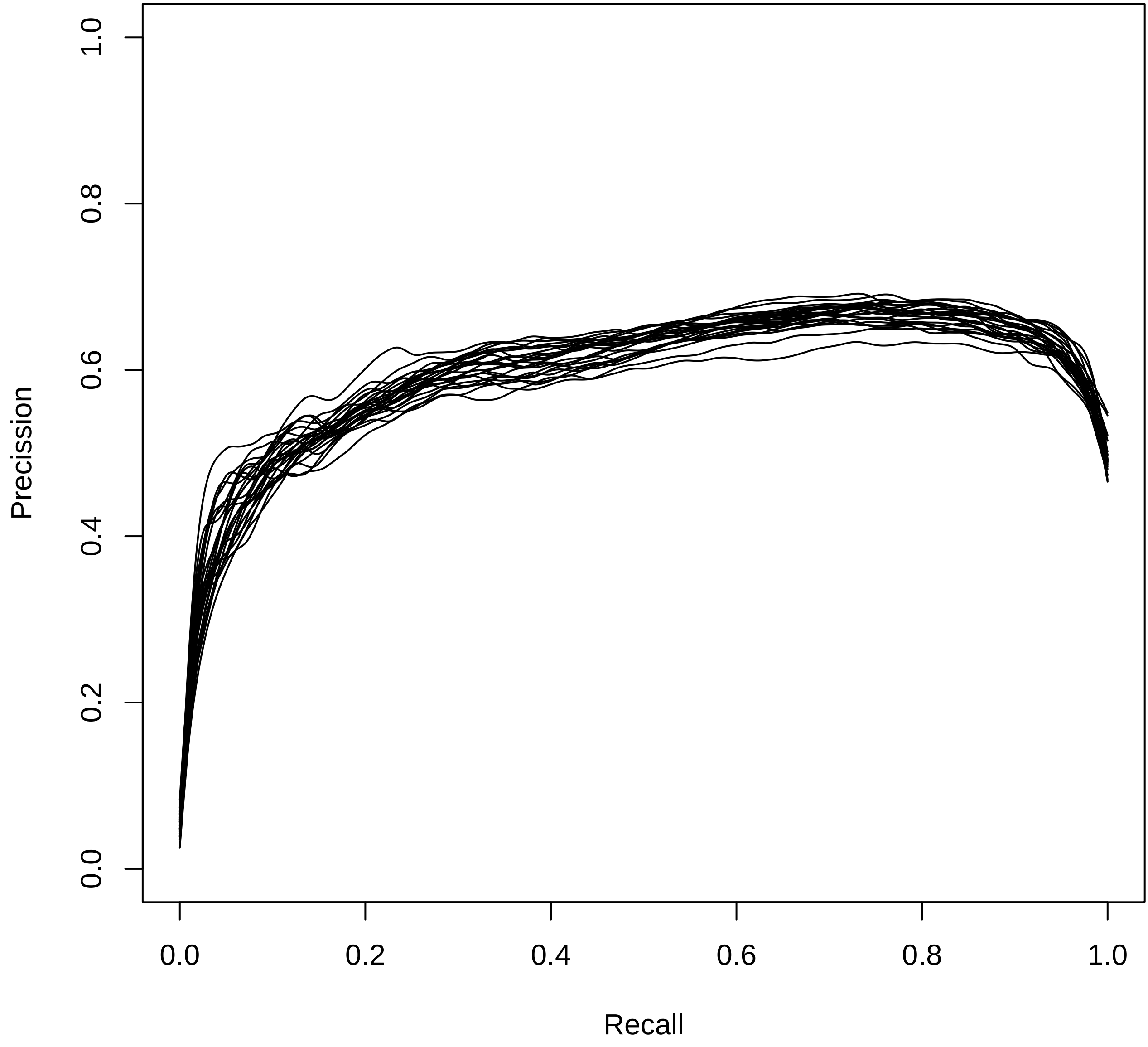

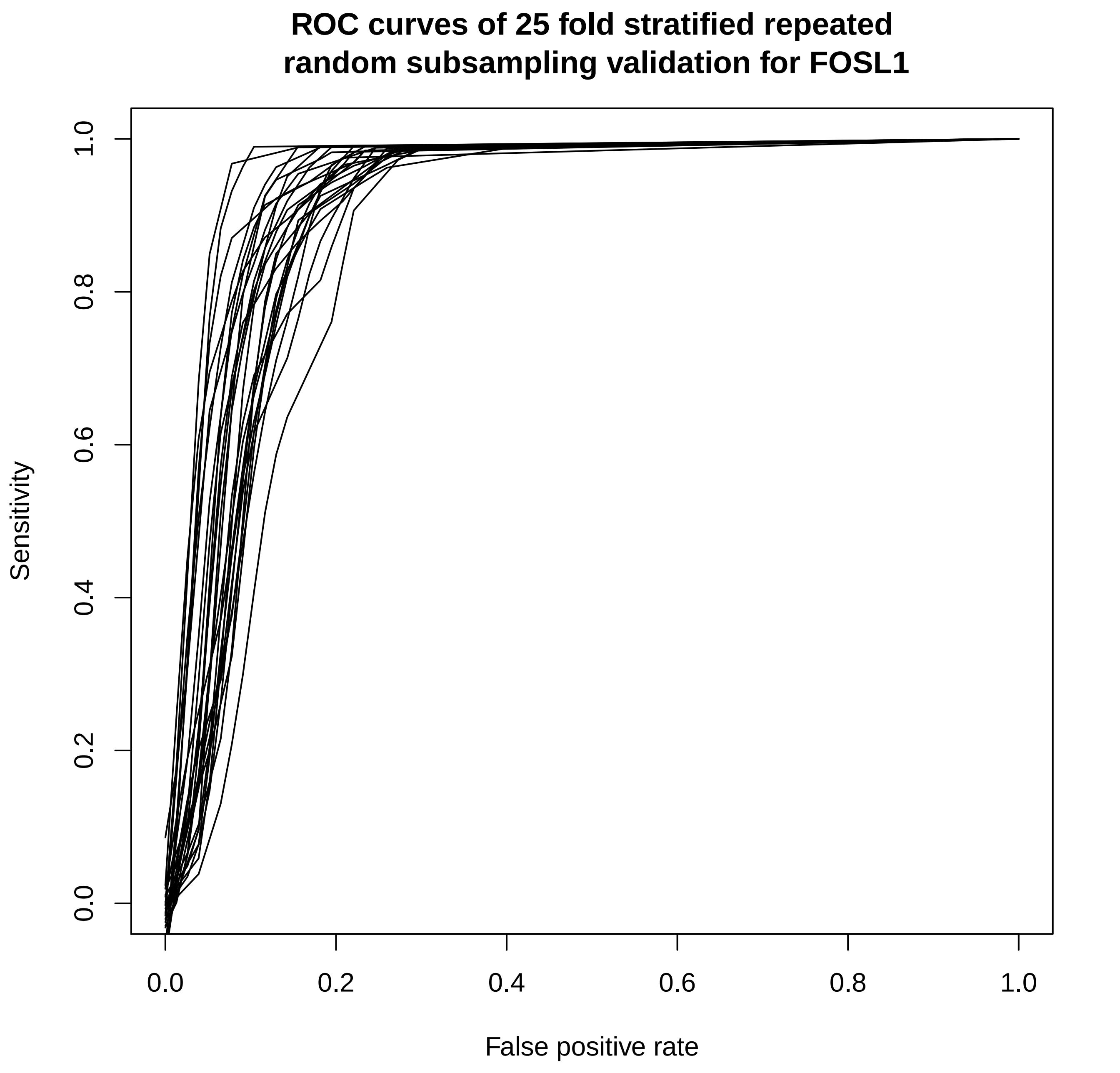
ROC curves of 25 fold stratified repeated
random subsampling validation for FOSL1
Sensitivity
0.0
0.2
0.4
0.6
0.8
1.0
False positive rate
0.0
0.2
0.4
0.6
0.8
1.0

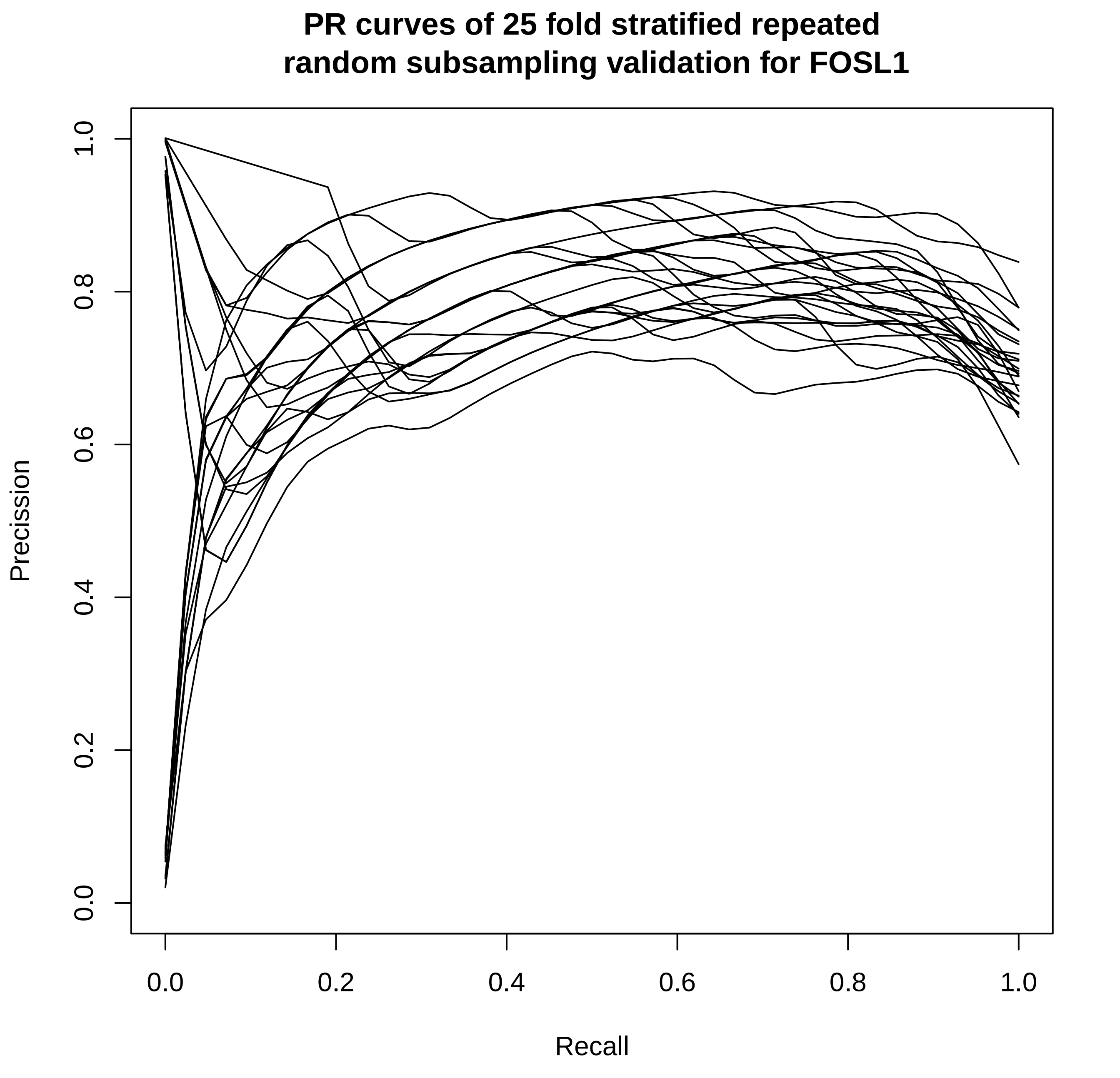
PR curves of 25 fold stratified repeated
random subsampling validation for FOSL1
Precission
Recall
0.0
0.2
0.4
0.6
0.8
1.0

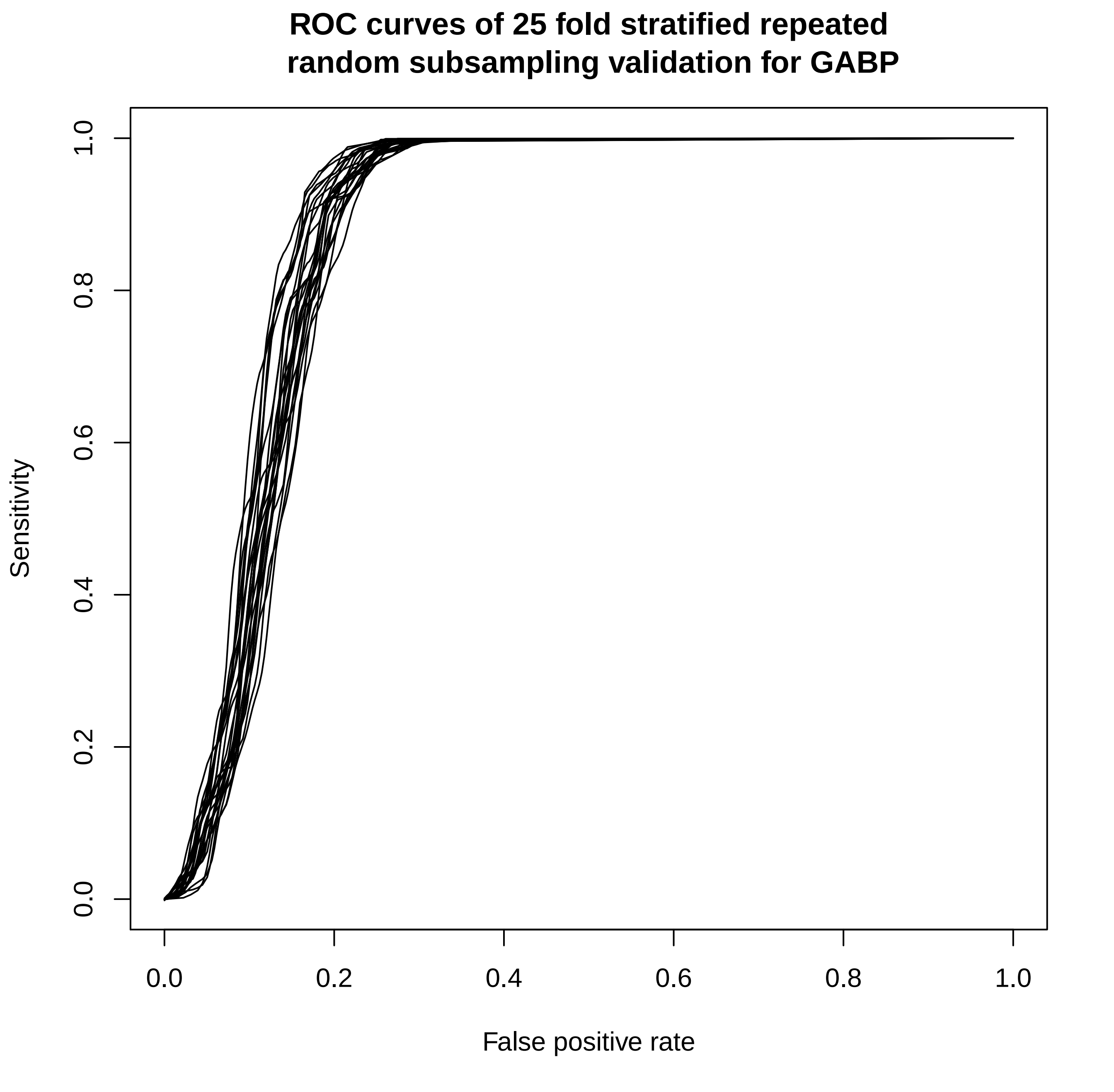

ROC curves of 25 fold stratified repeated
random subsampling validation for GABP
Sensitivity
False positive rate
0.0
0.2
0.4
0.6
0.8
1.0
0.0
0.2
0.4
0.6
0.8
1.0

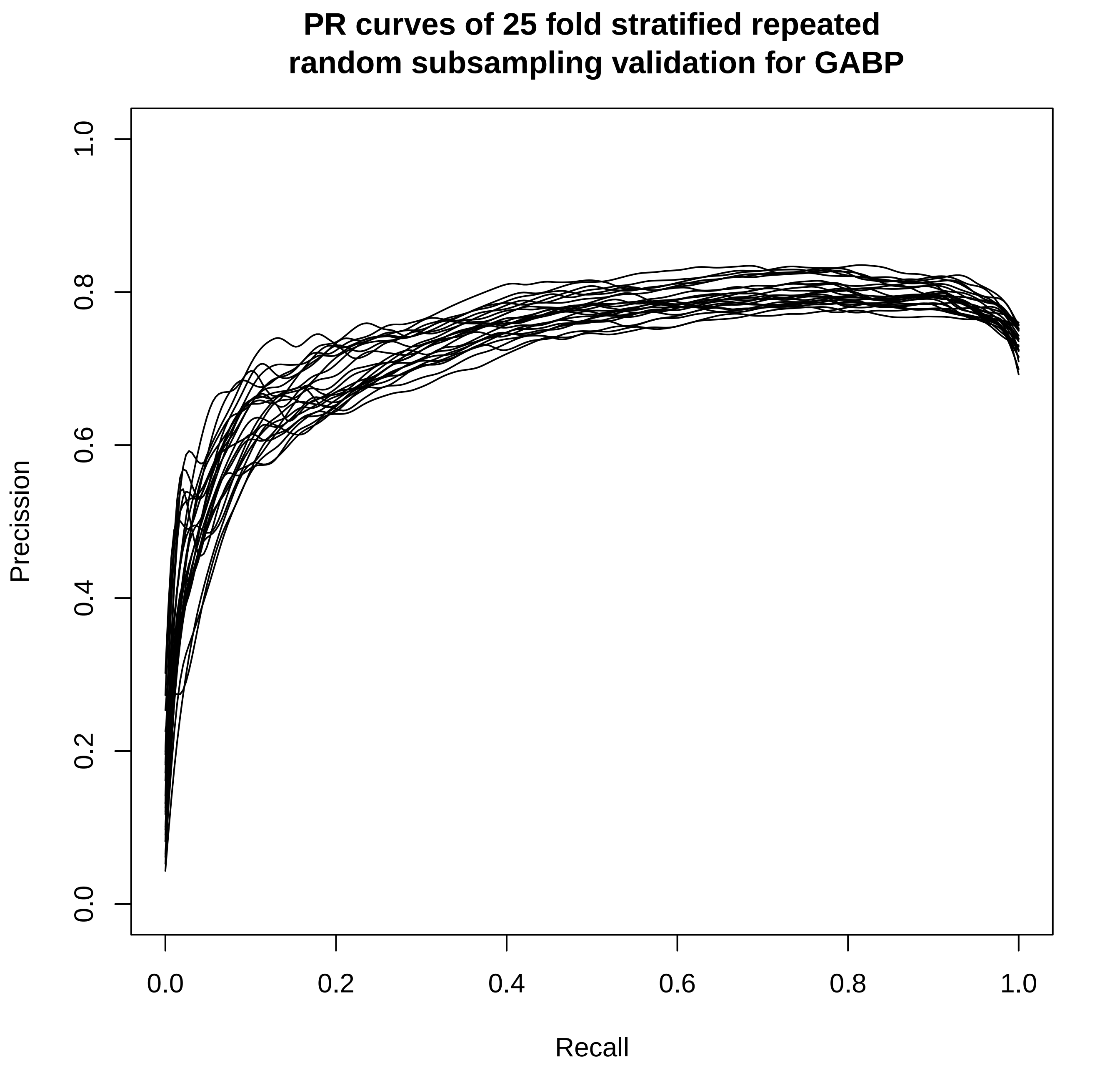
PR curves of 25 fold stratified repeated
random subsampling validation for GABP
Precission
0.0
0.2
0.4
0.6
0.8
1.0
Recall
0.0
0.2
0.4
0.6
0.8
1.0

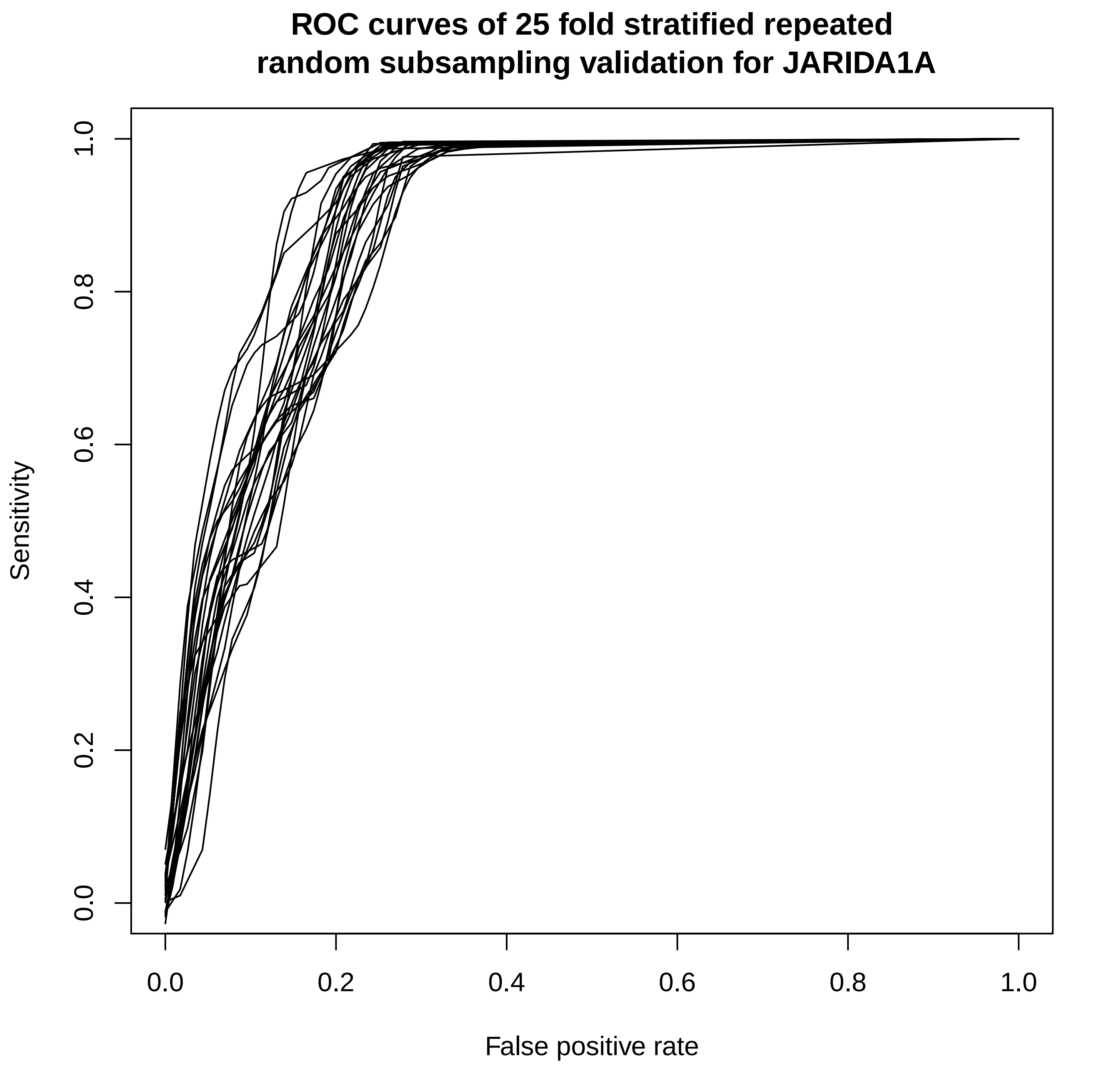
ROC curves of 25 fold stratified repeated
random subsampling validation for JARIDA1A
Sensitivity
False positive rate
0.0
0.2
0.4
0.6
0.8
1.0
0.0
0.2
0.4
0.6
0.8
1.0

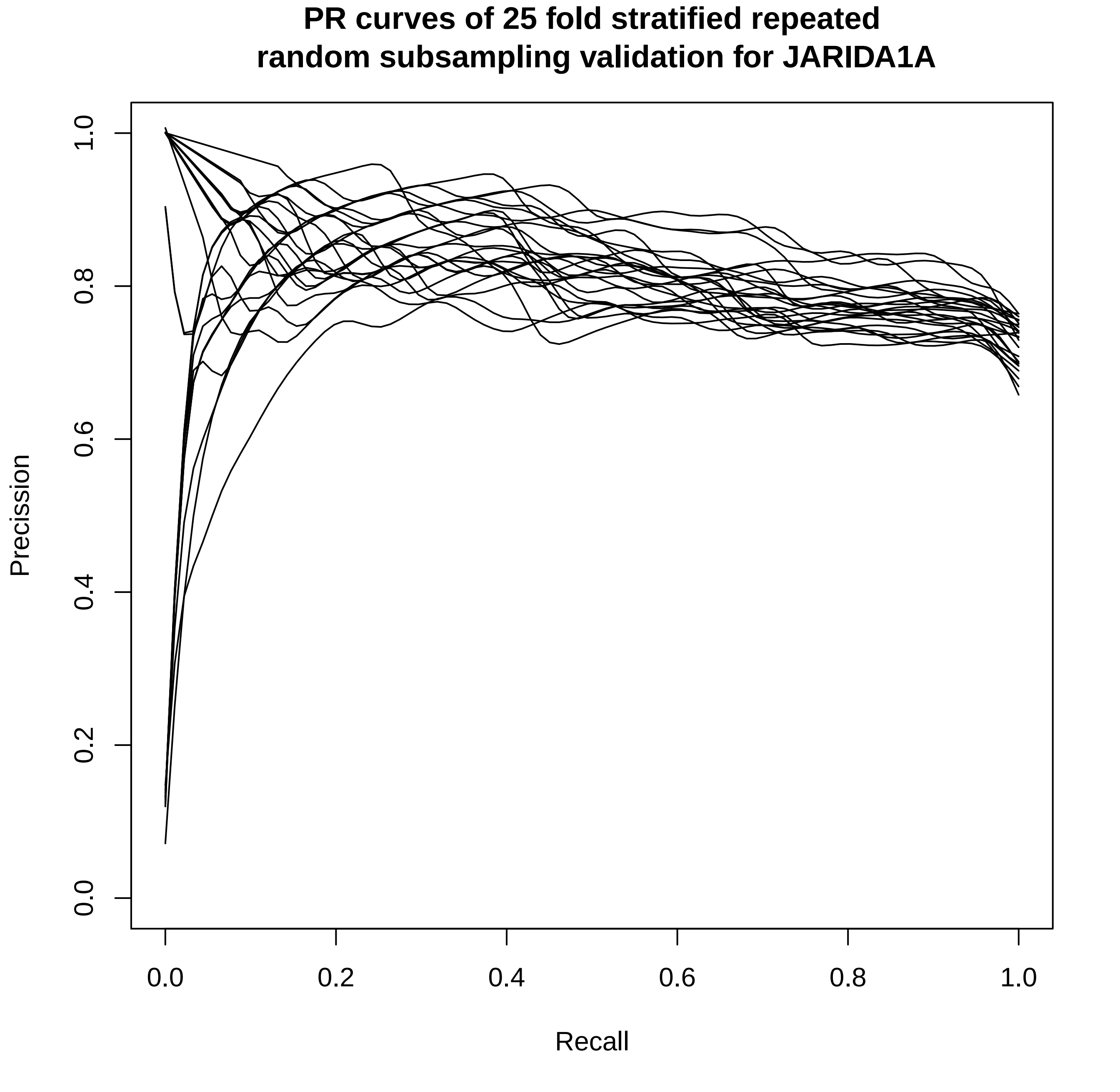
PR curves of 25 fold stratified repeated random subsampling validation for JARIDA1A
Precission
Recall
0.0
0.2
0.4
0.6
0.8
1.0
0.0
0.2
0.4
0.6
0.8
1.0

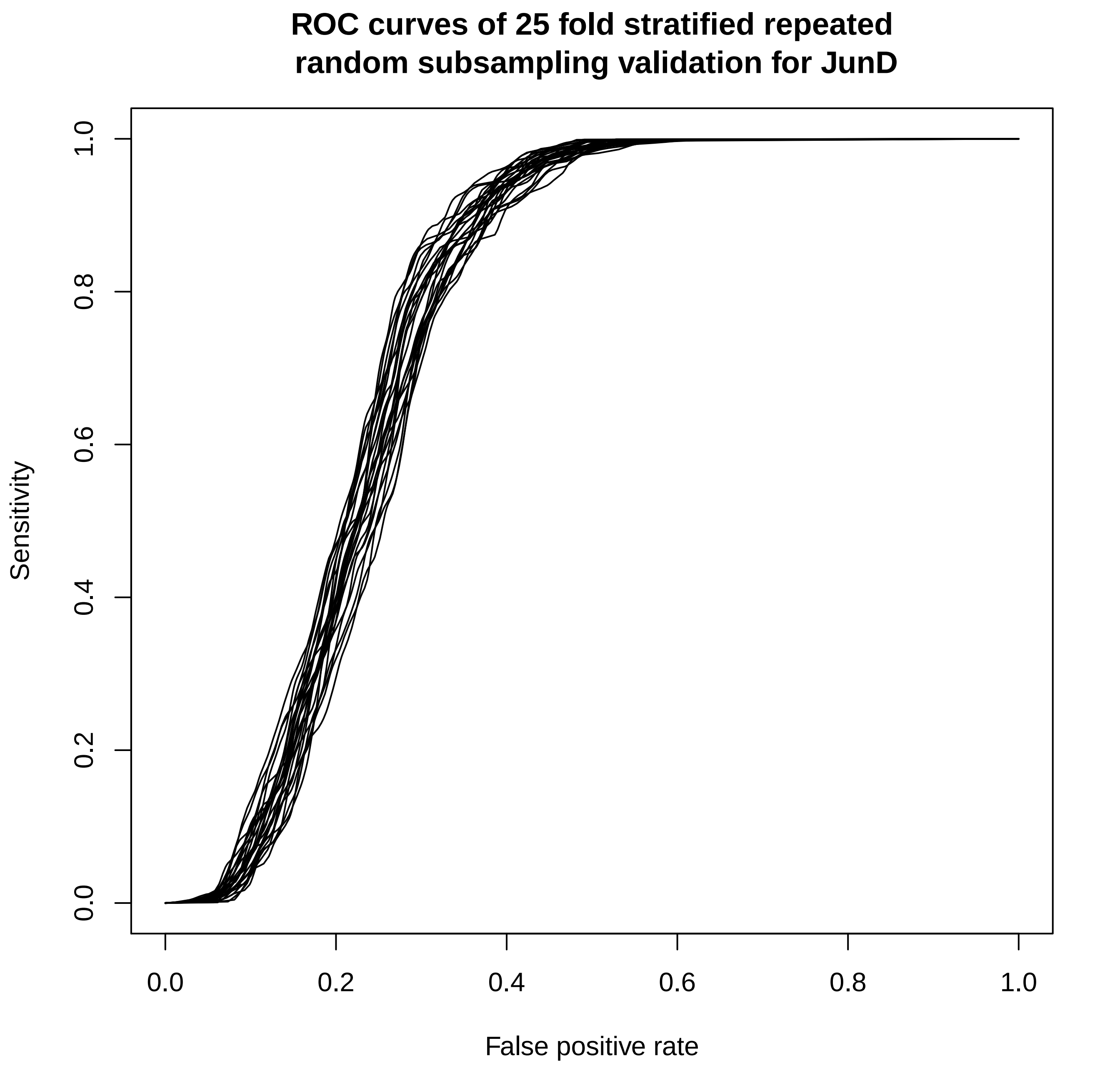
ROC curves of 25 fold stratified repeated
random subsampling validation for JunD
Sensitivity
False positive rate
0.0
0.2
0.4
0.6
0.8
1.0

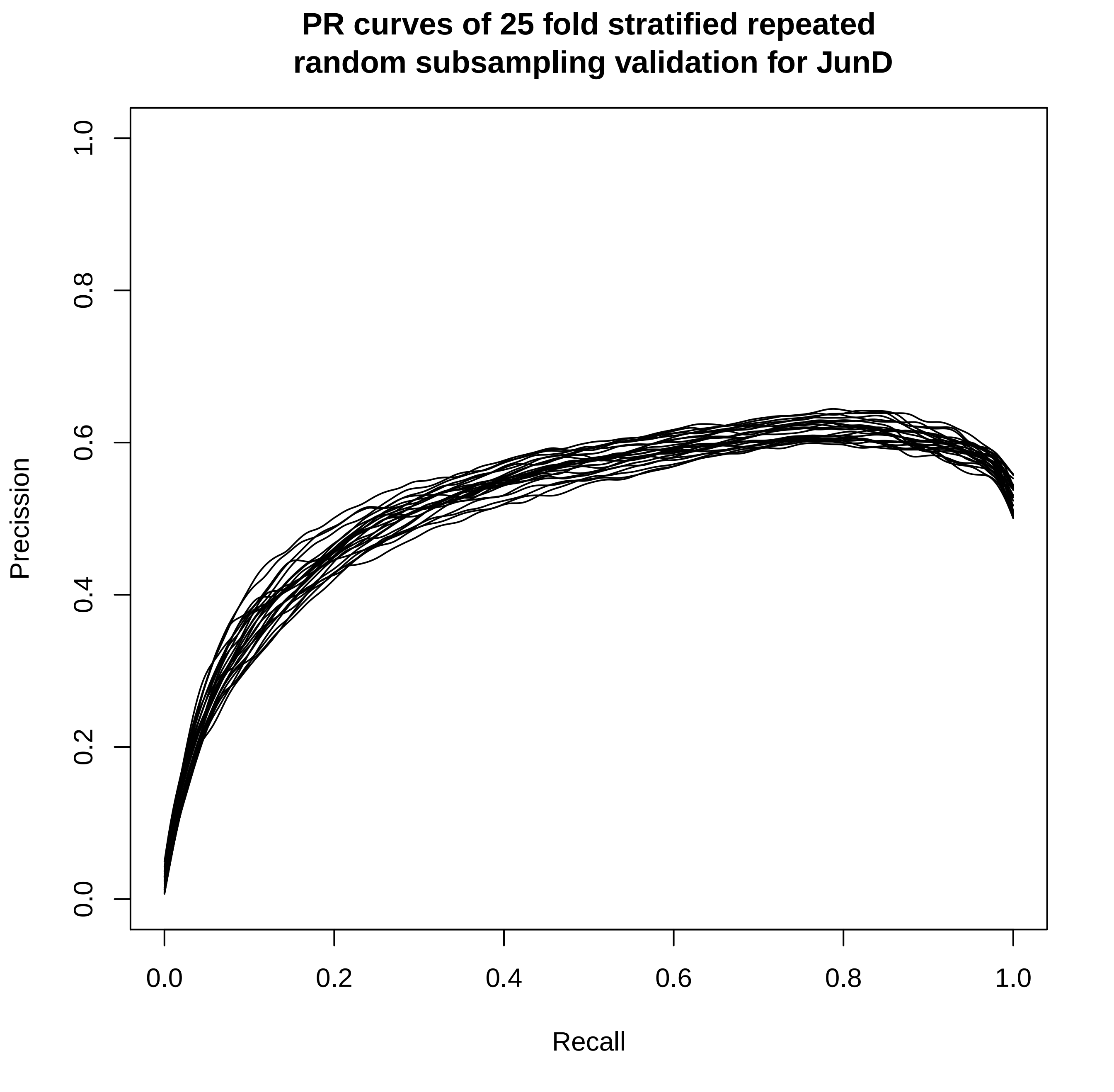

PR curves of 25 fold stratified repeated
random subsampling validation for JunD
Precission
Recall
0.0
0.2
0.4
0.6
0.8
1.0
0.0
0.2
0.4
0.6
0.8
1.0

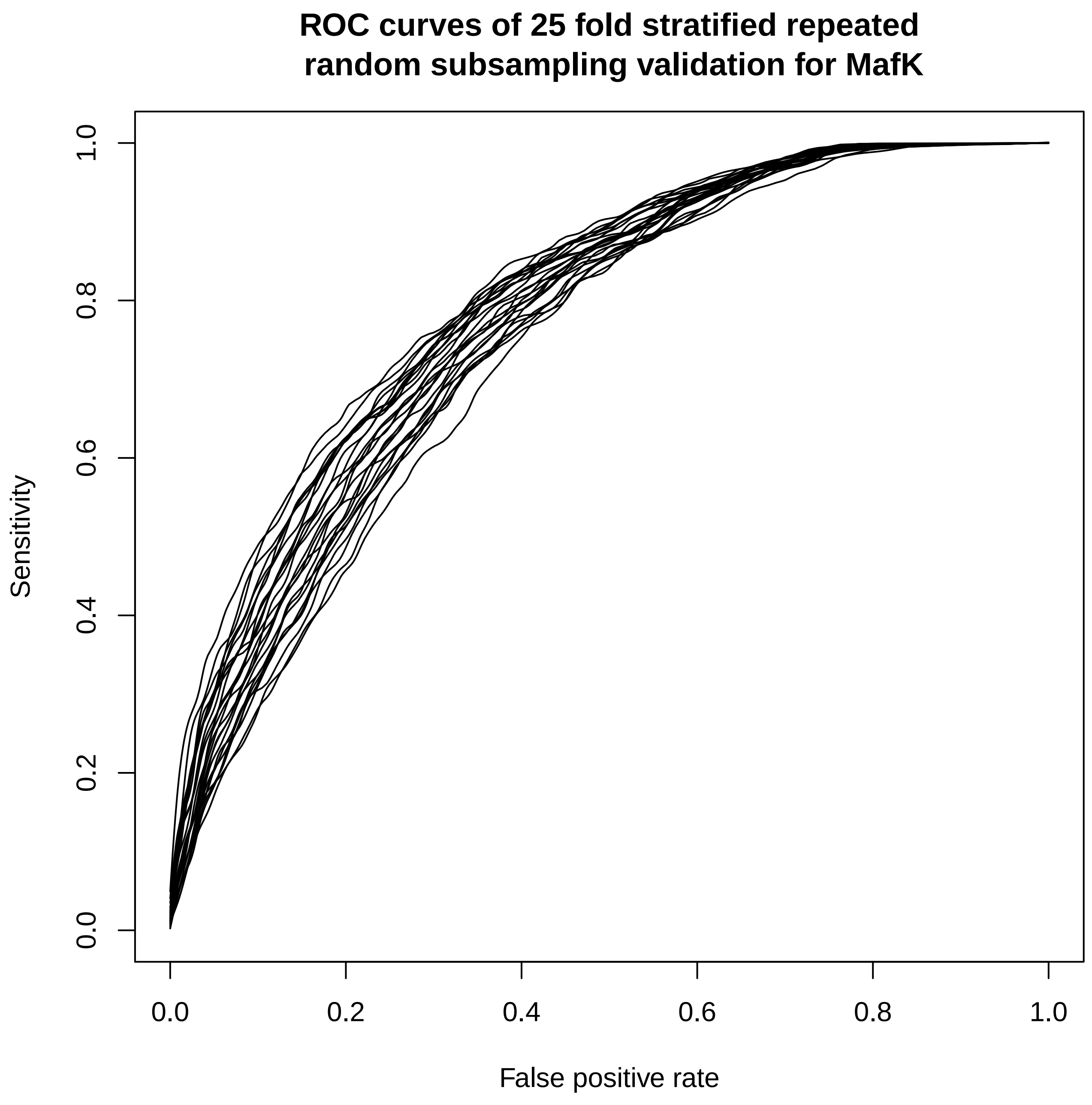

ROC curves of 25 fold stratified repeated
random subsampling validation for MafK
Sensitivity
False positive rate
0.0
0.2
0.4
0.6
0.8
1.0

PR curves of 25 fold stratified repeated random subsampling validation for MafK

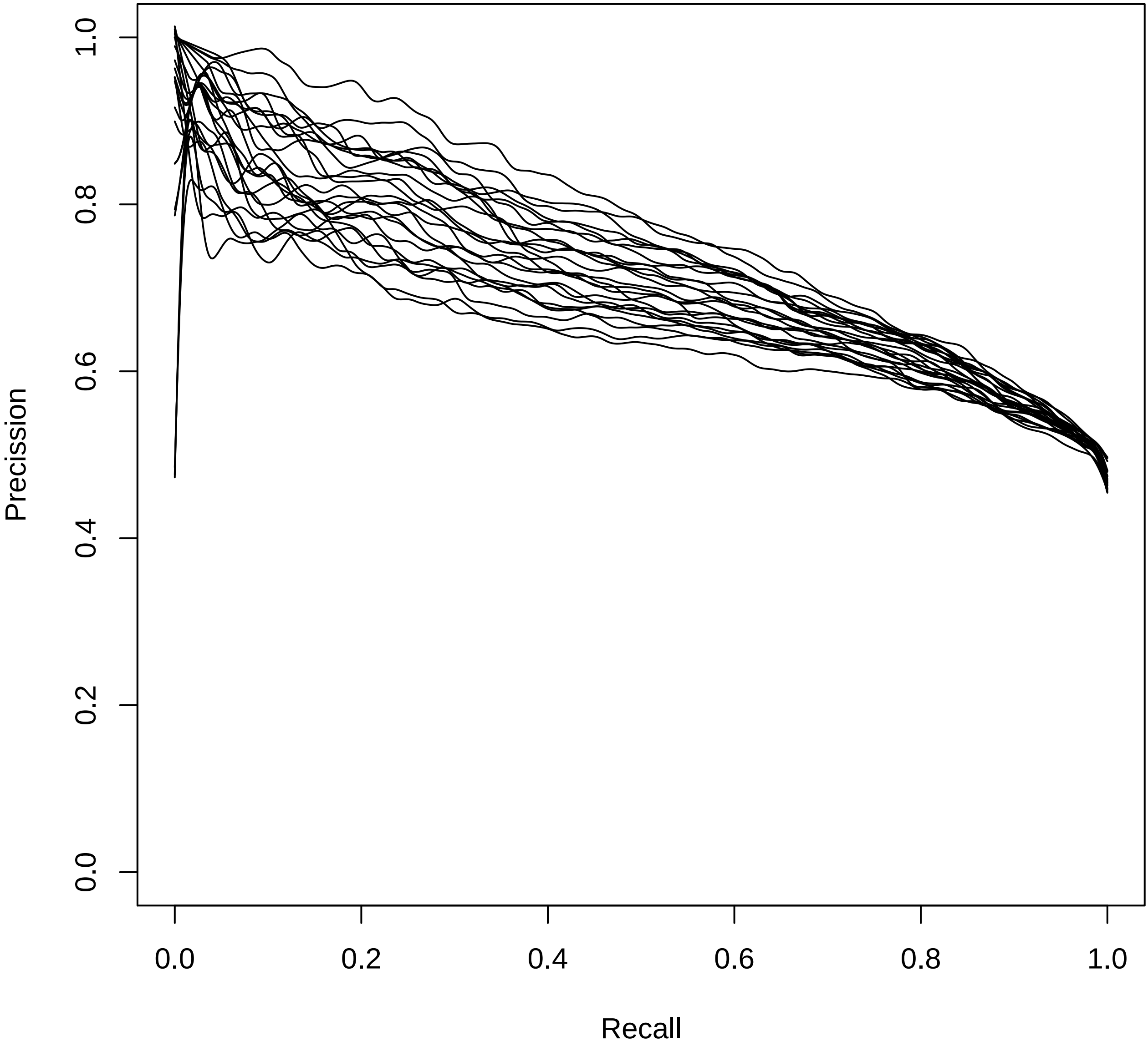

ROC curves of 25 fold stratified repeated random subsampling validation for Max

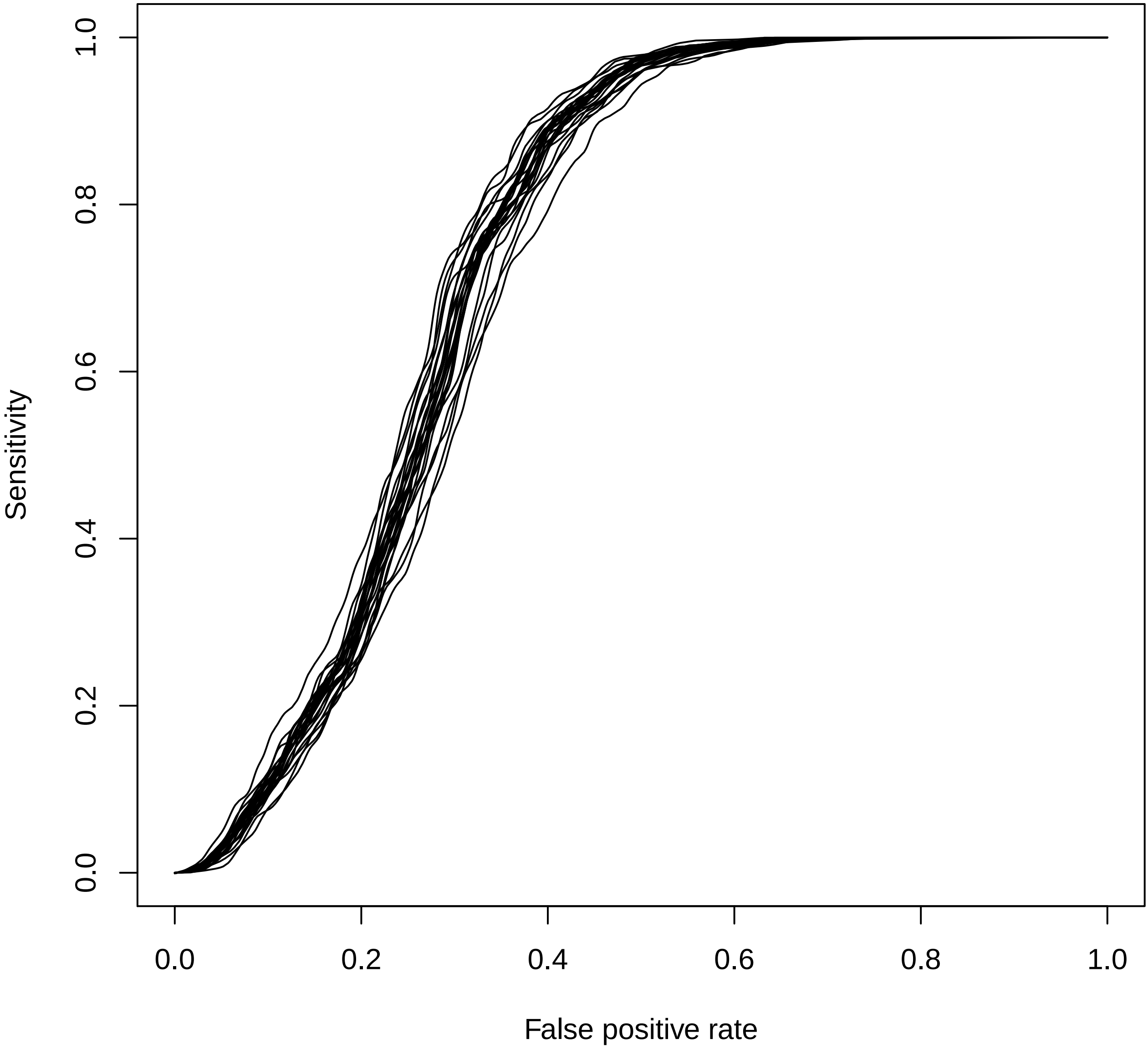

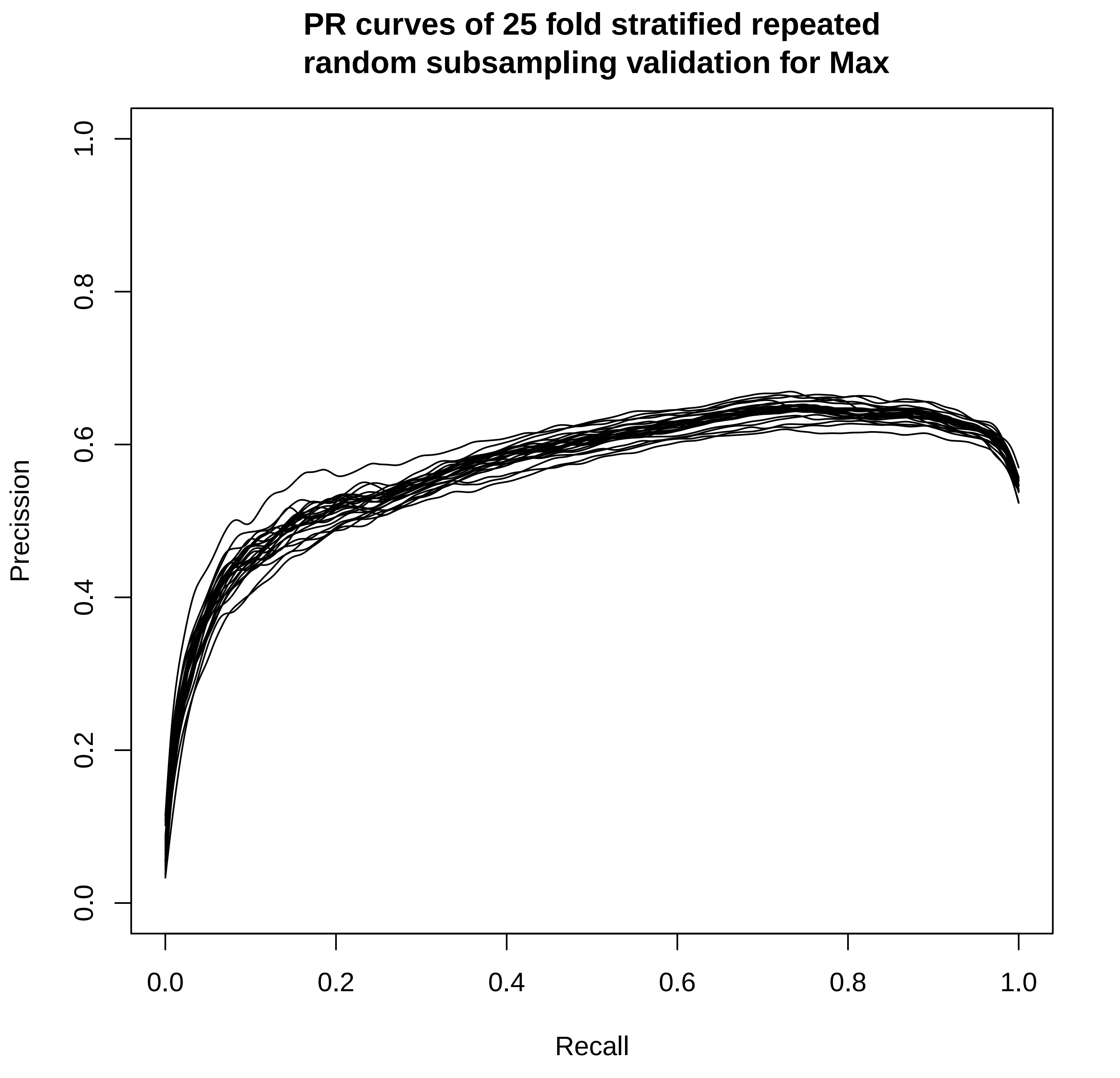
PR curves of 25 fold stratified repeated
random subsampling validation for Max
Precission
Recall
0.0
0.2
0.4
0.6
0.8
1.0

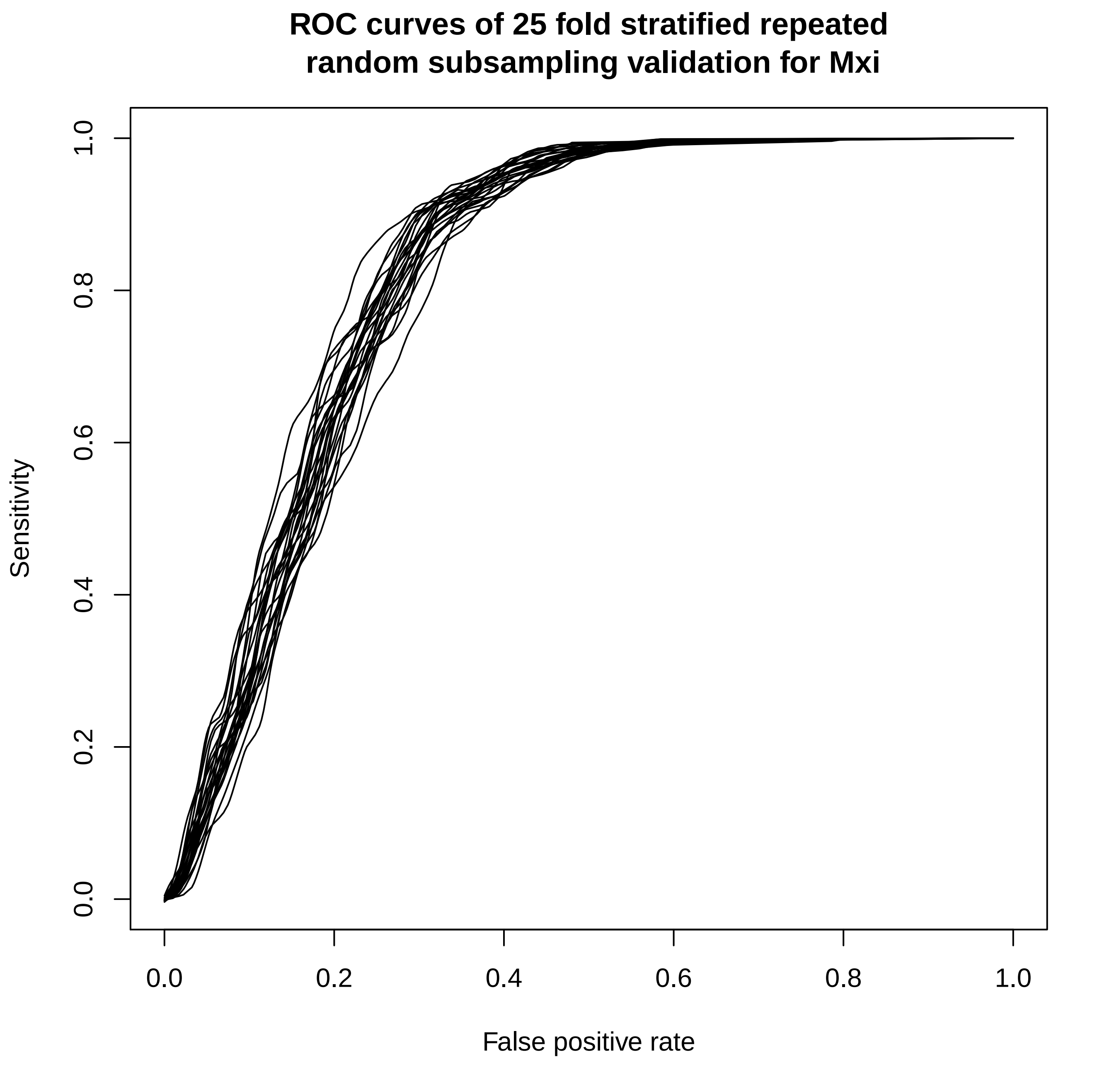
ROC curves of 25 fold stratified repeated
random subsampling validation for Mxi
Sensitivity
0.0
0.2
0.4
0.6
0.8
1.0
False positive rate
0.0
0.2
0.4
0.6
0.8
1.0

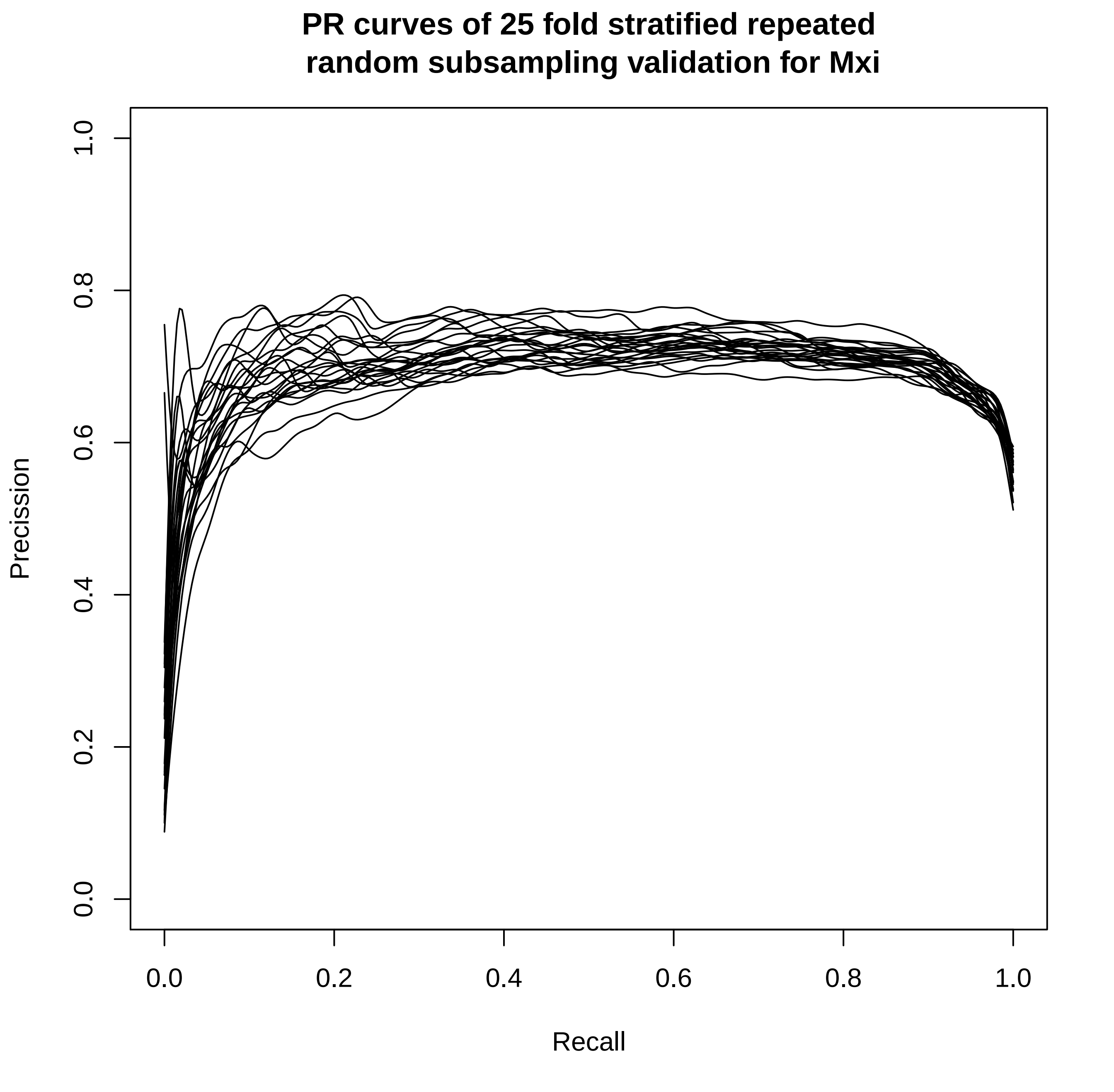
PR curves of 25 fold stratified repeated
random subsampling validation for Mxi
Precission
Recall
0.0
0.2
0.4
0.6
0.8
1.0

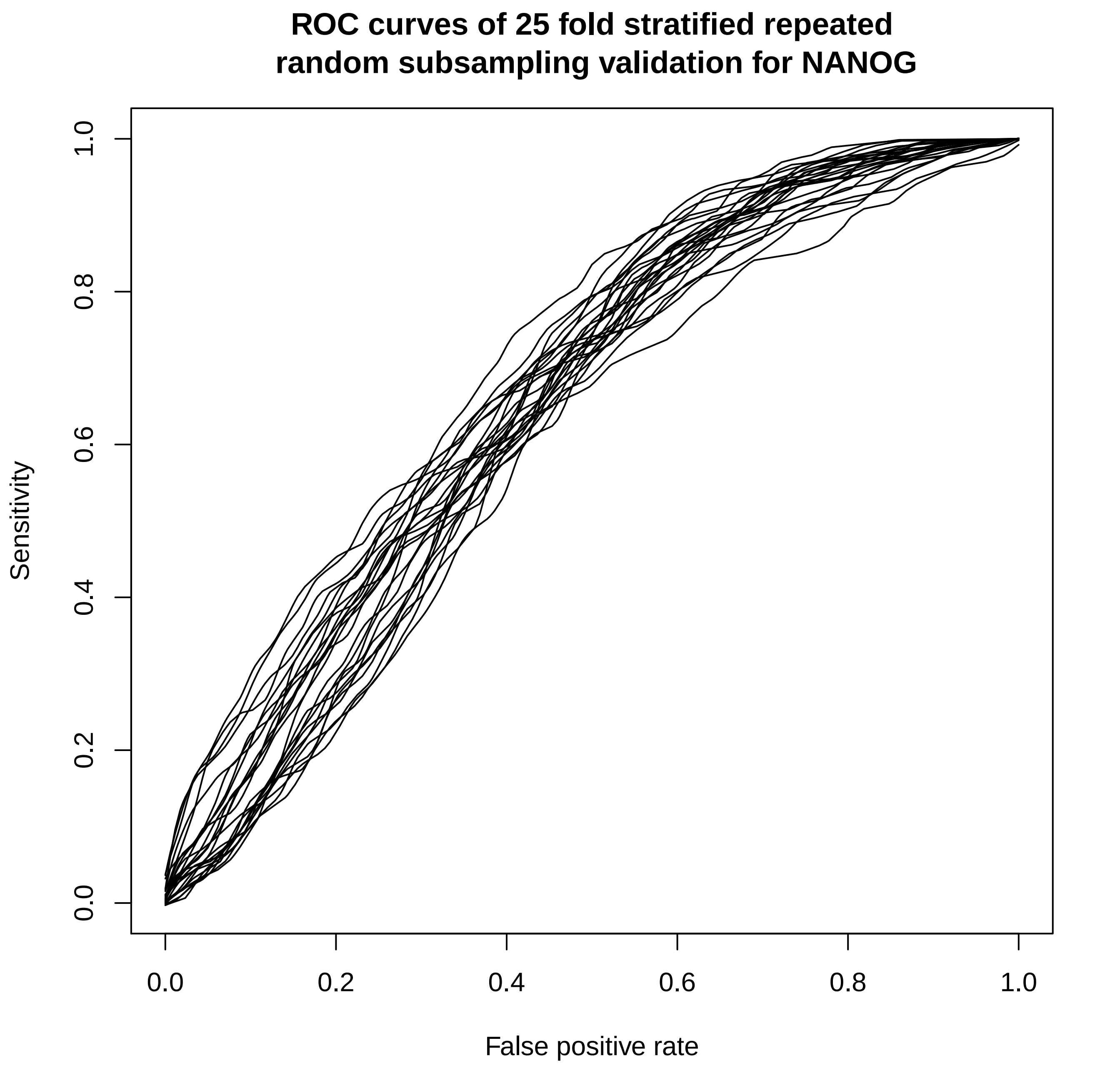
ROC curves of 25 fold stratified repeated random subsampling validation for NANOG
Sensitivity
False positive rate
0.0
0.2
0.4
0.6
0.8
1.0

PR curves of 25 fold stratified repeated random subsampling validation for NANOG

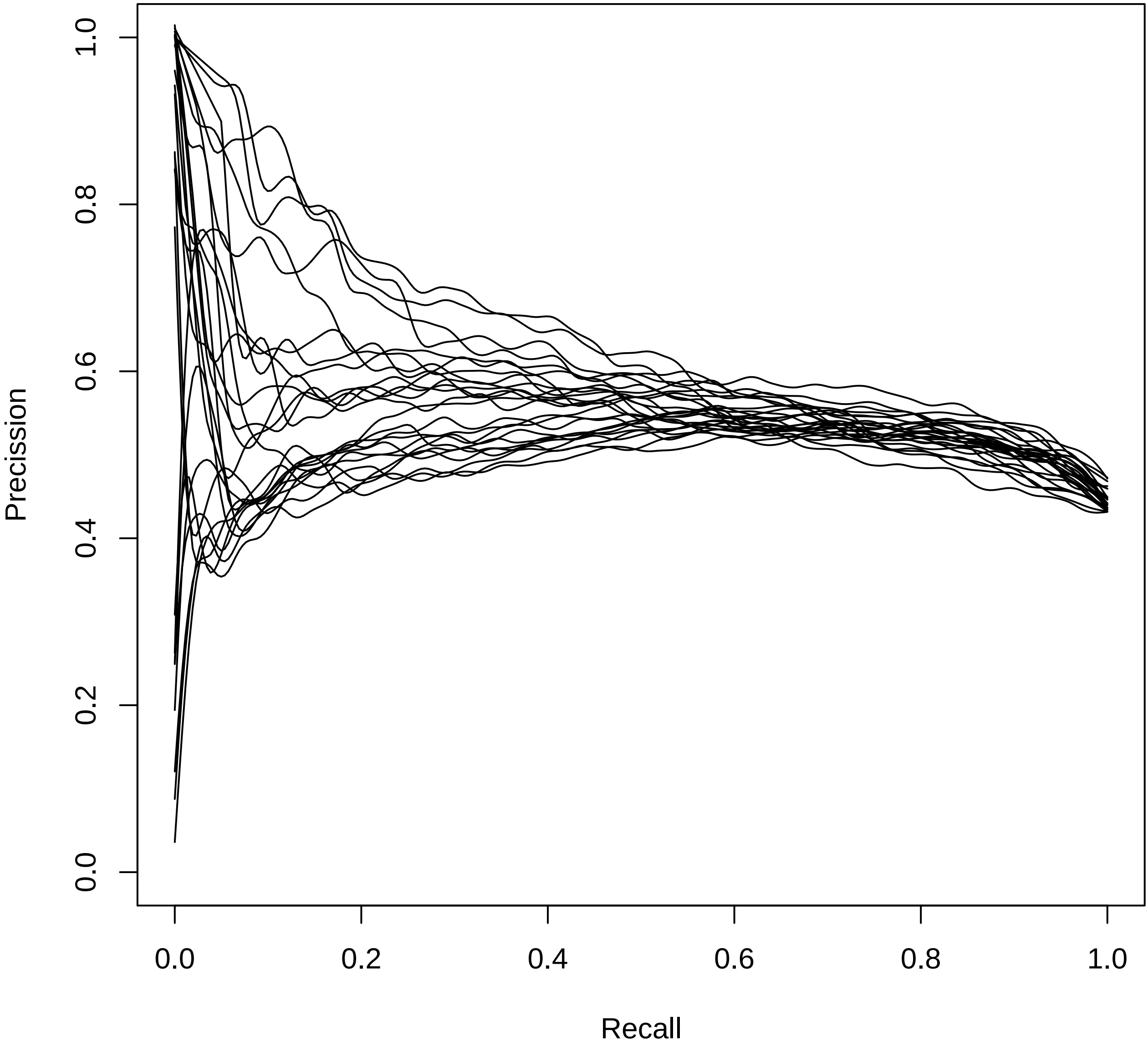

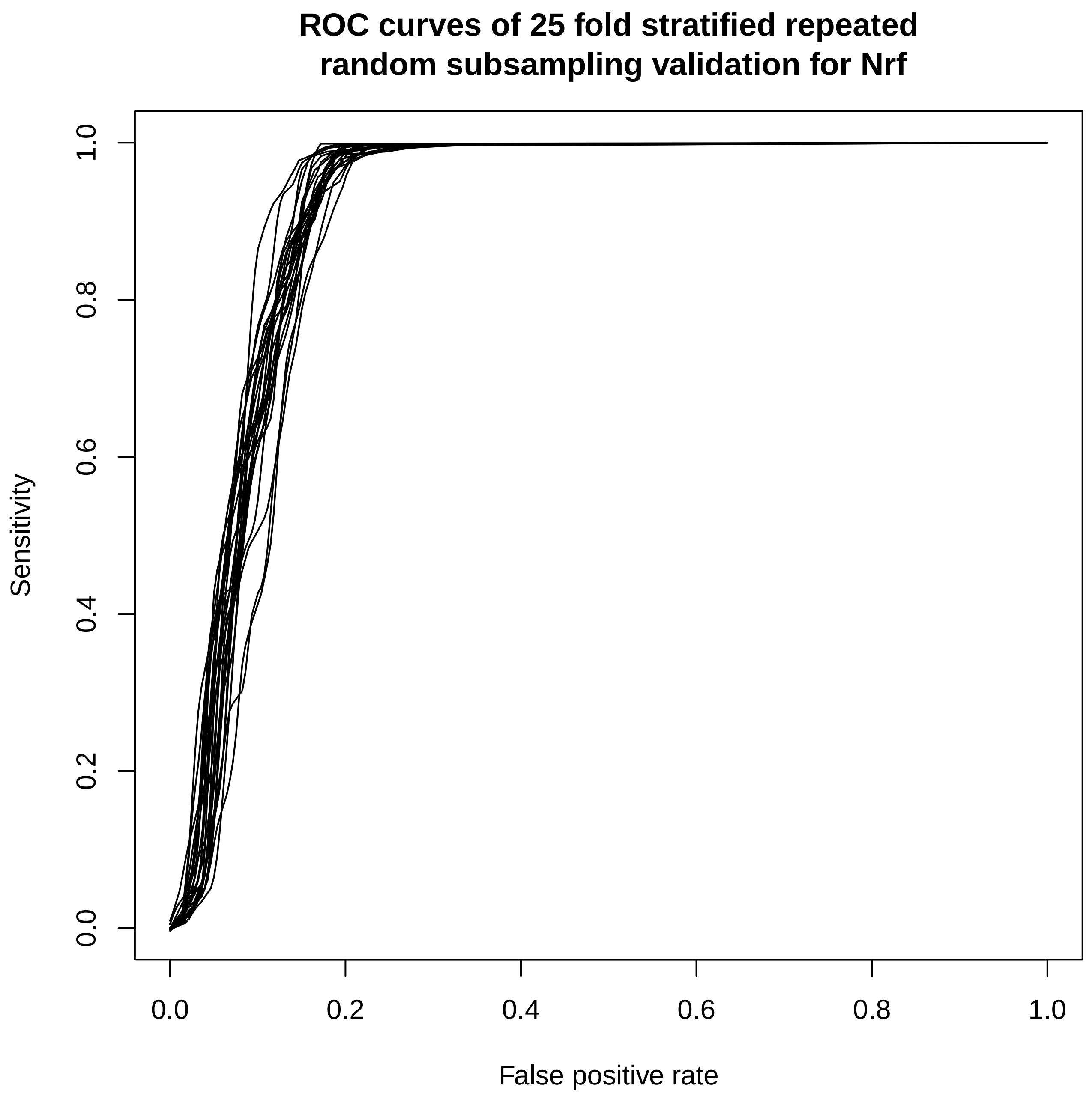

ROC curves of 25 fold stratified repeated random subsampling validation for Nrf
Sensitivity
0.0
0.2
0.4
0.6
0.8
1.0
False positive rate
0.0
0.2
0.4
0.6
0.8
1.0

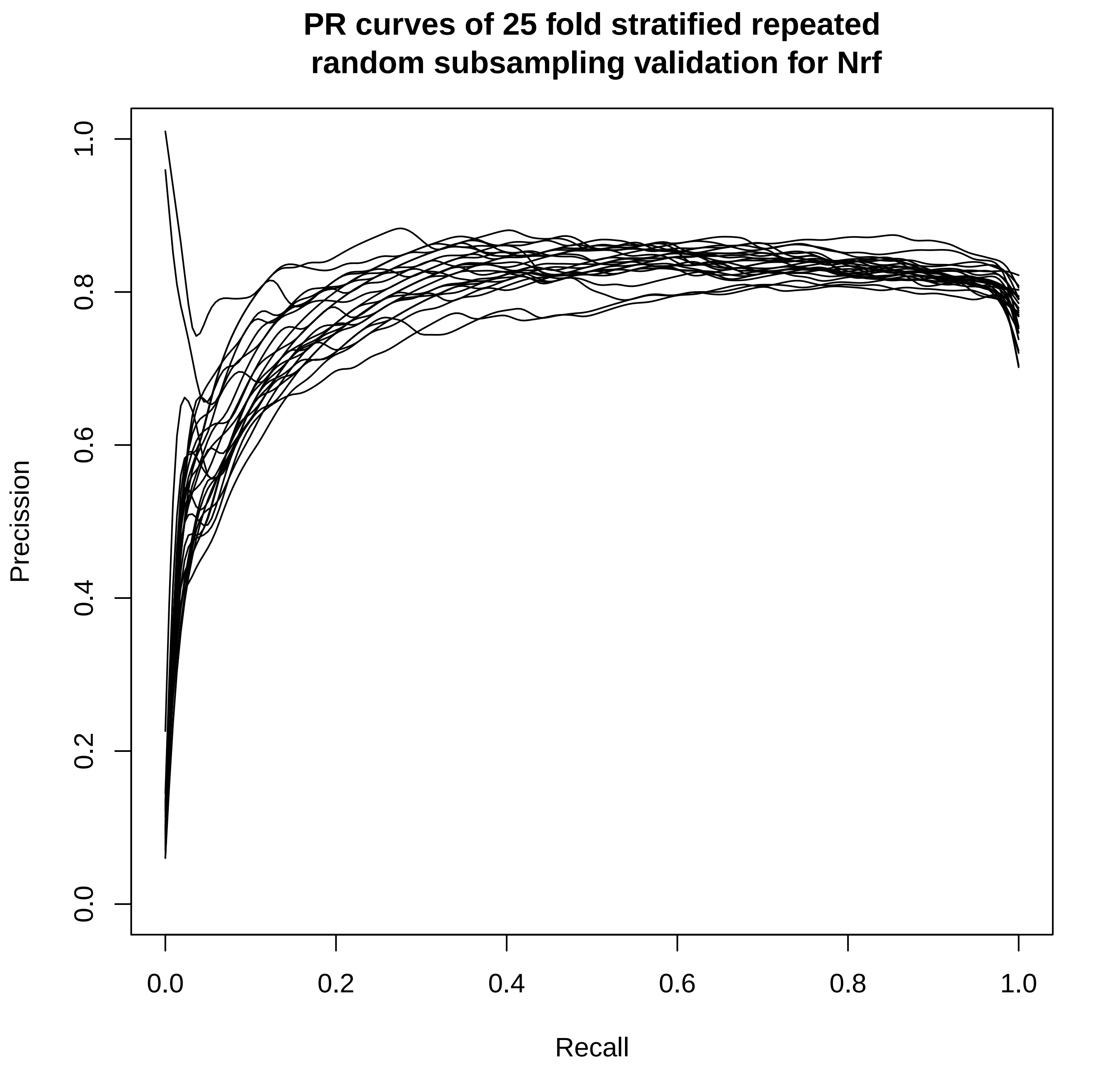
PR curves of 25 fold stratified repeated
random subsampling validation for Nrf
Precission
Recall
0.0
0.2
0.4
0.6
0.8
1.0

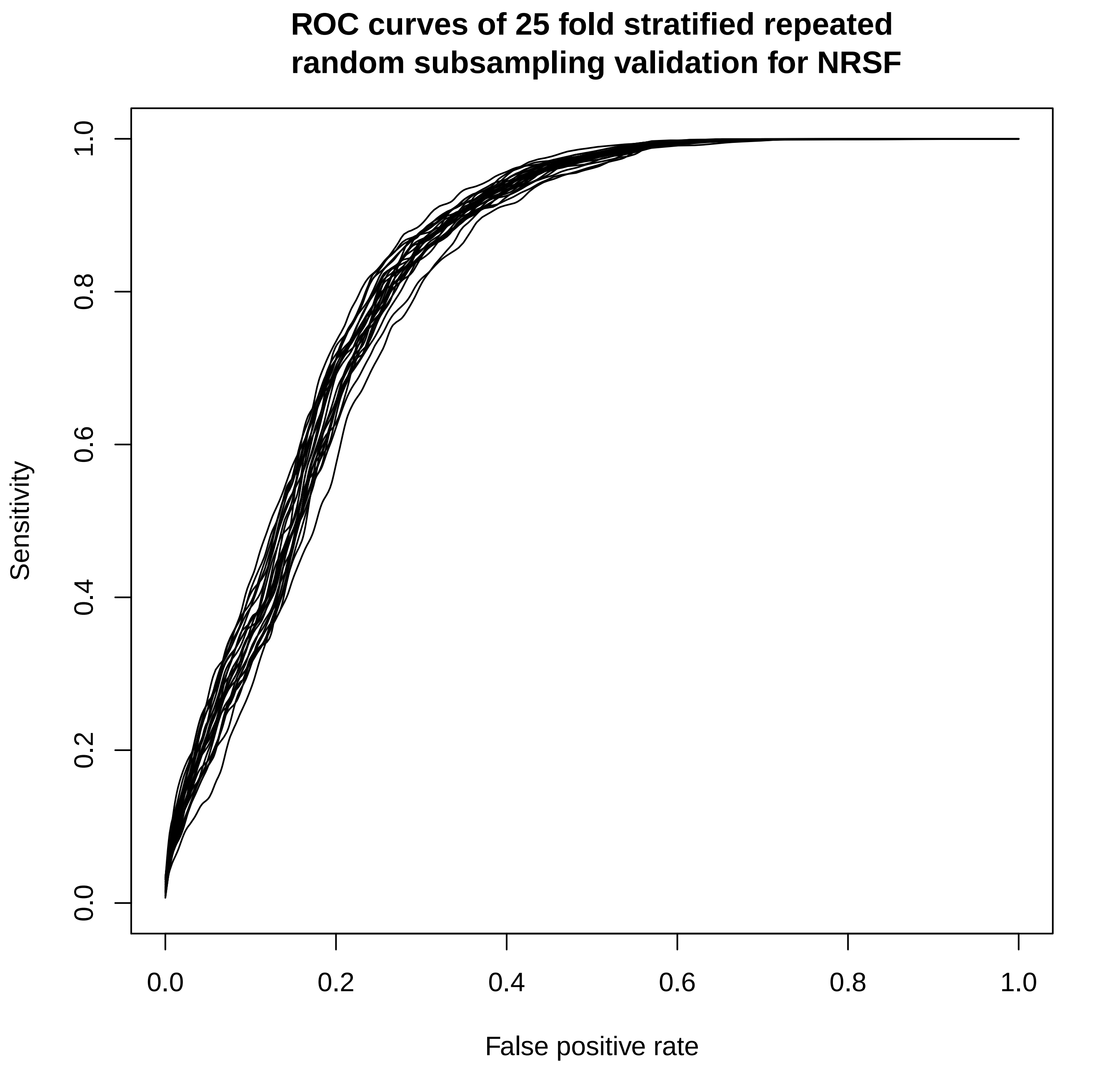
ROC curves of 25 fold stratified repeated
random subsampling validation for NRSF
Sensitivity
0.0
0.2
0.4
0.6
0.8
1.0
False positive rate
0.0
0.2
0.4
0.6
0.8
1.0

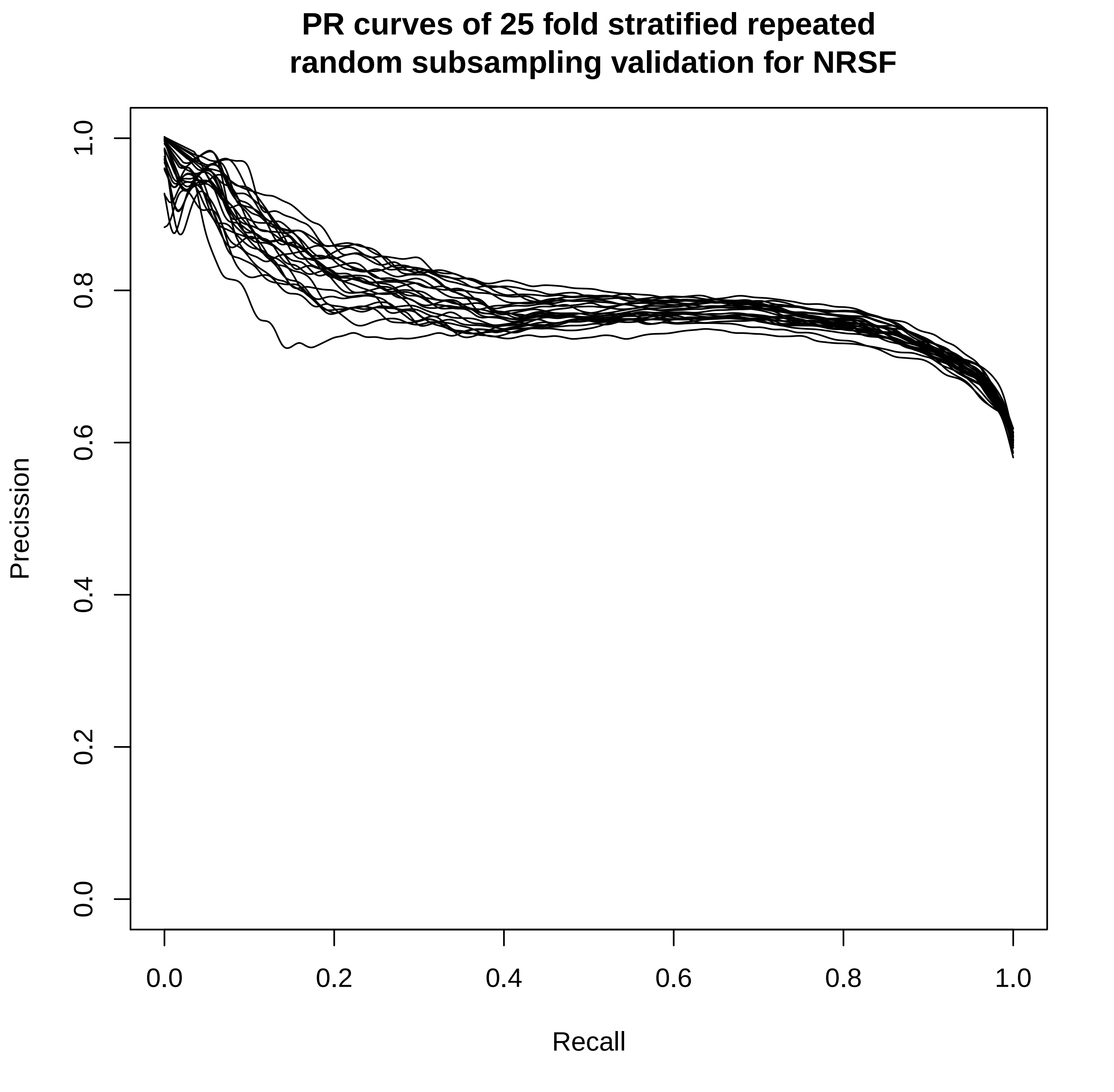

PR curves of 25 fold stratified repeated random subsampling validation for NRSF
Precission
Recall
0.0
0.2
0.4
0.6
0.8
1.0

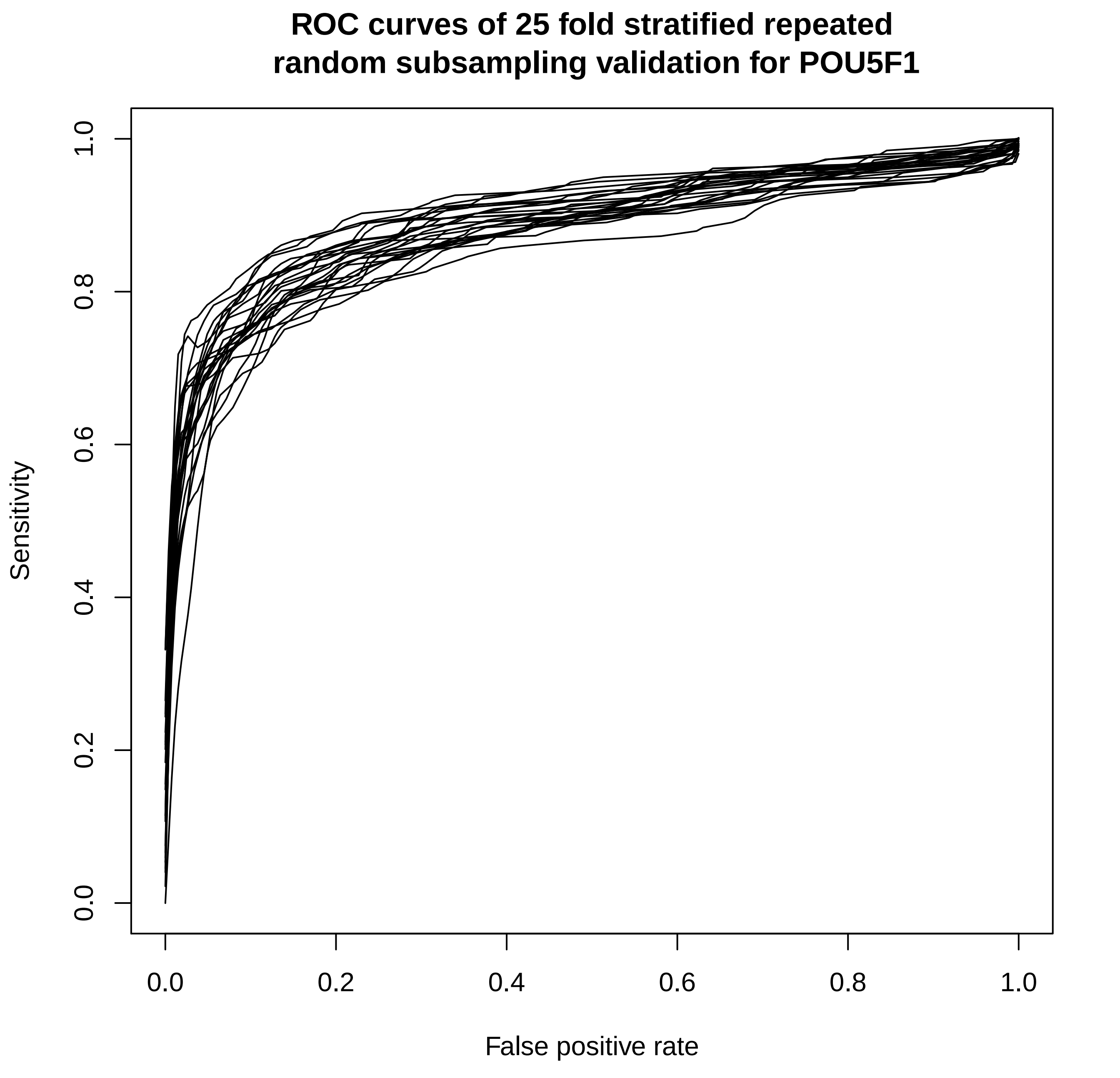
ROC curves of 25 fold stratified repeated
random subsampling validation for POU5F1
Sensitivity
False positive rate
0.0
0.2
0.4
0.6
0.8
1.0

PR curves of 25 fold stratified repeated random subsampling validation for POU5F1

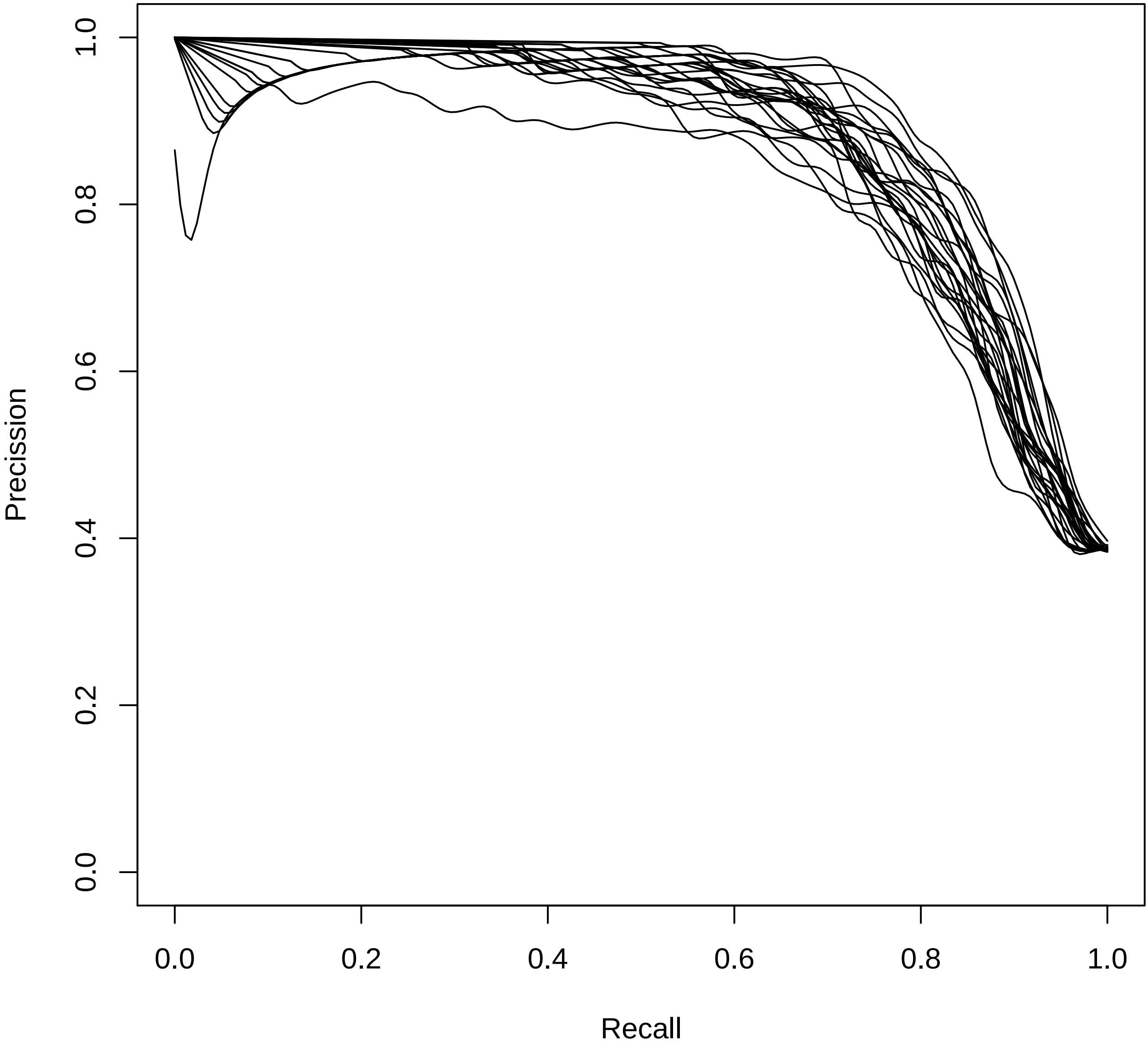

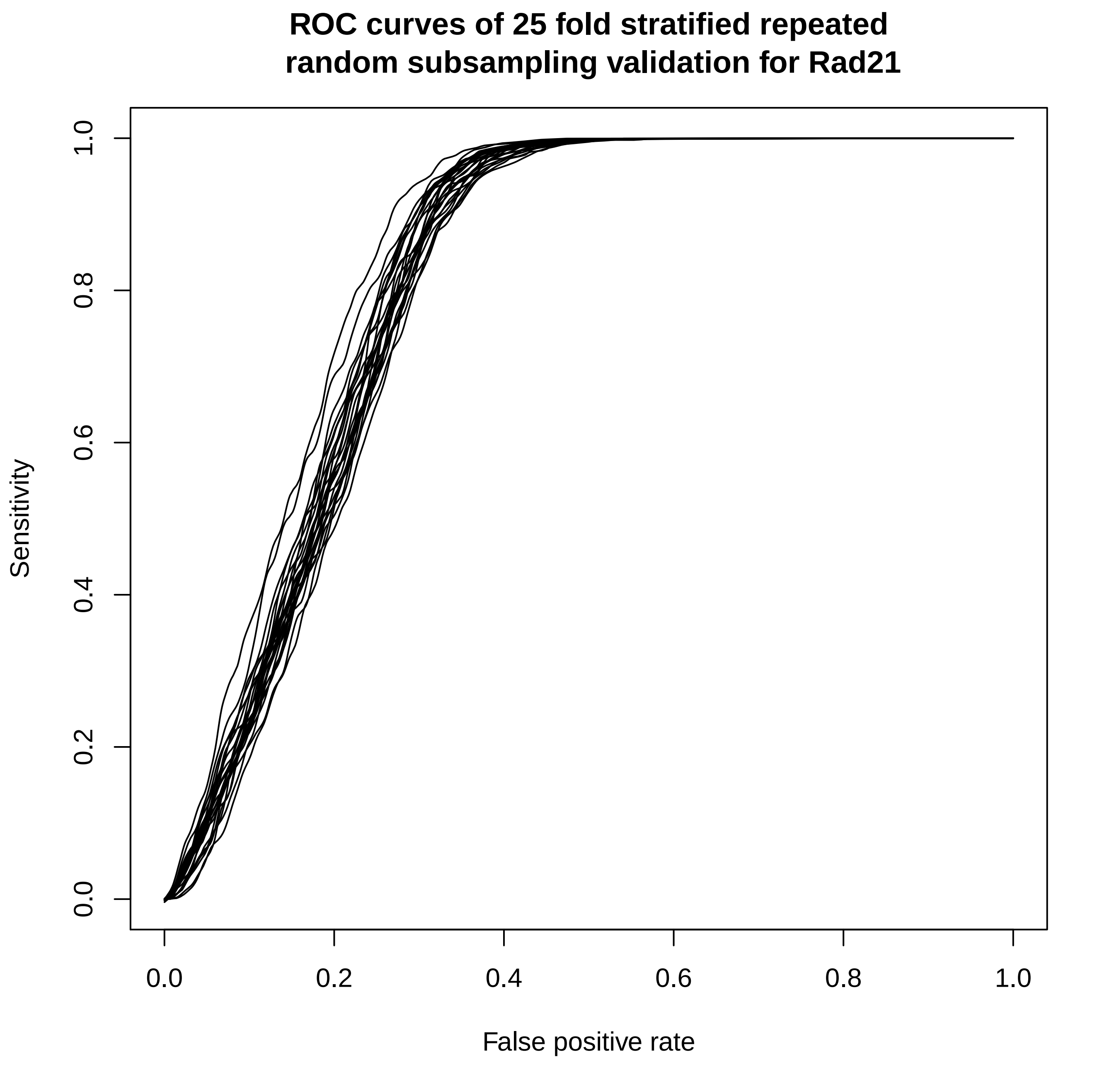
ROC curves of 25 fold stratified repeated
random subsampling validation for Rad21
Sensitivity
False positive rate
0.0
0.2
0.4
0.6
0.8
1.0

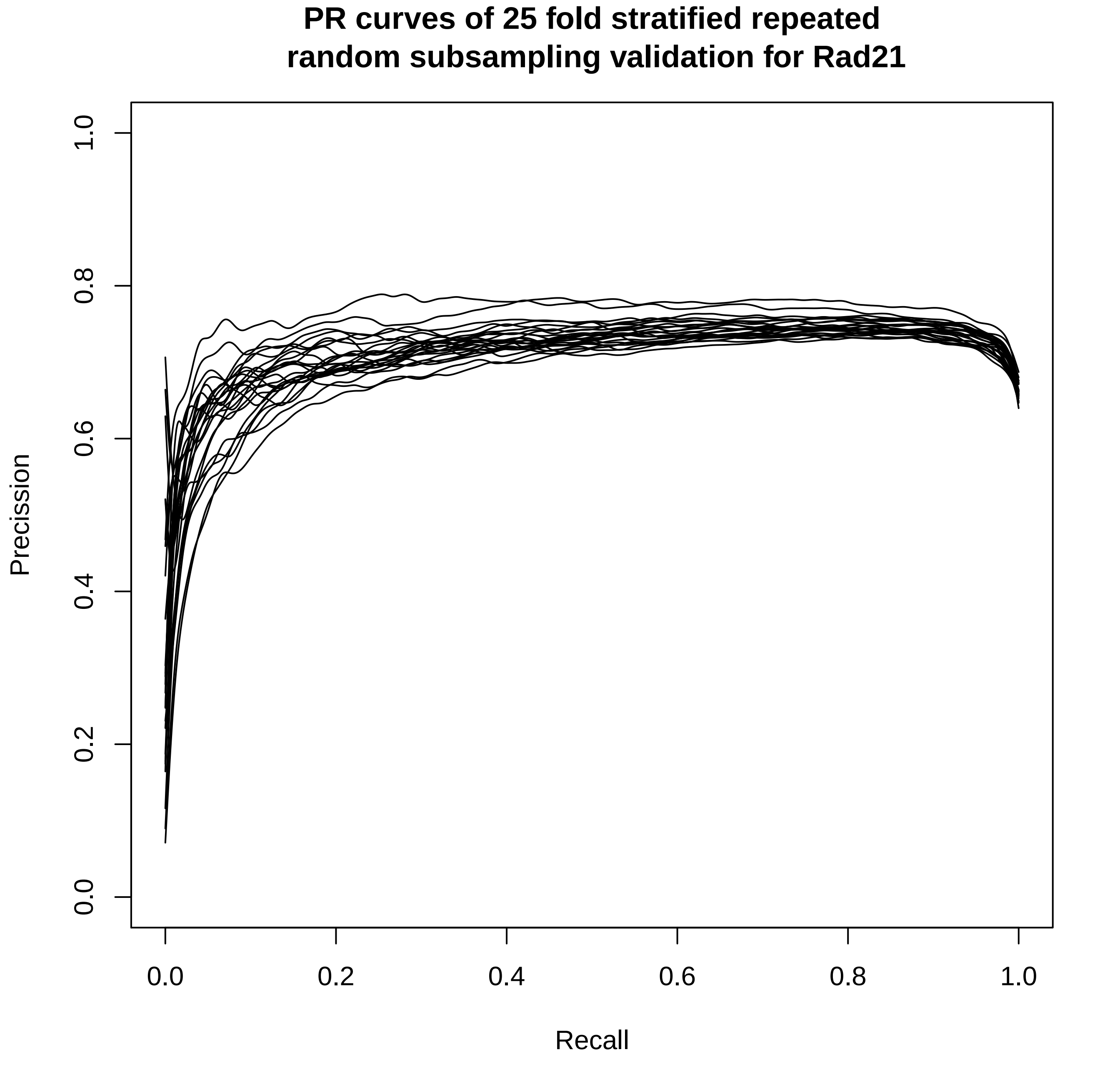

PR curves of 25 fold stratified repeated random subsampling validation for Rad21
Precission
Recall
0.0
0.2
0.4
0.6
0.8
1.0
0.0
0.2
0.4
0.6
0.8
1.0

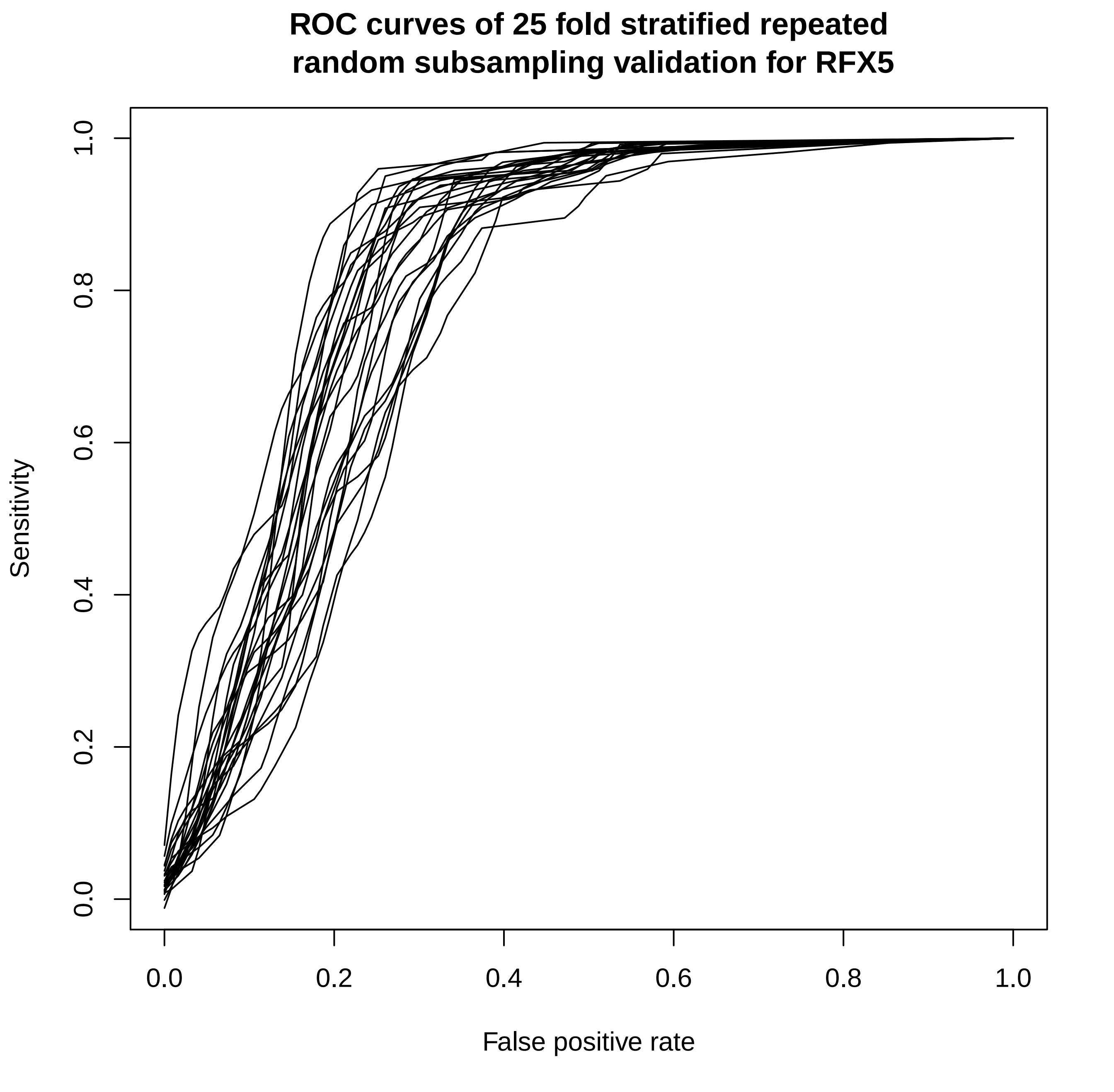
ROC curves of 25 fold stratified repeated
random subsampling validation for RFX5
Sensitivity
0.0
0.2
0.4
0.6
0.8
1.0
False positive rate

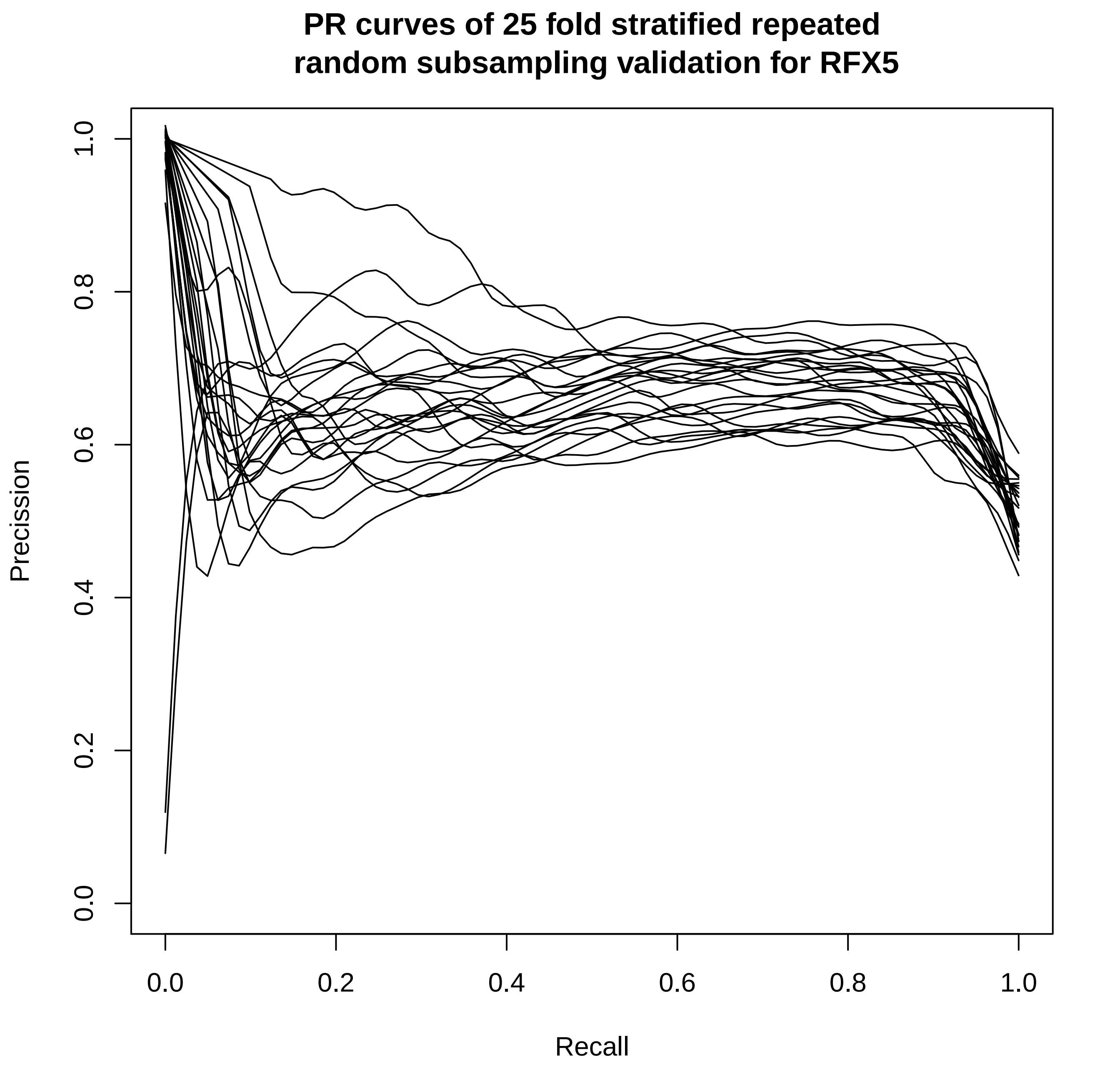
PR curves of 25 fold stratified repeated
random subsampling validation for RFX5
Precission
Recall
0.0
0.2
0.4
0.6
0.8
1.0

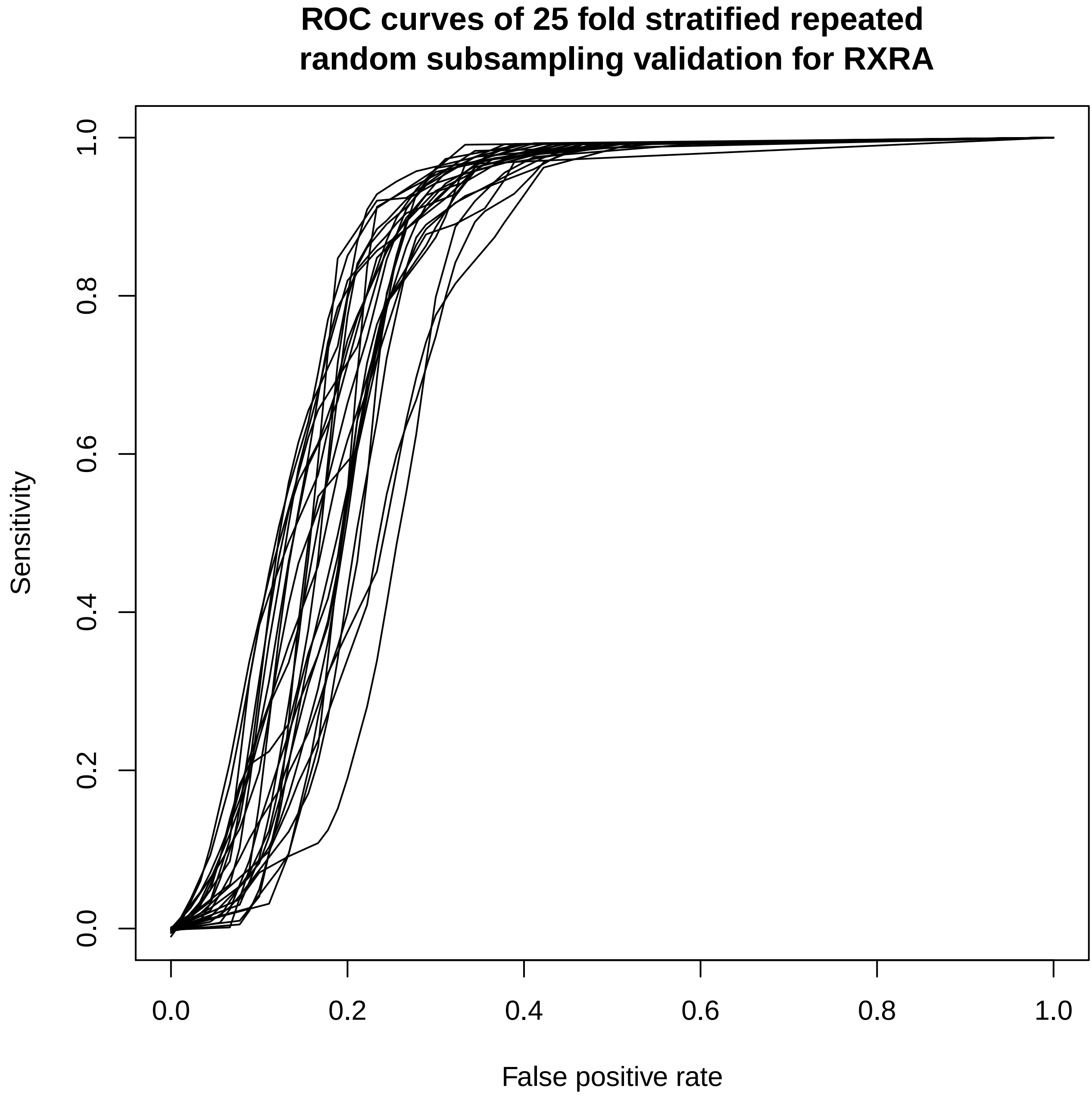
ROC curves of 25 fold stratified repeated
random subsampling validation for RXRA
Sensitivity
0.0
0.2
0.4
0.6
0.8
1.0
False positive rate
0.0
0.2
0.4
0.6
0.8
1.0

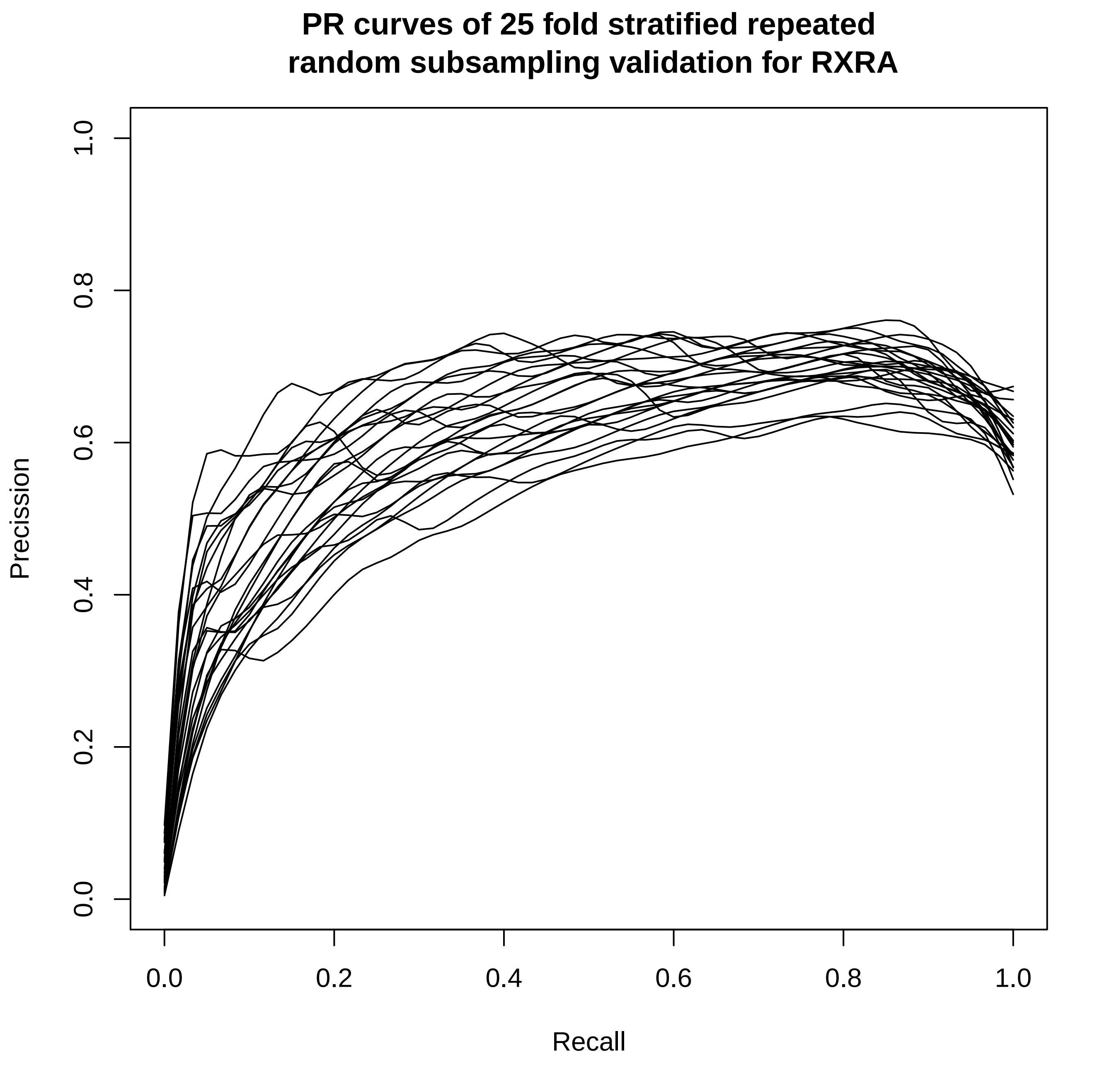
PR curves of 25 fold stratified repeated
random subsampling validation for RXRA
Precission
Recall
0.0
0.2
0.4
0.6
0.8
1.0

ROC curves of 25 fold stratified repeated random subsampling validation for Sin3Ak

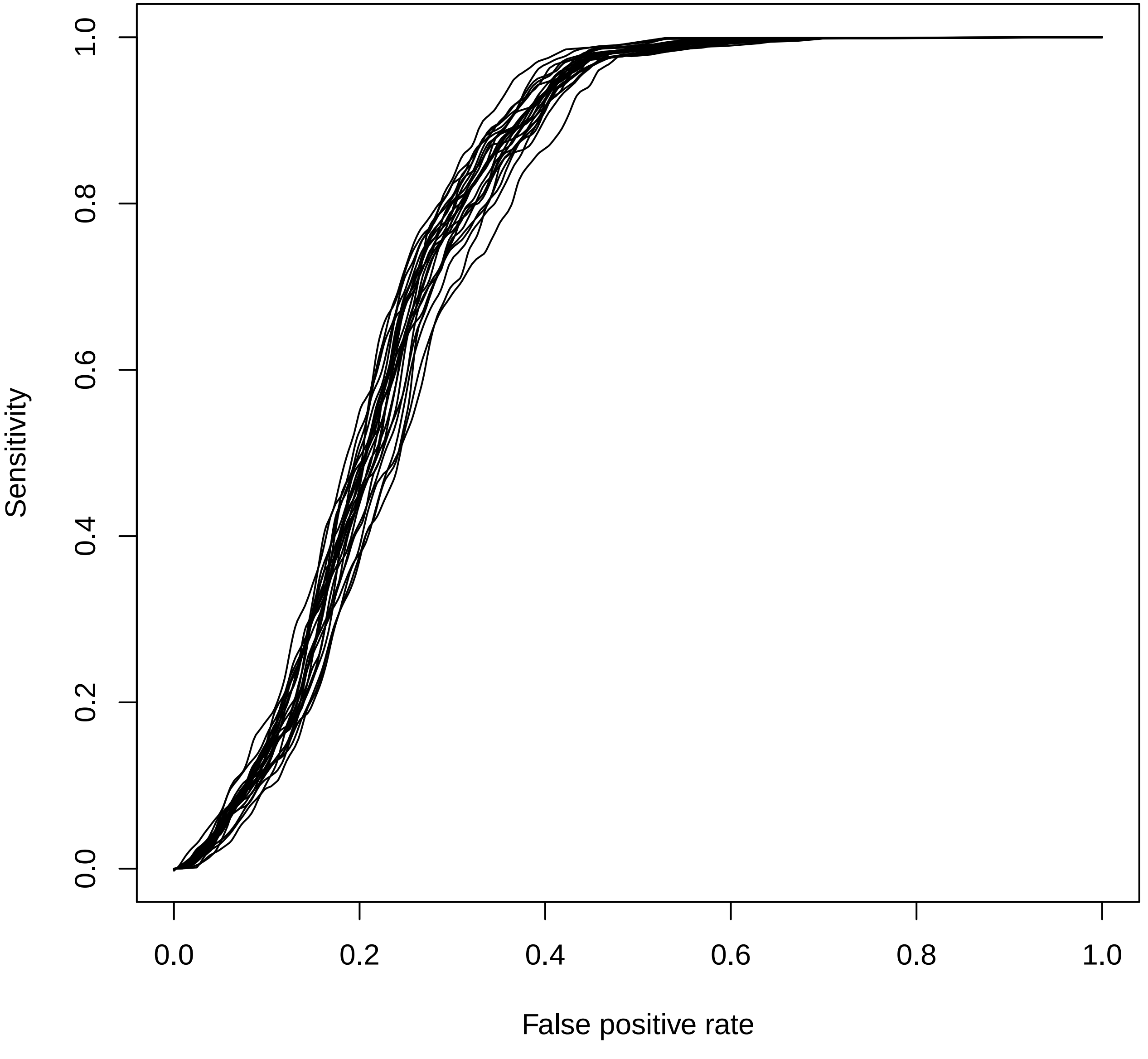

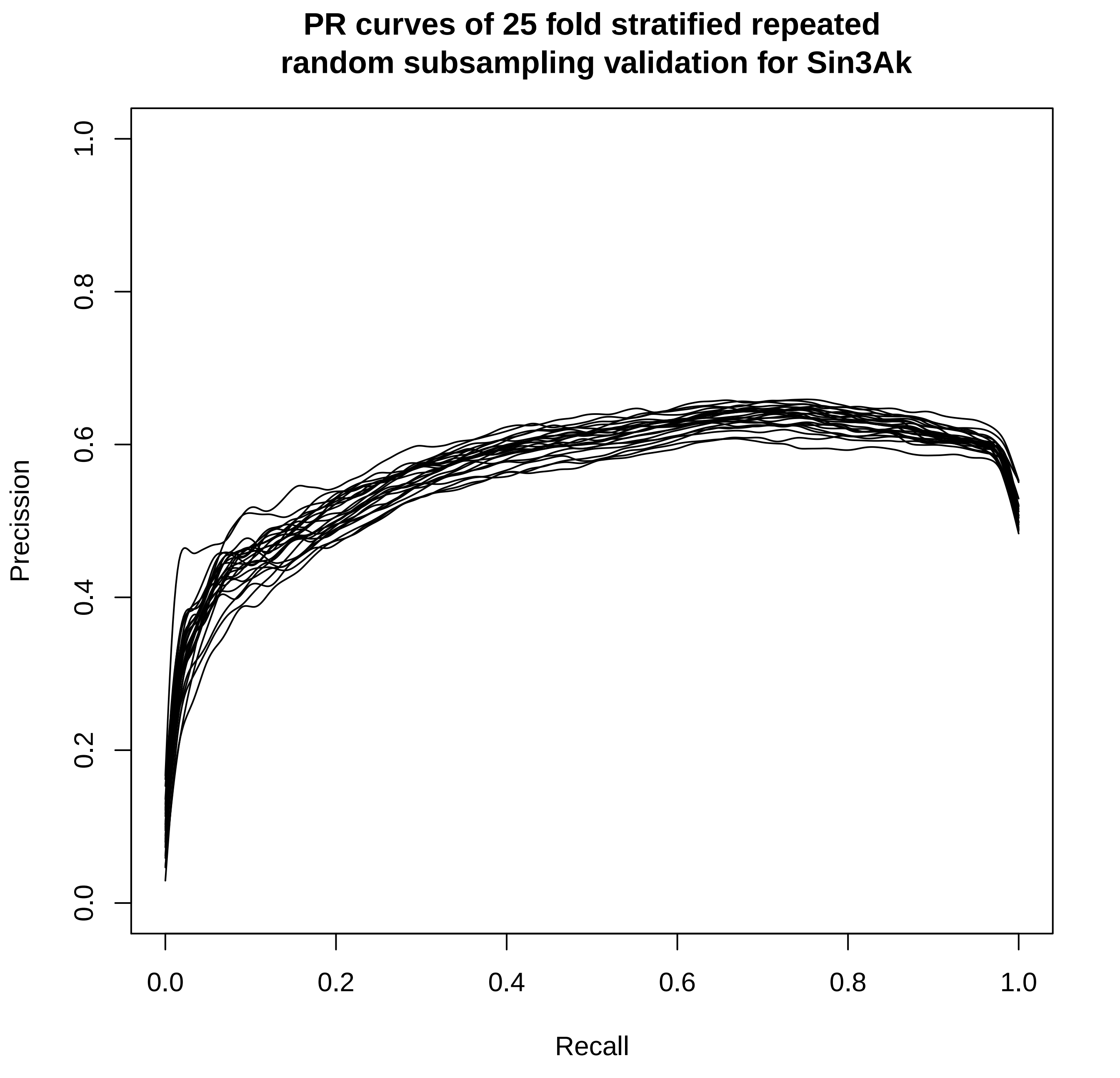

PR curves of 25 fold stratified repeated
random subsampling validation for Sin3Ak
Precission
Recall
0.0
0.2
0.4
0.6
0.8
1.0

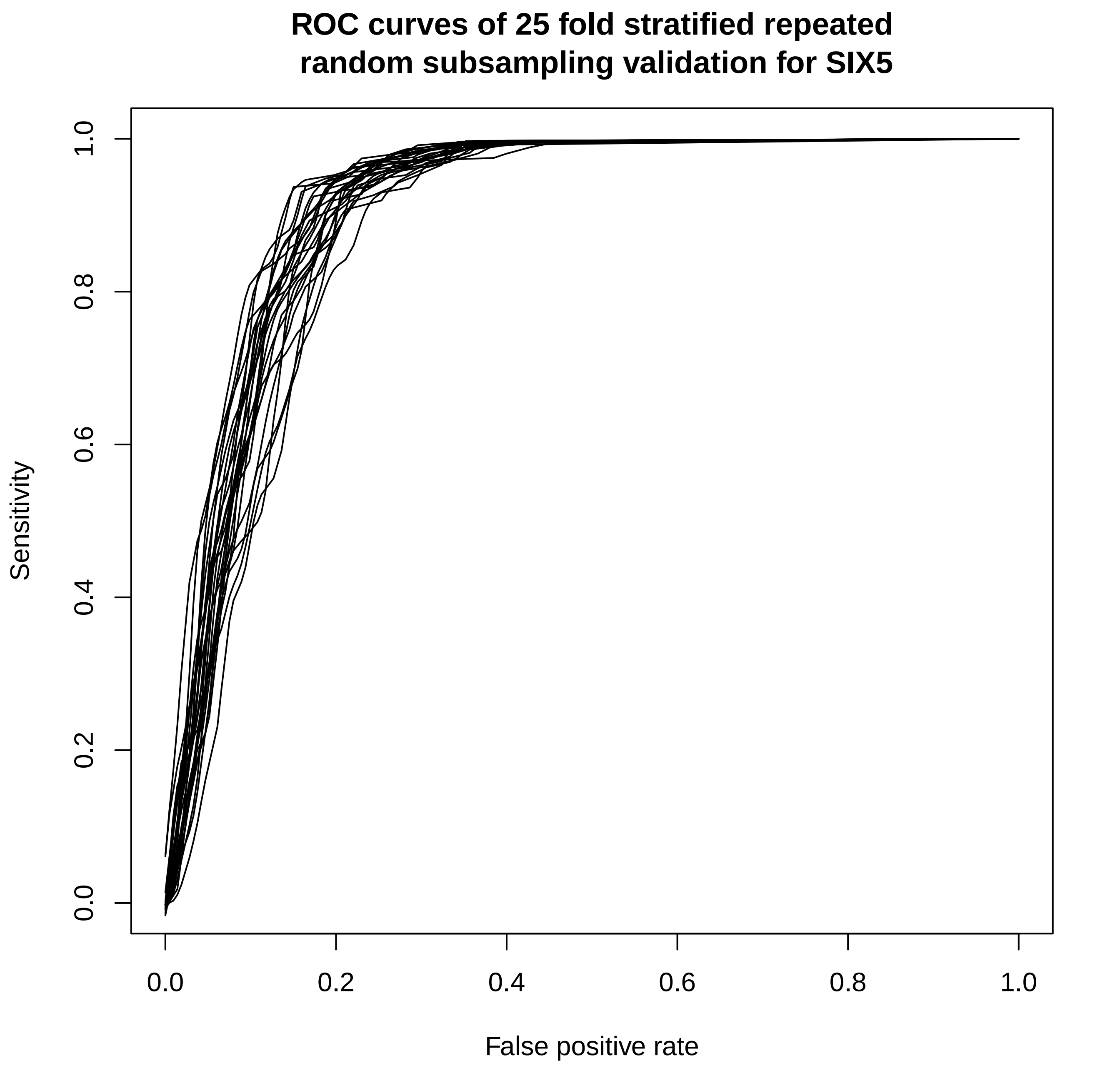
ROC curves of 25 fold stratified repeated
random subsampling validation for SIX5
Sensitivity
False positive rate
0.0
0.2
0.4
0.6
0.8
1.0

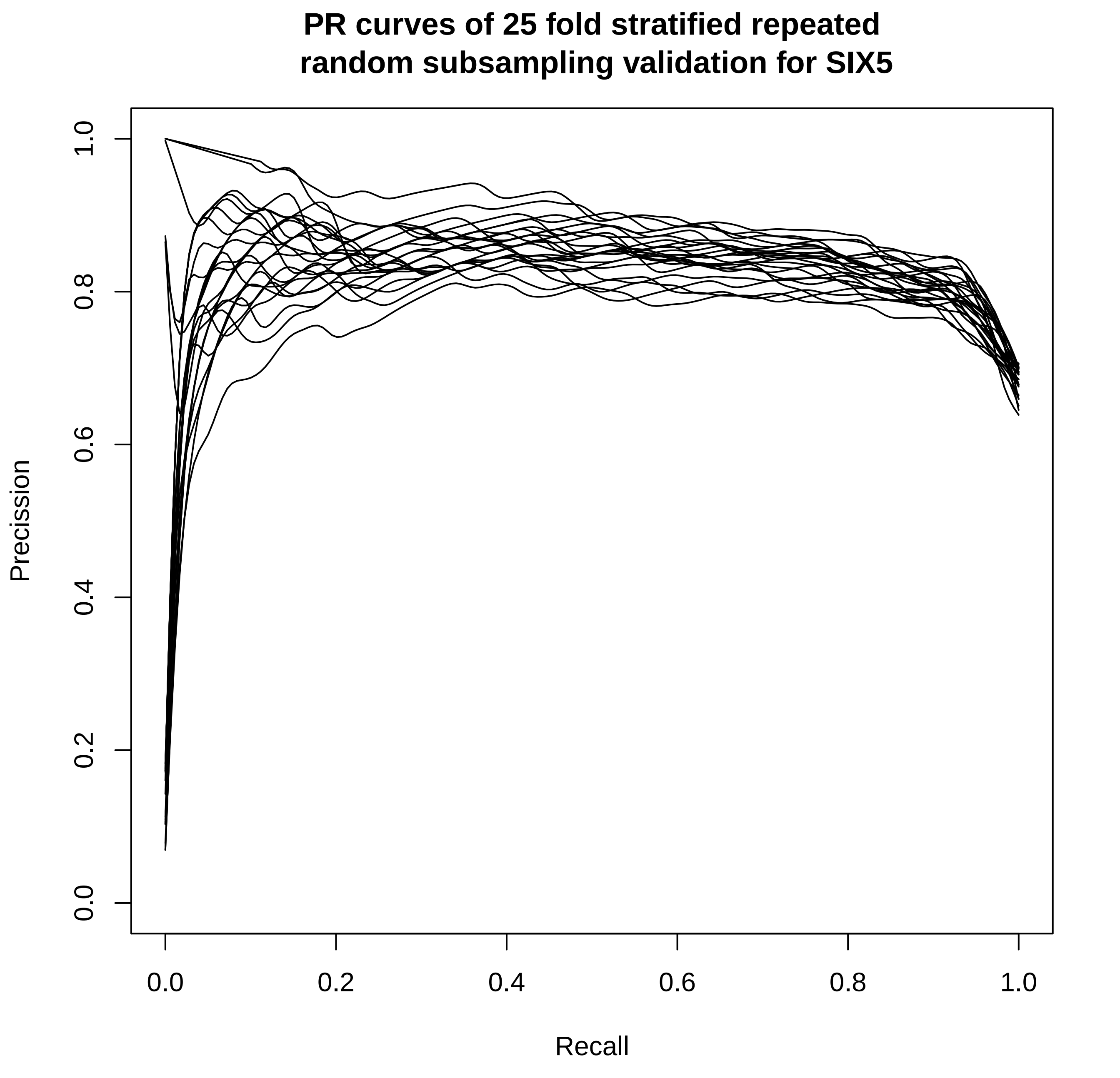
PR curves of 25 fold stratified repeated
random subsampling validation for SIX5
Precission
Recall
0.0
0.2
0.4
0.6
0.8
1.0

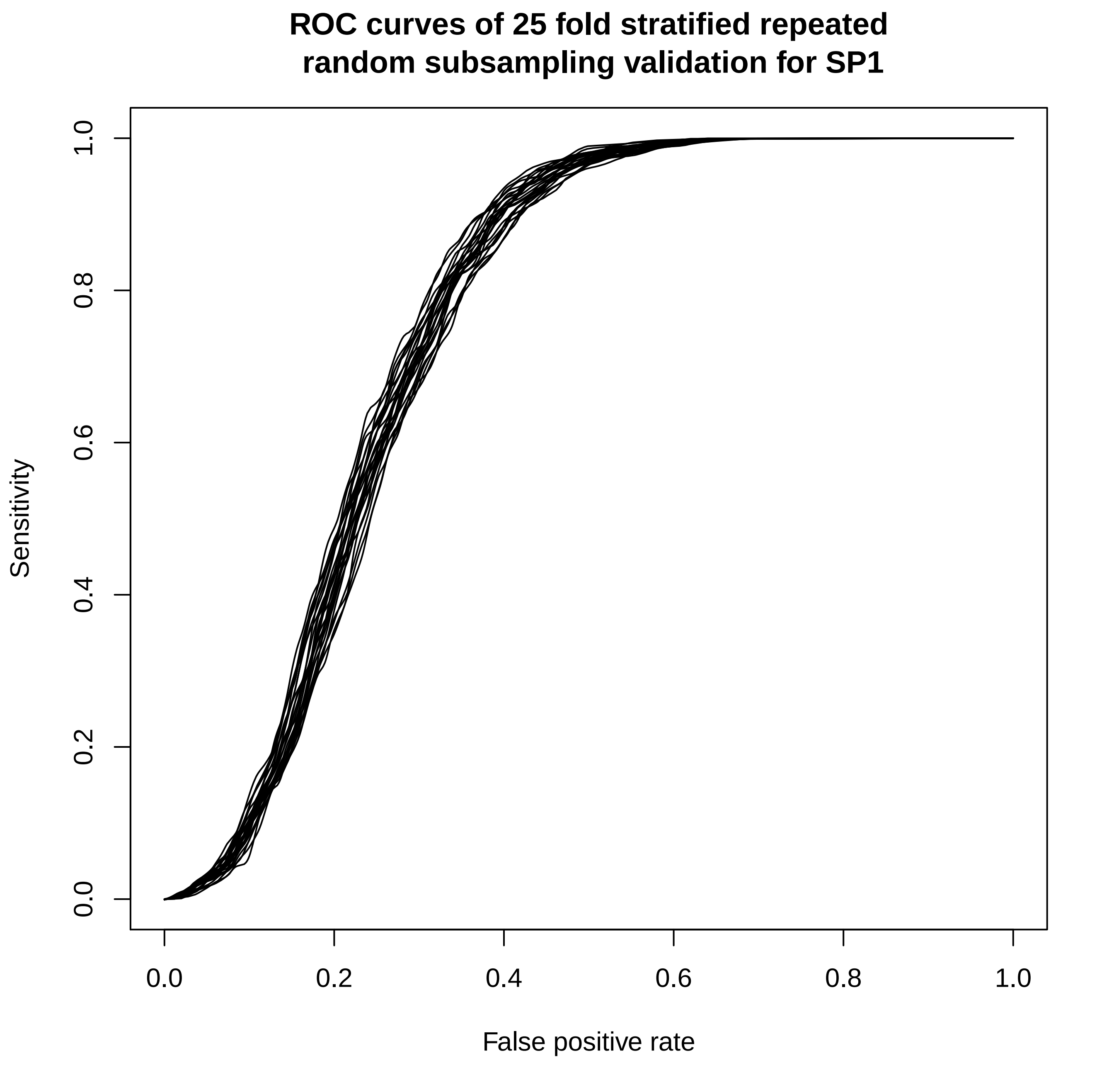

ROC curves of 25 fold stratified repeated
random subsampling validation for SP1
Sensitivity
False positive rate
0.0
0.2
0.4
0.6
0.8
1.0

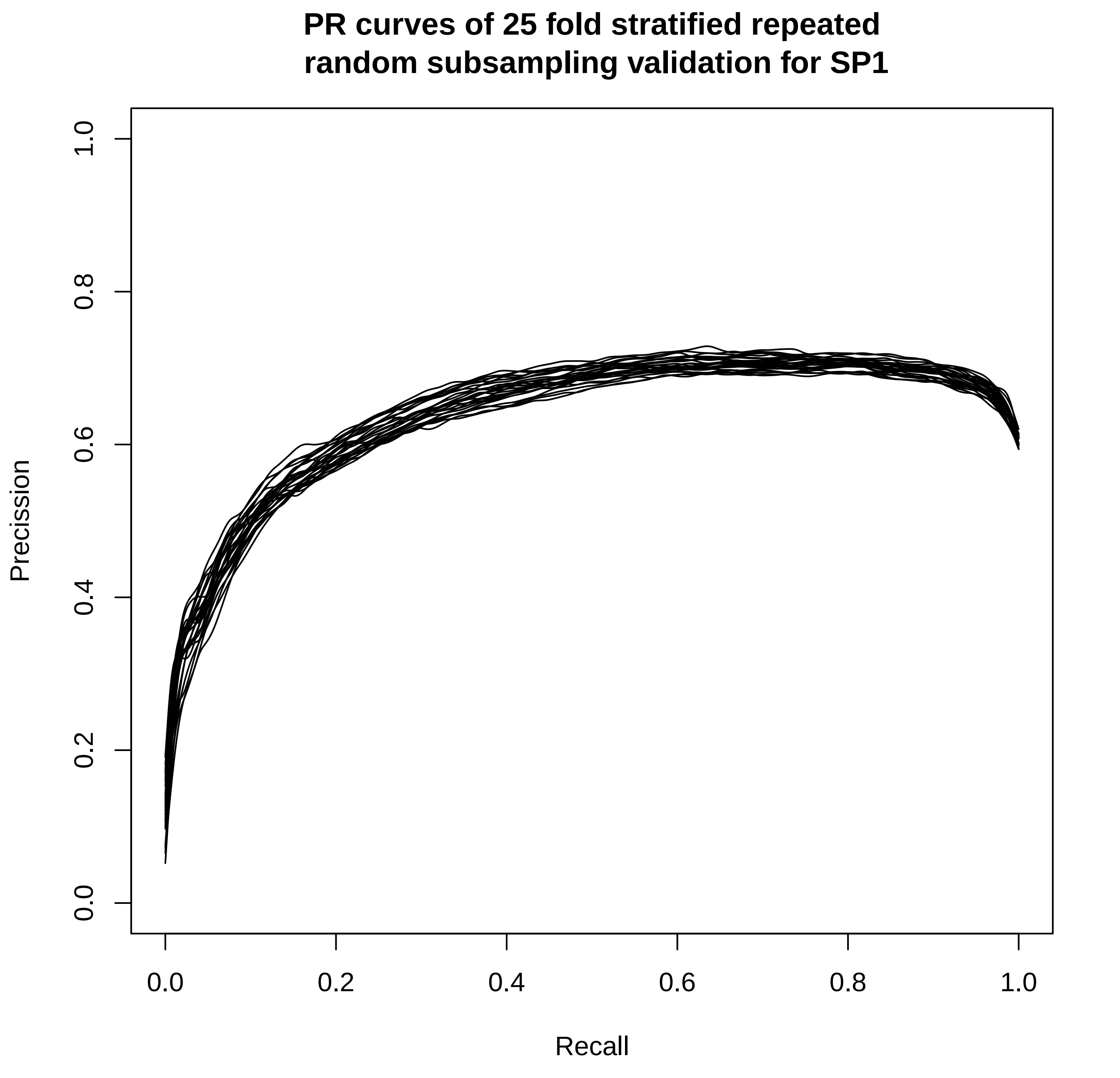
PR curves of 25 fold stratified repeated
random subsampling validation for SP1
Precission
Recall
0.0
0.2
0.4
0.6
0.8
1.0

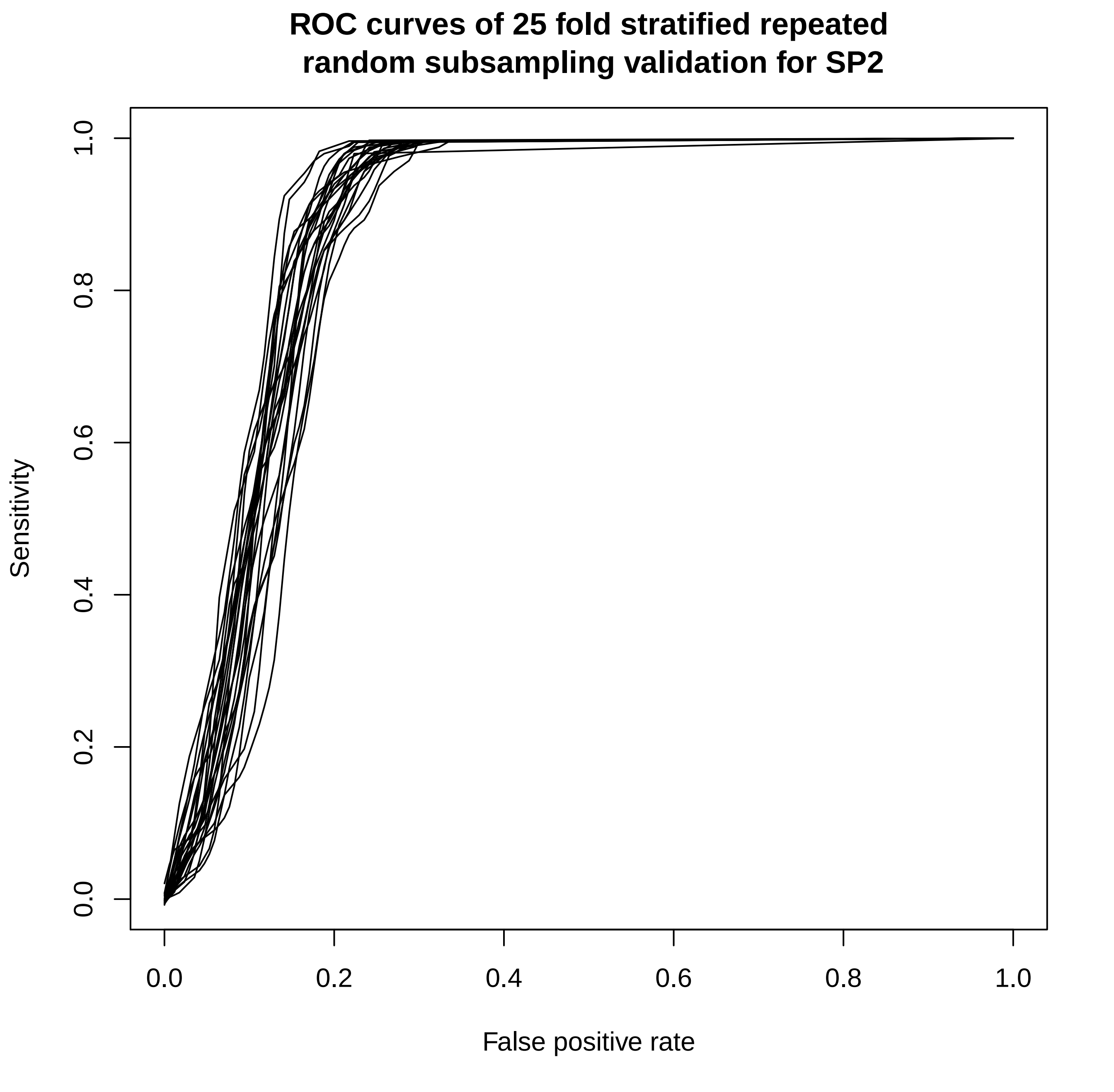
ROC curves of 25 fold stratified repeated
random subsampling validation for SP2
Sensitivity
False positive rate
0.0
0.2
0.4
0.6
0.8
1.0
0.0
0.2
0.4
0.6
0.8
1.0

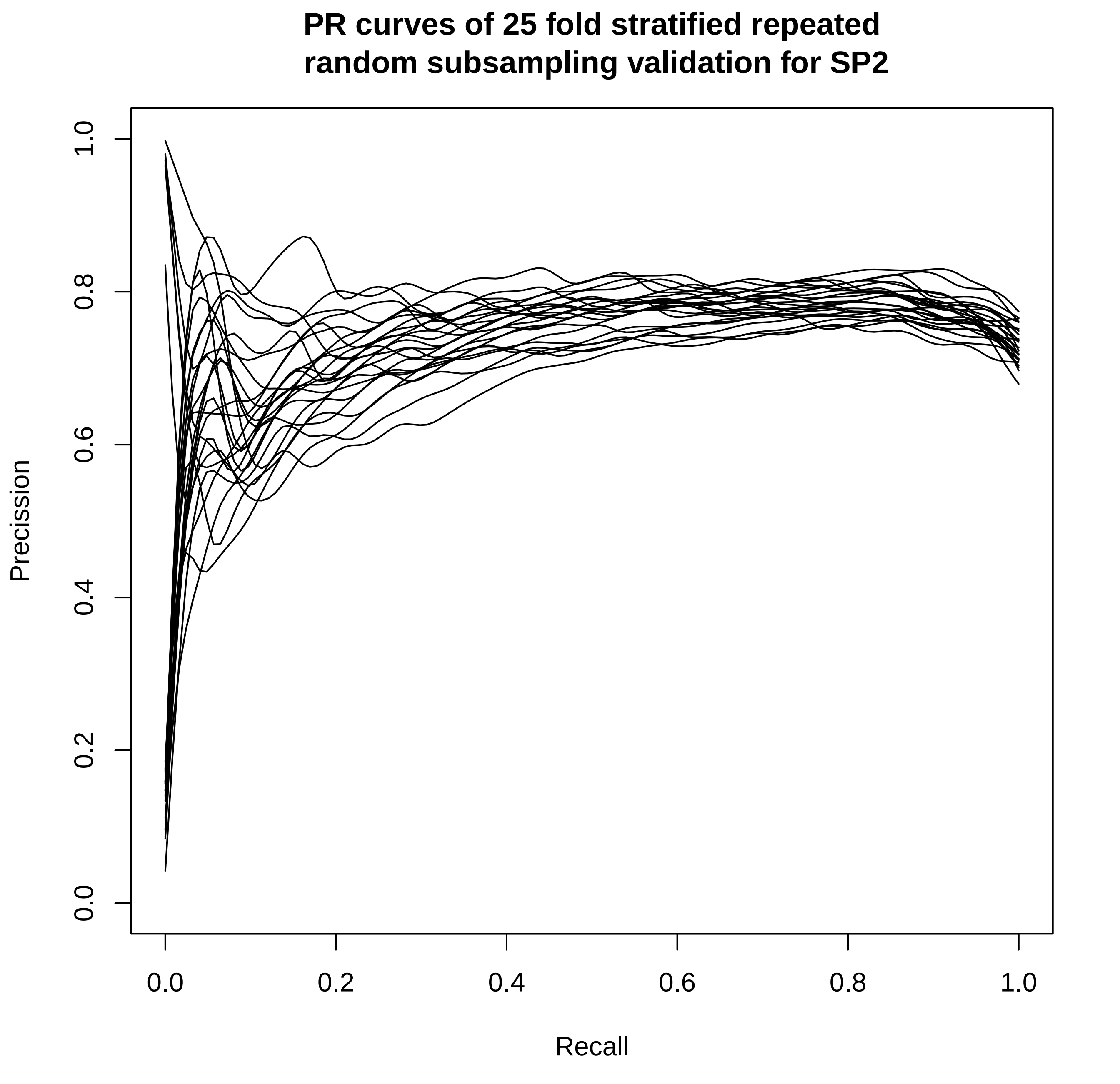
PR curves of 25 fold stratified repeated random subsampling validation for SP2
Precission
Recall
0.0
0.2
0.4
0.6
0.8
1.0

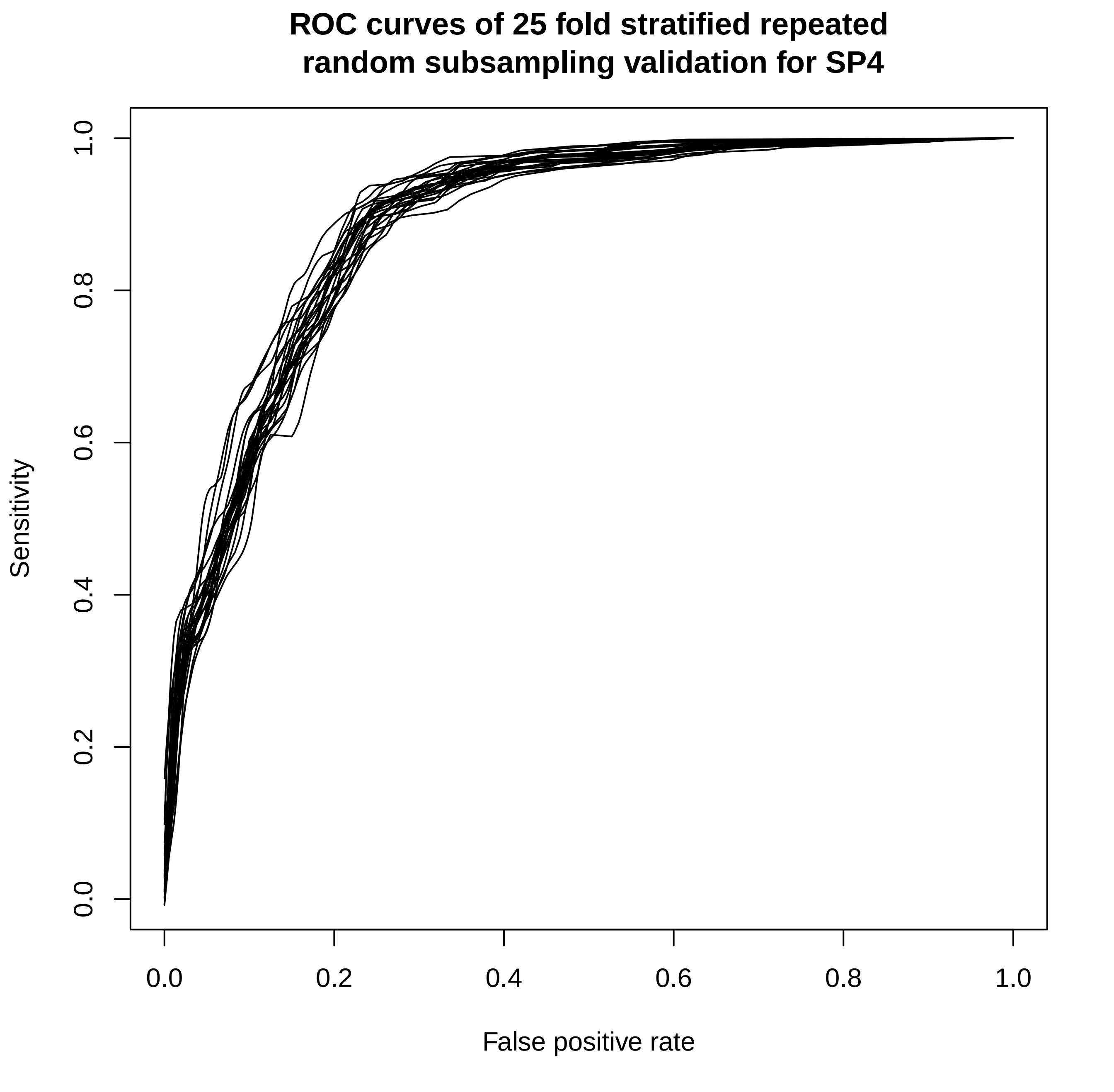

ROC curves of 25 fold stratified repeated
random subsampling validation for SP4
Sensitivity
0.0
0.2
0.4
0.6
0.8
1.0
False positive rate

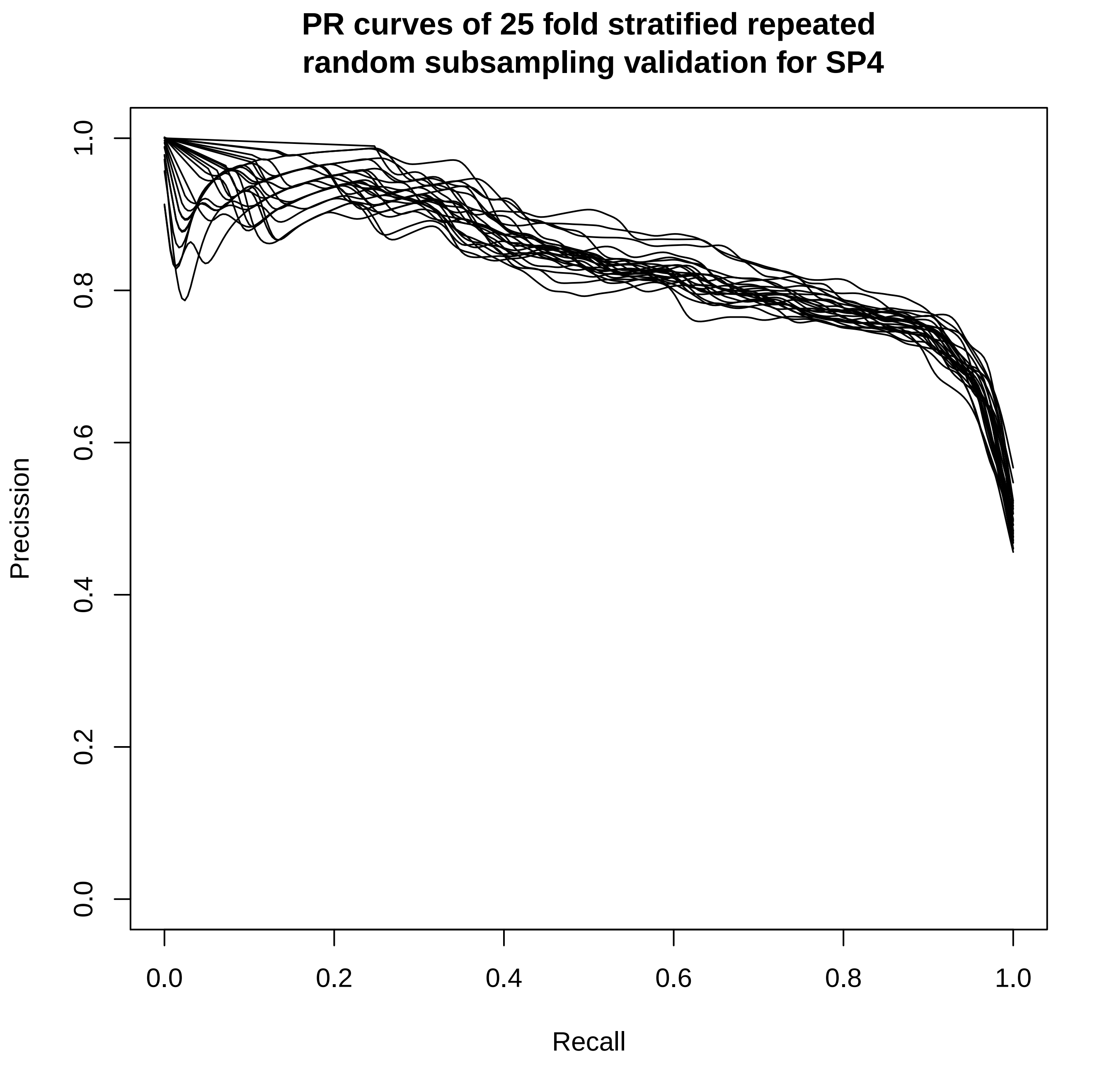
PR curves of 25 fold stratified repeated
random subsampling validation for SP4
Precission
Recall
0.0
0.2
0.4
0.6
0.8
1.0

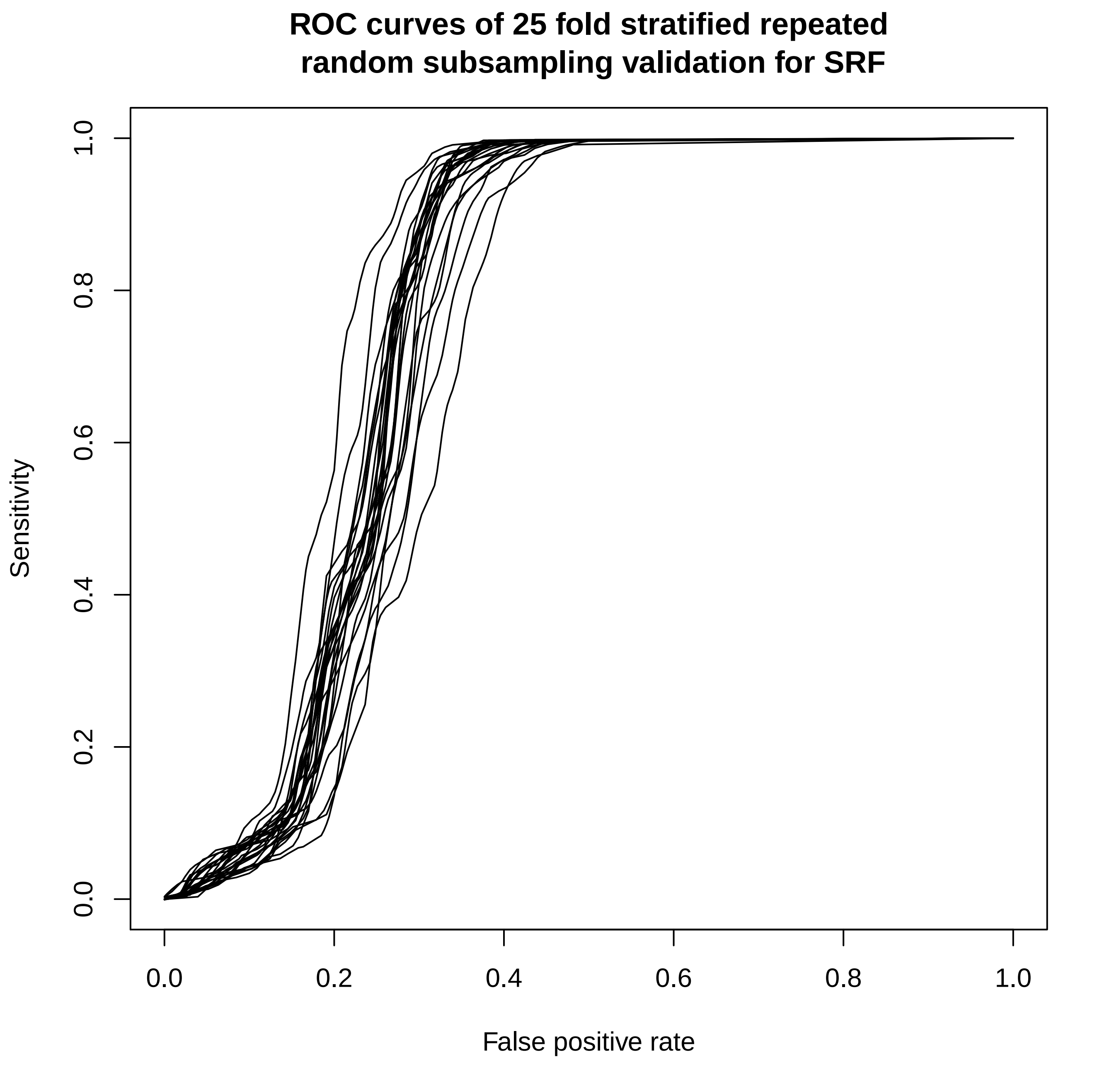
ROC curves of 25 fold stratified repeated
random subsampling validation for SRF
Sensitivity
False positive rate
0.0
0.2
0.4
0.6
0.8
1.0

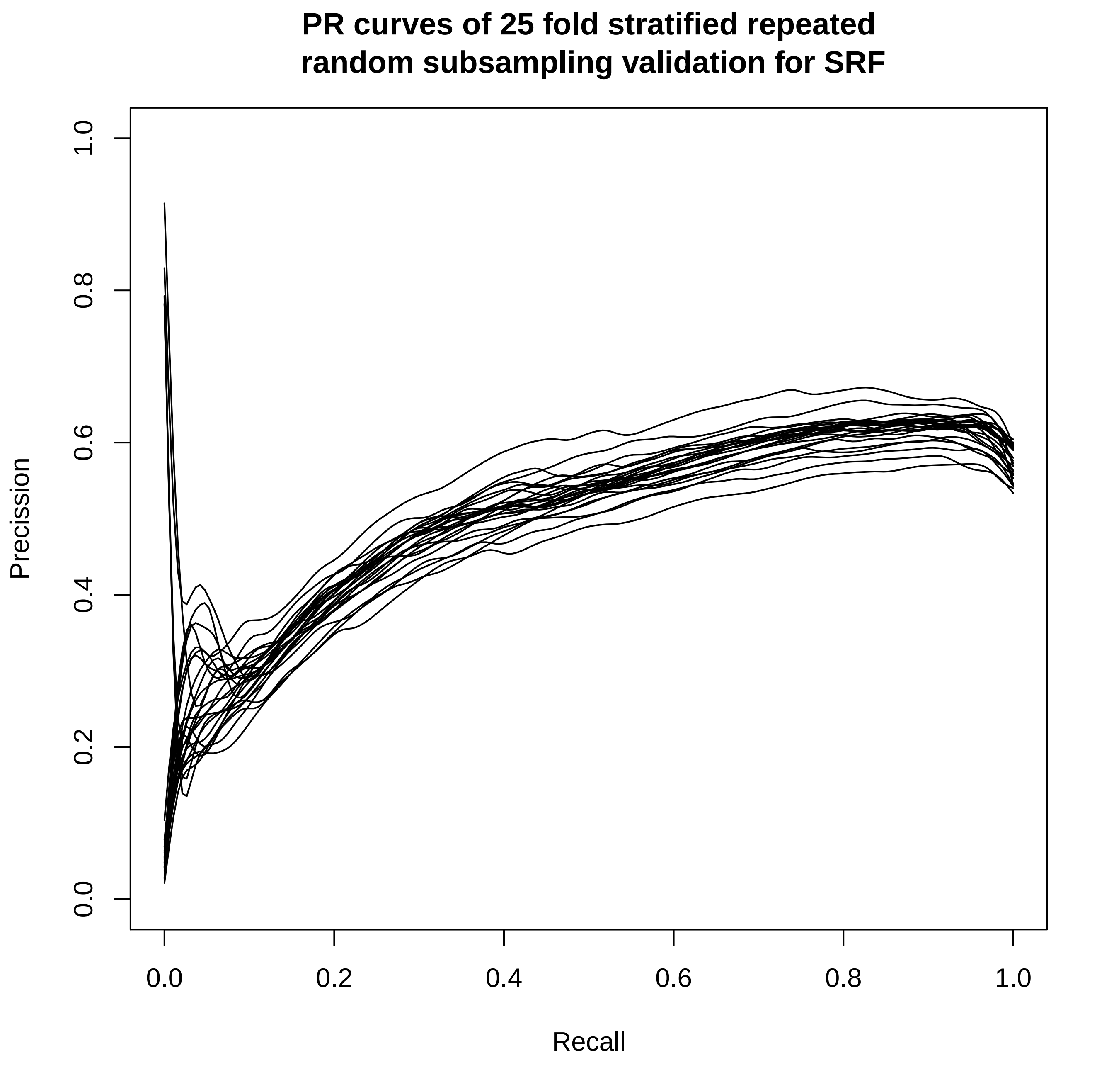
PR curves of 25 fold stratified repeated random subsampling validation for SRF
Precission
Recall
0.0
0.2
0.4
0.6
0.8
1.0

ROC curves of 25 fold stratified repeated random subsampling validation for TCF12

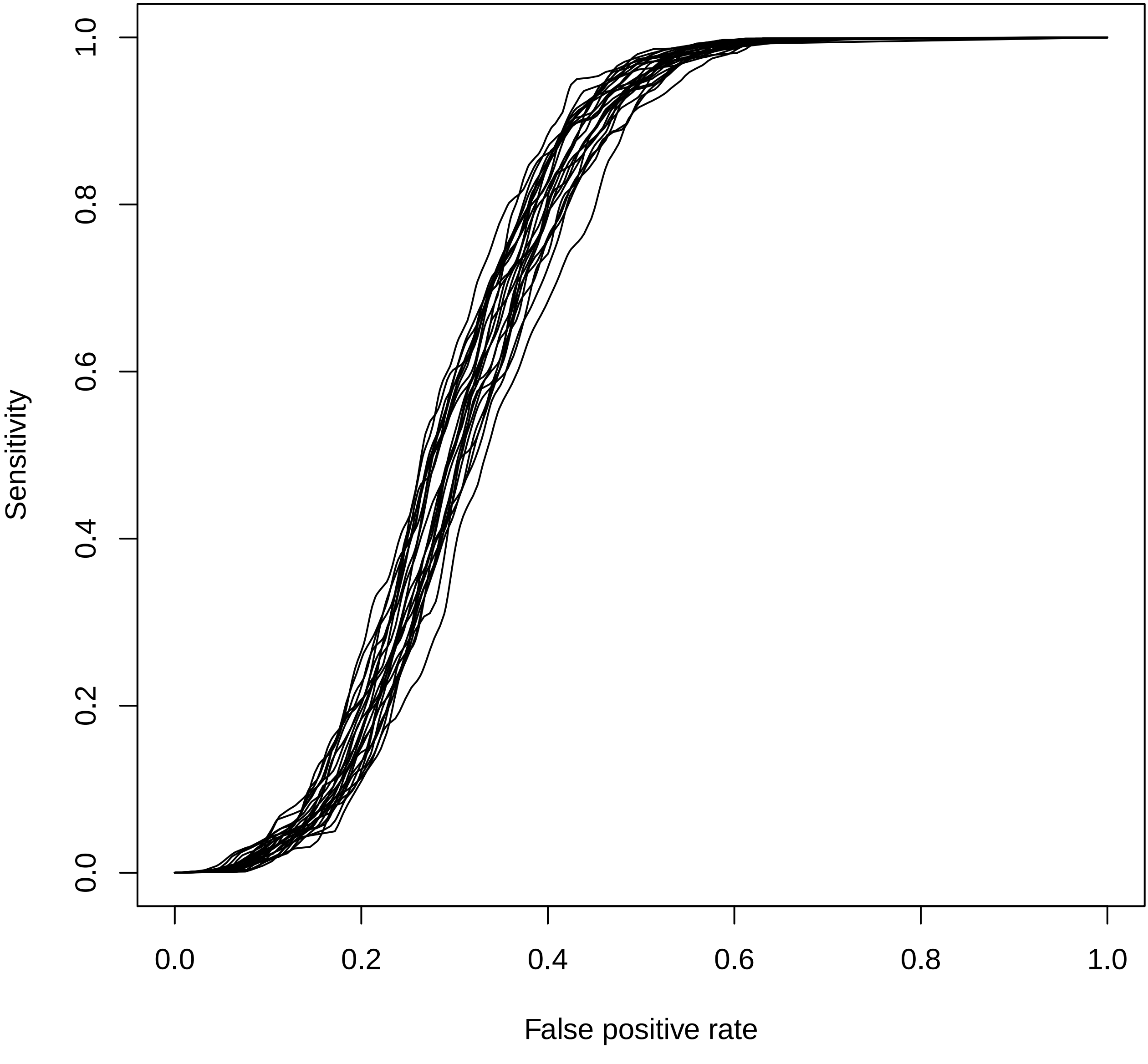

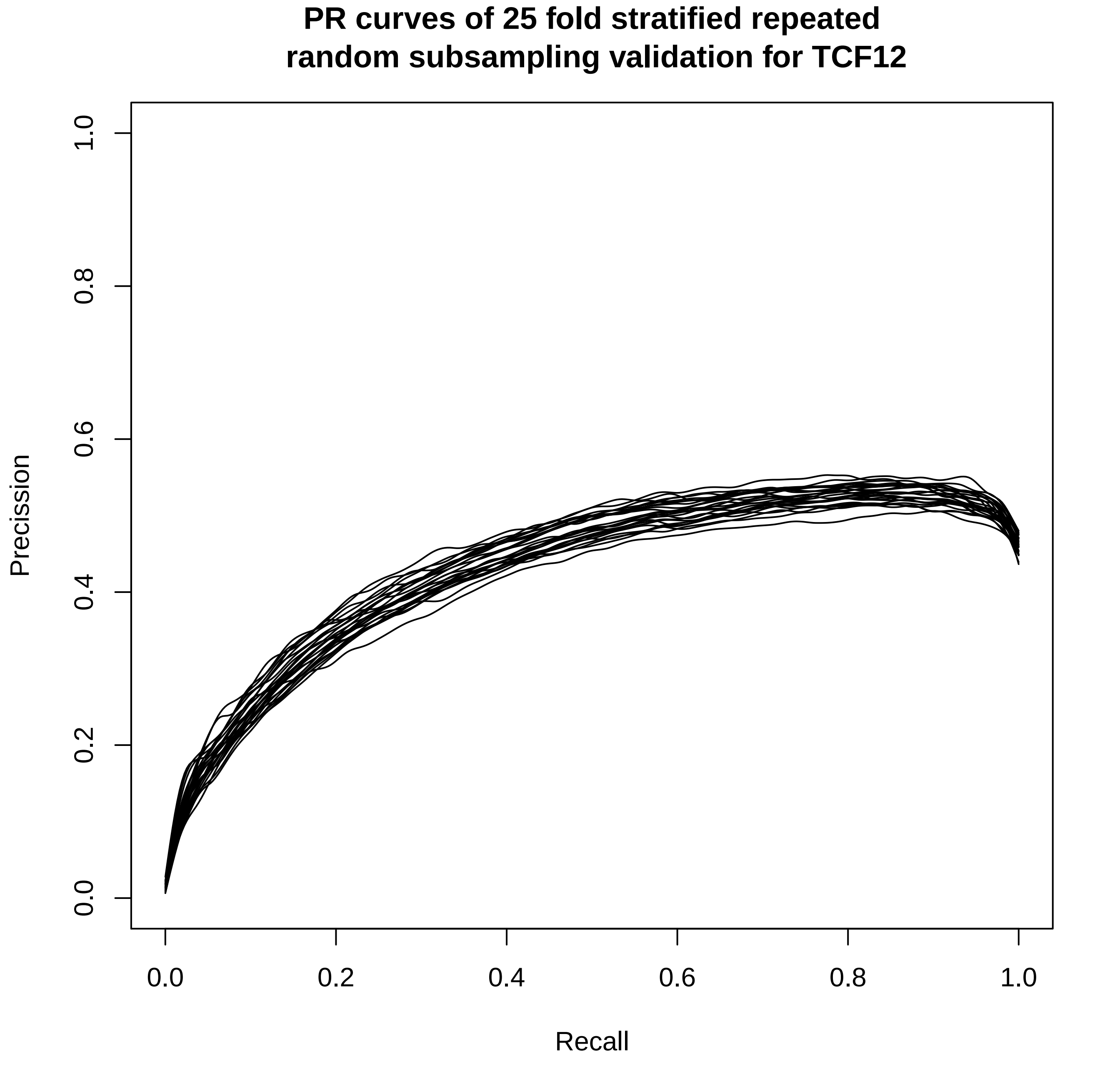

PR curves of 25 fold stratified repeated
random subsampling validation for TCF12
Precission
Recall
0.0
0.2
0.4
0.6
0.8
1.0

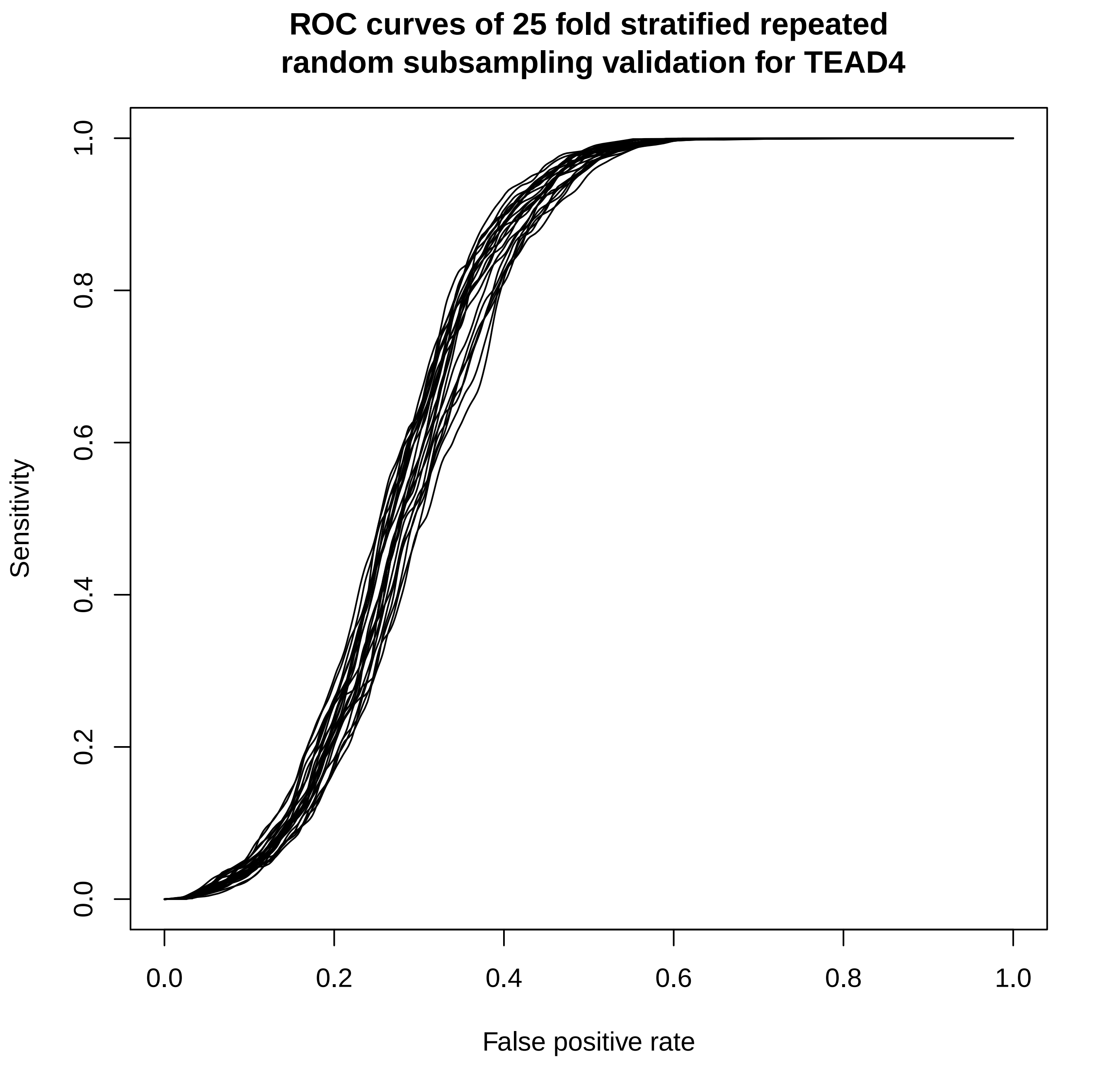

ROC curves of 25 fold stratified repeated random subsampling validation for TEAD4
Sensitivity
False positive rate
0.0
0.2
0.4
0.6
0.8
1.0

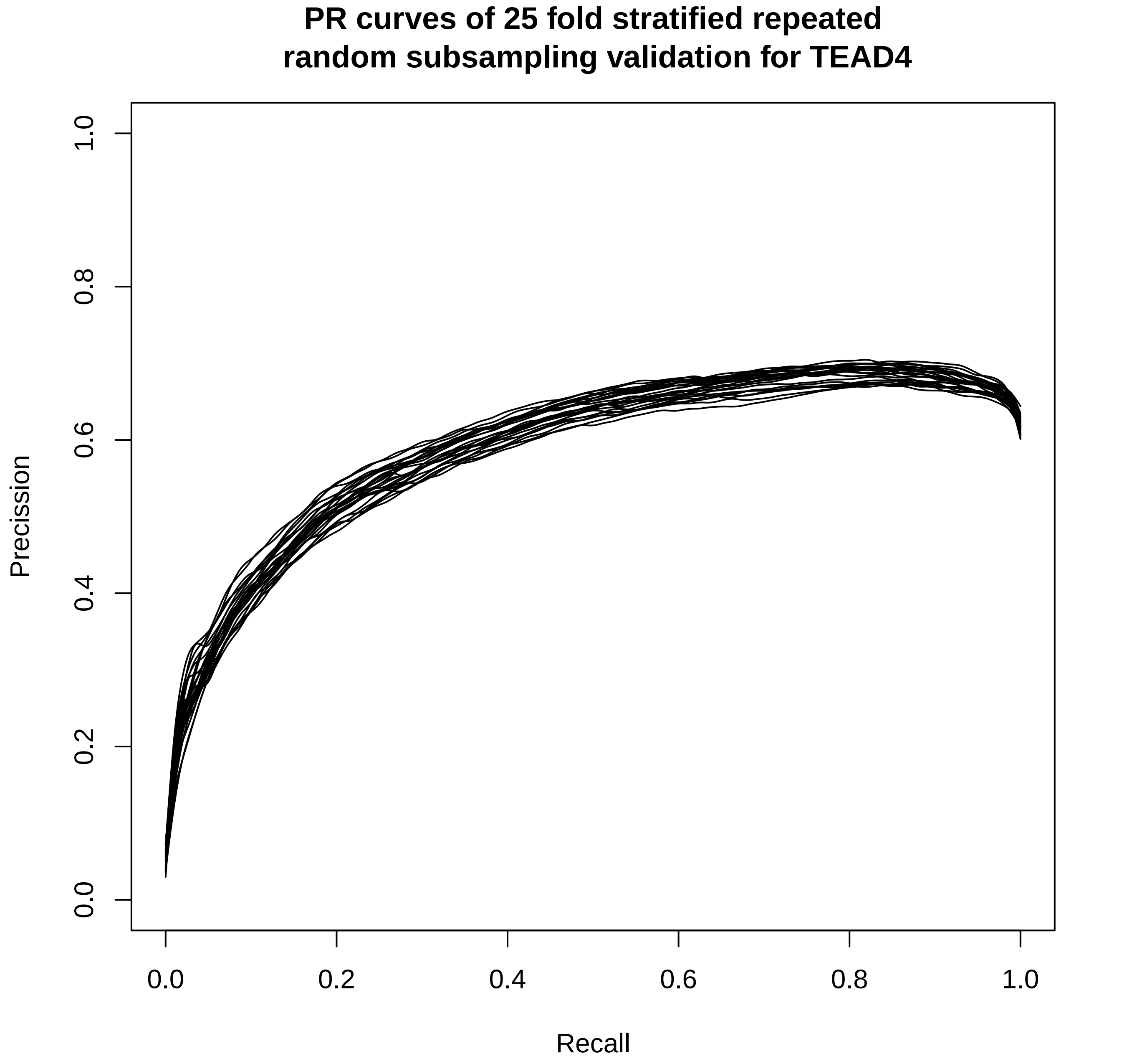
PR curves of 25 fold stratified repeated
random subsampling validation for TEAD4
Precission
Recall
0.0
0.2
0.4
0.6
0.8
1.0

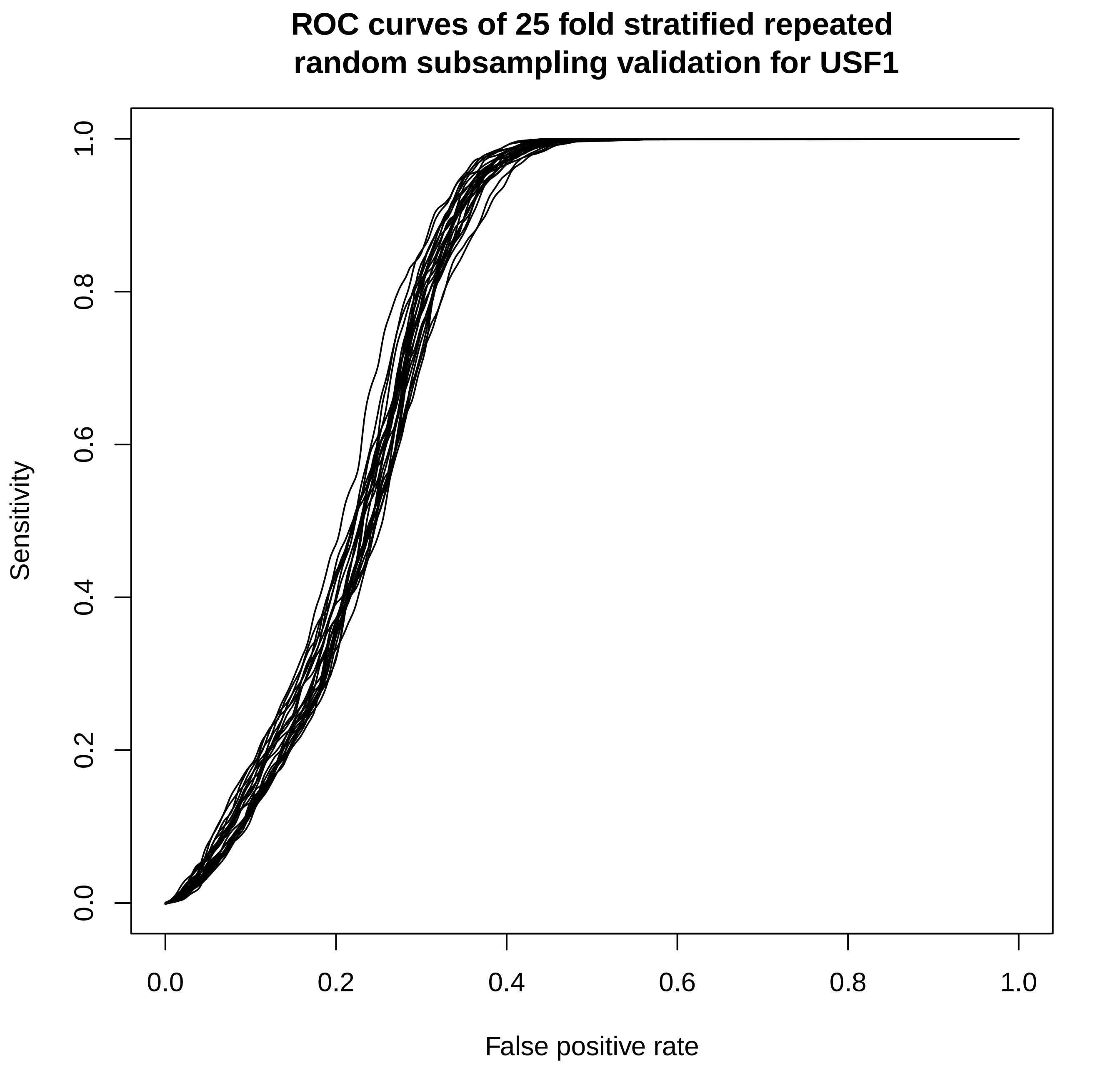
ROC curves of 25 fold stratified repeated random subsampling validation for USF1
Sensitivity
0.0
0.2
0.4
0.6
0.8
1.0
False positive rate
0.0
0.2
0.4
0.6
0.8
1.0

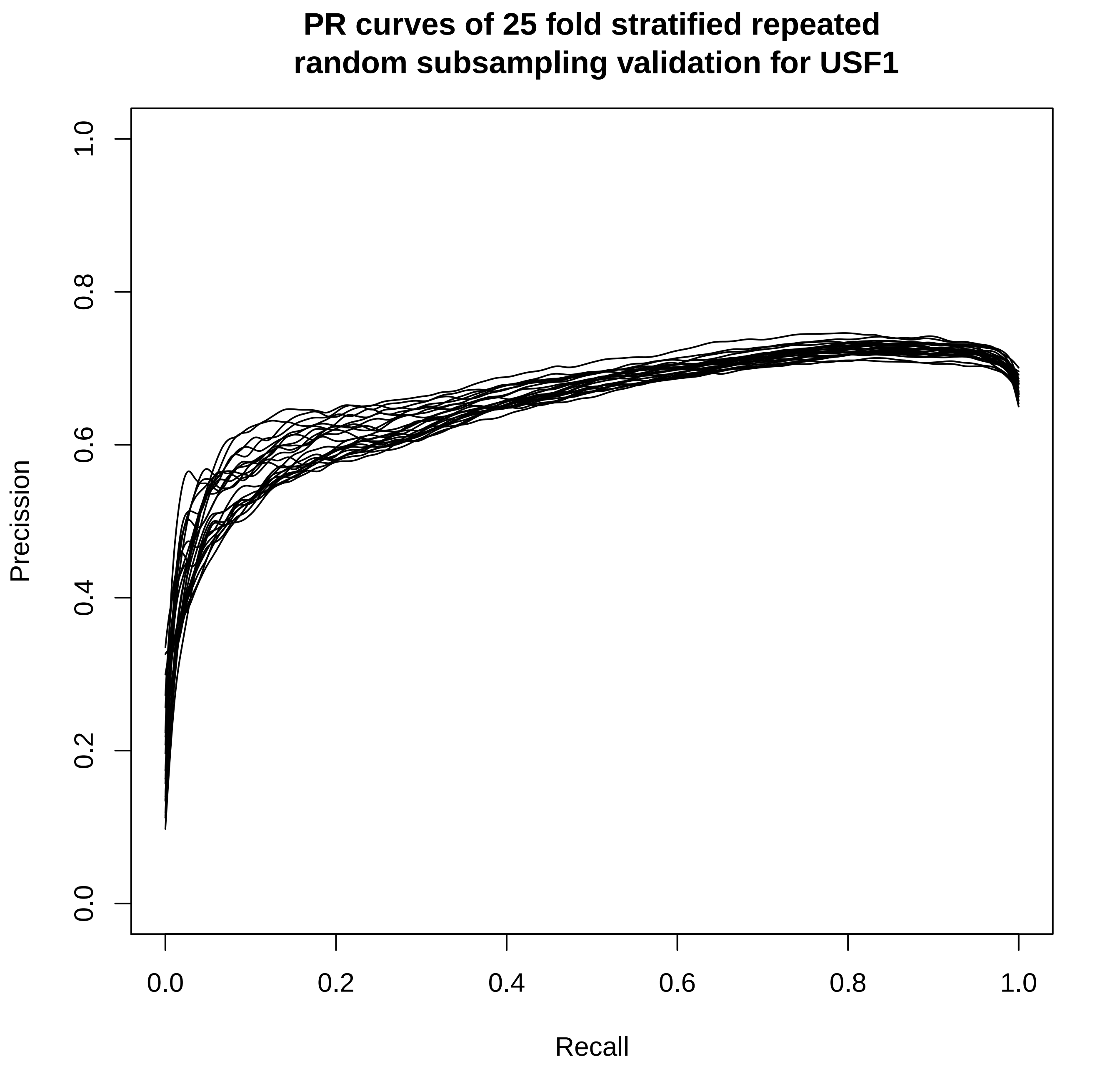
PR curves of 25 fold stratified repeated
random subsampling validation for USF1
Precission
Recall
0.0
0.2
0.4
0.6
0.8
1.0

ROC curves of 25 fold stratified repeated random subsampling validation for USF2

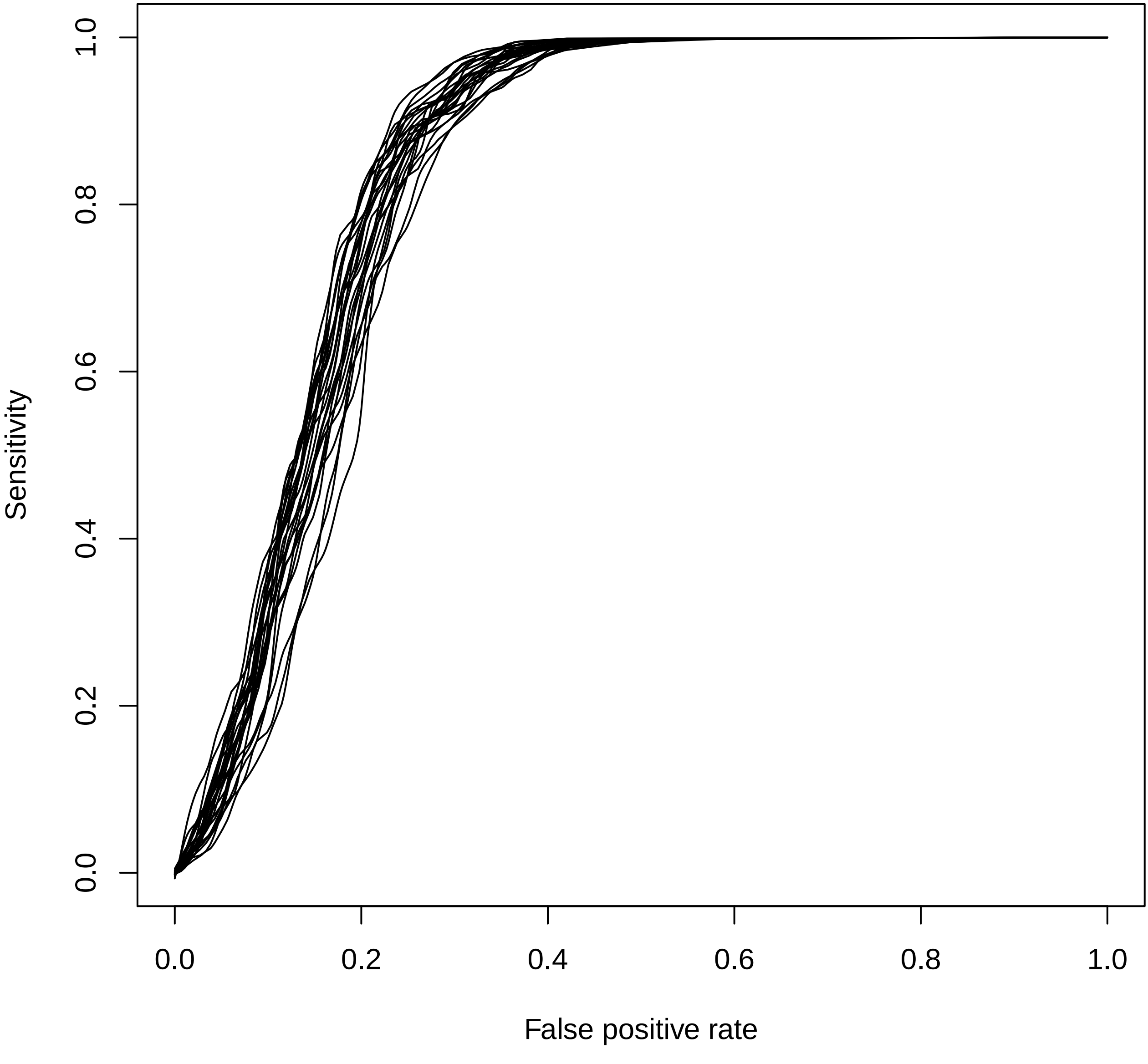

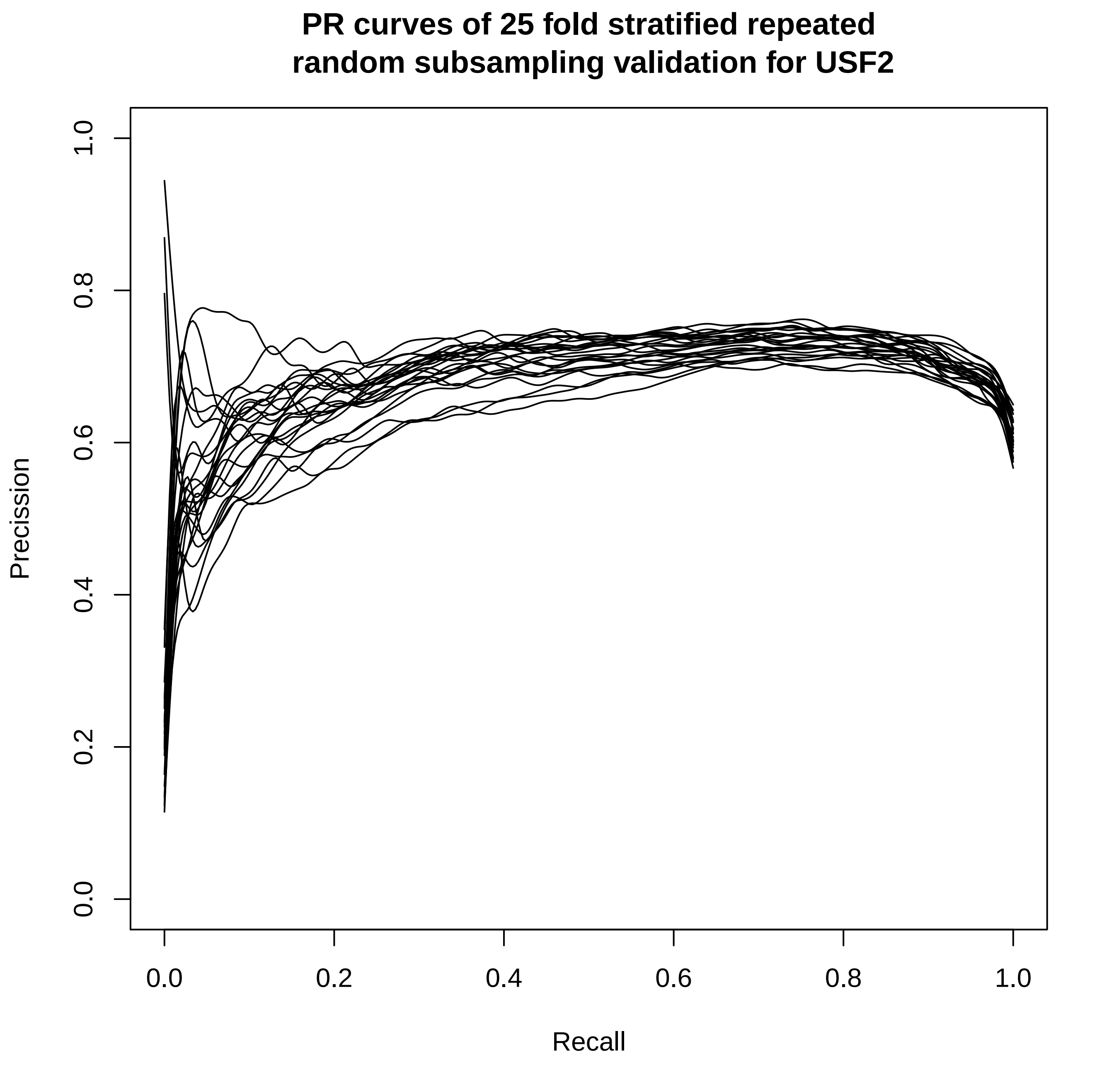
PR curves of 25 fold stratified repeated
random subsampling validation for USF2
Precission
Recall
0.0
0.2
0.4
0.6
0.8
1.0

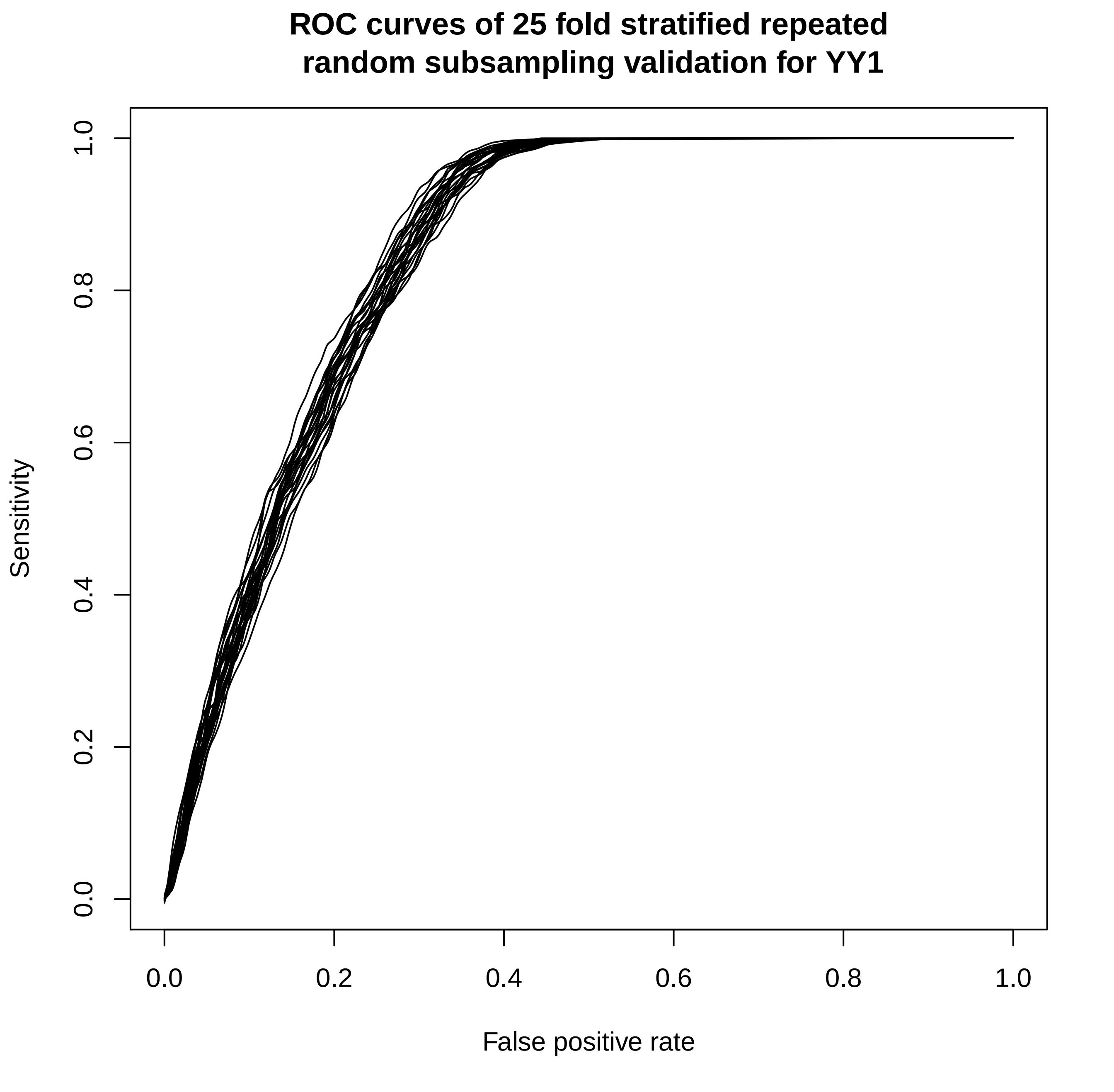

ROC curves of 25 fold stratified repeated
random subsampling validation for YY1
Sensitivity
0.0
0.2
0.4
0.6
0.8
1.0
False positive rate

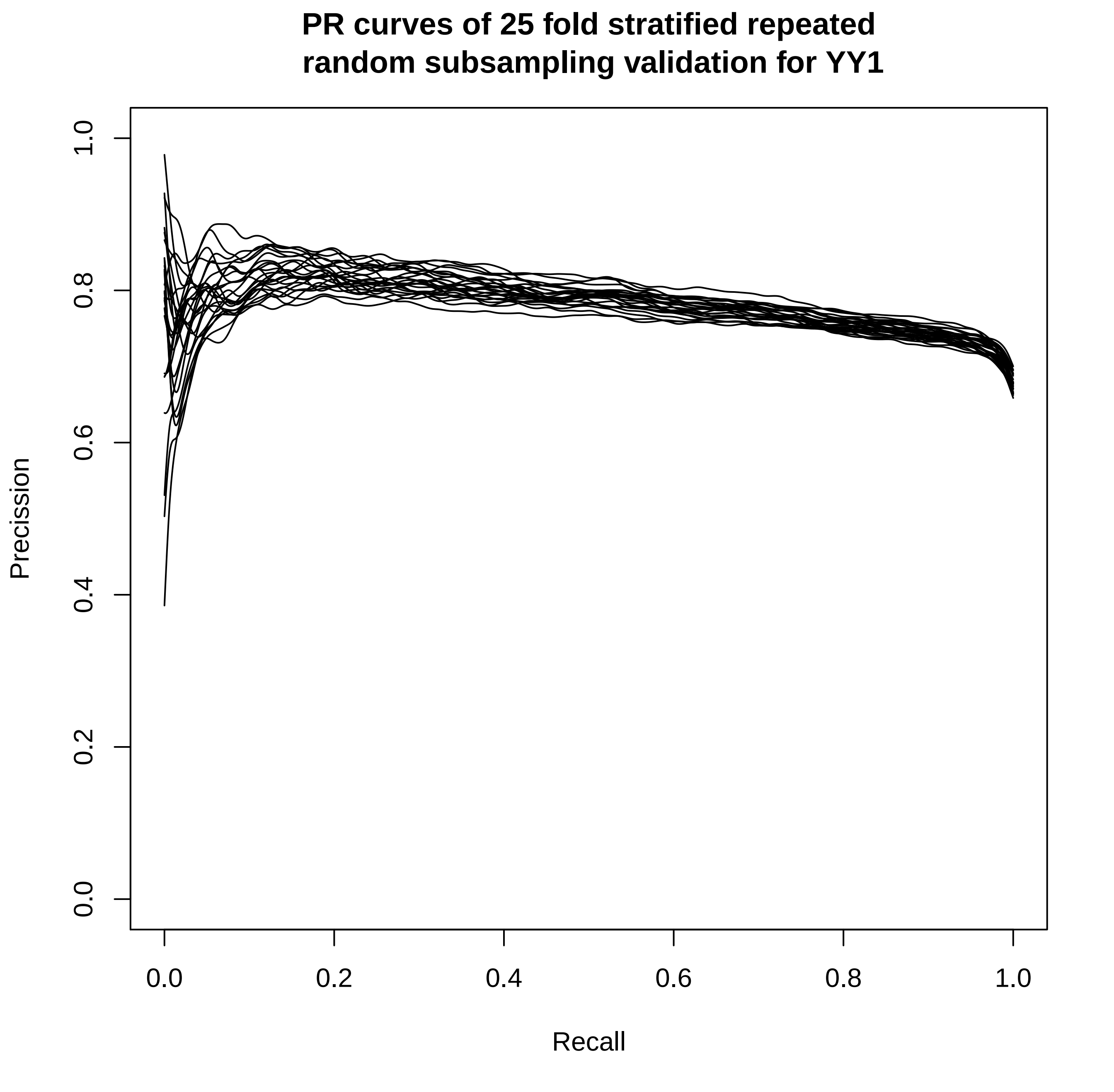
PR curves of 25 fold stratified repeated
random subsampling validation for YY1
Precission
Recall
0.0
0.2
0.4
0.6
0.8
1.0

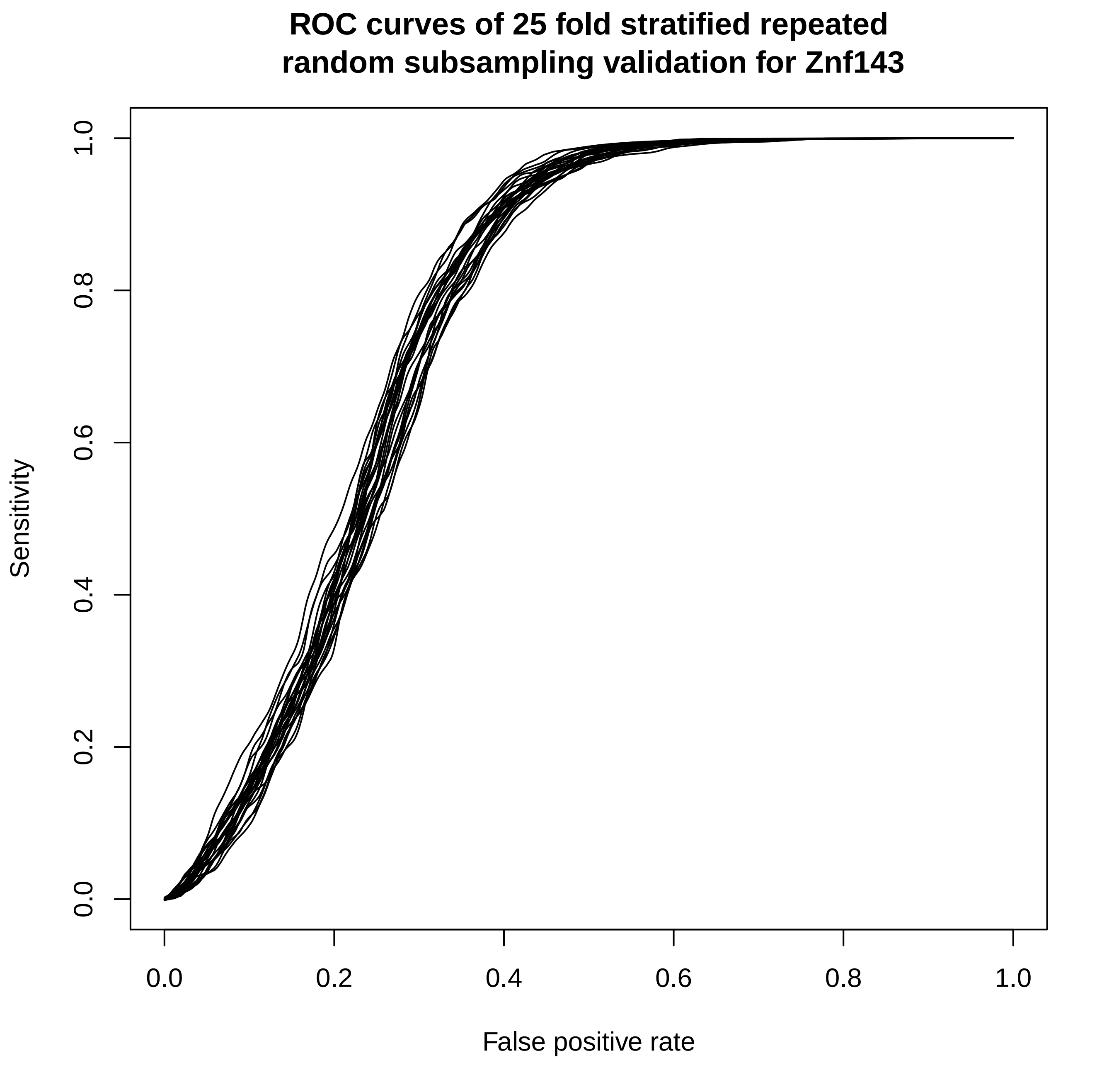
ROC curves of 25 fold stratified repeated random subsampling validation for Znf143
Sensitivity
0.0
0.2
0.4
0.6
0.8
1.0
False positive rate
0.0
0.2
0.4
0.6
0.8
1.0

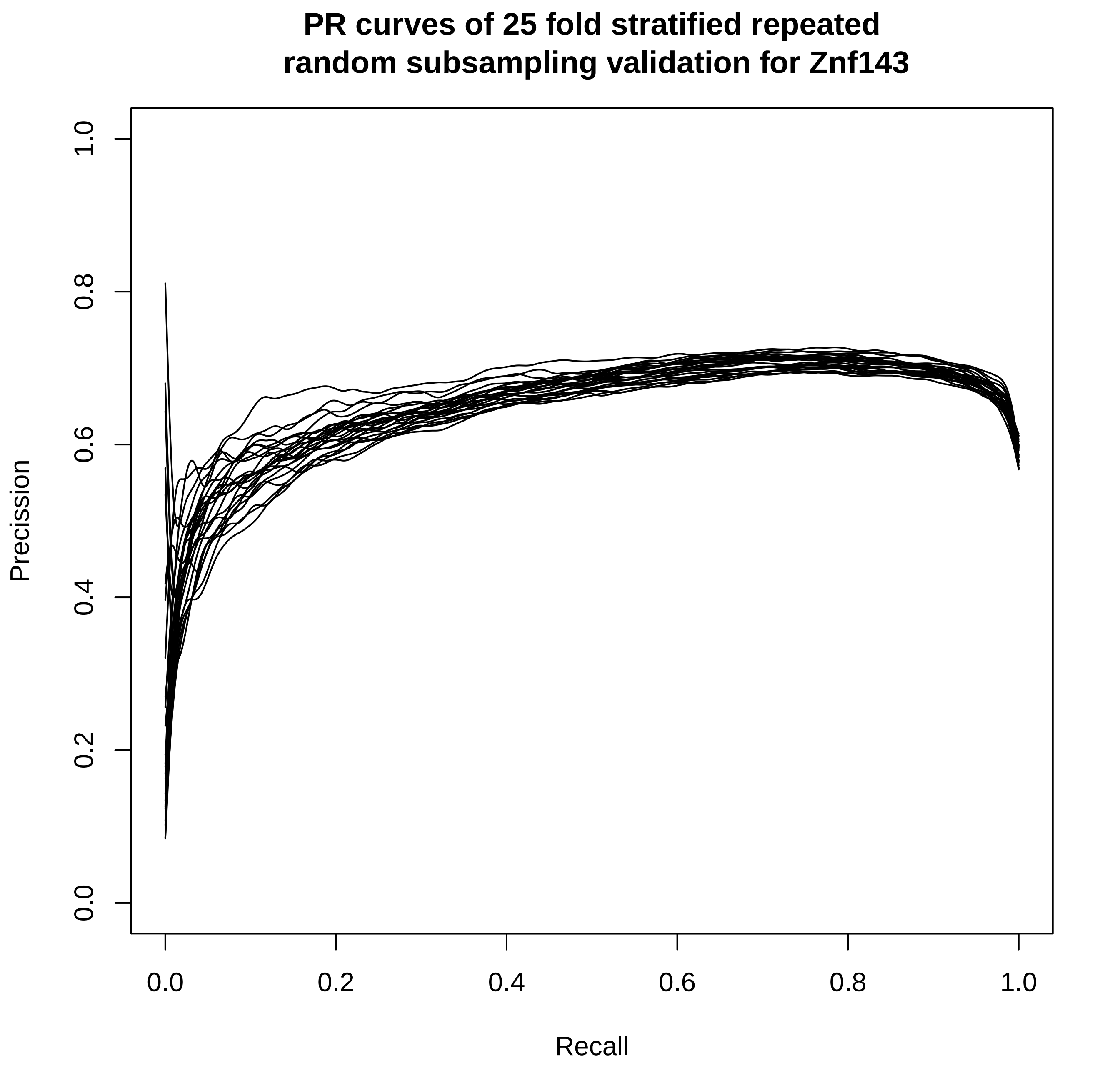
PR curves of 25 fold stratified repeated
random subsampling validation for Znf143
Precission
Recall
0.0
0.2
0.4
0.6
0.8
1.0
